# Genomic insights into endangerment and conservation of the garlic-fruit tree (*Malania oleifera*), a plant species with extremely small populations

**DOI:** 10.1093/gigascience/giae070

**Published:** 2024-09-23

**Authors:** Yuanting Shen, Lidan Tao, Rengang Zhang, Gang Yao, Minjie Zhou, Weibang Sun, Yongpeng Ma

**Affiliations:** Yunnan Key Laboratory for Integrative Conservation of Plant Species with Extremely Small Populations, Kunming Institute of Botany, Chinese Academy of Sciences, Kunming 650201, China; Key Laboratory for Plant Diversity and Biogeography of East Asia, Kunming Institute of Botany, Chinese Academy of Sciences, Kunming 650201, China; University of Chinese Academy of Sciences, Beijing 100049, China; State Key Laboratory of Plant Diversity and Specialty Crops, Institute of Botany, Chinese Academy of Sciences, Beijing 100093, China; Yunnan Key Laboratory for Integrative Conservation of Plant Species with Extremely Small Populations, Kunming Institute of Botany, Chinese Academy of Sciences, Kunming 650201, China; Key Laboratory for Plant Diversity and Biogeography of East Asia, Kunming Institute of Botany, Chinese Academy of Sciences, Kunming 650201, China; University of Chinese Academy of Sciences, Beijing 100049, China; Yunnan Key Laboratory for Integrative Conservation of Plant Species with Extremely Small Populations, Kunming Institute of Botany, Chinese Academy of Sciences, Kunming 650201, China; Key Laboratory for Plant Diversity and Biogeography of East Asia, Kunming Institute of Botany, Chinese Academy of Sciences, Kunming 650201, China; University of Chinese Academy of Sciences, Beijing 100049, China; Yunnan Key Laboratory for Integrative Conservation of Plant Species with Extremely Small Populations, Kunming Institute of Botany, Chinese Academy of Sciences, Kunming 650201, China; Key Laboratory for Plant Diversity and Biogeography of East Asia, Kunming Institute of Botany, Chinese Academy of Sciences, Kunming 650201, China; Yunnan Key Laboratory for Integrative Conservation of Plant Species with Extremely Small Populations, Kunming Institute of Botany, Chinese Academy of Sciences, Kunming 650201, China; University of Chinese Academy of Sciences, Beijing 100049, China; Yunnan Key Laboratory for Integrative Conservation of Plant Species with Extremely Small Populations, Kunming Institute of Botany, Chinese Academy of Sciences, Kunming 650201, China; Key Laboratory for Plant Diversity and Biogeography of East Asia, Kunming Institute of Botany, Chinese Academy of Sciences, Kunming 650201, China; Yunnan Key Laboratory for Integrative Conservation of Plant Species with Extremely Small Populations, Kunming Institute of Botany, Chinese Academy of Sciences, Kunming 650201, China; Key Laboratory for Plant Diversity and Biogeography of East Asia, Kunming Institute of Botany, Chinese Academy of Sciences, Kunming 650201, China

**Keywords:** recent inbreeding, deleterious mutation, demographic history, genomic offset, ecological niche modeling, conservation genomics

## Abstract

**Background:**

Advanced whole-genome sequencing techniques enable covering nearly all genome nucleotide variations and thus can provide deep insights into protecting endangered species. However, the use of genomic data to make conservation strategies is still rare, particularly for endangered plants. Here we performed comprehensive conservation genomic analysis for *Malania oleifera*, an endangered tree species with a high amount of nervonic acid. We used whole-genome resequencing data of 165 samples, covering 16 populations across the entire distribution range, to investigate the formation reasons of its extremely small population sizes and to evaluate the possible genomic offsets and changes of ecology niche suitability under future climate change.

**Results:**

Although *M. oleifera* maintains relatively high genetic diversity among endangered woody plants (θ_π_ = 3.87 × 10^−3^), high levels of inbreeding have been observed, which have reduced genetic diversity in 3 populations (JM, NP, and BM2) and caused the accumulation of deleterious mutations. Repeated bottleneck events, recent inbreeding (∼490 years ago), and anthropogenic disturbance to wild habitats have aggravated the fragmentation of *M. oleifera* and made it endangered. Due to the significant effect of higher average annual temperature, populations distributed in low altitude exhibit a greater genomic offset. Furthermore, ecological niche modeling shows the suitable habitats for *M. oleifera* will decrease by 71.15% and 98.79% in 2100 under scenarios SSP126 and SSP585, respectively.

**Conclusions:**

The basic realizations concerning the threats to *M. oleifera* provide scientific foundation for defining management and adaptive units, as well as prioritizing populations for genetic rescue. Meanwhile, we highlight the importance of integrating genomic offset and ecological niche modeling to make targeted conservation actions under future climate change. Overall, our study provides a paradigm for genomics-directed conservation.

## Introduction

Historical climate disturbances and frequent human activity have caused many species that were once widespread with continuous distributions to become small, fragmented populations [1]. High levels of inbreeding continually occur in these populations, leading to the accumulation of deleterious mutations and low species adaptability, ultimately increasing the risk of extinction [2, 3]. The genome contains evolutionary footprints that can be used to estimate inbreeding levels of species even without detailed pedigrees [4, 5]. For example, runs of homozygosity (ROH), genome regions with a certain length that is identical by descent, have been widely used as an indicator of inbreeding [6, 7]. The long ROH indicate a closer relationship to the most recent common ancestor, implying a higher level of inbreeding. For small and isolated populations with high inbreeding levels, genetic rescue is necessary to introduce beneficial mutations by establishing gene flow between populations [8]. However, caution should be taken when making decisions regarding genetic rescue, and comprehensive exploration of the genetic background of these small populations must be done in advance [9, 10].

Rapid climate change in the future is a widely recognized threat to global biodiversity [11–13]. The threatened degree of species depends on how they respond to climate change. There are 2 main mechanisms: migrating to new habitats or adapting to the changing environments through phenotypic plasticity or *de novo* mutations [14–16]. However, in the case of long-lived forest trees, individual organisms are nearly incapable of migrating to keep pace with changing climate and may experience maladaptation throughout their lifetimes [17–19]. Thus, if species’ responses to future climate change can be predicted, their extinction risks can be estimated, and targeted conservation guidelines and management strategies can be developed in advance.

*Malania oleifera* Chun & S. K. Lee (NCBI:txid397392), the single species in the genus *Malania* (Olacaceae), is an endemic, semi-parasitic evergreen tree (diploid) that naturally scattered in the west Guangxi (a.s.l. 300–1,000 m) and southeast Yunnan province, China (a.s.l. 300–1,640 m) [20]. It adapts well to rocky desert habitats and can be used as an afforestation tree in karst landscapes [21]. Moreover, it has extremely high economic and medicinal value due to the large amount of lipids in its seed. The main lipid component is nervonic acid (*cis*-tetracos-15-enoic acid, >60%), which is essential for human nervous health [22]. However, mainly due to overexploitation, wild resources of *M. oleifera* have decreased by approximately 25,000 individuals between 2000 and 2017 solely in Guangnan County (Yunnan, China) [23, 24]. Additionally, physiological factors of *M. oleifera*, including large seed size, short seed life span, difficulty in natural seed germination, low rate of pollen germination, and susceptibility to root rot, have made natural propagation and regeneration difficult [25–28]. Therefore, *M. oleifera* has been categorized as Vulnerable (VU) on the IUCN Red List (https://www.iucnredlist.org/species/32361/9701100) and recorded in the Class II Key Protected Wild Plant List in China [29], and it has also been listed as a plant species with an extremely small population size in China [30], highlighting the urgent need for its conservation.

Previous studies related to *M. oleifera* mainly focused on the biosynthetic pathway of nervonic acid [22, 31] and exploring the optimal condition of growing artificial seedlings for better utilization [32, 33]. However, the conservation process of *M. oleifera* is limited to *in situ* protection of existing wild resources [34]. Recent advancements in whole-genome sequencing techniques enable covering nearly all the nucleotide variations of a genome and can provide deep insights into protecting endangered species [35]. However, there is a gap in using genomic data to guide conservation strategies, particularly for plants. Therefore, we utilize *M. oleifera* as a study case to investigate the above issues. We aimed to provide a comprehensive framework for the *M. oleifera* conservation through the perspective of conservation genomics.

## Materials and Methods

### Sample collection and whole-genome resequencing

**Figure 1:**
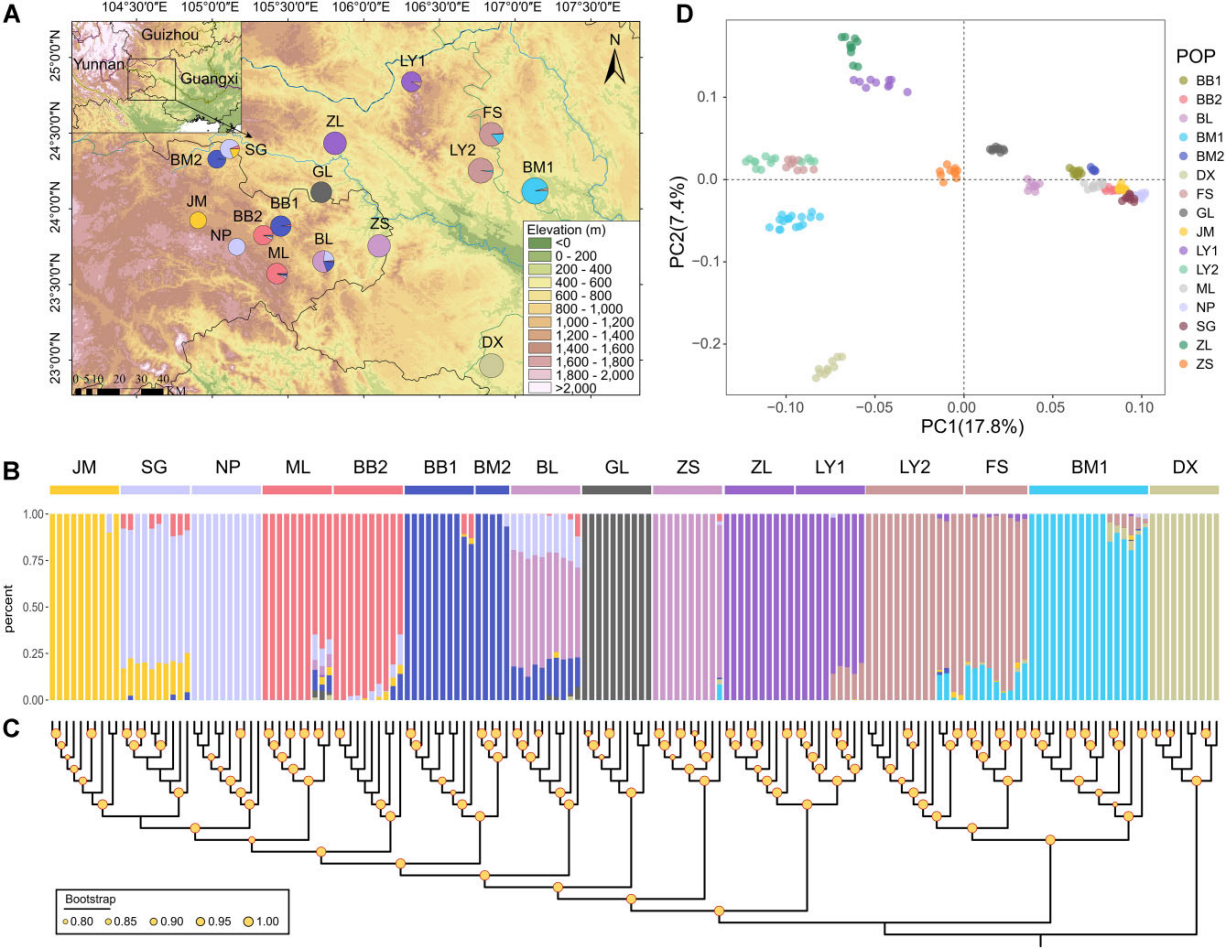
Population genomics of *M. oleifera*. (A) Geographic distribution and sampled populations of *M. oleifera*. Different colors in the pie chart represent the genetic groups identified by ADMIXTURE based on adaptive loci, and the size of the pie corresponds to the level of heterozygosity. The optimal population genetic structure of *M. oleifera* with K = 10 (B) and a neighbor-joining (NJ) phylogenetic tree (C) based on adaptive loci. Samples in the STRUCTURE and phylogenetic tree results correspond. Node bootstrap values below 0.8 are not shown. (D) Results of PCA based on adaptive loci, with the first 2 PCs explaining 25.2% of the genome covariance. Populations are defined as BB1 = Banbeng, BB2 = Babao, BL = Banlun, BM1 = Bama, BM2 = Bamei, DX = Daxin, FS = Fengshan, GL = Gaolong, JM = Jiumo, LY1 = Leye, LY2 = Linyun, ML = Mulun, NP = Nanping, SG = Shuguang, ZL = Zhemiao, and ZS = Zhesang.

A total of 165 leaf samples were collected from 16 wild populations across the entire distribution of *M. oleifera* from Yunnan and Guangxi provinces, China (Fig. 1A; Supplementary Table S1). Among them, 76 samples were collected based on our field investigation, and the remaining 89 samples were obtained from the Germplasm Bank of Wild Species in Southwest China. The number of individuals collected per population (including the samples from germplasm bank) varied from 5 to 17, and if populations contained fewer than 10 individuals, all individuals were sampled.

Genomic DNA was extracted from silica-dried leaf tissues using a modified CTAB method [36], and the concentration and quality of the DNA was determined using a NanoDrop2000 Spectrophotometer (Thermo Fisher Scientific). Samples were sent to Beijing Ori-Gene Science and Technology Co., Ltd for Illumina sequencing library preparation according to the manufacturer’s specifications. Paired-end raw reads (150 bp) were generated on the Illumina HiSeq platform.

### Read mapping and single nucleotide polymorphism calling

The raw data were filtered using Fastp (RRID:SCR_016962) v. 0.19.3 [37]. Paired-end clean reads were mapped to the chromosome-level genome of *M. oleifera* (∼1.5 Gb) [31] using BWA-MEM (RRID:SCR_022192) v. 2.1 [38]. SAMtools (RRID:SCR_002105) v. 1.9 [39] was used to convert sequence alignment map (SAM) format files to sorted binary alignment map (BAM) format files. Sambamba (RRID:SCR_024328) v.0.7.1 [40] was used to mark and remove duplicate reads. Freebayes (RRID:SCR_010761) v. 1.3.6 [41] was employed to call variants, and only bases with a quality score ≥20 and reads with a mapping quality score ≥30 were included. This produced a total of 43,413,408 initial variant sites. We then employed VCFtools (RRID:SCR_001235) v. 0.1.15 [42] to filter sites with the following criteria: (i) sites with coverage depth below 1/2 * average site coverage and above 2 * average site coverage were discarded after investigating the coverage distribution; (ii) sites located on the organelle genomes or contigs that were not anchored on the chromosomes were excluded; (iii) single nucleotide polymorphisms (SNPs) with a depth below 3× or a genotype quality score <20 were redefined as missing; (iv) only biallelic SNPs were reserved; (v) SNPs with a missing rate >20% were removed, leaving 2,144,506 SNPs (dataset 1); and (vi) SNPs with a minor allele frequency <0.05 were all excluded. Finally, 250,362 SNPs remained (dataset 2), which distributed on 13 pseudochromosomes (Supplementary Fig. S2).

### Population genetic diversity and runs of homozygosity

Based on dataset 2, we detected a genome-wide linkage disequilibrium (LD) decay among 16 populations using PopLDdecay (RRID:SCR_022509) v. 3.4.0 [43]. Nucleotide diversity (θ_π_), Watterson’s θ (θ_w_), and heterozygosity rate were calculated using ANGSD (RRID:SCR_021865) v. 0.921 [44] based on bam files, which removed duplicates (see above). In addition, we calculated the values of the 3 parameters in more specific genomic regions (intergenic, CDS, intron, fold-0, and fold-4). To examine inbreeding depression, we detected ROH using vcftools v. 0.1.15 [42] based on dataset 2 with the key parameters “–LROH”, and only ROH longer than 100 kb were kept. Moreover, we calculated the frequency of runs of homozygosity (FROH), which is equal to the sum of all ROH lengths longer than 100 kb divided by genome effective length [45].

### Inference of population structure based on all loci, adaptive loci, and neutral loci

We employed 2 software to detect outlier SNPs potentially related to adaptive evolution. First, we used the sparse nonnegative matrix factorization (snmf) function applied in the R package LEA (RRID:SCR_009090) v. 3.1.4 [46] to estimate the most likely number of ancestral populations based on dataset 2. To reduce the number of false positives, we reserved SNPs with the false discovery rate (FDR) less than 0.01. Second, we applied a principal component analysis (PCA) method using R package Pcadapt (RRID:SCR_022019) v. 4.3.3 [47] with a 0.01 cutoff of FDR to identify SNPs that highly influenced the formation of observed differentiation. SNPs detected by both methods were identified as potential adaptive SNPs; otherwise, they were considered as neutral SNPs. Finally, we employed PLINK (RRID:SCR_001757) v. 1.90b4.1 [48] to filter out linkage disequilibrium sites and ultimately obtained 33,971 (dataset 3, all loci), 1,515 (dataset 4, adaptive loci), and 32,930 (dataset 5, neutral loci) SNPs for downstream analysis (Supplementary Fig. S1).

Based on the 3 datasets described above, we employed ADMIXTURE (RRID:SCR_001263) v. 1.3.0 [49] to infer the population structure. The most likely population number of K was determined by the minimizing cross-validation error. PCA was conducted in GCTA v1.94.1 [50] and MEGA (RRID:SCR_000667) v. 7.0 [51] was used to construct NJ trees. Pairwise fixation statistics (F_st_) among the 16 populations were calculated using vcftools v. 0.1.15 [42].

### Estimation of demographic history

We employed Stairway Plot v.2 [52] and MSMC (RRID:SCR_023677) v.2 [53] to infer the population demographic history of *M. oleifera*. For the analysis of the stairway plot, we first performed ancestral sequence reconstruction to infer ancestral status (see details in Supplementary Note S1 and Supplementary Table S16). To mitigate the effects of selection, we excluded the upstream and downstream 5-kb regions of genes to infer folded site frequency spectrum (SFS) and unfolded SFS using ANGSD v. 0.921 [44]. We set the average generation time of *M. oleifera* as 10 years because it takes about 10 years for a seed to grow into a seed-producing plant according to our field observations. The mutation rate was set to 2.5 × 10^−8^ per site per generation (see details in Supplementary Note S2 and Supplementary Table S17). MSMC is a multiple sequentially Markovian coalescent approach that uses the density of heterozygous sites to estimate the effective population size (*Ne*) through time. Different individual numbers or haplotypes provide distinct resolutions for the analysis of demographic histories. Therefore, we employed MSMC to separately estimate the coalescence rate within 2, 4, and 8 haplotypes, referring to the simulation results of Schiffels and Durbin [53]. A total of 100 random combinations of individuals were used for the 3 haplotype analyses to estimate the medians and 95% confidence interval values. The average generation time and mutation rate values were set to the same as stairway plot analysis.

### Detection of deleterious mutations

Deleterious mutations in *M. oleifera* were predicted using the Sorting Intolerant From Tolerant (SIFT) algorithm [54]. We used a modified approach to perform the SIFT prediction (see Supplementary Note S3). The TrEMBL plant database [55] was used to search for orthologous genes, and the SIFT scores were calculated based on the degree of conservation among loci. Based on dataset 1 (included low-frequency variants), SNPs in the coding regions were categorized as deleterious (SIFT score <0.05), tolerated (SIFT score ≥0.05), or synonymous using SIFT4G (RRID:SCR_021850) [56]. The low confidence sites and “NA” sites were not considered.

To provide accurate and direct genetic rescue guidance for *M. oleifera* populations with a high genetic load, we selected 5 populations, including 1 as the potentially threatened population and 4 as the candidate pollen donors. We drew a Venn diagram to explore the distribution of shared or unique homozygous deleterious mutations among the 5 populations. The 4-candidate pollen donors were characterized by (i) having low genetic load, low levels of inbreeding, high genetic diversity, and high heterozygosity; (ii) low genetic differentiation with the rescued population; or (iii) sharing the same genetic lineage as the rescued population based on adaptive loci.

### Identification of environment-associated adaptive variants

The environmental data, which included 19 bioclimatic variables at 2.5-minute resolution (5 km), were downloaded from WorldClim v.2.1 database (RRID:SCR_010244) (Supplementary Table S9). Each environmental factor was extracted through the coordinates of sampling points. We selected the top climatic factors based on weighted *R*^2^ importance (Supplementary Fig. S14) using a machine learning gradient forest (GF) model in the R package gradientforest v.0.1–37 [57] by modeling the relationship of climatic variables and SNPs with 500 regression trees. To avoid multicollinearity, we kept variables with |correlation coefficient| <0.7 by calculating Pearson’s correlation coefficient in the R package corrplot v.0.92 (RRID:SCR_024683) [58] and ultimately retained the 4 most important and uncorrelated factors, including BIO3 (Isothermality), BIO7 (Temperature Annual Range), BIO14 (Precipitation of the Driest Month), and BIO15 (Precipitation Seasonality). Next, we used BayeScEnv [59] and redundancy analysis (RDA) [60] to identify environment-associated SNPs. BayeScEnv represents a univariate genotype–environment association approach. For BayeScEnv method, the input files included an environmental factor standardized by the mean variance and contained codominant data, which converted by PGDSpider v. 2.1.1.5 [61] based on dataset 2. RDA is a multivariate linear regression-based method [62]. We ran RDA analysis in the R package vegan v.2.5–7 [63], using function “anova.cca” to check the significance of the RDA model and function “outliers” to identify local adaptation–associated SNPs that loaded in the tails of the ±3 standard deviation cutoff (2-tailed *P* = 0.0027). To recognize the gene functions of the candidate SNPs obtained from BayeScEnv and RDA, we employed a Gene Ontology enrichment analysis using the eggNOG-mapper (RRID:SCR_021165) v.2 [64].

### Genomic offset modeling with gradientforest

We used the GF model in the R package gradientforest v.0.1–37 [57] to predict genomic vulnerability to future climate change. The environmental-associated SNPs detected by both BayeScEnv and RDA were denoted as the candidate SNP dataset. In addition, 500 randomly selected SNPs based on dataset 2 were recorded as a reference SNP dataset to match the magnitude of candidate SNP dataset. The SNPs data with minor allele frequencies (MAFs) >10% were converted into MAFs per population. To ameliorate the linkage effect, we only kept 1 SNP per 100,000 bp range and finally obtained 326 reference SNPs and 213 candidate SNPs. We employed the GF model with 500 regression trees per SNP to build a function for the 4 most important environmental factors (BIO3, BIO7, BIO14, BIO15). Genomic offset (GO) was defined by Euclidean distance between current (1970–2000) and future (2081–2100) climate, which used the current condition as the baseline [16]. To explore possible future climate conditions and predict GO for *M. oleifera*, we employed 3 widely used global climate models (BCC-CSM2-MR, CNRM-CM6-1, and CNRM-ESM2-1) and 2 emission scenarios (SSP126 and SSP585) that represent the mild and extreme future carbon emissions. The predicted GO results of 3 global climate models for each grid were averaged with assigned weights.

### Ecological niche modeling

The distribution records of *M. oleifera* were collected from online databases, published academic articles [65, 66], and field investigation, and all records were manually verified using an online map. To reduce sampling bias, we kept only 1 record within 5 km using the rarefy function of the R package Humboldt [67], with 87 records remaining (Supplementary Table S10). The 19 bioclimatic variables (Supplementary Table S9) were also used in ecological niche modeling. Since background or pseudo-absence data of ecological niche models were sampled from the entire modeling map, we recalculated the correlation coefficients of 19 bioclimatic variables based on the whole distribution area. We removed autocorrelated variables (|Pearson’s *r|* >0.7 and variance inflation factor >10) using the R package usdm v.2.1 [68] and kept 6 uncorrelated variables (BIO1, BIO2, BIO7, BIO12, BIO14, and BIO18) for ecological niche modeling.

The ecological niche model (ENM) was built using an ensemble modeling method that combined outputs of 5 single models with high performance: GAM (generalized additive model by the R package mgcv v.1.9) [69], MaxEnt (tuned MaxEnt model by the R package dismo v.1.3) [70], RF (random forest with downsampling by the R package randomForest v.4.7) [71], Lasso (by the R package glmnet v.4.1) [72], and BRT (boosted regression trees by the R package dismo) [73]. Each model was run for 10 replicates, with pseudo-absence data of 10,000 points randomly generated using the R package Biomod2 [74] for 3 replicates, resulting in a total of 5 * 10 * 3 = 150 single models. However, only models with positive Somer’s *D* values were employed to create the final ensemble prediction, which was weighted by the true skill statistic (TSS) value of each model. The evaluation of the single model and ensemble model was performed by the R packages Ecospat v.4.0.0 [75] and prg v.0.5.1 [76].

The niche suitability of *M. oleifera* under future (2081–2100) carbon emission scenarios (SSP126 and SSP585) was predicted using the same climate models (BCC-CSM2-MR, CNRM-CM6-1, and CNRM-ESM2-1) as GO analysis. We used R package PresenceAbsence v. 1.1.11 [77] to calculate the threshold of ecological niche suitability, and grids with values higher than the threshold were defined as suitable habitats. Furthermore, referring to the method of Chen et al. [16], we defined niche suitability change (NSC) as the niche suitability index in the current climate minus the niche suitability index in the future climate. A positive value implies that niche suitability will decrease in the future compared to the present condition, while a negative value means increasing niche suitability. The NSC results of a single model for each emission scenario were averaged.

## Results

### Population structure and phylogeny of *M. oleifera*

Whole-genome resequencing generated an average of ∼4.71 Gb raw data and 65,007,259 paired-end reads for each sample, and the average sequencing depth was 6.5-fold. After filtering, the average Q20 and Q30 rates of paired-end reads were 97.51% and 92.68%, respectively, with an average mapping rate of 99.39% (Supplementary Tables S2 and S3). Neutral and adaptive genomic variations have inconsistent evolutionary patterns [78] and provide different types of information when determining optimal conservation measures [79]. To disentangle these discrepancies, we used 3 SNP datasets, including all loci (dataset 3), adaptive loci (dataset 4), and neutral loci (dataset 5), to decipher the genetic relationships within *M. oleifera* by constructing population structure, PCA, and phylogenetic trees.

The ADMIXTURE analysis results from all loci and neutral loci both revealed the optimal number of cluster (K) was 14 (Supplementary Fig. S7). Samples in most populations were relatively pure with no or only a mild genetic mixture with other populations, except for SG, ML, FS, and LY2 populations (SupplementaryFigs. S8a and S9a). However, ADMIXTURE analysis based on adaptive loci indicated that K = 10 was optimal (Supplementary Fig. S7), with ML-BB2, BB1-BM2, and ZL-LY1 paired populations having the same genetic composition, implying the paired population has similar adaptability, respectively (Fig. 1B). Notably, DX and GL populations were found to be 100% pure based on ADMIXTURE analysis of all datasets. Measures of PCA based on all datasets revealed clear separation of the DX population from other populations by PC1 and PC2, which explained 29.6%, 68.4%, and 25.2% of the genome covariance based on the results of all loci, neutral loci, and adaptive loci, respectively (Fig. 1D, Supplementary Figs. S8b and S9b). The NJ trees based on all datasets were consistent with the corresponding ADMIXTURE analysis, showing populations with similar genetic components had closer phylogenetic relationships (Fig. 1C, Supplementary Figs. S8c and S9c).

### Genetic diversity, heterozygosity, and genetic differentiation

The average whole genomic genetic diversity of *M. oleifera* was 3.87 × 10^−3^ ± 1.34 × 10^−3^ for pairwise nucleotide differences (θ_π_) and 3.46 × 10^−3^ ± 1.23 × 10^−3^ for Watterson"s θ (θ_w_) (Table 1, Supplementary Table S4 ). The BM1 population showed the highest θ_π_ and θ_w_ compared with other populations, while NP, JM, and BM2 populations had lower θ_π_ and θ_w_ (Supplementary Fig. S4) and showed a more obvious sawtooth-like distribution pattern of genetic diversity across the genome (Supplementary Fig. S5). When we divided the genome into 5 specific genomic regions, the values of θ_π_ and θ_w_ showed the trend of intergenic > fold-4 > intron > CDS > fold-0, which was highly consistent among all 16 populations (Table 1, Supplementary Table S4). The mean heterozygosity rate in *M. oleifera* was 0.50% ± 0.14%, and it varied among populations, with the BM1 (0.56% ± 0.16%) population showing the highest values and with the lowest heterozygosity rate seen in JM (0.30% ± 0.04%), NP (0.31% ± 0.04%), and BM2 (0.35% ± 0.02%) populations (Supplementary Table S5). As expected, the results of the heterozygosity rate across more specific genomic regions showed the same trend as the genetic diversity (Supplementary Fig. S6). Moreover, the genome-wide LD decay analysis revealed that the level of LD varied greatly between populations, with the BM2 population showing the slowest decay of LD, whereas the BM1 population had the fastest LD decay (Supplementary Fig. S3).

**Table 1:** Sample sizes and nucleotide diversity (θ_π_) in *M. oleifera* populations within the whole genome, CDS, fold-0, fold-4, intergenic, and intron regions.

Population	Sample size	Number of SNPs	θ_π__whole (× 10^−3^)	θ_π__CDS (× 10^−3^)	θ_π__fold-0 (× 10^−3^)	θ_π__fold-4 (× 10^−3^)	θ_π__intergenic (× 10^−3^)	θ_π__intron (× 10^−3^)
BB1	10	141,725	3.10 ± 0.42	1.19 ± 0.15	0.99 ± 0.13	1.93 ± 0.23	3.52 ± 0.49	1.83 ± 0.18
BB2	10	132,480	2.79 ± 0.41	1.08 ± 0.16	0.90 ± 0.13	1.75 ± 0.25	3.17 ± 0.47	1.68 ± 0.24
BL	10	146,763	3.56 ± 0.47	1.38 ± 0.17	1.15 ± 0.14	2.19 ± 0.27	4.07 ± 0.57	2.12 ± 0.25
BM1	17	218,460	6.13 ± 0.58	2.41 ± 0.18	2.00 ± 0.15	3.92 ± 0.26	7.56 ± 0.44	3.87 ± 0.20
BM2	5	101,436	2.15 ± 0.25	0.86 ± 0.13	0.73 ± 0.11	1.34 ± 0.17	2.45 ± 0.29	1.30 ± 0.13
DX	10	155,019	4.57 ± 0.57	1.73 ± 0.27	1.44 ± 0.22	2.77 ± 0.43	5.20 ± 0.62	2.71 ± 0.39
FS	9	189,667	5.13 ± 0.41	1.94 ± 0.22	1.61 ± 0.18	3.15 ± 0.31	5.86 ± 0.45	3.04 ± 0.26
GL	10	137,835	3.56 ± 0.45	1.35 ± 0.18	1.13 ± 0.15	2.13 ± 0.26	4.11 ± 0.52	2.05 ± 0.26
JM	10	98,031	2.07 ± 0.40	0.83 ± 0.20	0.69 ± 0.17	1.34 ± 0.30	2.36 ± 0.46	1.25 ± 0.22
LY1	10	157,624	3.89 ± 0.47	1.47 ± 0.22	1.22 ± 0.19	2.39 ± 0.33	4.47 ± 0.52	2.25 ± 0.31
LY2	14	200,098	5.50 ± 0.27	2.03 ± 0.19	1.68 ± 0.16	3.28 ± 0.26	6.28 ± 0.27	3.20 ± 0.21
ML	10	151,563	3.53 ± 0.40	1.34 ± 0.18	1.12 ± 0.15	2.13 ± 0.26	4.05 ± 0.47	2.08 ± 0.21
NP	10	106,945	2.03 ± 0.48	0.82 ± 0.25	0.69 ± 0.20	1.30± 0.38	2.31 ± 0.54	1.25 ± 0.30
SG	10	133,980	2.75 ± 0.34	1.10 ± 0.20	0.91 ± 0.17	1.80 ± 0.30	3.12 ± 0.40	1.65 ± 0.18
ZL	10	169,320	4.48 ± 0.41	1.67 ± 0.21	1.41 ± 0.17	2.61 ± 0.32	5.15 ± 0.43	2.59 ± 0.29
ZS	10	163,672	4.35 ± 0.32	1.63 ± 0.14	1.36 ± 0.12	2.60 ± 0.21	4.99 ± 0.36	2.55 ± 0.20

The values of pairwise F_st_ based on adaptive loci (dataset 4) were significantly higher than those of F_st_ based on all loci (dataset 3) and neutral loci (dataset 5), showing that adaptive loci > all loci > neutral loci in all paired populations (Supplementary Table S6). This was particularly prominent between the DX population and other populations, with a significant high pairwise F_st_ ranging from 0.87 to 0.91 based on adaptive loci, compared to 0.26–0.46 and 0.20–0.41 based on all loci and neutral loci, respectively (Supplementary Table S6). Moreover, BM2, JM, and NP populations, which have the lowest genetic diversity, showed high genetic differentiation from other populations (average pairwise F_st_ = 0.54 based on adaptive loci). In contrast, the BM1 population, with the highest genetic diversity, showed relatively low genetic differentiation from other populations (average pairwise F_st_ = 0.45 based on adaptive loci) (Supplementary Table S6).

### Demographic history of *M. oleifera*

**Figure 2:**
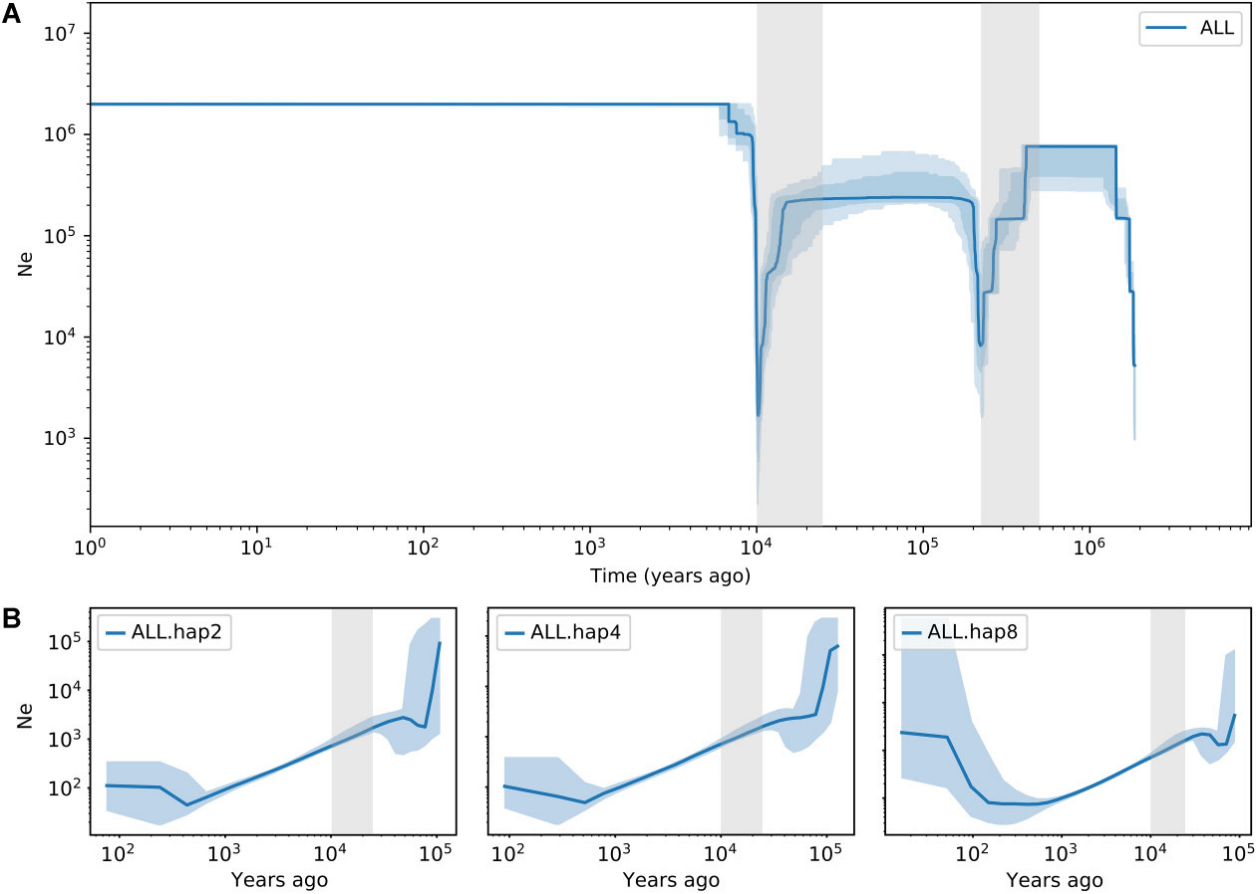
Demographic history of *M. oleifera* inferred by Stairway Plot v.2 based on unfolded SFS (A) and MSMC v.2 within 2, 4, and 8 haplotypes (B). The light blue lines correspond to the upper and lower bounds of the 95% confidence intervals. The severe effective population size (*Ne*) declines observed during the last glacial maximum (LGM) and the Middle Pleistocene are highlighted with gray vertical bars.

The stairway plot detected 2 severe population declines of *M. oleifera* based on unfolded SFS. The first occurred around 0.5–0.22 million years ago, corresponding to the Middle Pleistocene with climate upheaval, and the *Ne* was reduced to ∼8,230 (Fig. 2A). Subsequently, all the populations quickly recovered to ∼2.4 × 10^5^ and remained stable until a recent bottleneck at around 10 Ka during the last glacial maximum (LGM), where there was a sharp population contraction to its lowest level (∼1,676). The result based on folded SFS also showed 2 bottleneck events at the corresponding time (Supplementary Fig. S10). MSMC tracked the more recent demographic trajectory of *M. oleifera*, especially within the past 10,000 years (Fig. 2B). Based on the results of 2, 4, and 8 haplotypes, the *Ne* of *M. oleifera* experienced a significant decline over time, reaching a nadir (below 75) around 400 to 500 years ago, followed by a slight population expansion. It is worth mentioning that both programs detected a population decline in *M. oleifera* during the LGM, which strengthened the reliability of the results.

### Characterization of runs of homozygosity and deleterious mutations

**Figure 3:**
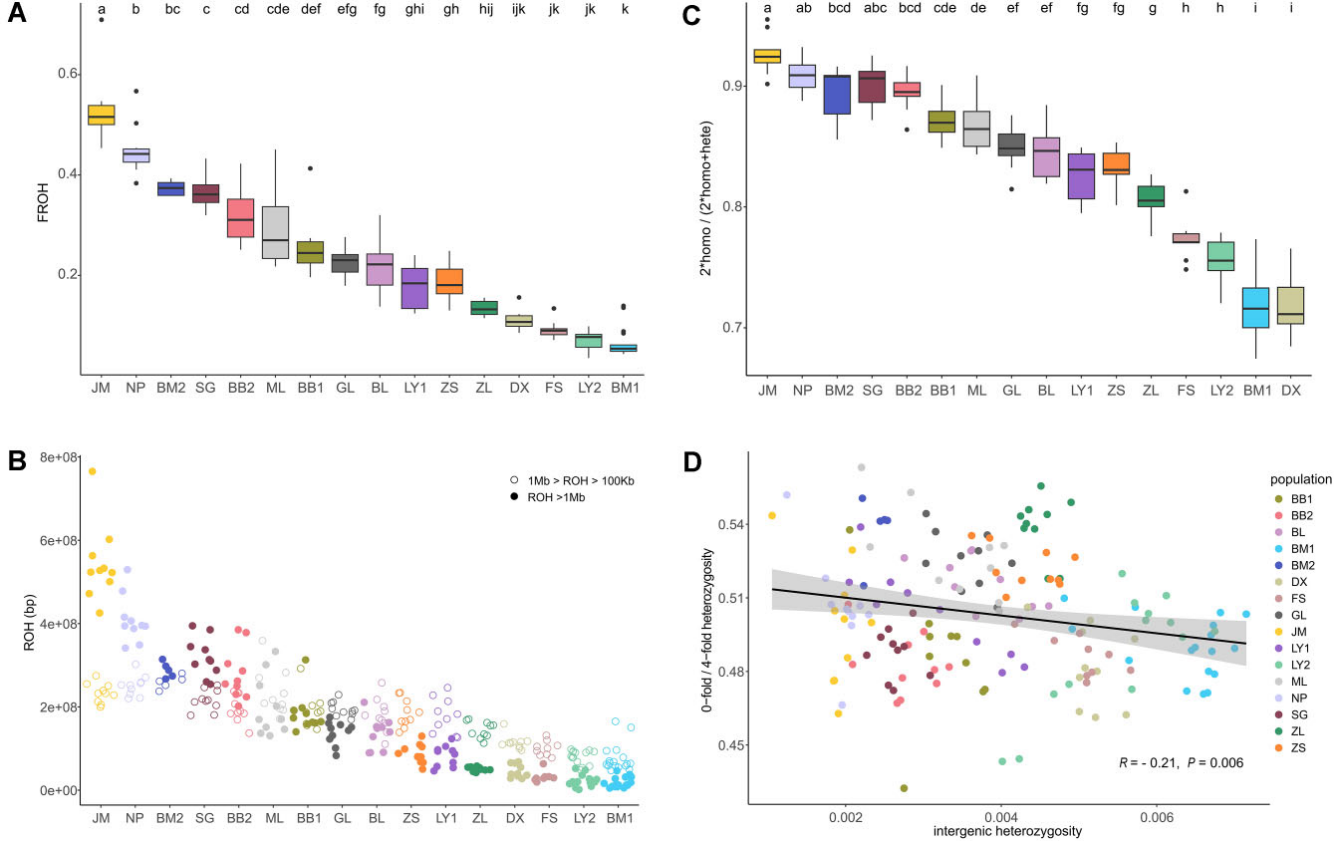
Levels of inbreeding and genetic load in different *M. oleifera* populations. (A) Fractions of the runs of homozygosity (FROH) show discrepancies in inbreeding levels in *M. oleifera* populations. (B) Distributions of long (>1 Mb) and medium (100 kb–1 Mb) runs of homozygosity (ROH) among 16 populations of *M. oleifera*. Solid dots represent ROH >1 Mb and hollow dots represent 1 Mb > ROH > 100 Kb. (C) Ratios of homozygous-derived deleterious mutations show the discrepancies in genetic load in different *M. oleifera* populations. Populations marked with the same letters in (A) and (C) are not significantly different. (D) Distributions of the ratio of 0- to 4-fold heterozygosity versus the intergenic heterozygosity across the 16 populations of *M. oleifera*. The dark line represents the significant negative correlation between these populations, with *R* = −0.21 and *P* = 0.006. Each dot represents an individual, which is colored by population.

We investigated whether *M. oleifera* showed signs of recent inbreeding by calculating the ROH. Referring to the method of Robinson et al. [10], we used the physical length of ROH to estimate the number of generations to the common ancestor (*g*) as *g* = 100/(2 * *L*), where *L* is the mean length of ROH in megabases (Mb). Here, the *L* of all 16 populations of *M. oleifera* ranged from 0.46 Mb (BM1) to 1.03 Mb (JM) (Supplementary Fig. S11). Our results indicated that inbreeding occurred about 49 to 112 generations ago. Specially, the effects of inbreeding varied greatly among populations (Fig. 3A). We found the FROH was significantly higher in the JM (45.37%–70.95%) population than in other populations, whereas it was lower in LY2 (3.52%–9.82%), BM1 (4.31%–13.92%), FS (7.11%–13.39%), and DX (8.51%–15.60%) populations. Moreover, populations with severe inbreeding would be predicted to have larger numbers of long ROH (>1 Mb) than short ROH (100 Kb–1 Mb) (Fig. 3B). Specifically, the JM population harbored maximum number of ROH >1 Mb, which represented 37.07% of the total genome. In contrast, the BM1 population had a minimum number of long ROH, with only 1.20% of ROH being longer than 1 Mb (Supplementary Table S7).

Based on the modified SIFT prediction approach, we detected a total of 2,404 deleterious mutations, 5,040 tolerated mutations, and 6,172 synonymous mutations (Supplementary Table S8 and Supplementary Fig. S13a). In particular, the frequency of deleterious mutations of homozygous-derived alleles reflects genetic load and adaptability of species, and it varies greatly among populations even within the same species [80]. Our results showed that the number of homozygous deleterious sites to the total deleterious mutations in the JM population was significantly higher than in most other populations except NP and SG, suggesting that the JM population had a higher genetic load (Fig. 3C). Interestingly, the ratio of 0-fold to 4-fold degenerate site heterozygosity showed a significant negative correlation with intergenic (neutral) heterozygosity (Fig. 3D). It implied that more severely deleterious mutations can be effectively purged by purifying selection, which may be the maintaining mechanism of *M. oleifera* with a small population size [45]. To provide accurate and direct genetic rescue guidance for the JM population, we selected 4 populations as potential pollen donors to construct a Venn diagram of homozygous deleterious variants (Supplementary Fig. S12a). Our results showed that the most homozygous deleterious variants were shared among all the 5 populations, and the JM population had the least shared homozygous deleterious variants with the BM1 population (172 variants).

### Signals of genomic offset to future climate change

Potential genomic variants related to climate adaptation were detected using BayeScEnv and RDA. For BayeScEnv analysis, with a *q*-value cutoff of 0.05, we identified 589 SNPs related to climate adaptation. Of these, 491 SNPs were associated with BIO7 and BIO14, respectively, followed by BIO3 (471 SNPs) and BIO15 (168 SNPs) (Supplementary Table S11). For RDA, 694 SNPs were detected along 5 significant RDA axes, of which 459 SNPs were correlated most to BIO14, 156 SNPs to BIO3, 40 SNPs to BIO7, and 39 SNPs to BIO15. To avoid false positives, we assigned the 380 SNPs detected by both BayeScEnv and RDA as environment-associated SNPs (Supplementary Table S11). To figure out the potential function of genomic variants associated with climate adaptation, we conducted a functional annotation of outlier SNPs. Gene Ontology enrichment analysis assigned a total of 258 Gene Ontology categories (*P* < 0.05), of which 158 categories belonged to biological processes and abundant genes were associated with metabolism, transmembrane transport, methylation, cell development, flowering, and telomere maintenance (Supplementary Table S12).

**Figure 4:**
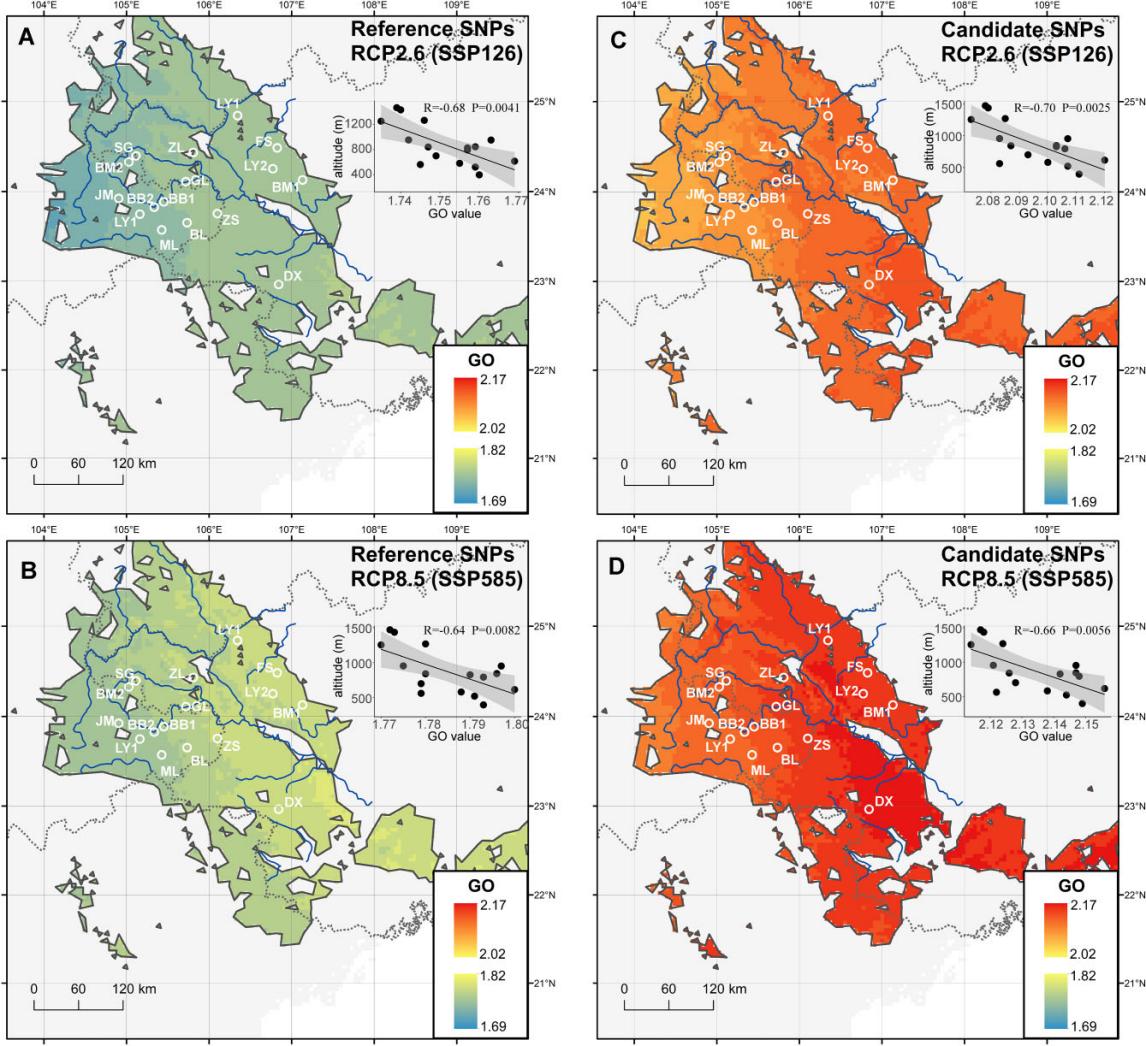
Predicted genetic offset of *M. oleifera* in the year 2100 under the SSP126 and SSP585 scenarios based on all SNPs (A, B) and adaptive SNPs (C, D), with higher values (red) representing more severe genomic vulnerability to future climate change. The inner mini plot represents the correlation between altitude and GO value in the corresponding scenario.

To assess which population of *M. oleifera* will be most likely disrupted in the future (2081–2100) under 2 greenhouse gas scenarios (SSP126 and SSP585), we employed the GF method to investigate the GO using integrated results of BCC-CSM2-MR, CNRM-CM6-1, and CNRM-ESM2-1 climate models. The GO is measured by the Euclidean distance of future climate condition compared to current climate status. Higher GO means greater allele frequency changes are required to adapt to the changing climate [81]. GF modeling showed that the degree of GO of all populations increased under scenario SSP585 compared to scenario SSP126, suggesting that extreme future climate change will cause severe genomic vulnerability to *M. oleifera* (Fig. 4). Compared with all SNPs (reference), we found that adaptive SNPs (candidate) exhibited higher GO under the same scenarios, implying that adaptive variants were more sensitive to climate change (Fig. 4). In addition, we found a strong negative correlation between GO and altitude, with populations at higher altitudes generally having lower GO values (Fig. 4).

### Ecological niche modeling predicted niche suitability change

**Figure 5:**
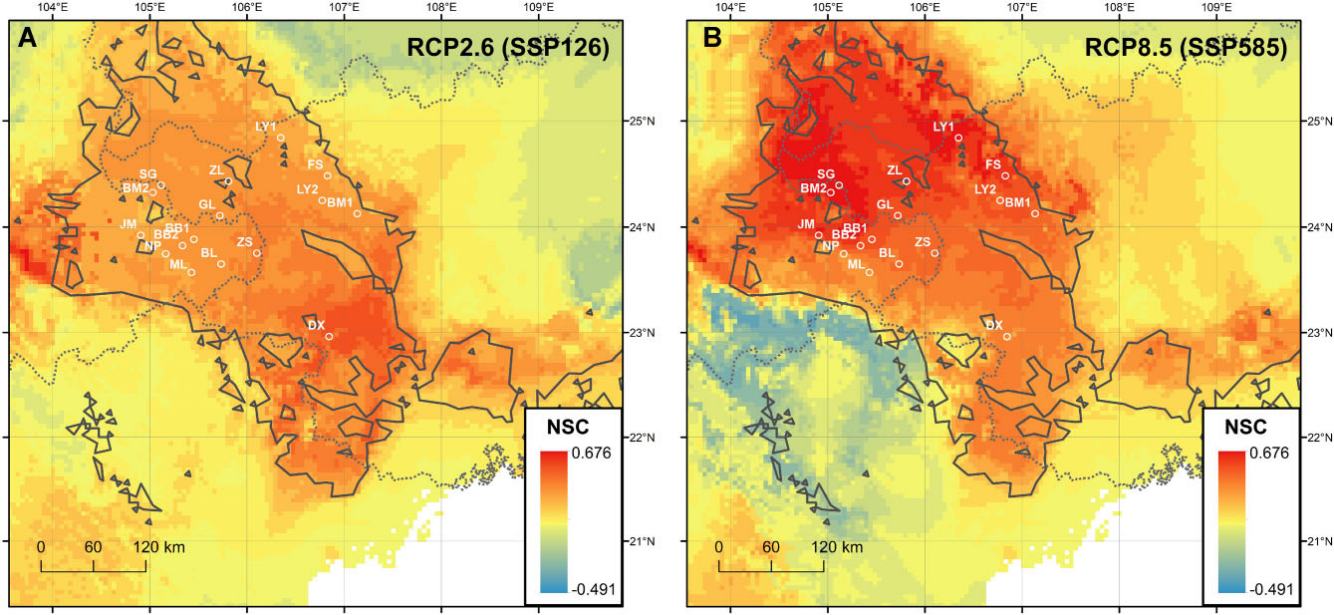
Predicted niche suitability change (NSC) of *M. oleifera* in the year 2100 under the SSP126 (A) and SSP585 (B) scenarios. Higher positive value suggests more severe degree of unsuitability.

We integrated results from 5 models to perform ecological niche modeling for *M. oleifera* (Supplementary Table S13). The area under the curve (AUC) value, Somer’s *D* value, TSS value, Boyce value, and the area under the precision–recall gain curve (AUCprg) were about 0.99, 0.99, 0.98, 0.74, and 0.97, respectively, indicating high performance of the ecological niche models (Supplementary Table S14). Compared to the current state, the potential suitable region in 2100 will reduce by 71.15% and 98.79% under scenarios SSP126 and SSP585, respectively, with the threshold of ecological niche suitability equal to 0.56 (Supplementary Fig. S15). Further, we calculated NSC between current and future climate for each grid using the following equation: NSC = niche suitability index in the current climate − niche suitability index in the future climate. A positive value indicates that niche suitability will be decreased under future climate change. Higher positive value means more severe degree of unsuitability. Our results showed slightly higher NSC in the southern part of the distribution range under scenario SSP126 (Fig. 5A). However, under scenario SSP585, the NSC increased to a much higher extent in the northern part of the distribution range (Fig. 5B), which is the Karst basin harboring the Nanpan, Beipan, and Tuoniang rivers. This suggest that drastic climate change will exacerbate ecological vulnerability in karst landforms by affecting hydrological processes [82, 83].

## Discussion

The genome harbors valuable evolutionary information of a species and provides deep insights into genetic diversity and evolutionary dynamics, but the full implementation of conservation genomics in practice is still limited [84]. In this study, we conducted a conservation genomics study on *M. oleifera* based on population-wide genome resequencing data, including 165 individuals. We aim to distinguish the potential factors that affect genetic diversity of *M. oleifera*, reveal the causes for the formation of its extremely small population patterns, and assess its adaptability under future climate change. It is our hope that based on the comprehensive results of conservation genomics, it will be possible to provide practical and meaningful suggestions for the conservation actions of this ecologically and economically important species.

### Recent inbreeding affected genetic diversity

*M. oleifera* has relatively high genetic diversity among endangered woody plants (Supplementary Table S15). This is confirmed by a population structure result, which shows K = 14 is optimal based on all loci (Supplementary Fig. S8a), suggesting *M. oleifera* has complex ancestral components despite occupying a narrow distribution. It seems optimistic, but genetic diversity cannot determine the endangered status of a species even though it is an important criterion for species conservation [85]. Genetic diversity is affected by many factors such as inbreeding, gene flow, life form, distribution, and rarity [86]. The key to perform conservation actions for endangered species is to realize the pivotal factor affecting genetic diversity. Our results showed that populations with low genetic diversity (JM, NP, and BM2) have a severe degree of recent inbreeding and displayed more pronounced sawtooth-like distribution patterns of nucleotide diversity across the genome due to long ROH (Fig.   3A, Supplementary Figs. S4 and S5). Furthermore, high levels of inbreeding in JM, NP, and BM2 populations have led to the accumulation of deleterious mutations (Fig. 3C), and they showed greater differentiation from other populations (Supplementary Table S6), which may result in a vicious circle of inbreeding depression without intervention [87].

### Causes for the formation of small and isolated population

Historical climate disturbances were one of the reasons for the formation of currently observed small and isolated *M. oleifera* populations. Based on the estimations of Stairway Plot v.2 and MSMC v.2, we observed a bottleneck event of *M. oleifera* during the LGM, resulting in a swift decline in *Ne* (Fig. 2). Although the stairway plot suggested the *Ne* recovered to its historical peak at the end of the LGM (Fig. 2A), this inference is deemed unreliable of very recent demographic events based on SFS [88]. In contrast, the MSMC results suggested that the *Ne* of *M. oleifera* underwent a protracted decline after the LGM, with a slight recovery occurring approximately 500 years ago (Fig. 2B). Moreover, we utilized the mean length of ROH as a metric to estimate the generations of inbreeding, referring to the method of Robinson et al. [10]. Our findings revealed that the JM population had experienced the most recent inbreeding, approximately 490 years ago (Supplementary Fig. S11). Intriguingly, the demographic history inferred by MSMC showed that the *Ne* of *M. oleifera* reached its nadir (below 75) about 400–500 years ago (Fig. 2B), which may have contributed to the extensive inbreeding. At the same time, human overexploitation and destruction of wild resources have exerted unbearable demographic pressures and resulted in further population fragmentation, as it is hard to find wild individuals again according to a large number of previous distribution records like Mashan, Pingguo, Tiandong, Tianyang, Youjiang, and Longzhou counties in Guangxi province [34]. Overall, the combined effect of historical bottleneck events, recent inbreeding, and excessive human disturbance may have led to the formation of small and isolated populations of *M. oleifera*.

### Local adaptation-related alleles lead to climate change-driven genomic vulnerability

The climate is currently shifting, and many species face the challenge of keeping pace with ongoing climate changes [89, 90]. Therefore, supporting species able to adapt to the variable climate is a key but tough task to future conservation action. For *M. oleifera*, we detected a total of 380 SNPs that are related to climate adaptation. The associated genes are also significantly enriched in key processes of metabolism, transmembrane transport, flower development, and telomere maintenance (Supplementary Table S12). Moreover, each climate factor is associated with dozens to hundreds of SNPs accordingly (Supplementary Table S11). This is consistent with the polygenic effects underlying local adaptability, meaning that organisms can adapt to rapid climate change through small polygenic allele frequency shifts [18, 91].

Inequivalent response to climate change exists within populations of the same species due to local adaptation to heterogeneous environments [92]. Analyzing local adaptation pattern can help us better understand how species respond to future climate change. Here, we used the Euclidean distance between future and current climate environments to measure GO. By incorporating intraspecific variations into the predictive GF model, our results showed *M. oleifera* populations distributed in the low elevation exhibit higher GO under both future scenarios (Fig. 4). Elevation also showed a strong negative correlation with annual mean temperature (*R* = –0.91, *P* < 2.2 × 10^−16^) across the distribution range of *M. oleifera* (Supplementary Fig. S16). This suggests populations in low altitude have a more significant adaptive lag in response to rapid climate change (especially temperature), indicating a greater risk of local extinction if appropriate conservation measures are not taken.

### Ecological niche modeling provides insights into *ex situ* conservation

Ecological niche modeling can predict potential current distribution ranges and suitable habitats under future climate change by linking observed species distribution and abundance to selected environmental variables [93]. Our results showed the suitable habitats for *M. oleifera* will be decreased and ecological niche suitability will be further reduced in the future (Supplementary Fig. S15). However, the degree of niche suitability change is varied under different climate scenarios. The extreme climate (SSP585) is likely to have direct impact on hydrological processes in karst landforms, making the northern part across the distribution range that harbors the Nanpan, Beipan, and Tuoniang rivers most unsuitable for living (Fig. 5B). BM2, SG, ZL, and LY1 populations located in the area deserve the highest priority for *ex situ* conservation when future climate becomes extremely severe. Ecological niche modeling reveals niche suitability change, while GO provides information about genomic inadaptation to future climate change [16]. The 2 methods provide disparate views to estimate climate-driven vulnerability. It is necessary to combine the methods of ecological niche modeling and genomic offset to make conservation decisions.

### Implications for conservation guidelines and management strategies

Currently, a wide range of field investigations and *in situ* protection of existing *M. oleifera* resources have been implemented [94, 95]. For example, in 2017, Guangnan County labeled 7,941 wild individuals and recorded their growth state [34]. But these measures only have a limited impact on guiding future conservation actions. The conservation guidelines and management strategies should be made under demarcating reasonable management units (MUs) and adaptive units (AUs) [96]. Based on the result of the population structure and phylogenetic tree (neutral loci), we suggest delineating 14 MUs of *M. oleifera*, with the most single population being separate MUs (Supplementary Fig. S9). Maintaining multiple MUs ensures long-term persistence of the species. Based on adaptive loci, we identified 10 AUs, including JM, SG-NP, ML-BB2, BB1-BM2, GL, ZS, ZL-LY1, LY2-FS, BM1, and DX AUs (Fig. 1B). Different AUs represent varied evolutionary potential. Understanding the patterns of adaptive differentiation is crucial when considering conservation priorities, assisted gene flow, migration, and supplementation [79].

For populations with recent inbreeding, genetic rescue is necessary through assisted gene flow [97]. The JM population has the lowest genetic diversity and the highest inbreeding and genetic load (Fig. 3, Supplementary Fig. S4), and thus it needs urgent genetic rescue. In some cases, a high F_st_ combination among parents generated offspring with a high heterozygosity, which may produce a heterozygous advantage, but in most cases, outbreeding depression has occurred [98]. It may occur between interpopulation crossing even in the same species [99]. So, to minimize the risk of outbreeding depression, we do not consider the DX population as a potential pollen donor because it has the highest F_st_ (0.90) with the JM population based on adaptive SNPs (Supplementary Table S6), suggesting severely adaptive differentiation has occurred. Under comprehensive evaluation, we propose the BM1 population as the prior pollen donor for the JM population, because (i) it displays moderate genetic differentiation (F_st_ = 0.51) with the JM population based on adaptive SNPs (Supplementary Table S6), (ii) it has the highest genetic diversity and heterozygosity (Supplementary Figs. S4 and S6), and (iii) it has the lowest inbreeding level and fewest shared homozygous deleterious mutations with the JM population (Supplementary Fig. S12a), positively reducing the impact of genetic load on hybrid offspring. Moreover, previous research has shown that *M. oleifera* seeds have low germination rates under natural conditions [24], so it is better to keep the seeds for artificial germination after implementing assisted gene flow measures and introduce robust seedlings back to the natural population later.

GO analysis predicts that populations located at lower altitudes require a greater change in adaptive allele frequencies to adapt to extreme climates (Fig. 4). Low-altitude areas are more susceptible to extreme temperatures than higher elevations (Supplementary Fig. S16). Therefore, we suggest cultivating heat-resistant individuals and screening a preadapted genotype under controlled conditions in the laboratory, and then regression experiments can be conducted. Future work should prioritize the conservation of *M. oleifera* because its lasting existence is a prerequisite to exploit resources.

## Supplementary Material

giae070_GIGA-D-24-00159_Original_Submission

giae070_GIGA-D-24-00159_Revision_1

giae070_Response_to_Reviewer_Comments_Original_Submission

giae070_Reviewer_1_Report_Original_Submission Yongzhi Yang, Ph.D. -- 6/24/2024 Reviewed

giae070_Reviewer_2_Report_Original_Submission Scott Ferguson -- 6/26/2024 Reviewed

giae070_Reviewer_3_Report_Original_Submission Wei Zhao -- 6/30/2024 Reviewed

giae070_Supplemental_Files

## Data Availability

Raw resequencing data are available at the NCBI Sequence Read Archive under BioProject PRJNA978997. All additional supporting data are available in the *GigaScience* repository, GigaDB [100].

## Additional Files

**Supplementary Note S1**. Ancestral sequence reconstruction.

**Supplementary Note S2**. Estimation of mutation rate.

**Supplementary Note S3**. Detection of deleterious mutations based on REF-ALT strategy.

**Supplementary Fig. S1**. Resequencing data processing workflow of *Malania oleifera*.

**Supplementary Fig. S2**. The distribution of SNPs (dataset 2) across 13 pseudochromosomes under 1-Mb windows.

**Supplementary Fig. S3**. Genome-wide linkage disequilibrium (LD) decay of *Malania oleifera*. (a) Considering 16 populations separately and (b) as a whole.

**Supplementary Fig. S4**. The comparison of mean θ_π_ and θ_w_ among 16 populations of *Malania oleifera* in whole-genome (a), intergenic (b), CDS (c), intron (d), fold-0 (e), and fold-4 (f) regions.

**Supplementary Fig. S5**. Distributions of nucleotide diversity (θ_π_) across the genome with (a–c) representing NP, JM, and BM2 populations (lowest average θ_π_), respectively, and (d) representing the BM1 population (highest average θ_π_).

**Supplementary Fig. S6**. The comparison of heterozygosity rate among 16 populations of *Malania oleifera* in whole-genome (a), intergenic (b), CDS (c), intron (d), fold-0 (e), and fold-4 (f) regions.

**Supplementary Fig. S7**. Cross-validation error curve based on all loci (a), neutral loci (b), and adaptive loci (c) for the 16 populations of *Malania oleifera* inferred by ADMIXTURE.

**Supplementary Fig. S8**. The inference of population structure (a), principal component analysis (b), and NJ tree (c) of *Malania oleifera* based on all loci.

**Supplementary Fig. S9**. The inference of population structure (a), principal component analysis (b), and NJ tree (c) of *Malania oleifera* based on neutral loci.

**Supplementary Fig. S10**. Demographic history of *Malania oleifera* inferred by Stairway Plot v.2 based on folded SFS.

**Supplementary Fig. S11**. Runs of homozygosity (ROH) frequency differences among 16 populations of *Malania oleifera*. The dashed lines correspond to the mean ROH lengths for each population.

**Supplementary Fig. S12**. The Venn diagrams of share and private homozygous deleterious mutations of JM (a), SG (b), and BM2 (c) populations with candidate pollen donors.

**Supplementary Fig. S13**. Differences in the number of deleterious mutations detected by REF-ALT strategy and ancestral status-based strategy of *Malania oleifera* (a) and *Acer yangbiense* (b).

**Supplementary Fig. S14**. The importance of environmental variables inferred by gradient forest modeling. *Top uncorrelated environment variables (Pearson’s |*r*| < 0.7) used in BayeScEnv, RDA, and GF analysis.

**Supplementary Fig. S15**. Integrated results of ecological niche modeling based on 5 models in the current (a) and future SSP126 (b) and SSP585 (c) scenarios. Higher values represent higher suitability.

**Supplementary Fig. S16**. Correlation of altitude with BIO1, BIO2, BIO4, BIO13, and BIO14 climate variables used in GO analysis.

**Supplementary Table S1**. Geographical location of all sampled individuals of *Malania oleifera*.

**Supplementary Table S2**. Statistical analysis of resequencing data before and after filtering with Fastp.

**Supplementary Table S3**. Statistical analysis of each individual mapping to the *Malania oleifera* reference genome.

**Supplementary Table S4**. Statistics on Watterson’s θ (θ_w_) of *Malania oleifera* populations within whole-genome, CDS, fold-0, fold-4, intergenic, and intron regions.

**Supplementary Table S5**. Statistics of heterozygosity and homozygosity rates of all individuals used in the whole-genome resequencing.

**Supplementary Table S6**. Weighted fixation statistics (F_st_) between populations of all loci, adaptive loci, and neutral loci and geographical distances between populations of *Malania oleifera*.

**Supplementary Table S7**. Estimation of runs of homozygosity (ROH) and frequency of runs of homozygosity (FROH).

**Supplementary Table S8**. Summary of deleterious, tolerated, and synonymous mutations of *Malania oleifere* based on REF-ALT strategy.

**Supplementary Table S9**. A list of 19 bioclimatic variables used in this study.

**Supplementary Table S10**. The projection coordinates of *M. oleifera* distribution records for ecological niche modeling.

**Supplementary Table S11**. Environment-associated SNPs and corresponding genes detected by BayeScEnv and redundancy analysis (RDA).

**Supplementary Table S12**. Gene ontology enrichment analysis of environment-associated genetic variants.

**Supplementary Table S13**. Predicted GO and NSC values of populations under future SSP126 and SSP585 scenarios using BCC-CSM2-MR, CNRM-CM6-1, and CNRM-ESM2-1 climate models.

**Supplementary Table S14**. The evaluation of ecological niche models.

**Supplementary Table S15**. Comparison of nucleic acid diversity of endangered species.

**Supplementary Table S16**. Information of 17 individuals for ancestral sequence reconstruction.

**Supplementary Table S17**. The genome list of 17 published species used in our research to estimate mutation rate for *Malania oleifera*.

## Abbreviations

a.s.l: above sea level; AUs: adaptive units; CDS: coding sequences; ENM: ecological niche model; FDR: false discovery rate; FROH: frequency of runs of homozygosity; F_st_: pairwise fixation statistics; GF: gradient forest; GO: genomic offset; IUCN: International Union for Conservation of Nature; LD: linkage disequilibrium; LGM: last glacial maximum; MAF: minor allele frequency; MUs: management units; Ne: effective population size; NJ: neighbor-joining; NSC: niche suitability change; PCA: principal component analysis; RDA: redundancy analysis; ROH: run of homozygosity; SFS: site frequency spectrum; SIFT: sorting intolerant from tolerant; SNP: single nucleotide polymorphism; SSP: Shared Socioeconomic Pathways; θ_w_: Watterson’s θ; θ_π_: nucleotide diversity.
