# Supplementary material for: Genomic insights into endangerment and conservation of the garlic-fruit tree (Malania oleifera), a plant species with extremely small populations
Source: Gigascience. 2024 Sep 23;13:giae070. doi: 10.1093/gigascience/giae070 (PMC11417964; doi:10.1093/gigascience/giae070)
Supplement: giae070_GIGA-D-24-00159_Original_Submission [file giae070_giga-d-24-00159_original_submission.pdf]

GigaScience

Genomic insights into endangerment and conservation of the garlic-fruit tree (Malania oleifera), a plant species with extremely small populations

--Manuscript Draft--

**Manuscript Number:**	GIGA-D-24-00159
**Full Title:**	Genomic insights into endangerment and conservation of the garlic-fruit tree (Malania oleifera), a plant species with extremely small populations
**Article Type:**	Research
**Funding Information:**	Key Project of Natural Science Foundation of Yunnan Province (202001AS070019) — Dr. Yongpeng Ma<br>the CAS “Light of West China” Program — Dr. Yongpeng Ma<br>Ten Thousand Talent Program of Yunnan Province (YNWRQNBJ-2018-174) — Dr. Yongpeng Ma
**Abstract:**	Background<br><br>Advanced whole genome sequencing techniques enable covering nearly all genome nucleotide variations, thus can provide deep insights into protecting endangered species. However, the use of genomic data to make conservation strategies is still rare, particularly for endangered plants. Here we performed comprehensive conservation genomic analysis for Malania oleifera, an endangered tree species with high amount of nervonic acid. We used whole-genome resequencing data of 165 samples, covering 16 populations across the entire distribution range to investigate the formation reasons of its extremely small population sizes and to evaluate the possible genomic offsets and changes of ecology niche suitability under future climate change.<br><br>Results<br><br>Although M. oleifera maintains relatively high genetic diversity among endangered woody plants ($\theta\pi$ = 3.87e-3), high levels of inbreeding have been observed, which has reduced genetic diversity in three populations (JM, NP and BM2) and caused the accumulation of deleterious mutations with weaker purifying selection effects. Repeated bottleneck events, recent inbreeding (~490 years ago) and anthropogenic disturbance to wild habitats have aggravated the fragmentation and endangered status of M. oleifera. Due to the significant effect of higher average annual temperature, populations distributed in low altitude exhibit greater genomic offset. Furthermore, ecological niche modelling shows the suitable habitats for M. oleifera will decrease by 71.15% and 98.79% in 2100 under scenarios SSP126 and SSP585, respectively.<br><br>Conclusions<br><br>The basic realizations concerning the threats to M. oleifera provide scientific foundation for defining management and adaptive units, and prioritizing populations for genetic rescue. Meanwhile, we highlight the importance of integrating genomic offset and ecological niche modeling to make targeted conservation actions under future climate change. Overall, our study provides a paradigm for genomics-directed conservation.
**Corresponding Author:**	Yongpeng Ma<br>Kunming Institute of Botany Chinese Academy of Sciences<br>Kunming, CHINA
**Corresponding Author Secondary Information:**	
**Corresponding Author's Institution:**	Kunming Institute of Botany Chinese Academy of Sciences
**Corresponding Author's Secondary Institution:**	
**First Author:**	Yuanting Shen

Powered by Editorial Manager® and ProduXion Manager® from Aries Systems Corporation

First Author Secondary Information:	
Order of Authors:	Yuanting Shen
	Lidan Tao
	Gang Yao
	Rengang Zhang
	Weibang Sun
	Yongpeng Ma
Order of Authors Secondary Information:	
Additional Information:	
**Question**	**Response**
Are you submitting this manuscript to a special series or article collection?	No
**Experimental design and statistics**<br><br>Full details of the experimental design and statistical methods used should be given in the Methods section, as detailed in our Minimum Standards Reporting Checklist. Information essential to interpreting the data presented should be made available in the figure legends.<br><br>Have you included all the information requested in your manuscript?	Yes
**Resources**<br><br>A description of all resources used, including antibodies, cell lines, animals and software tools, with enough information to allow them to be uniquely identified, should be included in the Methods section. Authors are strongly encouraged to cite Research Resource Identifiers (RRIDs) for antibodies, model organisms and tools, where possible.<br><br>Have you included the information requested as detailed in our Minimum Standards Reporting Checklist?	Yes
**Availability of data and materials**	Yes

Powered by Editorial Manager® and ProduXion Manager® from Aries Systems Corporation

All datasets and code on which the conclusions of the paper rely must be either included in your submission or deposited in publicly available repositories (where available and ethically appropriate), referencing such data using a unique identifier in the references and in the "Availability of Data and Materials" section of your manuscript.<br><br>Have you have met the above requirement as detailed in our Minimum Standards Reporting Checklist?	

Powered by Editorial Manager® and ProduXion Manager® from Aries Systems Corporation

Manuscript
Click here to access/download;Manuscript;manuscript-Gigascience.docx

Genomic insights into endangerment and conservation of the garlic-fruit tree (*Malania oleifera*), a plant species with extremely small populations

Yuanting Shen[1,2,3,4]†, Lidan Tao[1,2,3]†, Gang Yao[1,2], Rengang Zhang[1,2,3*], Weibang Sun[1,2*], Yongpeng Ma[1,2*]

[1]Yunnan Key Laboratory for Integrative Conservation of Plant Species with Extremely Small Populations, Kunming Institute of Botany, Chinese Academy of Sciences, Kunming 650201, China
[2]Key Laboratory for Plant Diversity and Biogeography of East Asia, Kunming Institute of Botany, Chinese Academy of Sciences, Kunming 650201, China
[3]University of Chinese Academy of Sciences, Beijing 100049, China
[4]State Key Laboratory of Plant Diversity and Specialty Crops, Institute of Botany, Chinese Academy of Sciences, Beijing 100093, China.

* Corresponding authors. E-mail address: mayongpeng@mail.kib.ac.cn; wbsun@mail.kib.ac.cn; zhangrengang@mail.kib.ac.cn
†These authors contribute equally to this work.

Abstract

Background: Advanced whole genome sequencing techniques enable covering nearly all genome nucleotide variations, thus can provide deep insights into protecting endangered species. However, the use of genomic data to make conservation strategies is still rare, particularly for endangered plants. Here we performed comprehensive conservation genomic analysis for *Malania oleifera*, an endangered tree species with high amount of nervonic acid. We used whole-genome resequencing data of 165 samples, covering 16 populations across the entire distribution range to investigate the formation reasons of its extremely small population sizes and to evaluate the possible genomic offsets and changes of ecology niche suitability under future climate change.

Results: Although *M. oleifera* maintains relatively high genetic diversity among endangered woody plants ($\theta_\pi = 3.87e\text{-}3$), high levels of inbreeding have been observed,

which has reduced genetic diversity in three populations (JM, NP and BM2) and caused the accumulation of deleterious mutations with weaker purifying selection effects. Repeated bottleneck events, recent inbreeding (~490 years ago) and anthropogenic disturbance to wild habitats have aggravated the fragmentation and endangered status of *M. oleifera*. Due to the significant effect of higher average annual temperature, populations distributed in low altitude exhibit greater genomic offset. Furthermore, ecological niche modelling shows the suitable habitats for *M. oleifera* will decrease by 71.15 % and 98.79 % in 2100 under scenarios SSP126 and SSP585, respectively.

Conclusions: The basic realizations concerning the threats to *M. oleifera* provide scientific foundation for defining management and adaptive units, and prioritizing populations for genetic rescue. Meanwhile, we highlight the importance of integrating genomic offset and ecological niche modeling to make targeted conservation actions under future climate change. Overall, our study provides a paradigm for genomics-directed conservation.

Keywords

Recent inbreeding; Deleterious mutation; Demographic history; Genomic offset; Ecological niche modelling; Conservation genomics

Introduction

Historical climate disturbances and frequent human activity have caused many species that were once widespread with continuous distributions to become small, fragmented populations [1]. High levels of inbreeding continually occur in these populations, leading to the accumulation of deleterious mutations and low species adaptability, ultimately increasing the risk of extinction [2, 3]. Genome contains evolutionary footprints which can be used to estimate inbreeding levels of species even without detailed pedigrees [4, 5]. For example, runs of homozygosity (ROH), genome regions with a certain length that is identical by descent, has been widely used as an indicator of inbreeding [6, 7]. The long ROH indicate a closer relationship to the most recent common ancestor, implying a higher level of inbreeding. For small and isolated populations with high inbreeding levels, genetic rescue is necessary to introduce beneficial mutations by establishing gene flow between populations [8]. However, cautions should be taken when making decisions regarding genetic rescue, and comprehensive exploration of genetic background of these small populations must be done in advance [9, 10].

Rapid climate change in the future is a widely recognized threat to global biodiversity [11-13]. The threatened degree of species depends on how they respond to climate change. There are two main mechanisms: migrating to new habitats or adapting to the changing environments through phenotypic plasticity or *de novo* mutations [14-16]. However, in the case of long-lived forest trees, individual organisms are nearly

incapable of migrating to keep pace with changing climate and may experience maladaptation throughout their lifetimes [17-19]. Thus, if species' responses to future climate change can be predicted, their extinction risks can be estimated and targeted conservation guidelines and management strategies can be developed in advance.

Malania oleifera Chun & S. K. Lee, the single species in the genus *Malania* (Olacaceae), is an endemic, semi-parasitic evergreen tree that naturally scattered in the west Guangxi (a.s.l 300~1000 m) and southeast Yunnan province, China (a.s.l 300~1640 m) [20]. It adapts well to rocky desert habitats and can be used as an afforestation tree in karst landscapes [21]. Moreover, it has extremely high economic and medicinal value due to the large amount of lipids in its seed. The main lipid component is nervonic acid (cis-tetracos-15-enoic acid, >60%), which is essential for human nervous health [22]. However, mainly due to overexploitation, wild resources of *M. oleifera* have decreased by approximately 25,000 individuals between 2000 and 2017 solely in Guangnan County (Yunnan, China) [23, 24]. Additionally, physiological factors of *M. oleifera*, including large seed size, short seed lifespan, difficulty in natural seed germination, low rate of pollen germination and susceptibility to root rot, have made natural propagation and regeneration difficult [25-28]. Therefore, *M. oleifera* has been categorized as Vulnerable (VU) on the IUCN Red List (Sun, 1998) and recorded in the Class II Key Protected Wild Plant List in China [29], and it has also been listed as a plant species with extremely small population size in China [30], highlighting the urgent need for its conservation.

Previous studies related to *M. oleifera* mainly focused on the biosynthetic pathway of nervonic acid [22, 31] and exploring the optimal condition of growing artificial seedlings for better utilization [32, 33]. However, the conservation process of *M. oleifera* is limited to in situ protection of existing wild resources [34]. Recent advancements in whole genome sequencing techniques enable covering nearly all the nucleotide variations of a genome and can provide deep insights into protecting endangered species [35]. However, there is a gap in using genomic data to guide conservation strategies, particularly for plants. Therefore, we utilize *M. oleifera* as a study case to investigate the above issues. We aimed to provide a comprehensive framework for the *M. oleifera* conservation through the perspective of conservation genomics.

Materials and methods

Sample collection and whole genome resequencing

A total of 165 leaf samples were collected from 16 populations across the entire distribution of *M. oleifera* from Yunnan and Guangxi provinces, China (Figure 1a; Table S1). Among them, 76 samples were collected from wild individuals, and the remaining 89 samples were obtained from the Germplasm Bank of Wild Species in Southwest China. The number of individuals collected per population varied from 5 to 17, and if populations contained fewer than ten individuals, all individuals were

sampled.

Genomic DNA was extracted from silica-dried leaf tissues using a modified CTAB method [36] and the concentration and quality of the DNA was determined using a NanoDrop2000 Spectrophotometer (Thermo Fisher Scientific). Samples were sent to Beijing Ori-Gene Science and Technology Co., Ltd for Illumina sequencing library preparation according to the manufacturer's specifications. Paired-end raw reads (150bp) were generated on the Illumina HiSeq platform.

Read mapping and SNP calling

The raw data were filtered using Fastp v. 0.19.3 [37]. Paired-end clean reads were mapped to the *M. oleifera* reference genome [31] using BWA-MEM v. 2.1 [38]. SAMtools v. 1.9 [39] was used to convert sequence alignment map (SAM) format files to sorted binary alignment map (BAM) format files. Sambamba v.0.7.1 [40] was used to mark and remove duplicate reads. Freebayes v. 1.3.6 [41] was employed to call variants, and only bases with a quality score $\geq$20 and reads with a mapping quality score $\geq$30 were included. This produced total of 43,413,408 initial variant sites (dataset 1). We then employed VCFtools v. 0.1.15 [42] to filter sites with the following criteria: (1) sites with coverage depth below 1/2*average site coverage and above 2*average site coverage were discarded after investigating the coverage distribution; (2) sites located on the organelle genomes, or contigs that were not anchored on the chromosomes were excluded; (3) SNPs with depth below 3× or genotype quality score < 20 were redefined as missing; (4) only bi-allelic SNPs were reserved; (5) SNPs with a missing rate >20 % were removed, leaving 2,144,506 SNPs (dataset 2); and (6) SNPs with a minor allele frequency < 0.05 were all excluded, finally remained 250,362 SNPs (dataset 3) for the subsequent analyses.

Population genetic diversity and runs of homozygosity

Based on dataset 3, we detected genome-wide linkage disequilibrium (LD) decay among 16 populations using PopLDdecay v. 3.4.0 [43]. Nucleotide diversity (θ_π), Watterson's θ (θ_W) and heterozygosity rate were calculated using ANGSD v. 0.921 [44] based on the bam files. In addition, we calculated the values of the three parameters in more specific genomic regions (intergenic, CDS, intron, fold-0 and fold-4). To examine inbreeding depression, we detected runs of homozygosity (ROH) using vcftools v. 0.1.15 [42] based on dataset 3 with the key parameters "--LROH" and only ROH longer than 100 kb were kept. Moreover, we calculated the frequency of runs of homozygosity (FROH), which is equal to the sum of all ROH lengths longer than 100 kb divided by genome effective length [45].

Inference of population structure and genetic differentiation based on all loci, adaptive loci and neutral loci

We employed two distinct methods to detect outlier SNPs potentially related to adaptive evolution. Firstly, we used the sparse non-negative matrix factorization (snmf) function

applied in the R package LEA v. 3.1.4 [46] to estimate the most likely number of ancestral populations based on dataset 3. To reduce the number of false positives, we reserved SNPs with the false discovery rate (FDR) less than 0.01 and denoted as dataset 4. Secondly, we applied a principal component analysis (PCA) method using R package Pcadapt v. 4.3.3 [47] to identify adaptive SNPs highly influential in forming the observed differentiation based on dataset 3. A value of 0.01 was also set for the FDR, and the retained SNPs were recorded as dataset 5. SNPs presented in both datasets 4 and 5 were considered as the adaptive SNPs, otherwise, they were considered as neutral SNPs. Finally, we employed PLINK v. 1.90b4.1 [48] to filter out linkage disequilibrium sites from dataset 3, the adaptive dataset and the neutral dataset. We ultimately obtained 33,971 (dataset 6, all loci), 1,515 (dataset 7, adaptive loci) and 32,930 (dataset 8, neutral loci) SNPs for downstream analysis (Figure S1).

Based on the three datasets described above, we employed ADMIXTURE v. 1.3.0 [49] to infer the population structure. The most likely population number of K was determined by the minimizing cross-validation error. Principal component analysis (PCA) was conducted in GCTA v1.94.1 [50] and MEGA v. 7.0 [51] was used to construct NJ trees. Pairwise fixation statistics (Fst) among the 16 populations were calculated using vcftools v. 0.1.15 [42].

Estimation of demographic history

We used Stairway Plot v.2 [52] and MSMC v.2 [53] to infer the population demographic history of *M. oleifera*. Stairway Plot uses site frequency spectrum (SFS) (see Note S1 and Table S16) as input, and we set the average generation time of *M. oleifera* as ten years because it takes about ten years for a seed to grow into a seed-producing plant according to our field observations. The mutation rate was set to 2.5e-8 per site per generation (see Note S2 and Table S17). MSMC is a Multiple Sequentially Markovian Coalescent approach which uses the density of heterozygous sites to estimate the effective population size (*Ne*) through time. Different individual numbers or haplotypes provide distinct resolutions for the analysis of demographic histories. Therefore, we employed MSMC to separately estimate the coalescence rate within two, four and eight haplotypes, referring to the simulation results of Schiffels & Durbin [53]. A total of 100 random combinations of individuals were used for the three haplotype analyses to estimate the medians and 95% CI values. The average generation time and mutation rate values were set to the same as in Stairway Plot.

Detection of deleterious mutations

Deleterious mutations in *M. oleifera* were predicted using the Sorting Intolerant From Tolerant (SIFT) algorithm [54]. We used a modified approach to perform the SIFT prediction (see Note S3). The TrEMBL plant database [55] was used to search for orthologous genes and the SIFT scores were calculated based on the degree of conservation among loci. Based on dataset 2 (included low frequency variants), SNPs in the coding regions were categorized as deleterious (SIFT score <0.05), tolerated

(SIFT score ≥0.05) or synonymous using SIFT4G [56]. The low confidence sites and "NA" sites were not considered.

To provide accurate and direct genetic rescue guidance for *M. oleifera* populations with high genetic load, we selected five populations, including one as the potentially threatened population and four as the candidate pollen-donors. We drew a Venn diagram to explore the distribution of shared or unique homozygous deleterious mutations among the five populations. The four-candidate pollen-donors were characterized by (1) having low genetic load, low levels of inbreeding, high genetic diversity, and high heterozygosity; or (2) low genetic differentiation with the rescued population; or (3) sharing the same genetic lineage as the rescued population based on adaptive loci.

Identification of environment-associated adaptive variants

The environmental data included 19 bioclimatic variables at 2.5-minute resolution (5 km) were downloaded from the WorldClim v.2.1 database (https://www.worldclim.org/) (Table S9). Each environmental factor is extracted through the coordinates of the sampling points. To avoid multicollinearity, we kept variables with correlation coefficients less than |0.7| by calculating Pearson's correlation coefficient in the R package corrplot v.0.92 [57] and ultimately retained five uncorrelated factors, including BIO1 (Annual Mean Temperature), BIO2 (Mean Diurnal Range), BIO4 (Temperature Seasonality), BIO13 (Precipitation of the Wettest Month) and BIO14 (Precipitation of the Driest Month). Next，we used BayeScEnv [58] and Redundancy Analysis (RDA) [59] to identify environment-associated SNPs. BayeScEnv represents a univariate genotype-environment association approach. For BayeScEnv method, the input files included environmental factor standardized by the mean variance and contained codominant data which converted by PGDSpider v. 2.1.1.5 [60] based on dataset 3. RDA is a multivariate linear regression-based method [61]. We ran RDA analysis in the R package vegan v.2.5-7 [62], using function "anova.cca" to check the significance of RDA model and function "outliers" to identify local-adaptation-associated SNPs that load in the tails of ±3 standard deviation cut-off (two tailed P-value = 0.0027). To recognize the gene functions of the candidate SNPs obtained from BayeScEnv and RDA, we employed a Gene Ontology enrichment analysis using the eggNOG-mapper v.2 [63].

Genomic offset modeling with gradientforest

We used the machine-learning gradient forest (GF) model in the R package gradientforest v.0.1-37 [64] to predict genomic vulnerability to future climate change. The environmental-associated SNPs detected by both BayesScEnv and RDA were denoted as candidate SNPs dataset. And 500 randomly selected SNPs based on dataset 3 were recorded as reference SNPs dataset to match the magnitude of candidate SNPs dataset. The SNPs data with MAF > 10 % were converted into minor allele frequencies (MAF) per population. To ameliorate the linkage effect, we only kept one SNP per 1,000,000 bp range and finally obtained 326 reference SNPs and 279 candidate SNPs.

We employed the GF model with 500 regression trees per SNP to build a function for five of the most important environmental factors (BIO2, BIO4, BIO1, BIO14, BIO13). Genomic offset (GO) was defined by the Euclidean distance between current (1970-2000) and future (2081-2100) climate which used the current condition as baseline [16]. To explore possible future climate conditions and predict GO for *M. oleifera*, we employed three widely used global climate models (BCC-CSM2-MR, CNRM-CM6-1 and CNRM-ESM2-1) and two emission scenarios (SSP126 and SSP585) which represent the mild and extreme future carbon emissions. The predicted GO results of three global climate models for each grid were averaged with assigned weights.

Ecological niche modelling

The distribution records of *M. oleifera* were collected from online databases (https://www.cvh.ac.cn/, https://www.gbif.org/), published academic articles [65, 66] and field investigation, and all records were manually verified using online map. To reduce sampling bias, we only kept one record within 5km using the rarefy function of the R package Humboldt [67], remaining a total of 87 records (Table S10). The 19 bioclimatic variables (Table S9) were also used in ecological niche modelling. Since background or pseudo-absence data of ecological niche models were sampled from the entire modeling map, we recalculated the correlation coefficients of 19 bioclimatic variables based on the whole distribution area. We removed autocorrelated variables (Pearson's $r > 0.7$ and variance inflation factor > 10) using the R package usdm v.2.1 [68] and kept six uncorrelated variables (BIO1, BIO2, BIO7, BIO12, BIO14 and BIO18) for ecological niche modelling.

The ecological niche model (ENM) was built using an ensemble modeling method that combined outputs of five single models with high performance: GAM (Generalized Additive Model by the R package mgcv v.1.9) [69], MaxEnt (tuned MaxEnt model by the R package dismo v.1.3) [70], RF (Random Forest with down-sampling by the R package randomForest v.4.7) [71], Lasso (by the R package glmnet v.4.1) [72], and BRT (Boosted Regression Trees by the R package dismo) [73]. Each model was run for ten replicates, with pseudo-absence data of 10,000 points randomly generated using the R package Biomod2 [74] for three replicates, resulting in a total of 5*10*3=150 single models. However, only models with positive Somer's D values were employed to create the final ensemble prediction, which was weighted by the TSS value of each model. The evaluation of single model and ensembled model was performed by the R packages Ecospat v.4.0.0 [75] and prg v.0.5.1 [76].

The niche suitability of *M. oleifera* under future (2081-2100) carbon emission scenarios (SSP126 and SSP585) was predicted using the same climate models (BCC-CSM2-MR, CNRM-CM6-1 and CNRM-ESM2-1) as GO analysis. We used R package PresenceAbsence v. 1.1.11 [77] to calculate the threshold of ecological niche suitability and grids with values higher than the threshold were defined as suitable habitats. Furthermore, referring to the method of Chen et al. [16], we defined NSC as niche

suitability change between current and future climate, which is equal to the niche suitability index in the current climate minus the niche suitability index in the future climate. A positive value implies that niche suitability will decrease in the future compared to the present condition, while a negative value means increasing niche suitability. The NSC results of single model for each emission scenario were averaged.

Results

Linkage disequilibrium, nucleotide diversity and heterozygosity

Whole genome resequencing generated an average of ~4.71 Gb raw data and 65,007,259 paired-end reads for each sample, and the average sequencing depth was 6.5-fold. After filtering, the average Q20 and Q30 rates of paired-end reads were 97.51 % and 92.68 %, respectively, with an average mapping rate of 99.39 % (Table S2 and S3).

The genome-wide LD decay analysis revealed that the level of LD varied greatly between populations, with BM2 population showing the slowest decay of LD, whereas BM1 population had the fastest LD decay (Figure S2). The average whole genomic genetic diversity of *M. oleifera* was 3.87 ± 1.34e-3 for pairwise nucleotide differences (θ_{π}) and 3.46 ± 1.23e-3 for Watterson's θ (θ_W) (Table 1 and S4). BM1 population showed the highest θ_{π} and θ_W compared with other populations, while NP, JM and BM2 populations had lower θ_{π} and θ_W (Figure S3) and showed a more obvious sawtooth-like distribution pattern of genetic diversity across the genome (Figure S4). When we divided the genome into five specific genomic regions, the values of θ_{π} and θ_W showed the trend of intergenic > fold-4 > intron > CDS > fold-0, which was highly consistent among all 16 populations (Table 1 and S4). The mean heterozygosity rate in *M. oleifera* was 5.09 ± 1.43e-3, and it varied among populations, with BM1 (5.62 ± 1.56e-3) population showing the highest values, and with the lowest heterozygosity rate seen in JM (2.99 ± 0.42e-3), NP (3.14 ± 0.37e-3) and BM2 (3.54 ± 0.16e-3) populations (Table S5). As expected, the results of heterozygosity rate across more specific genomic regions showed the same trend as the genetic diversity (Figure S5).

Population structure and genetic differentiation

Neutral and adaptive genomic variations have inconsistent evolutionary patterns [78] and provide different types of information when determining optimal conservation measures [79]. To disentangle these discrepancies, we used three SNP datasets, including all loci (dataset 6), adaptive loci (dataset 7) and neutral loci (dataset 8), to decipher the genetic relationships within *M. oleifera* by constructing population structure, PCA and phylogenetic trees.

The ADMIXTURE analysis results from all loci and neutral loci both revealed the optimal number of clusters (K) was 14 (Figure S6). Samples in most populations were relatively pure with no or only mild genetic mixture with other populations, except for SG, ML, FS and LY2 populations (Figure S7a and S8a). However, ADMIXTURE analysis based on adaptive loci indicated that K = 10 was optimal (Figure S6) with ML-

BB2, BB1-BM2 and ZL-LY1 paired populations have the same genetic composition, implying the paired population have similar adaptability, respectively (Figure 1b and S6). Notably, DX and GL populations were found to be 100 % pure based on ADMIXTURE analysis of all datasets. Measures of PCA based on all datasets revealed clear separation of DX population from other populations by PC1 and PC2, which explained 29.6 %, 68.4 % and 25.2 % of the genome covariance based on the results of all loci, neutral loci, and adaptive loci, respectively (Figure 1d, S7b and S8b). The NJ trees based on all datasets were consistent with the corresponding ADMIXTURE analysis, showing populations with similar genetic components had closer phylogenetic relationships (Figure 1c, S7c and S8c).

The results of pairwise Fst based on all loci indicated DX population have significant higher Fst with other populations (average pairwise Fst = 0.38). Moreover, BM2, JM and NP populations, which have the lowest genetic diversity, showed high genetic differentiation from other populations (average pairwise Fst = 0.31). In contrast, BM1 population with highest genetic diversity, showed relatively low genetic differentiation from other populations (average pairwise Fst = 0.21) (Table S6).

Demographic history of *M. oleifera*

Stairway plot detected two severe population declines in *M. oleifera.* The first occurred around 0.5-0.22 Ma, corresponding to the Middle Pleistocene with climate upheaval and the *Ne* was reduced to ~8230 (Figure 2a). Subsequently, all the populations quickly recovered to ~2.4×10^5 and remained stable until a recent bottleneck at around 10 Ka during the last glacial maximum (LGM), where there was a sharp population contraction to its lowest level (~1676). MSMC tracked the more recent demographic trajectory of *M. oleifera*, especially within the last 10,000 years (Figure 2b). Based on the analyses results of two, four and eight haplotypes, the *Ne* of *M. oleifera* experienced a significant decline over time, reaching a nadir (below 75) around 400-500 years ago, followed by a slight population expansion. It is worth mentioning that both programs detected a population decline in *M. oleifera* during the LGM, which strengthened the reliability of the results.

Characterization of runs of homozygosity and deleterious mutations

We investigated whether *M. oleifera* showed signs of recent inbreeding by calculating the runs of homozygosity (ROH). Referring to the method of Robinson et al [10], we used the physical length of ROH to estimate the number of generations to the common ancestor (g) as $g = 100/(2*L)$, where L is the mean length of ROH in megabases (Mb). Here, the L of all 16 populations of *M. oleifera* ranged from 0.46 Mb (BM1) to 1.03 Mb (JM) (Figure S9). Our results indicated that inbreeding occurred about 49 to 112 generations ago (Figure S9). Specially, the effects of inbreeding varied greatly among populations (Figure 3a). We found the frequency of runs of homozygosity (FROH) was significantly higher in JM (45.37 %-70.95 %) population than in other populations, whereas it was lower in LY2 (3.52 %-9.82 %), BM1 (4.31 %-13.92 %), FS (7.11 %-

13.39 %) and DX (8.51 %-15.60 %) populations. Moreover, populations with severe inbreeding would be predicted to have larger numbers of long ROH (> 1 Mb) than short ROH (100 Kb - 1 Mb) (Figure 3b). Specifically, JM population harbored maximum number of ROH > 1 Mb, which represented 37.07 % of the total genome. In contrast, BM1 population had minimum number of long ROH, with only 1.20 % of ROH being longer than 1 Mb (Table S7).

Based on the modified SIFT prediction approach, we detected a total of 2,404 deleterious mutations, 5,040 tolerated mutations and 6,172 synonymous mutations (Table S8 and Figure S11a). Particularly, the frequency of deleterious mutations of homozygous-derived alleles reflects genetic load and adaptability of species, and it varies greatly among populations even within the same species [80]. Our results showed that the ratio of the number of homozygous deleterious sites to the total deleterious mutations in JM population was significantly higher than in most other populations except NP and SG, suggesting that JM population had higher genetic load (Figure 3c). Interestingly, we found that populations with severe inbreeding also had stronger genetic load, but experienced weaker purifying selection against nonsynonymous mutations (Figure 3d). Therefore, the accumulation of genetic load may be related to severe inbreeding but reduced efficiency of purifying selection. To provide accurate and direct genetic rescue guidance for JM population, we selected four populations as potential pollen-donors to construct a Venn diagram of homozygous deleterious variants (Figure S10a). Our results showed that the most homozygous deleterious variants were shared among all the five populations and JM population had least shared homozygous deleterious variants with BM1 population (172 variants).

Signals of genomic offset to future climate change

Potential genomic variants related to climate adaptation were detected using BayeScEnv and Redundancy Analysis (RDA). For BayeScEnv analysis, with a q-value cut-off of 0.05, we identified 578 SNPs related to climate adaptation. Of these, 491 SNPs were associated with BIO14, followed by BIO2 (477 SNPs), BIO1 (443 SNPs), BIO13 (204 SNPs) and BIO4 (69 SNPs) (Table S11). For RDA, 971 SNPs were detected along five significant RDA axes, of which 509 SNPs were correlated most to BIO13, 178 SNPs to BIO1, 157 SNPs to BIO4, 64 SNPs to BIO14, and 63 SNPs to BIO2 (Table S11). Combining the two results, we recognized a total of 1,156 environment-associated SNPs. To figure out the potential function of genomic variants associated with climate adaptation, we conducted a functional annotation of outlier SNPs. Gene Ontology enrichment analysis assigned a total of 287 Gene Ontology categories ($p < 0.05$), of which 186 categories belonged to biological processes and abundant genes were associated with metabolism, transmembrane transport, methylation, stress response, photosynthetic acclimation, and flowering (Table S12).

To assess which population of *M. oleifera* will be most likely disrupted in the future (2081-2100) under two greenhouse gas scenarios (SSP126 and SSP585), we employed

gradient forest (GF) method to investigate the genomic offset (GO) using integrated results of BCC-CSM2-MR, CNRM-CM6-1 and CNRM-ESM2-1 climate models. The GO is measured by the Euclidean distance of future climate condition compared to current climate status. Higher GO means greater allele frequency changes are required to adapt to the changing climate [81]. GF modeling showed that the degree of GO of all populations increased under scenario SSP585 compared to scenario SSP126, suggesting that extreme future climate change will cause severe genomic vulnerability to *M. oleifera* (Figure 4). Compared with all SNPs (reference), we found that adaptive SNPs (candidate) exhibited higher GO under the same scenarios, implying that adaptive variants were more sensitive to climate change (Figure 4). In addition, we found a strong negative correlation between GO and altitude, with populations at higher altitudes generally having lower GO values (Figure 4).

Ecological niche modelling predicted niche suitability change

We integrated results from five models to perform ecological niche modelling for *M. oleifera* (Table S13). The Area Under the Curve (AUC) value, Somer's D value, True Skill Statistic (TSS) value, Boyce value and the area under the precision-recall gain curve (AUCprg) were about 0.99, 0.99, 0.98, 0.74 and 0.97, respectively, indicating high performance of the ecological niche models (Table S14). Compared to the current state, the potential suitable region in 2100 will reduce by 71.15 % and 98.79 % under scenarios SSP126 and SSP585, respectively, with the threshold of ecological niche suitability equal to 0.56 (Figure S12). Further, we calculated niche suitability change (NSC) between current and future climate for each grid using the equation: NSC = niche suitability index in the current climate − niche suitability index in the future climate. A positive value indicates that niche suitability will be decreased under future climate change. Higher positive value means more severe degree of unsuitability. Our results showed slightly higher NSC in the southern part of the distribution range under scenario SSP126 (Figure 5a). However, under scenario SSP585, the NSC increased to a much higher extent in the northern part of the distribution range (Figure 5b), which is the Karst basin harboring Nanpan river, Beipan river and Tuoniang river. This suggest that drastic climate change will exacerbate ecological vulnerability in karst landforms by affecting hydrological processes [82, 83].

Discussion

The genome harbors valuable evolutionary information of a species and provides deep insights into genetic diversity and evolutionary dynamics, however, the full implementation of conservation genomics in practice is still limited [84]. In this study, we conducted a conservation genomics study on *M. oleifera* based on population-wide genome resequencing data, including 165 individuals. We aim to distinguish the potential factors that affect genetic diversity of *M. oleifera* and to reveal the causes for the formation of its extremely small population patterns, and to assess its adaptability under future climate change. It is our hope that based on the comprehensive results of

conservation genomics, it will be possible to provide practical and meaningful suggestions for the conservation actions of this ecologically and economically important species.

Recent inbreeding affected genetic diversity

M. oleifera has relatively high genetic diversity among endangered woody plants (Table S15). This is confirmed by population structure result, which shows K = 14 is optimal based on all loci (Figure S7a), suggesting *M. oleifera* has complex ancestral components despite it occupies a narrow distribution. It seems optimistic, however, genetic diversity cannot determine the endangered status of a species even though it is an important criterion for species conservation [85]. Genetic diversity is affected by many factors such as inbreeding, gene flow, life form, distribution, and rarity [86]. The key to perform conservation actions for endangered species is to realize the pivotal factor affecting genetic diversity. Our results showed that populations with low genetic diversity (JM, NP and BM2) have severe degree of recent inbreeding and displayed more pronounced sawtooth-like distribution patterns of nucleotide diversity across the genome due to long ROH (Figure 3a, S3 and S4). Furthermore, high levels of inbreeding in JM, NP and BM2 populations have led to the accumulation of deleterious mutations with weaker purifying selection effects (Figure 3c and 3d), and they showed greater differentiation from other populations (Table S6), which may result in a vicious circle of inbreeding depression if without intervention [87].

Causes for the formation of small and isolated population

Historical climate disturbances were one of the reasons for the formation of currently observed small and isolated *M. oleifera* populations. Based on the estimations of Stairway Plot v.2 and MSMC v.2, we observed a bottleneck event of *M. oleifera* during the LGM, resulting in a swift decline in *Ne* (Figure 2). Although the Stairway Plot suggested the *Ne* recovered to its historical peak at the end of the LGM (Figure 2a), this inference is deemed unreliable of very recent demographic events based on site frequency spectrum (SFS) [88]. In contrast, the MSMC results suggested that the *Ne* of *M. oleifera* underwent a protracted decline after the LGM, with a slight recovery occurring approximately 500 years ago (Figure 2b). Moreover, we utilized the mean length of ROH as a metric to estimate the generations of inbreeding, referring to the method of Robinson et al. [10]. Our findings revealed that JM population had experienced the most recent inbreeding, approximately 490 years ago (Figure S9). Intriguingly, the demographic history inferred by MSMC showed that the *Ne* of *M. oleifera* reached its nadir (below 75) about 400-500 years ago (Figure 2b), which may have contributed to the extensive inbreeding. At the same time, human overexploitation and destruction of wild resources have exerted unbearable demographic pressures and resulted in further population fragmentation, as it is hard to find wild individuals again according to a large number of previous distribution records like Mashan, Pingguo, Tiandong, Tianyang, Youjiang, Longzhou counties in Guangxi province [34]. Overall,

the combined effect of historical bottleneck events, recent inbreeding, and excessive human disturbance may have led to the formation of small and isolated populations of *M. oleifera.*

Local adaptation-related alleles lead to climate change-driven genomic vulnerability

The climate is currently shifting, and many species face the challenge of keeping pace with ongoing climate changes [89, 90]. Therefore, supporting species adapt to the variable climate is a key but tough task to future conservation action. For *M. oleifera*, we detected a total of 1,156 SNPs that are related to climate adaptation. And the associated genes are significantly enriched in metabolism, transmembrane transport, methylation, stress response, photosynthetic acclimation, and flowering, showing highly vital functions (Table S12). Moreover, each climate factor is associated with dozens to hundreds of SNPs accordingly (Table S11). This is consistent with the polygenic effects underlying local adaptability, meaning that organisms can adapt to rapid climate change through small polygenic allele frequency shifts [18, 91].

Inequivalent response to climate change exists within populations of the same species, due to local adaptation to heterogeneous environments [92]. Analyzing local adaptation pattern can help us better understand how species respond to future climate change. Here, we used the Euclidean distance between future and current climate environments to measure GO. By incorporating intraspecific variations into the predictive GF model, our results showed *M. oleifera* populations distributed in the low elevation exhibit higher GO under both future scenarios (Figure 4). And elevation showed strong negative correlation with annual mean temperature ($R = -0.91$, $P < 2.2e-16$) across the distribution range of *M. oleifera* (Figure S13). This suggests populations in low altitude have a more significant adaptive lag in response to rapid climate change (especially temperature), indicating a greater risk of local extinction if appropriate conservation measures are not taken.

Ecological niche modelling provides insights into ex situ conservation

Ecological niche modelling can predict potential current distribution ranges and suitable habitats under future climate change by linking observed species distribution and abundance to selected environmental variables [93]. Our results showed the suitable habitats for *M. oleifera* will be decreased and ecological niche suitability will be further reduced in the future (Figure S12). However, the degree of niche suitability change is varied under different climate scenarios. The extreme climate (SSP585) is likely to have direct impact on hydrological processes in karst landforms, making the northern part across the distribution range which harbors Nanpan river, Beipan river and Tuoniang river becomes most unsuitable for living (Figure 5b). BM2, SG, ZL and LY1 populations that located in the area deserve highest priority for ex situ conservation when future climate becomes extremely severe. Ecological niche modelling reveals niche suitability change, while GO provides information about genomic inadaptation to

future climate change [16]. The two methods provide disparate views to estimate climate-driven vulnerability. It is necessary to combine the methods of ecological niche modelling and genomic offset to make conservation decisions.

Implications for conservation guidelines and management strategies

Currently, a wide range of field investigation and in situ protection of existing *M. oleifera* resources have been implemented [94, 95]. For example, in 2017, Guangnan County labeled 7,941 wild individuals and recorded their growth state [34]. But these measures only have limited impact on guiding future conservation actions. The conservation guidelines and management strategies should be made under demarcating reasonable management units (MUs) and adaptive units (AUs) [96]. Based on the result of population structure and phylogenetic tree (neutral loci), we suggest delineating 14 MUs of *M. oleifera* with most single population being separate MU (Figure S8). Maintaining multiple MUs ensures long-term persistence of the species. Based on adaptive loci, we identified 10 AUs, including JM, SG-NP, ML-BB2, BB1-BM2, GL, ZS, ZL-LY1, LY2-FS, BM1 and DX AUs (Figure 1b). Different AUs represent varied evolutionary potential. Understanding the patterns of adaptive differentiation is crucial when considering conservation priorities, assisted gene flow, migration, and supplementation [79].

For populations with recent inbreeding, genetic rescue is necessary through assisted gene flow [97]. JM population has the lowest genetic diversity and the highest inbreeding and genetic load, thus it needs urgent genetic rescue (Figure 3 and S3). We propose BM1 population as the prior pollen donor for JM population, because (1) it has the highest genetic diversity and heterozygosity (Figure S3 and S5); (2) it displays low genetic differentiation (Fst) with JM populations (Table S6); and (3)it has the lowest inbreeding level and fewest shared homozygous deleterious mutations with JM population (Figure S10a), positively reducing the impact of genetic load on hybrid offspring. Moreover, previous research has shown that *M. oleifera* seeds have low germination rates under natural conditions [24], so it is better to keep the seeds for artificial germination after implementing assisted gene flow measures and introduce robust seedlings back to the natural population later. GO analysis predict that populations located at lower altitudes require a greater change in adaptive allele frequencies to adapt to extreme climates (Figure 4). Low altitude areas are more susceptible to extreme temperature than higher elevations (Figure S13). Therefore, we suggest cultivating heat-resistant individuals and screening pre-adapted genotype under controlled conditions in the laboratory, and then regression experiments can be conducted. Future work should prioritize the conservation of *M. oleifera* because its lasting existence is a prerequisite to exploit resources.

Data Availability

Raw resequencing data are available at the NCBI Sequence Read Archive under BioProject PRJNA978997: https://www.ncbi.nlm.nih.gov/bioproject/PRJNA978997.

Additional Files

Supplementary note S1. Ancestral sequence reconstruction.

Supplementary note S2. Estimation of mutation rate.

Supplementary note S3. Detection of deleterious mutations based on REF-ALT strategy.

Supplementary Fig. S1. Resequencing data processing workflow of *Malania oleifera*.

Supplementary Fig. S2. Genome-wide linkage disequilibrium (LD) decay of *Malania oleifera*. (a) Considering 16 populations separately and (b) considering them as a whole.

Supplementary Fig. S3. The comparison of mean θ_π and θ_W among 16 populations of *Malania oleifera* in whole genome (a), intergenic (b), CDS (c), intron (d), fold-0 (e) and fold-4 (f) regions.

Supplementary Fig. S4. Distributions of nucleotide diversity ($\theta\pi$) across the genome with (a)(b)(c) representing NP, JM and BM2 populations (lowest average $\theta\pi$), respectively and (d) representing BM1 population (highest average $\theta\pi$).

Supplementary Fig. S5. The comparison of heterozygosity rate among 16 populations of *Malania oleifera* in whole genome (a), intergenic (b), CDS (c), intron (d), fold-0 (e) and fold-4 (f) regions.

Supplementary Fig. S6. Cross-validation error curve based on all loci (a), neutral loci (b) and adaptive loci (c) for the 16 populations of *Malania oleifera* inferred by ADMIXTURE.

Supplementary Fig. S7. The inference of population structure (a), principal component analysis (b) and NJ tree (c) of *Malania oleifera* based on all loci.

Supplementary Fig. S8. The inference of population structure (a), principal component analysis (b) and NJ tree (c) of *Malania oleifera* based on neutral loci.

Supplementary Fig. S9. Runs of homozygosity (ROH) frequency differences among 16 populations of *Malania oleifera.* The dashed lines correspond to the mean ROH lengths for each population.

Supplementary Fig. S10. The Venn diagrams of share and private homozygous deleterious mutations of JM (a), SG (b) and BM2 (c) populations with candidate pollen donors.

Supplementary Fig. S11. Differences in the number of deleterious mutations detected by REF-ALT strategy and ancestral status based strategy of *Malania oleifera* (a) and *Acer yangbiense* (b).

Supplementary Fig. S12. Integrated results of ecological niche modelling based on

five models in the current (a) and future SSP126 (b) and SSP585 (c) scenarios. Higher values represent higher suitability.

Supplementary Figure S13. Correlation of altitude with BIO1, BIO2, BIO4, BIO13 and BIO14 climate variables used in GO analysis.

Supplementary Table S1. Geographical location of all sampled individuals of *Malania oleifera*.

Supplementary Table S2. Statistical analysis of resequencing data before and after filtering with Fastp.

Supplementary Table S3. Statistical analysis of each individual mapping to the *Malania oleifera* reference genome.

Supplementary Table S4. Statistics on Watterson's θ (θW) of *Malania oleifera* populations within whole genome, CDS, fold-0, fold-4, intergenic and intron regions.

Supplementary Table S5. Statistics of heterozygosity and homozygosity rates of all individuals used in the whole genome resequencing.

Supplementary Table S6. Weighted fixation statistics (Fst) between populations based on all loci, and geographical distances between populations of *Malania oleifera*.

Supplementary Table S7. Estimation of runs of homozygosity (ROH) and frequency of runs of homozygosity (FROH).

Supplementary Table S8. Summary of deleterious, tolerated, and synonymous mutations of *Malania oleifere* based on REF-ALT strategy.

Supplementary Table S9. A list of 19 bioclimatic variables used in this study.

Supplementary Table S10. The projection coordinates of *M. oleifera* distribution records for ecological niche modelling.

Supplementary Table S11. Environment-associated SNPs and corresponding genes detected by BayeScEnv and Redundancy Analysis (RDA).

Supplementary Table S12. GO (gene ontology) enrichment analysis of environment-associated genetic variants.

Supplementary Table S13. Predicted GO and NSC values of populations under future SSP126 and SSP585 scenarios using BCC-CSM2-MR, CNRM-CM6-1 and CNRM-ESM2-1 climate models.

Supplementary Table S14. The evaluation of ecological niche models.

Supplementary Table S15. Comparison of nucleic acid diversity of endangered species.

Supplementary Table S16. Information of 17 individuals for ancestral sequence reconstruction.

Supplementary Table S17. The genome list of 17 published species used in our research to estimate mutation rate for *Malania oleifera*.

Authors' contributions

Y.P.M., W.B.S. and R.G.Z. designed the study. G.Y. collected and prepared the materials. Y.T.S., L.D.T and R.G.Z. performed the research and analyzed the data. Y.T.S. wrote the manuscript. Y.P.M. and W.B.S. revised the manuscript. All authors approved the final manuscript.

Competing Interests

We declare we have no competing interests.

Funding

This work was supported by the Key Project of Natural Science Foundation of Yunnan Province (Grant No. 202001AS070019), the CAS "Light of West China" Program and the Ten Thousand Talent Program of Yunnan Province (Grant No. YNWRQNBJ-2018-174).

Ethics Statement

All plant molecular materials and specimens were collected with permission.

References

1. Miraldo A, Li S, Borregaard MK, et al. An Anthropocene map of genetic diversity. Science 2016;353(6307):1532-35. doi:10.1126/science.aaf4381.
2. Lynch M, Conery J, Burger R. Mutation accumulation and the extinction of small populations. American Society of Naturalists 1995;146(4):489-518. doi:https://doi.org/10.1086/285812.
3. Charlesworth D, Willis JH. The genetics of inbreeding depression. Nat Rev Genet 2009;10(11):783-96. doi:10.1038/nrg2664.
4. Xue Y, Prado-Martinez J, Sudmant PH, et al. Mountain gorilla genomes reveal the impact of long-term population decline and inbreeding. Science 2015;348(6231):242-45. doi:10.1126/science.aaa3952.
5. Feng S, Fang Q, Barnett R, et al. The genomic footprints of the fall and recovery of the crested ibis. Curr Biol 2019;29(2):340-49 e7. doi:10.1016/j.cub.2018.12.008.
6. Keller MC, Visscher PM, Goddard ME. Quantification of inbreeding due to distant ancestors and its detection using dense single nucleotide polymorphism data. Genetics 2011;189(1):237-49. doi:10.1534/genetics.111.130922.
7. Ma Y, Liu D, Wariss HM, et al. Demographic history and identification of

threats revealed by population genomic analysis provide insights into conservation for an endangered maple. Mol Ecol 2022;31(3):767-79. doi:10.1111/mec.16289.

8. Hedrick PW, Garcia-Dorado A. Understanding inbreeding depression, purging, and genetic rescue. Trends Ecol Evol 2016;31(12):940-52. doi:10.1016/j.tree.2016.09.005.
9. Caballero A, Bravo I, Wang J. Inbreeding load and purging: implications for the short-term survival and the conservation management of small populations. Heredity (Edinb) 2017;118(2):177-85. doi:10.1038/hdy.2016.80.
10. Robinson JA, Kyriazis CC, Nigenda-Morales SF, et al. The critically endangered vaquita is not doomed to extinction by inbreeding depression. Science 2022;376(6593):635-39. doi:10.1126/science.abm1742.
11. Malcolm JR, Liu C, Neilson RP, et al. Global warming and extinctions of endemic species from biodiversity hotspots. Conserv Biol 2006;20(2):538-48. doi:10.1111/j.1523-1739.2006.00364.x.
12. Wiens JJ. Climate-related local extinctions are already widespread among plant and animal Species. PLoS Biol 2016;14(12):e2001104. doi:10.1371/journal.pbio.2001104.
13. Bay RA, Harrigan RJ, Underwood VL, et al. Genomic signals of selection predict climate-driven population declines in a migratory bird. Science 2018;359(6371):83-86. doi:10.1126/science.aan4380.
14. Aitken SN, Yeaman S, Holliday JA, et al. Adaptation, migration or extirpation: climate change outcomes for tree populations. Evol Appl 2008;1(1):95-111. doi:10.1111/j.1752-4571.2007.00013.x.
15. Aguirre-Liguori JA, Ramirez-Barahona S, Gaut BS. The evolutionary genomics of species' responses to climate change. Nat Ecol Evol 2021;5(10):1350-60. doi:10.1038/s41559-021-01526-9.
16. Chen Y, Jiang Z, Fan P, et al. The combination of genomic offset and niche modelling provides insights into climate change-driven vulnerability. Nat Commun 2022;13(1):4821. doi:10.1038/s41467-022-32546-z.
17. Jia KH, Zhao W, Maier PA, et al. Landscape genomics predicts climate change-related genetic offset for the widespread *Platycladus orientalis* (Cupressaceae). Evol Appl 2020;13(4):665-76. doi:10.1111/eva.12891.
18. Sang Y, Long Z, Dan X, et al. Genomic insights into local adaptation and future climate-induced vulnerability of a keystone forest tree in East Asia. Nat Commun 2022;13(1):6541. doi:10.1038/s41467-022-34206-8.
19. Yang H, Li J, Milne RI, et al. Genomic insights into the genotype-environment mismatch and conservation units of a Qinghai-Tibet Plateau endemic cypress under climate change. Evol Appl 2022;15(6):919-33. doi:10.1111/eva.13377.
20. Li SG. *Malania*, a new genus of oil-yielding plant. Bulletin of Botanical Laboratory of North-Eastern Forestry Institute 1980;1:67-72.
21. Lv SH, Wei CQ, Huang FZ, et al. Fruit and seed traits and adaptability to rocky

desertification mountain of rare tree species *Malania oleifera*. Chinese Journal of Ecology 2016;35(1):57-62. doi:10.13292/1.1000-4890.201601.008.

22. Yang T, Yu Q, Xu W, et al. Transcriptome analysis reveals crucial genes involved in the biosynthesis of nervonic acid in woody *Malania oleifera* oilseeds. BMC Plant Biol 2018;18(1):247. doi:10.1186/s12870-018-1463-6.
23. Lu SG, Lei LB, Yang QS, et al. The current status and the cause of the endangerment of *Malania oleifera* Chun et Lee in southeast Yunnan. In: Chen YY, (ed.). *Biodiversity Conservation and Regional Sustainable Development-The 4th Biodiversity Conservation and Sustainable Use Conference*. China Forestry Press, 2000, p. 169-72.
24. Xu DB, Chen F, Guo XC, et al. Research on the bottleneck of resource protection and industrialization development of rarely endangered *Malania oleifera*. Issues of Forestry Economics 2018;38(3):13-20. doi:10. 16832 /j. cnki. 1005-9709. 2018. 03. 003.
25. Wu YQ, Li XD, Hu YJ. Reproductive biology of *Malania oleifera*. Acta Scientiarum Naturalium Universitatis Sunyatseni 2004;43(2):81-83.
26. Lai JY, Shi HM, Pan CL, et al. Pollination biology of rare and endangered species *Malania oleifera* Chun et Lee. Journal of Beijing Forestry University 2008;30(2):59-64. doi:10.13332/j.1000-1522.2008.02.021.
27. Li XD. Life-table analysis of *Malania oleifera*, a rare and endangered plant. Journal of Central South University of Forestry & Technology 2009;29(2):73-76.
28. Xu SS, Kan W, Kong BH, et al. First report of *Fusarium oxysporum* and *Fusarium solani* causing root rot on *Malania oleifera* in China. Plant Disease 2020;104(2):584-84. doi:10.1094/PDIS-07-19-1426-PDN.
29. Fu LG. Red data book of Chinese plant-the rare and endangered plants. Beijing: Science Press; 1992.
30. Ma Y, Chen G, Edward Grumbine R, et al. Conserving plant species with extremely small populations (PSESP) in China. Biodiversity and Conservation 2013;22(3):803-09. doi:10.1007/s10531-013-0434-3.
31. Yang T, Zhang R, Tian X, et al. The chromosome-level genome assembly and genes involved in biosynthesis of nervonic acid of *Malania oleifera*. Sci Data 2023;10(1):298. doi:10.1038/s41597-023-02218-8.
32. Chen W, Wang P, Pu T, et al. Symbiotic effect of co-cultivated plants on *Malania oleifera* seedlings. Acta Agriculturae Universitatis Jiangxiensis 2022;44(5):1197-206. doi:10.13836/j.jjau.2022119.
33. Chen Q, Li Y, Li Y, et al. Dynamics of tissue nutrient content in relation to declining seedling growth in *Malania Oleifera*. Guihaia 2024;44(1):137-46. doi:10.11931/guihaia.gxzw202303048.
34. Su C, Wang G, Gao Y, et al. Resource protection and development counterplants of *Malania Oleifera*. J Anhui Agric Sci 2023;51(12):104-07. doi:10.3969/j.issn.0517-6611.2023.12.024.

35. Supple MA, Shapiro B. Conservation of biodiversity in the genomics era. Genome Biology 2018;19(1) doi:10.1186/s13059-018-1520-3.
36. Doyle JJ, Doyle JL. A rapid DNA isolation procedure for small quantities of fresh leaf tissue. Phytochemical Bulletin 1987;19(1):11-15. doi:10.1016/0031-9422(80)85004-7.
37. Chen S, Zhou Y, Chen Y, et al. fastp: an ultra-fast all-in-one FASTQ preprocessor. Bioinformatics 2018;34(17):i884-i90. doi:10.1093/bioinformatics/bty560.
38. Li H. Aligning sequence reads, clone sequences and assembly contigs with BWA-MEM. arXiv: Genomics 2013; doi:10.6084/M9.FIGSHARE.963153.V1.
39. Li H, Handsaker B, Wysoker A, et al. The Sequence Alignment/Map format and SAMtools. Bioinformatics 2009;25(16):2078-9. doi:10.1093/bioinformatics/btp352.
40. Tarasov A, Vilella AJ, Cuppen E, et al. Sambamba: fast processing of NGS alignment formats. Bioinformatics 2015;31(12):2032-4. doi:10.1093/bioinformatics/btv098.
41. Garrison E, Marth G. Haplotype-based variant detection from short-read sequencing. arXiv:12073907 [q-bioGN] 2012; doi:10.48550/arXiv.1207.3907.
42. Danecek P, Auton A, Abecasis G, et al. The variant call format and VCFtools. Bioinformatics 2011;27(15):2156-8. doi:10.1093/bioinformatics/btr330.
43. Zhang C, Dong SS, Xu JY, et al. PopLDdecay: a fast and effective tool for linkage disequilibrium decay analysis based on variant call format files. Bioinformatics 2019;35(10):1786-88. doi:10.1093/bioinformatics/bty875.
44. Korneliussen TS, Albrechtsen A, Nielsen R. ANGSD: Analysis of next generation sequencing data. BMC Bioinformatics 2014;15(1):356. doi:10.1186/s12859-014-0356-4.
45. Yang Y, Ma T, Wang Z, et al. Genomic effects of population collapse in a critically endangered ironwood tree *Ostrya rehderiana*. Nat Commun 2018;9(1):5449. doi:10.1038/s41467-018-07913-4.
46. Frichot E, François O, O'Meara B. LEA: An R package for landscape and ecological association studies. Methods in Ecology and Evolution 2015;6(8):925-29. doi:10.1111/2041-210x.12382.
47. Luu K, Bazin E, Blum MG. pcadapt: an R package to perform genome scans for selection based on principal component analysis. Mol Ecol Resour 2017;17(1):67-77. doi:10.1111/1755-0998.12592.
48. Purcell S, Neale B, Todd-Brown K, et al. PLINK: a tool set for whole-genome association and population-based linkage analyses. Am J Hum Genet 2007;81(3):559-75. doi:10.1086/519795.
49. Alexander DH, Novembre J, Lange K. Fast model-based estimation of ancestry in unrelated individuals. Genome Res 2009;19(9):1655-64. doi:10.1101/gr.094052.109.
50. Yang J, Lee SH, Goddard ME, et al. GCTA: a tool for genome-wide complex

trait analysis. Am J Hum Genet 2011;88(1):76-82. doi:10.1016/j.ajhg.2010.11.011.

51. Kumar S, Stecher G, Tamura K. MEGA7: Molecular evolutionary genetics analysis version 7.0 for bigger datasets. Mol Biol Evol 2016;33(7):1870-4. doi:10.1093/molbev/msw054.

52. Liu X, Fu YX. Stairway Plot 2: demographic history inference with folded SNP frequency spectra. Genome Biol 2020;21(1):280. doi:10.1186/s13059-020-02196-9.

53. Schiffels S, Durbin R. Inferring human population size and separation history from multiple genome sequences. Nat Genet 2014;46(8):919-25. doi:10.1038/ng.3015.

54. Sim NL, Kumar P, Hu J, et al. SIFT web server: predicting effects of amino acid substitutions on proteins. Nucleic Acids Res 2012;40(Web Server issue):W452-7. doi:10.1093/nar/gks539.

55. Boeckmann B, Bairoch A, Apweiler R, et al. The SWISS-PROT protein knowledgebase and its supplement TrEMBL in 2003. Nucleic Acids Res 2003;31(1):365-70. doi:10.1093/nar/gkg095.

56. Vaser R, Adusumalli S, Leng SN, et al. SIFT missense predictions for genomes. Nat Protoc 2016;11(1):1-9. doi:10.1038/nprot.2015.123.

57. Friendly M. Corrgrams: Exploratory displays for correlation matrices. The American Statistician 2002;56(4):316-24. doi:10.1198/000313002533.

58. Villemereuil P, Gaggiotti OE. A new FST-based method to uncover local adaptation using environmental variables. Methods in Ecology and Evolution 2015;6(11):1248-58. doi:10.1111/2041-210x.12418.

59. Legendre P, Legendre L. Numerical ecology. Elsevier; 2012.

60. Lischer HE, Excoffier L. PGDSpider: an automated data conversion tool for connecting population genetics and genomics programs. Bioinformatics 2012; 28(2):298-9. doi: 10.1093/bioinformatics/btr642.

61. Forester BR, Lasky JR, Wagner HH, et al. Comparing methods for detecting multilocus adaptation with multivariate genotype-environment associations. Mol Ecol 2018;27(9):2215-33. doi:10.1111/mec.14584.

62. Oksanen J, Blanchet FG, Kindt R, et al. Package ‘vegan’: Community ccology package. 2013. R package version 2.3-0. https://cran.r-project.org/web/packages/vegan/index.html.

63. Cantalapiedra CP, Hernandez-Plaza A, Letunic I, et al. eggNOG-mapper v2: Functional annotation, orthology assignments, and domain prediction at the metagenomic scale. Mol Biol Evol 2021;38(12):5825-29. doi:10.1093/molbev/msab293.

64. Ellis N, Smith SJ, Pitcher CR. Gradient forests: calculating importance gradients on physical predictors. Ecology 2012;93(1):156-68. doi:10.1890/11-0252.1.

65. Yu X, Dai M, Pu T, et al. Population structure and dynamics analysis of rare and

endangered plant *Malania oleifera*. Journal of West China Forestry Science 2023;52(3):8-16. doi: 10.16473/j.cnki.xblykx1972.2023.03.002.

66. Gong MJ, Wang J, Fu XY, et al. Suitable regions forecasting and environmental influencing factors of *Malania oleifera* in Yunnan and Guangxi. Journal of Nanjing Forestry University (Natural Sciences Edition) 2022;46(2):44-52. doi:10. 12302/j. issn. 1000-2006. 202109039.
67. Brown JL, Carnaval AC. A tale of two niches: methods, concepts, and evolution. Frontiers of Biogeography 2019;11(4) doi:10.21425/f5fbg44158.
68. Naimi B, Hamm NAS, Groen TA, et al. Where is positional uncertainty a problem for species distribution modelling? Ecography 2013;37(2):191-203. doi:10.1111/j.1600-0587.2013.00205.x.
69. Wood SN. Fast stable restricted maximum likelihood and marginal likelihood estimation of semiparametric generalized linear models. J R Statist Soc B 2011;73:3-36. doi: 10.1111/j.1467-9868.2010.00749.x.
70. Hijmans RJ, Phillips S, Leathwick J, et al. dismo: Species distribution Modelling. 2023. R package version 1.3-14. https://CRAN.R-project.org/package=dismo.
71. Liaw A, Wiener M. Classification and regression by randomForest. R news 2002;2:18-22.
72. Friedman J, Tibshirani R, Hastie T. Regularization paths for generalized linear models via coordinate descent. J Stat Softw 2010;33(1):1-22. doi:10.18637/jss.v033.i01.
73. Valavi R, Guillera-Arroita G, Lahoz-Monfort JJ, et al. Predictive performance of presence-only species distribution models: a benchmark study with reproducible code. Ecol Monogr 2022; 92: e01486. doi: 10.1002/ecm.1486
74. Wilfried T, Damien G, Maya G, et al. biomod2: Ensemble platform for species distribution modeling. 2023. R package version 4.2-4. https://CRAN.R-project.org/package=biomod2.
75. Di Cola V, Broennimann O, Petitpierre B, et al. ecospat: an R package to support spatial analyses and modeling of species niches and distributions. Ecography 2017;40(6):774-87. doi:10.1111/ecog.02671.
76. Kull M, Flach P. prg: creates the Precision-Recall-Gain curve and calculates the area under the curve. 2023. R package version 0.5.1. https://github.com/meeliskull/prg.
77. Elizabeth F. PresenceAbsence: Presence-Absence Model Evaluation. 2023. R package version 1.1.11. https://CRAN.R-project.org/package=PresenceAbsence.
78. Guzmán S, Giudicelli GC, Turchetto C, et al. Neutral and outlier single nucleotide polymorphisms disentangle the evolutionary history of a coastal Solanaceae species. Mol Ecol 2022;31(10):2847-64. doi:10.1111/mec.16441.
79. Funk WC, McKay JK, Hohenlohe PA, et al. Harnessing genomics for delineating conservation units. Trends Ecol Evol 2012;27(9):489-96.

doi:10.1016/j.tree.2012.05.012.

80. Hu JY, Hao ZQ, Frantz L, et al. Genomic consequences of population decline in critically endangered pangolins and their demographic histories. Natl Sci Rev 2020;7(4):798-814. doi:10.1093/nsr/nwaa031.

81. Fitzpatrick MC, Keller SR. Ecological genomics meets community-level modelling of biodiversity: mapping the genomic landscape of current and future environmental adaptation. Ecol Lett 2015;18(1):1-16. doi:10.1111/ele.12376.

82. Rao W, Shen Z, Duan X. Spatiotemporal patterns and drivers of soil erosion in Yunnan, Southwest China: Rulse assessments for recent 30 years and future predictions based on CMIP6. Catena 2023;220 doi:10.1016/j.catena.2022.106703.

83. Liu H, Zhang M, Lin Z, et al. Spatial heterogeneity of the relationship between vegetation dynamics and climate change and their driving forces at multiple time scales in Southwest China. Agricultural and Forest Meteorology 2018;256-257:10-21. doi:10.1016/j.agrformet.2018.02.015.

84. Theissinger K, Fernandes C, Formenti G, et al. How genomics can help biodiversity conservation. Trends in Genetics 2023;39(7):545-59. doi:10.1016/j.tig.2023.01.005.

85. Kahilainen A, Puurtinen M, Kotiaho JS. Conservation implications of species–genetic diversity correlations. Global Ecology and Conservation 2014;2:315-23. doi:10.1016/j.gecco.2014.10.013.

86. He ZZ, Stotz GC, Liu X, et al. A global synthesis of the patterns of genetic diversity in endangered and invasive plants. Biological Conservation 2024;291 doi:10.1016/j.biocon.2024.110473.

87. Ellegren H, Galtier N. Determinants of genetic diversity. Nat Rev Genet 2016;17(7):422-33. doi:10.1038/nrg.2016.58.

88. Chen H, Hey J, Chen K. Inferring very recent population growth rate from population-scale sequencing data: Using a large-sample coalescent estimator. Mol Biol Evol 2015;32(11):2996-3011. doi:10.1093/molbev/msv158.

89. Dawson TP, Jackson ST, House JI, et al. Beyond predictions: biodiversity conservation in a changing climate. Science 2011;332(6025):53-8. doi:10.1126/science.1200303.

90. Pacifici M, Foden WB, Visconti P, et al. Assessing species vulnerability to climate change. Nature Climate Change 2015;5(3):215-24. doi:10.1038/nclimate2448.

91. Fagny M, Austerlitz F. Polygenic adaptation: Integrating population genetics and gene regulatory networks. Trends Genet 2021;37(7):631-638. doi: 10.1016/j.tig.2021.03.005.

92. Yuan S, Shi Y, Zhou BF, et al. Genomic vulnerability to climate change in *Quercus acutissima*, a dominant tree species in East Asian deciduous forests. Mol Ecol 2023;32(7):1639-55. doi:10.1111/mec.16843.

93. Thuiller W. Ecological niche modelling. Current Biology 2024;34(6):R225-R29.

doi:10.1016/j.cub.2024.02.018.

94. Jia D, Mao J, Chen F, et al. Investigation and analysis of wild garlic fruit resources in Guangnan. Forest By-Product and Speciality in China 2017;3:72-76. doi:10.13268/j.cnki, fbsic.2017.03.032.

95. Liu Y, Ning S. Status and evaluation of natural resources of emphasis protective wilding plant in Guangxi. Guangxi Sciences 2002;9(2):124-32. doi:10.13656/j.cnki, gxkx.2002.02.012.

96. Barbosa S, Mestre F, White TA, et al. Integrative approaches to guide conservation decisions: Using genomics to define conservation units and functional corridors. Mol Ecol 2018;27(17):3452-65. doi:10.1111/mec.14806.

97. Pavlova A, Beheregaray LB, Coleman R, et al. Severe consequences of habitat fragmentation on genetic diversity of an endangered Australian freshwater fish: A call for assisted gene flow. Evol Appl 2017;10(6):531-50. doi:10.1111/eva.12484.

Table

Table 1. Sample sizes and nucleotide diversity (θ_π) in *M. oleifera* populations within the whole genome, CDS, fold-0, fold-4, intergenic and intron regions.

**Population**	**Sample size**	**Number of SNPs**	**θ_π_whole × 10^{-3}**	**θ_π_CDS × 10^{-3}**	**θ_π_fold-0 × 10^{-3}**	**θ_π_fold-4 × 10^{-3}**	**θ_π_intergenic × 10^{-3}**	**θ_π_intron × 10^{-3}**
BB1	10	141725	3.10 ± 0.42	1.19 ± 0.15	0.99 ± 0.13	1.93 ± 0.23	3.52 ± 0.49	1.83 ± 0.18
BB2	10	132480	2.79 ± 0.41	1.08 ± 0.16	0.90 ± 0.13	1.75 ± 0.25	3.17 ± 0.47	1.68 ± 0.24
BL	10	146763	3.56 ± 0.47	1.38 ± 0.17	1.15 ± 0.14	2.19 ± 0.27	4.07 ± 0.57	2.12 ± 0.25
BM1	17	218460	6.13 ± 0.58	2.41 ± 0.18	2.00 ± 0.15	3.92 ± 0.26	7.56 ± 0.44	3.87 ± 0.20
BM2	5	101436	2.15 ± 0.25	0.86 ± 0.13	0.73 ± 0.11	1.34 ± 0.17	2.45 ± 0.29	1.30 ± 0.13
DX	10	155019	4.57 ± 0.57	1.73 ± 0.27	1.44 ± 0.22	2.77 ± 0.43	5.20 ± 0.62	2.71 ± 0.39
FS	9	189667	5.13 ± 0.41	1.94 ± 0.22	1.61 ± 0.18	3.15 ± 0.31	5.86 ± 0.45	3.04 ± 0.26
GL	10	137835	3.56 ± 0.45	1.35 ± 0.18	1.13 ± 0.15	2.13 ± 0.26	4.11 ± 0.52	2.05 ± 0.26
JM	10	98031	2.07 ± 0.40	0.83 ± 0.20	0.69 ± 0.17	1.34 ± 0.30	2.36 ± 0.46	1.25 ± 0.22
LY1	10	157624	3.89 ± 0.47	1.47 ± 0.22	1.22 ± 0.19	2.39 ± 0.33	4.47 ± 0.52	2.25 ± 0.31
LY2	14	200098	5.50 ± 0.27	2.03 ± 0.19	1.68 ± 0.16	3.28 ± 0.26	6.28 ± 0.27	3.20 ± 0.21
ML	10	151563	3.53 ± 0.40	1.34 ± 0.18	1.12 ± 0.15	2.13 ± 0.26	4.05 ± 0.47	2.08 ± 0.21
NP	10	106945	2.03 ± 0.48	0.82 ± 0.25	0.69 ± 0.20	1.30± 0.38	2.31 ± 0.54	1.25 ± 0.30
SG	10	133980	2.75 ± 0.34	1.10 ± 0.20	0.91 ± 0.17	1.80 ± 0.30	3.12 ± 0.40	1.65 ± 0.18
ZL	10	169320	4.48 ± 0.41	1.67 ± 0.21	1.41 ± 0.17	2.61 ± 0.32	5.15 ± 0.43	2.59 ± 0.29
ZS	10	163672	4.35 ± 0.32	1.63 ± 0.14	1.36 ± 0.12	2.60 ± 0.21	4.99 ± 0.36	2.55 ± 0.20

Figures

Figure 1. Population genomics of *M. oleifera.* (a) Geographic distribution and sampled populations of *M. oleifera*. Different colors in the pie chart represent the genetic groups identified by ADMIXTURE based on adaptive loci, and the size of the pie corresponds to the level of heterozygosity. The optimal population genetic structure of *M. oleifera* with K = 10 (b) and a neighbor-joining (NJ) phylogenetic tree (c) based on adaptive loci. Samples in the STRUCTURE and phylogenetic tree results correspond. Node bootstrap values below 0.8 are not shown. (d) Results of PCA based on adaptive loci, with the first two PCs explaining 25.2% of the genome covariance. Populations are defined as BB1 = Banbeng, BB2 = Babao, BL = Banlun, BM1 = Bama, BM2 = Bamei, DX = Daxin, FS = Fengshan, GL = Gaolong, JM = Jiumo, LY1 = Leye, LY2 = Linyun, ML = Mulun, NP = Nanping, SG = Shuguang, ZL = Zhemiao, ZS = Zhesang.

Figure 2. Demographic history of *M. oleifera* inferred by Stairway Plot v.2 (a) and MSMC v.2 within two, four and eight haplotypes (b). The light blue lines correspond to the upper and lower bounds of the 95% confidence intervals. The severe effective population size (*Ne*) declines observed during the last glacial maximum (LGM) and the Middle Pleistocene are highlighted with gray vertical bars.

Figure 3. Levels of inbreeding and genetic load in different *M. oleifera* populations. (a) Fractions of the runs of homozygosity (FROH) show discrepancies in inbreeding levels in *M. oleifera* populations. (b) Distributions of long (> 1 Mb) and medium (100 kb-1 Mb) runs of homozygosity (ROH) among 16 populations of *M. oleifera.* Solid dots represent ROH > 1 Mb and hollow dots represent 1 Mb > ROH > 100 Kb. (c) Ratios of homozygous-derived deleterious mutations show the discrepancies in genetic load in different *M. oleifera* populations. Populations marked with the same letters in (a) and (c) are not significantly different. (d) Distributions of the ratio of 0- to 4-fold heterozygosity versus the intergenic heterozygosity across the 16 populations of *M. oleifera*. The dark line represents the significant negative correlation between these populations, with R = -0.21 and p = 0.006. Each dot represents an individual, which is colored by population.

Figure 4. Predicted genetic offset of *M. oleifera* in the year 2100 under the SSP126 and SSP585 scenarios based on all SNPs (a, b) and adaptive SNPs (c, d), with higher values (red) representing more severe genomic vulnerability to future climate change. The inner mini plot represents the correlation between altitude and GO value in the corresponding scenario.

Figure 5. Predicted niche suitability change (NSC) of *M. oleifera* in the year 2100 under the SSP126 (a) and SSP585 (b) scenarios. Higher positive value suggests more severe degree of unsuitability.

Figure1

Click here to access/download;Figure;Figure 1.jpg

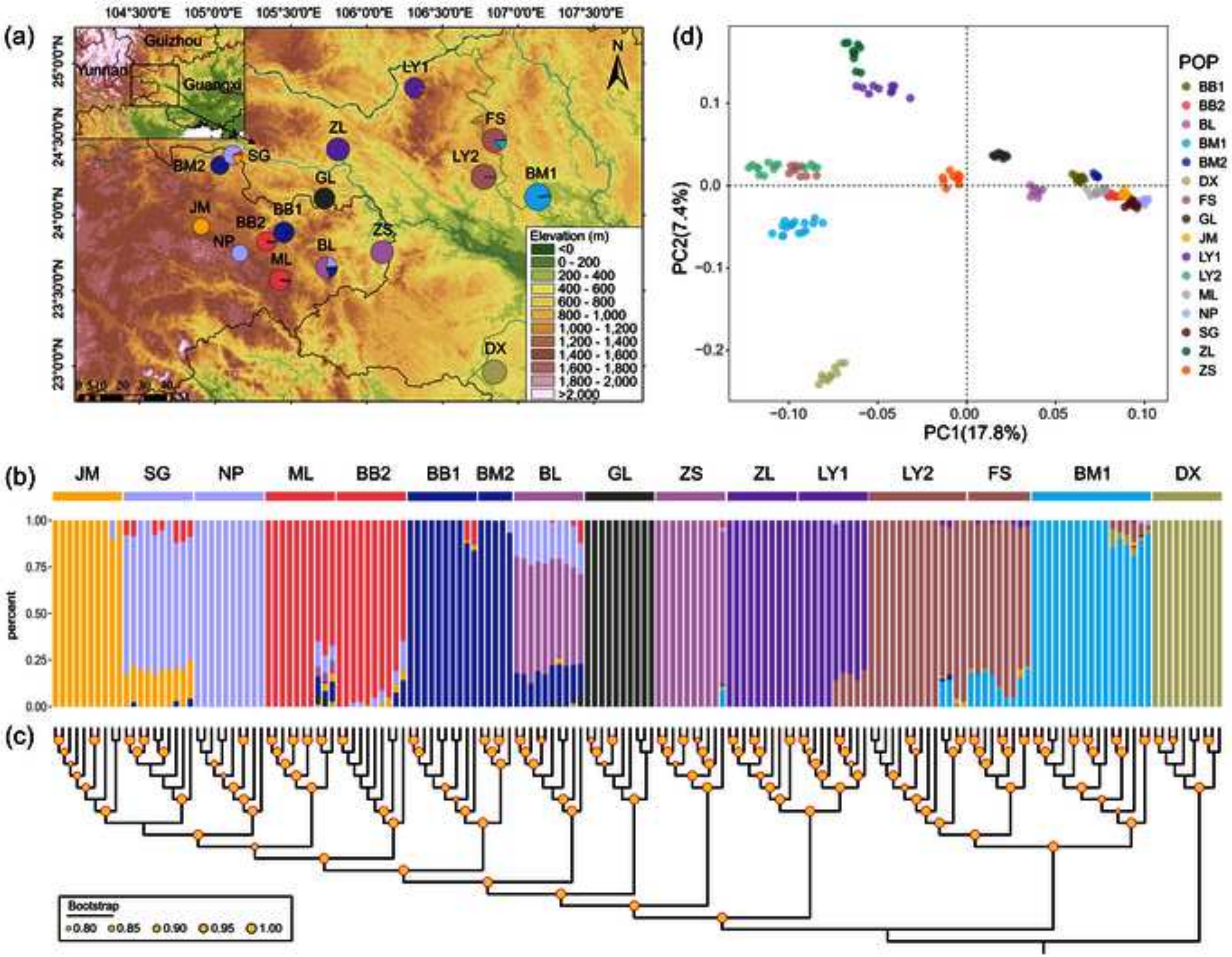

Figure2

Click here to access/download;Figure;Figure 2.jpg

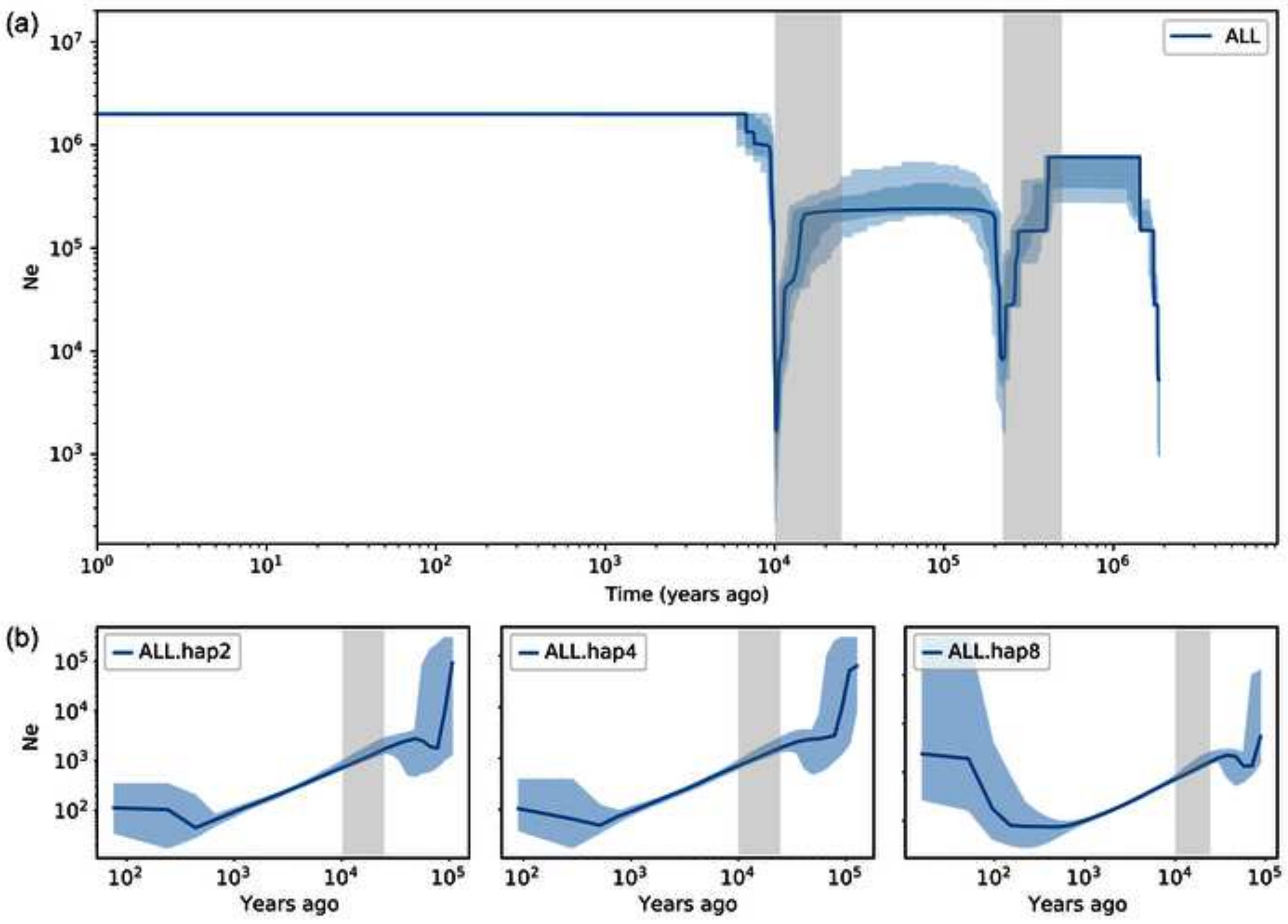

Figure3

Click here to access/download;Figure;Figure 3.jpg

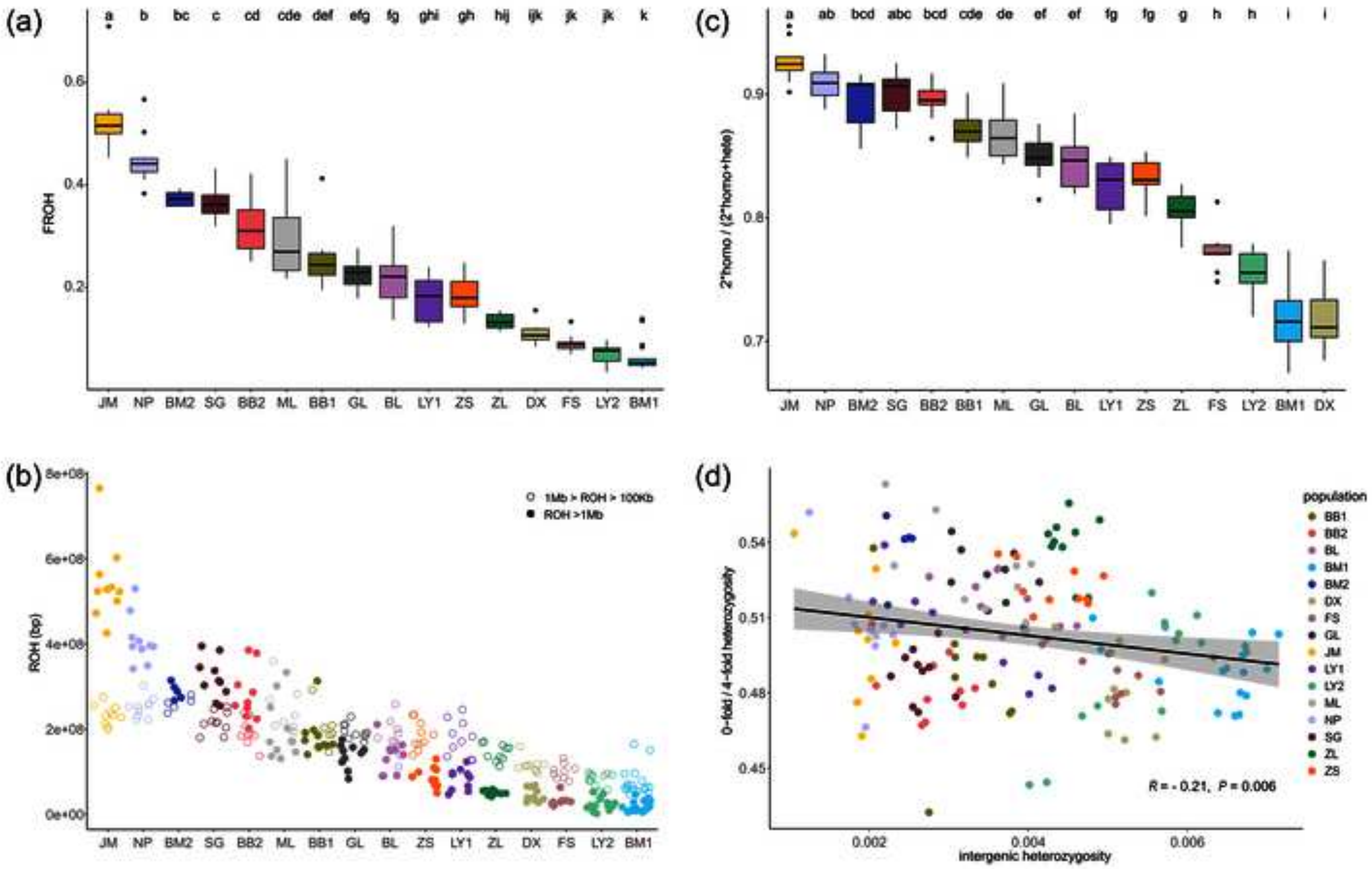

Figure4

Click here to access/download;Figure;Figure 4.jpg

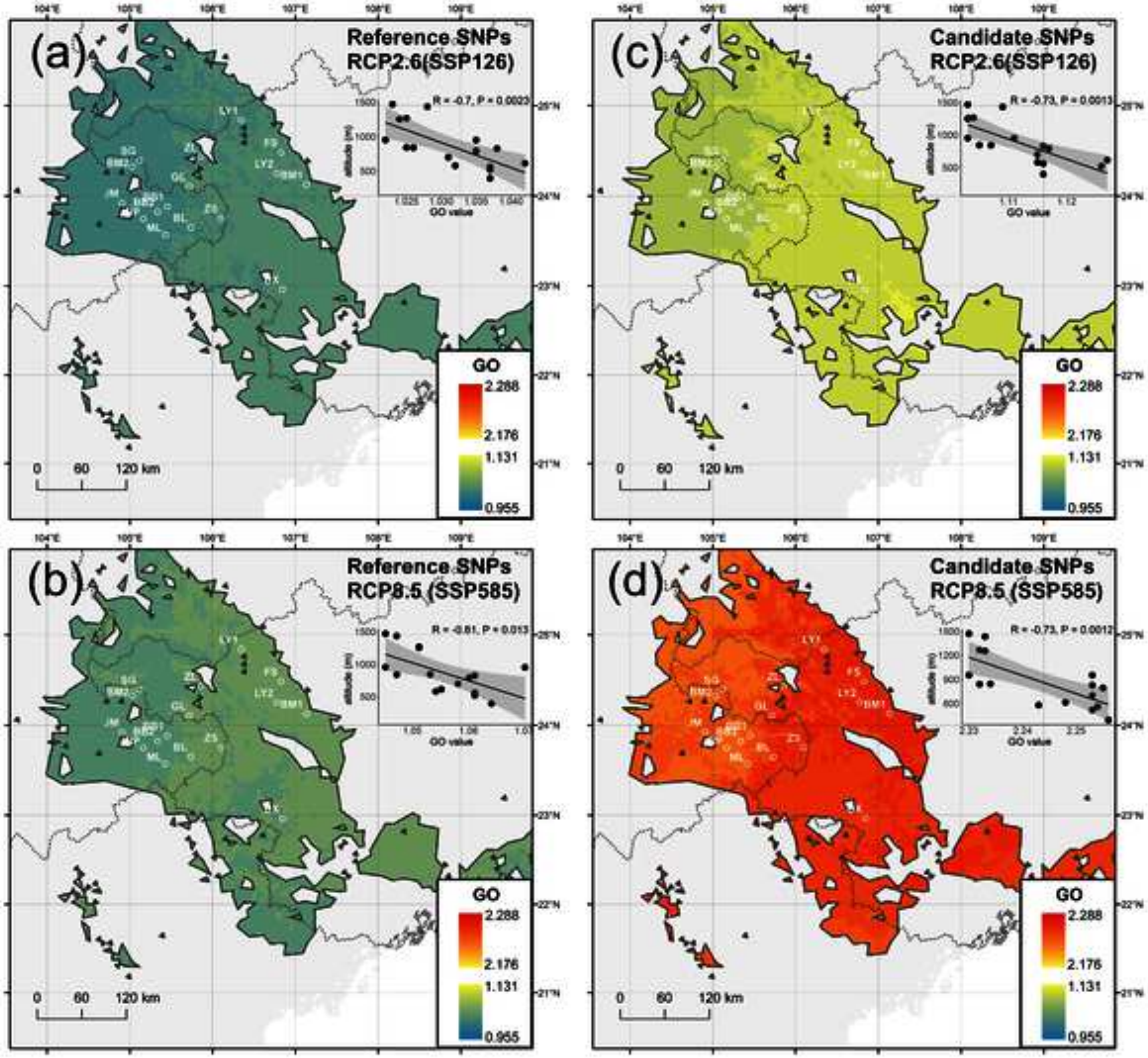

Figure5

Click here to access/download;Figure;Figure 5.jpg

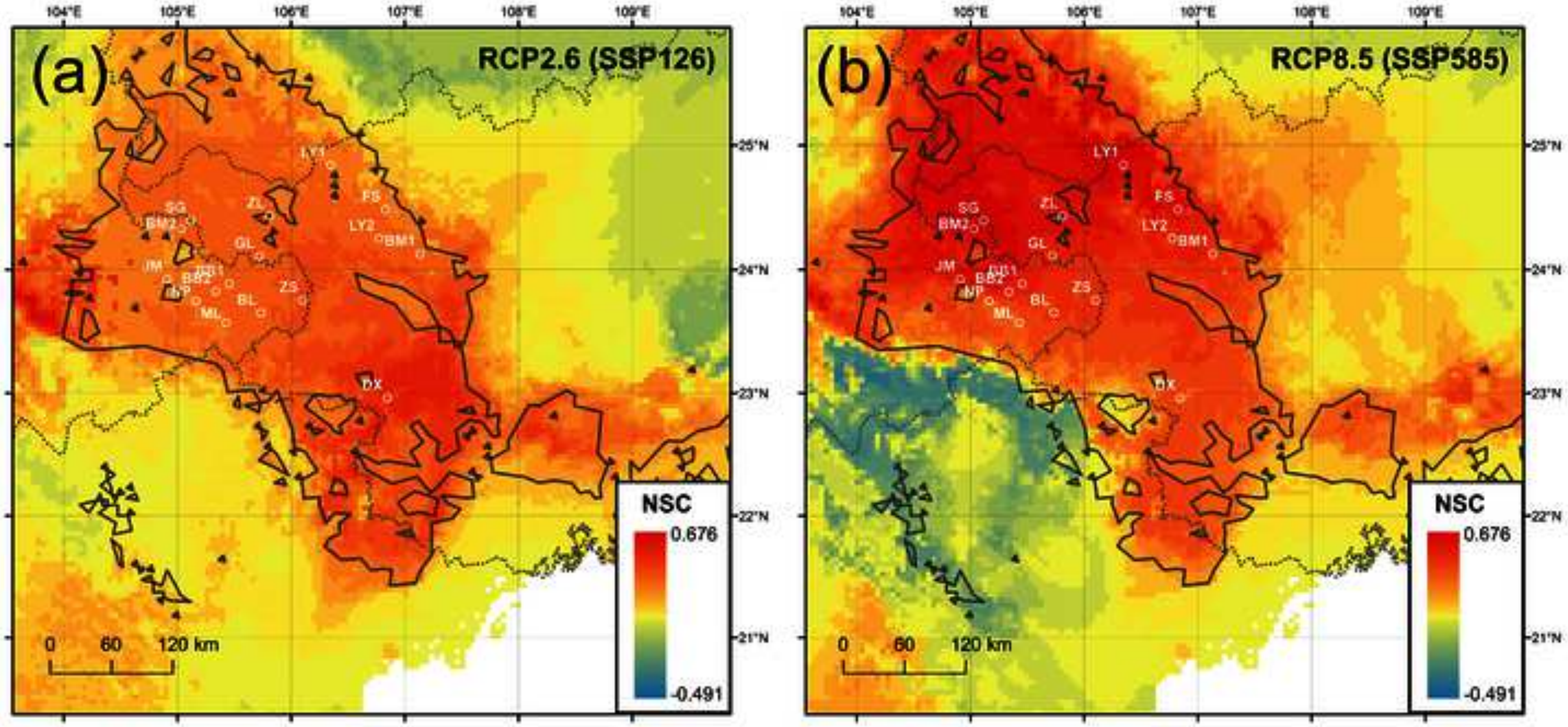

Click here to access/download
Supplementary Material
Supplementary note S1-S3.docx

Click here to access/download
Supplementary Material
Supplementary figure S1-S13.docx

Click here to access/download
Supplementary Material
supplementary table S1-S17.xlsx

response to reviewers

Click here to access/download;Personal Cover;response to reviewers.docx

Reviewer #1:

In the manuscript titled "Genomic insights into endangerment and conservation of the garlic-fruit tree (*Malania oleifera*), a plant species with extremely small populations," the author conducted a comprehensive conservation genomic analysis on *Malania oleifera* to investigate the reasons behind its extremely small population sizes and the potential extent of genotype-environment mismatch under future climate change. As a result, 10 adaptive units were identified, and conservation guidelines and management strategies for *M. oleifera* were proposed from a genomic perspective, The manuscript offers valuable insights for the conservation of *M. oleifera* and other endangered species. However, I believe there are still some issues in the manuscript that need improvement. Several suggestions are listed below.

①In the method section (page 11, line 38), the statement 'with coverage depth below 300× and above 1200×,' How was this parameter determined?

Response: We are sorry for the unclear description. This parameter was calculated by VCFtools v. 0.1.15 with "--site-depth". The average site coverage is about 600× and we discarded the sites with coverage depth below 1/2*average site coverage and above 2*average site coverage to keep the only one peak. The relevant description is revised in the resubmitted manuscript (page 4, line 17-18).

②In method BayeScEnv (page 14, line 17)，could you provide an explanation of how environmental factors were standardized and how codominant data were converted.

Response: Thanks for the comment. Each environmental factor was extracted through the coordinates of the sampling points, and it needs to be standardized by the mean variance of all individuals. Codominant data were converted by PGDSpider v. 2.1.1.5 based on vcf file (dataset 3). The relevant description is revised in the resubmitted manuscript (page 6, line 22-24).

③How were adaptive loci (dataset 7) and neutra loci (dataset 8) identified, and what was the rationale behind using three datasets for fst analysis.

Response: Thanks for pointing out the issue. Page 5, line 3-16 shows a detailed description about detecting adaptive and neutral loci. It is a general pattern that Fst based on adaptive loci is significantly higher than Fst based on all loci and neutral loci. The reason we used

three datasets for Fst analysis is that we would like to see if there was an exception in *M. oleifera*, with higher Fst based on all loci or neutral loci, but lower Fst based on adaptive loci. If there are such paired populations, it means they have similar adaptability and can serve as potential genetic rescue population for each other. But unfortunately, we didn't find such paired populations. Therefore, we only retained the results of Fst based on all loci in the revised manuscript (page 9, line 12-27).

④ In the section "Population structure and genetic differentiation ", you mentioned that "ADMIXTURE analysis based on adaptive loci indicated that K = 10 was optimal (Figure S6), implying more pairs of populations have similar genetic composition." Could you elaborate on the implications of this result and how it supports specific conclusions in your study?

Response: We are sorry for the unclear description. The sentence is revised with details (page 9, line 1-4). The result can be used to delimit adaptive units (AUs). Please see more specific conclusions in the discussion part (page 14, line 14-19).

⑤On page 6, you mentioned calculating Fst based on all loci, neutral loci and adaptive loci. Please clarify the purpose or significance of performing these calculations? What insights or conclusion do these Fst values provide in the context of your manuscript.

Response: Please see answer for the third comment. We have deleted the results of Fst based on adaptive and neutral loci in the revised manuscript.

⑥ On page 7, line 18, you stated, "inbreeding occurred about 49 to 112 generations ago", but it's unclear how this conclusion was derived. Please provide a more detailed explanation or reference to the specific methods or data that led to this conclusion.

Response: Thanks for pointing out the problem. Detailed descriptions are as follows. “Referring to the method of Robinson et al (2022), we used the physical length of ROH to estimate the number of generations to the common ancestor (g) as g = 100/(2*L), where L is the mean length of ROH in megabases (Mb). Here, the L of all 16 populations of *M. oleifera* ranged from 0.46 Mb (BM1) to 1.03 Mb (JM) (Figure S9). Our results indicated that inbreeding occurred about 49 to 112 generations ago (Figure S9).” See page 9, line 33-37.

⑦According to the results in "Characterization of runs of homozygosity and deleterious mutations", the JM population harbored the highest inbreeding level, however, in the results of the GF analysis, the BM2 and SG populations have the highest genomics vulnerability, how can this be explained.

Response: Genomics vulnerability is related to the frequency of adaptive alleles in the population and the degree of climate change, but not related to the inbreeding level. Higher GO means greater allele frequency changes are required to adapt to the changing climate (Fitzpatrick et al., 2015). And in our new GF results, populations distributed in the low elevation exhibit higher GO rather than BM2 and SG populations. Please see answer ⑧ for more information.

⑧In the discussion section, you mentioned that the stripe from northwest to southeast regions show relatively high genomic vulnerability. Can you provide further discussion within the context of climate and geographic data to explore possible correlations or explanations for these findings?

Response: Thanks for your suggestion. We redid gradient forest (GF) analysis to investigate the genomic offset (GO) using integrated results of BCC-CSM2-MR, CNRM-CM6-1 and CNRM-ESM2-1 climate models. Our results showed *M. oleifera* populations distributed in the low elevation exhibit higher GO under both future scenarios (Figure 4). This suggests populations in low altitude have a more significant adaptive lag in response to rapid climate change (especially temperature), indicating a greater risk of local extinction. See page 11, line 1-13 and page 13, line 21-28 for more detailed information.

Reviewer #2:

In this manuscript, the authors generated the whole genome re-sequencing data for 165 samples covering 16 groups of *Malania oleifera*. Based on these data, the genetic diversity, population structure and demographic history were studied. Finally, the authors claimed that "The basic realizations concerning the threats to *M. oleifera* provide scientific foundation for defining management and adaptive units, prioritizing populations for conservation, and establishing targeted genetic rescue measures." To my knowledge, the represented results here can NOT support this conclusion. The sequenced samples were classified into 16 groups, more detailed information should be provided for readers to

understand their relationships. Could the evolutionary history of these samples be revealed to some extent? I suggest that more comprehensive analyses of population genetics and some important morphological features such as nervonic acid be conducted by integrating the whole genome sequences with other data.

Response: Thanks for pointing out the problem. We have taken full account of your suggestions. We did ADMIXTURE, PCA and Fst analysis, and constructed a NJ tree of *M. oleifera*, which have clarified the phylogenetic relationships of the 16 populations (Page 8-9). We have used Stairway plot2 and MSMC2 to reveal the demographic history of *M. oleifera* (Page 9, line19-30). Moreover, we added ecological niche modelling analysis which shows the suitable habitats will decrease by 71.15 % and 98.79 % in 2100 under scenarios SSP126 and SSP585, respectively (Pages 7 and 11). And we re-performed the GF analysis using integrated results of multiple climate models and get an interesting result that populations distributed in the low elevation exhibit higher GO due to the significant effect of higher average annual temperature (Pages 7 and 10). These results provide insights into ex situ conservation under future climate change, and we suggest screening pre-adapted and heat-resistant genotype under controlled conditions in the laboratory (See Discussion, pages 13 and 14).

We aim to distinguish the potential factors that affect genetic diversity of *M. oleifera* and to reveal the causes for the formation of its extremely small population patterns, and to assess its adaptability under future climate change. We believe this revised manuscript fully reached the aim. As for the comment "some important morphological features such as nervonic acid be conducted by integrating the whole genome sequences with other data". It is meaningful and constructive, but we currently could not do this because extraction and analysis of nervonic acid from fruits collected from the re-sequenced plants will need lots of costs and time. Additionally, it has been reported that the production of nervonic acid can be largely affected by temperature, with individuals growing in areas with higher temperature generally producing larger amount of nervonic acid (Wang et al., 2021). Therefore, we do not think this point is essential to the conservation focus of the resubmitted paper.

References:

Fitzpatrick MC, Keller SR. Ecological genomics meets community-level modelling of biodiversity: mapping the genomic landscape of current and future environmental adaptation. Ecol Lett 2015;18(1):1-16. doi:10.1111/ele.12376.

Robinson JA, Kyriazis CC, Nigenda-Morales SF, et al. The critically endangered vaquita is not doomed to extinction by inbreeding depression. Science 2022;376(6593):635-39. doi:10.1126/science.abm1742.

Wang SH, Chen J, Yang W, et al. Fruiting character variability in wild individuals of *Malania oleifera*, a highly valued endemic species. Sci Rep 2021;11(1):23605. doi: 10.1038/s41598-021-03080-7.
