# Supplementary material for: Genomic insights into endangerment and conservation of the garlic-fruit tree (Malania oleifera), a plant species with extremely small populations
Source: Gigascience. 2024 Sep 23;13:giae070. doi: 10.1093/gigascience/giae070 (PMC11417964; doi:10.1093/gigascience/giae070)
Supplement: giae070_GIGA-D-24-00159_Revision_1 [file giae070_giga-d-24-00159_revision_1.pdf]

GigaScience

Genomic insights into endangerment and conservation of the garlic-fruit tree (Malania oleifera), a plant species with extremely small populations

--Manuscript Draft--

**Manuscript Number:**	GIGA-D-24-00159R1
**Full Title:**	Genomic insights into endangerment and conservation of the garlic-fruit tree (Malania oleifera), a plant species with extremely small populations
**Article Type:**	Research
**Funding Information:**	Key Project of Natural Science Foundation of Yunnan Province (202001AS070019) — Dr. Yongpeng Ma<br>the CAS "Light of West China" Program — Dr. Yongpeng Ma<br>Ten Thousand Talent Program of Yunnan Province (YNWRQNBJ-2018-174) — Dr. Yongpeng Ma
**Abstract:**	Background<br><br>Advanced whole genome sequencing techniques enable covering nearly all genome nucleotide variations, thus can provide deep insights into protecting endangered species. However, the use of genomic data to make conservation strategies is still rare, particularly for endangered plants. Here we performed comprehensive conservation genomic analysis for Malania oleifera, an endangered tree species with high amount of nervonic acid. We used whole-genome resequencing data of 165 samples, covering 16 populations across the entire distribution range to investigate the formation reasons of its extremely small population sizes and to evaluate the possible genomic offsets and changes of ecology niche suitability under future climate change.<br><br>Results<br><br>Although M. oleifera maintains relatively high genetic diversity among endangered woody plants ($\theta\pi$ = 3.87e-3), high levels of inbreeding have been observed, which has reduced genetic diversity in three populations (JM, NP and BM2) and caused the accumulation of deleterious mutations with weaker purifying selection effects. Repeated bottleneck events, recent inbreeding (~490 years ago) and anthropogenic disturbance to wild habitats have aggravated the fragmentation and endangered status of M. oleifera. Due to the significant effect of higher average annual temperature, populations distributed in low altitude exhibit greater genomic offset. Furthermore, ecological niche modelling shows the suitable habitats for M. oleifera will decrease by 71.15% and 98.79% in 2100 under scenarios SSP126 and SSP585, respectively.<br><br>Conclusions<br><br>The basic realizations concerning the threats to M. oleifera provide scientific foundation for defining management and adaptive units, and prioritizing populations for genetic rescue. Meanwhile, we highlight the importance of integrating genomic offset and ecological niche modeling to make targeted conservation actions under future climate change. Overall, our study provides a paradigm for genomics-directed conservation.
**Corresponding Author:**	Yongpeng Ma<br>Kunming Institute of Botany Chinese Academy of Sciences<br>Kunming, CHINA
**Corresponding Author Secondary Information:**	
**Corresponding Author's Institution:**	Kunming Institute of Botany Chinese Academy of Sciences
**Corresponding Author's Secondary Institution:**	
**First Author:**	Yuanting Shen

Powered by Editorial Manager® and ProduXion Manager® from Aries Systems Corporation

**First Author Secondary Information:**	
**Order of Authors:**	Yuanting Shen
	Lidan Tao
	Gang Yao
	Minjie Zhou
	Rengang Zhang
	Weibang Sun
	Yongpeng Ma
**Order of Authors Secondary Information:**	
**Response to Reviewers:**	Reviewer #1: This article presents a fascinating case that combines genomic and ecological vulnerability to help protect endangered plants. I enjoyed the analyses, particularly the use of genomic offset to predict genomic vulnerability. This approach takes into account the entire genome's environment-related variations, rather than focusing on single SNP. I have a few minor comments that could improve the manuscript's readability.<br>Response: Thanks for the positive assessment. We have revised our manuscript according to your below comments.<br>1. According to general reading customs, readers are usually interested in understanding the population structure before delving into population factors such as LD, nucleotide diversity, and heterozygosity at the population level. Therefore, I suggest that the author swap the order of the first two paragraphs in the results section to provide a better flow of information.<br>Response: Thanks for your advice. We have swapped the order of the first two paragraphs in the results section. Please see page 8 for details.<br>2. It would be helpful to use three-letter abbreviations for the populations. The current use of two-letter abbreviations, such as BB1 and BB2 or BM1 and BM2, can lead to confusion since these populations were not closely related geographically.<br>Response: Thanks for pointing out this deficiency. To keep consistency with the name of raw data that we have already uploaded to NCBI, we still use the current naming convention. To avoid confusing readers, we have provided detailed descriptions of the abbreviations for each population in the legend of figure 1. See page 26, line 10-12.<br>3. Since "GO" represents genomic offset throughout the article, I recommend deleting the repeated abbreviation "GO" for gene ontology in the title of Supplementary Table S12.<br>Response: Revised.<br>4. Are there any references that support the selection of low Fst populations for genetic rescue? Alternatively, is there weak reproductive isolation among populations with high Fst? If not, why not consider using populations with high Fst and lower deleterious accumulations? High Fst combination will generate a high heterozygosity, which will greatly use heterozygous advantage. So, the second reason (Page 14: Lines 23-24) you provided does not persuade me, and I would appreciate additional clarification or references to support the chosen approach.<br>Response: Thanks for the reviewer's constructive comment. Without knowing the changing relationships between Fst and reproductive isolation among populations in advance, it is not rigorous to propose genetic rescue program. In some cases, high Fst combination among parents generated offspring with high heterozygosity, which may produce heterozygous advantage, however, in most cases, outbreeding depression occurred (Frankham et al., 2010). It may occur between interpopulation crossing even in the same species (Severns, 2013). So, to minimize the risk of outbreeding depression, we do not consider DX population as a potential pollen donor because it has highest Fst (~0.90) with JM population based on adaptive SNPs (see below), suggesting severely adaptive differentiation has occurred. Under comprehensive evaluation, we still propose BM1 population as the prior pollen donor for JM population, because (1) it displays moderate genetic differentiation (Fst = 0.51) with JM population based on adaptive SNPs; (2) it has the highest genetic diversity and heterozygosity; and (3) it has the lowest inbreeding level and fewest shared homozygous deleterious mutations with JM population. See more details in page 14-15 and page 9, line 15-25.

Powered by Editorial Manager® and ProduXion Manager® from Aries Systems Corporation

Reviewer #2: In the study "Genomic insights into endangerment and conservation of the garlic-fruit tree (Malania oleifera), a plant species with extremely small populations" the authors examine an at risk species under upcoming climate models and advise on potential strategies to ensure its survival. The authors point out the plant's usefulness to people, nervonic acid, however this is not the main motivation presented for the protection of this species, rather its ecological importance is. This is a viewpoint I advocate for, too much attention can be given to human important species to the exclusion of other study species. The manuscript's introduction effectively introduces the study species, study goals, and motivation for study. All the analysis performed make sense and support the study goals. The presentation of the results was clear and methodical. The discussion was thoughtful and supported by the results. Furthermore, the discussion made clear how the conclusions should be viewed "to provide practical and meaningful suggestions for the conservation actions".
Response: Thanks for the positive assessment of our manuscript. Hopefully these comments are satisfactorily addressed in the revised manuscript.
1. My only criticism of this manuscript is that it lacks details on the Malania oleifera genome and SNP distribution. While referenced, the manuscript doesn't provide the genome size, ploidy level, or assembly status. This makes it difficult to assess the significance of the reported SNP count (43,413,408 in dataset 1). After filtering the SNP set is heavily reduced (as expected due to the low number of samples and small population sizes), again is this a lot of SNPs, what percent of the genome does this represent?
Response: Thanks for your advice. We have added the information of genome size, ploidy level, and assembly status of M. oleifera. See page 3, line 6 and page 4, line 10. Moreover, after strict quality controlling based on 43,413,408 initial variant sites, we got 250,362 SNPs (dataset 2) and showed the distribution of these SNPs across 13 pseudochromosomes of M. oleifera. These SNPs covered most genome regions and can well represent genome-wide variation. Please see the newly added supplementary figure S2 for details.

Reviewer #3: This study employs comprehensive conservation genomic analysis to investigate the endangerment and conservation of Malania oleifera. The manuscript is well-written, and the authors have utilized appropriate techniques and data analyses. Their results will provide a solid foundation for management efforts. However, I have several concerns about this manuscript.
Response: Thanks for the positive assessment of our manuscript. We have considered your suggestions seriously and hope our revised manuscript can fully address your concerns.
1. Page 3 Line 40, "The number of individuals collected per population varied from 5 to 17, and if populations contained fewer than ten individuals, all individuals were sampled." This sentence needs clarification. Are you referring to samples from the germplasm bank?
Response: Sorry for the unclear description. We have clarified the sentence. See page 3, line 39-40.
2. Page 4 Line 15, Page 5 lines 2-4,7, If the dataset was not used in the subsequent analysis, it does not need to be named. Only the datasets used in the following analysis should have a name. Therefore, the authors may consider reducing the naming of datasets.
Response: Thanks for your suggestion. We have deleted the redundant name of dataset across the whole manuscript.
3. Page 4 Line 29, Which BAM files did you use for the diversity analysis? Please clarify.
Response: Sorry for the unclear description. The diversity analysis was performed by ANGSD v. 0.921 which used binary alignment map (BAM) format file of each sample as input. To prepare the input BAM files, we firstly used BWA v. 2.1 to align paired-end clean reads to the genome of M. oleifera. And then used SAMtools v. 1.9 to convert sequence alignment map (SAM) format files to sorted BAM files. Finally, we employed Sambamba v.0.7.1 to mark and remove duplicate reads. We have clarified the sentence. See page 4, line 9-13 and line 29.
4. Page 4 Line 38, The methods you used are both based on Fst, so they are not very distinct.
Response: Revised. See page 4, line 37.
5. Page 5 Line 20, Reconstructing the ancestral sequence is not easy. The Stairway Plot v.2 can now use folded SFS to perform demographic analysis.

Response: Thanks for your advice. In the revised manuscript, we used both folded and unfolded SFS to infer demographic history of M. oleifera by Stairway Plot v.2. The two results showed similar bottleneck events at the corresponding time. See Figure 2a, Figure S10, line 18-22 in page 5 and line 27-33 in page 9.
6. Page 6 Lines 15-20, Selecting variables based solely on the correlation between them seems somewhat arbitrary. It would be more robust to first use analyses such as gradient forest to rank the importance of all the variables. Then, select variables based on the results of variable importance and their correlations. For the environment-associated analysis, you may consider skipping this step, as there are no model overfitting issues.
Response: Thanks for pointing out the shortcoming. As you advised, we ranked the climatic factors based on weighted R2 importance using gradient forest model and selected four most important and uncorrelated factors (Pearson's $|r| < 0.7$), including BIO3, BIO7, BIO14 and BIO15 for BayeScEnv, RDA and GF analysis. See Figure S14 and page 6, line 16-23.
7. Page 8 Lines 23-26, The values of mean heterozygosity rate should be converted into percentages.
Response: Revised. See page 9, line 6-9.
8. Page 10 Lines 15-17, The conclusion that "we found that populations with severe inbreeding also had stronger genetic load, but experienced weaker purifying selection against nonsynonymous mutations" is not very straightforward. You need to provide more explanation. Additionally, the ratio of 0-fold to 4-fold degenerate nucleotide diversity ($\pi 0/\pi 4$) is commonly used as an index of purifying selection efficiency.
Response: We are sorry for the unclear description. Relevant description has been verified. See page 10, line 25-29. We used the ratio of 0-fold/4-fold degenerate sites heterozygosity to intergenic (neutral) heterozygosity as an index of purifying selection efficiency referring to the method of Yang et al (2018) and Liu et al (2022). Significant negative correlation implied that severely deleterious mutations can be effectively purged, which may be the maintaining mechanism of M. oleifera with small population size.
9. Page 10 Lines 32-33, Each outlier detection method has a proportion of false positives (FP). When you combine the outliers from different methods, the FP rate might be even higher. How do you control for this?
Response: Thanks for pointing out the deficiency. To avoid false positives, we assigned the 380 SNPs that detected by both BayeScEnv and RDA as environment-associated SNPs (See page 11, line 1-5). And we re-inferred genomic offset (GO) using R package gradientforest v.0.1-37, also found a strong negative correlation between GO and altitude, with populations at higher altitudes generally having lower GO values (see Figure 4 and Table S13). The result is similar compared to the previous result based on all 1,156 SNPs detected by BayeScEnv and RDA, which highlight the reliability of our methods.
10. One suggestion you may consider: combine the results of niche modeling and genetic offset analysis to infer climate change-driven vulnerability. For more information, see this article (https://www.nature.com/articles/s41467-022-32546-z).
Response: Thanks for your advice. The article you mentioned is a very important reference for us and we have tried to combine the results of niche modeling and genetic offset by calculate genome-niche index (gni). Following the methods of Chen et al (2022), to detect populations that are least interrupted by future climate change, only the niches of increasing future suitability were considered because populations in these areas would not be challenged by niche suitability decline. However, in our case, niches with increasing future suitability were lacking and all sampling sites will have decreased niche fitness (see Figure 5). So, we think this method is not suitable for M. oleifera to combine genomic offset and niche suitability change.

References
Chen Y, Jiang Z, Fan P, et al. The combination of genomic offset and niche modelling provides insights into climate change-driven vulnerability. Nat Commun 2022;13(1):4821. doi:10.1038/s41467-022-32546-z.
Frankham R, Ballou JD, Eldridge MD, et al. Predicting the probability of outbreeding depression. Conserv Biol 2011;25(3):465-75. doi: 10.1111/j.1523-1739.2011.01662.x.
Liu S, Zhang L, Sang Y, et al. Demographic history and natural selection shape patterns of deleterious mutation load and barriers to introgression across populus genome. Mol Biol Evol 2022;39(2):msac008. doi: 10.1093/molbev/msac008.
Severns PM. Precautionary hand pollination suggests outbreeding depression between

	potential seed donor populations for a rare wetland plant. J Torrey Bot Soc 2013;140(1), 20-25. doi: 10.3159/TORREY-D-12-00046.1.<br>Yang Y, Ma T, Wang Z, et al. Genomic effects of population collapse in a critically endangered ironwood tree Ostrya rehderiana. Nat Commun 2018;9(1):5449. doi:10.1038/s41467-018-07913-4.
Additional Information:	
Question	Response
Are you submitting this manuscript to a special series or article collection?	No
**Experimental design and statistics**<br><br>Full details of the experimental design and statistical methods used should be given in the Methods section, as detailed in our Minimum Standards Reporting Checklist. Information essential to interpreting the data presented should be made available in the figure legends.<br><br>Have you included all the information requested in your manuscript?	Yes
**Resources**<br><br>A description of all resources used, including antibodies, cell lines, animals and software tools, with enough information to allow them to be uniquely identified, should be included in the Methods section. Authors are strongly encouraged to cite Research Resource Identifiers (RRIDs) for antibodies, model organisms and tools, where possible.<br><br>Have you included the information requested as detailed in our Minimum Standards Reporting Checklist?	Yes
**Availability of data and materials**<br><br>All datasets and code on which the conclusions of the paper rely must be either included in your submission or deposited in publicly available repositories (where available and ethically appropriate), referencing such data using	Yes

a unique identifier in the references and in the “Availability of Data and Materials” section of your manuscript.<br><br>Have you have met the above requirement as detailed in our Minimum Standards Reporting Checklist?	

Powered by Editorial Manager® and ProduXion Manager® from Aries Systems Corporation

Manuscript
Click here to access/download;Manuscript;Manuscript-Aug 22.docx

Genomic insights into endangerment and conservation of the garlic-fruit tree (*Malania oleifera*), a plant species with extremely small populations

Yuanting Shen[1,2,3,4†], Lidan Tao[1,2,3†], Rengang Zhang[1,2,3†], Gang Yao[1,2], Minjie Zhou[1,3], Weibang Sun[1,2*], Yongpeng Ma[1,2*]

[1]Yunnan Key Laboratory for Integrative Conservation of Plant Species with Extremely Small Populations, Kunming Institute of Botany, Chinese Academy of Sciences, Kunming 650201, China

[2]Key Laboratory for Plant Diversity and Biogeography of East Asia, Kunming Institute of Botany, Chinese Academy of Sciences, Kunming 650201, China

[3]University of Chinese Academy of Sciences, Beijing 100049, China

[4]State Key Laboratory of Plant Diversity and Specialty Crops, Institute of Botany, Chinese Academy of Sciences, Beijing 100093, China.

* Corresponding authors. E-mail address: mayongpeng@mail.kib.ac.cn; wbsun@mail.kib.ac.cn

†These authors contribute equally to this work.

Lidan Tao [0000-0002-1396-0524]; Gang Yao [0000-0002-3628-7088];Rengang Zhang [0000-0002-8028-9208]; Weibang Sun [0009-0009-5246-9226]; Yongpeng Ma [0000-0002-7725-3677]

Abstract

Background: Advanced whole genome sequencing techniques enable covering nearly all genome nucleotide variations, thus can provide deep insights into protecting endangered species. However, the use of genomic data to make conservation strategies is still rare, particularly for endangered plants. Here we performed comprehensive conservation genomic analysis for *Malania oleifera*, an endangered tree species with high amount of nervonic acid. We used whole-genome resequencing data of 165 samples, covering 16 populations across the entire distribution range to investigate the formation reasons of its extremely small population sizes and to evaluate the possible genomic offsets and changes of ecology niche suitability under future climate change.

Results: Although *M. oleifera* maintains relatively high genetic diversity among endangered woody plants (θ_π = 3.87×10^{-3}), high levels of inbreeding have been observed, which has reduced genetic diversity in three populations (JM, NP and BM2) and caused the accumulation of deleterious mutations. Repeated bottleneck events, recent inbreeding (~490 years ago) and anthropogenic disturbance to wild habitats have aggravated the fragmentation and endangered status of *M. oleifera*. Due to the significant effect of higher average annual temperature, populations distributed in low altitude exhibit greater genomic offset. Furthermore, ecological niche modelling shows the suitable habitats for *M. oleifera* will decrease by 71.15% and 98.79% in 2100 under scenarios SSP126 and SSP585, respectively.

Conclusions: The basic realizations concerning the threats to *M. oleifera* provide scientific foundation for defining management and adaptive units, and prioritizing populations for genetic rescue. Meanwhile, we highlight the importance of integrating genomic offset and ecological niche modeling to make targeted conservation actions under future climate change. Overall, our study provides a paradigm for genomics-directed conservation.

Keywords

Recent inbreeding; Deleterious mutation; Demographic history; Genomic offset; Ecological niche modelling; Conservation genomics

Introduction

Historical climate disturbances and frequent human activity have caused many species that were once widespread with continuous distributions to become small, fragmented populations [1]. High levels of inbreeding continually occur in these populations, leading to the accumulation of deleterious mutations and low species adaptability, ultimately increasing the risk of extinction [2, 3]. Genome contains evolutionary footprints which can be used to estimate inbreeding levels of species even without detailed pedigrees [4, 5]. For example, runs of homozygosity (ROH), genome regions with a certain length that is identical by descent, has been widely used as an indicator of inbreeding [6, 7]. The long ROH indicate a closer relationship to the most recent common ancestor, implying a higher level of inbreeding. For small and isolated populations with high inbreeding levels, genetic rescue is necessary to introduce beneficial mutations by establishing gene flow between populations [8]. However, cautions should be taken when making decisions regarding genetic rescue, and comprehensive exploration of genetic background of these small populations must be done in advance [9, 10].

Rapid climate change in the future is a widely recognized threat to global biodiversity [11-13]. The threatened degree of species depends on how they respond to climate change. There are two main mechanisms: migrating to new habitats or adapting

to the changing environments through phenotypic plasticity or *de novo* mutations [14-16]. However, in the case of long-lived forest trees, individual organisms are nearly incapable of migrating to keep pace with changing climate and may experience maladaptation throughout their lifetimes [17-19]. Thus, if species' responses to future climate change can be predicted, their extinction risks can be estimated and targeted conservation guidelines and management strategies can be developed in advance.

Malania oleifera Chun & S. K. Lee (NCBI:txid397392), the single species in the genus *Malania* (Olacaceae), is an endemic, semi-parasitic evergreen tree (diploid) that naturally scattered in the west Guangxi (a.s.l 300~1000 m) and southeast Yunnan province, China (a.s.l 300~1640 m) [20]. It adapts well to rocky desert habitats and can be used as an afforestation tree in karst landscapes [21]. Moreover, it has extremely high economic and medicinal value due to the large amount of lipids in its seed. The main lipid component is nervonic acid (cis-tetracos-15-enoic acid, >60%), which is essential for human nervous health [22]. However, mainly due to overexploitation, wild resources of *M. oleifera* have decreased by approximately 25,000 individuals between 2000 and 2017 solely in Guangnan County (Yunnan, China) [23, 24]. Additionally, physiological factors of *M. oleifera*, including large seed size, short seed lifespan, difficulty in natural seed germination, low rate of pollen germination and susceptibility to root rot, have made natural propagation and regeneration difficult [25-28]. Therefore, *M. oleifera* has been categorized as Vulnerable (VU) on the IUCN Red List (Sun, 1998) and recorded in the Class II Key Protected Wild Plant List in China [29], and it has also been listed as a plant species with extremely small population size in China [30], highlighting the urgent need for its conservation.

Previous studies related to *M. oleifera* mainly focused on the biosynthetic pathway of nervonic acid [22, 31] and exploring the optimal condition of growing artificial seedlings for better utilization [32, 33]. However, the conservation process of *M. oleifera* is limited to in situ protection of existing wild resources [34]. Recent advancements in whole genome sequencing techniques enable covering nearly all the nucleotide variations of a genome and can provide deep insights into protecting endangered species [35]. However, there is a gap in using genomic data to guide conservation strategies, particularly for plants. Therefore, we utilize *M. oleifera* as a study case to investigate the above issues. We aimed to provide a comprehensive framework for the *M. oleifera* conservation through the perspective of conservation genomics.

Materials and methods

Sample collection and whole genome resequencing

A total of 165 leaf samples were collected from 16 wild populations across the entire distribution of *M. oleifera* from Yunnan and Guangxi provinces, China (Figure 1a; Table S1). Among them, 76 samples were collected based on our field investigation, and the remaining 89 samples were obtained from the Germplasm Bank of Wild Species

in Southwest China. The number of individuals collected per population (including the samples from germplasm bank) varied from 5 to 17, and if populations contained fewer than ten individuals, all individuals were sampled.

Genomic DNA was extracted from silica-dried leaf tissues using a modified CTAB method [36] and the concentration and quality of the DNA was determined using a NanoDrop2000 Spectrophotometer (Thermo Fisher Scientific). Samples were sent to Beijing Ori-Gene Science and Technology Co., Ltd for Illumina sequencing library preparation according to the manufacturer's specifications. Paired-end raw reads (150bp) were generated on the Illumina HiSeq platform.

Read mapping and SNP calling

The raw data were filtered using Fastp (RRID:SCR_016962) v. 0.19.3 [37]. Paired-end clean reads were mapped to chromosome-level genome of *M. oleifera* (~1.5 Gb) [31] using BWA-MEM (RRID:SCR_022192) v. 2.1 [38]. SAMtools (RRID:SCR_002105) v. 1.9 [39] was used to convert sequence alignment map (SAM) format files to sorted binary alignment map (BAM) format files. Sambamba (RRID:SCR_024328) v.0.7.1 [40] was used to mark and remove duplicate reads. Freebayes (RRID:SCR_010761) v. 1.3.6 [41] was employed to call variants, and only bases with a quality score ≥20 and reads with a mapping quality score ≥30 were included. This produced total of 43,413,408 initial variant sites. We then employed VCFtools (RRID:SCR_001235) v. 0.1.15 [42] to filter sites with the following criteria: (1) sites with coverage depth below 1/2*average site coverage and above 2*average site coverage were discarded after investigating the coverage distribution; (2) sites located on the organelle genomes, or contigs that were not anchored on the chromosomes were excluded; (3) SNPs with depth below 3× or genotype quality score < 20 were redefined as missing; (4) only bi-allelic SNPs were reserved; (5) SNPs with a missing rate > 20% were removed, leaving 2,144,506 SNPs (dataset 1); and (6) SNPs with a minor allele frequency < 0.05 were all excluded. Finally, it remained 250,362 SNPs (dataset 2), which distributed on 13 pseudochromosomes (Figure S2).

Population genetic diversity and runs of homozygosity

Based on dataset 2, we detected genome-wide linkage disequilibrium (LD) decay among 16 populations using PopLDdecay (RRID:SCR_022509) v. 3.4.0 [43]. Nucleotide diversity (θ_π), Watterson's θ (θ_W) and heterozygosity rate were calculated using ANGSD (RRID:SCR_021865) v. 0.921 [44] based on bam files which removed duplicates (see above). In addition, we calculated the values of the three parameters in more specific genomic regions (intergenic, CDS, intron, fold-0, and fold-4). To examine inbreeding depression, we detected runs of homozygosity (ROH) using vcftools v. 0.1.15 [42] based on dataset 2 with the key parameters "--LROH" and only ROH longer than 100 kb were kept. Moreover, we calculated the frequency of runs of homozygosity (FROH), which is equal to the sum of all ROH lengths longer than 100 kb divided by genome effective length [45].

Inference of population structure based on all loci, adaptive loci and neutral loci

We employed two software to detect outlier SNPs potentially related to adaptive evolution. Firstly, we used the sparse non-negative matrix factorization (snmf) function applied in the R package LEA (RRID:SCR_009090) v. 3.1.4 [46] to estimate the most likely number of ancestral populations based on dataset 2. To reduce the number of false positives, we reserved SNPs with the false discovery rate (FDR) less than 0.01. Secondly, we applied a principal component analysis (PCA) method using R package Pcadapt (RRID:SCR_022019) v. 4.3.3 [47] with a 0.01 cutoff of FDR to identify SNPs which highly influenced the formation of observed differentiation. SNPs detected by both methods were considered as potential adaptive SNPs, otherwise, they were considered as neutral SNPs. Finally, we employed PLINK (RRID:SCR_001757) v. 1.90b4.1 [48] to filter out linkage disequilibrium sites and ultimately obtained 33,971 (dataset 3, all loci), 1,515 (dataset 4, adaptive loci) and 32,930 (dataset 5, neutral loci) SNPs for downstream analysis (Figure S1).

Based on the three datasets described above, we employed ADMIXTURE (RRID:SCR_001263) v. 1.3.0 [49] to infer the population structure. The most likely population number of K was determined by the minimizing cross-validation error. Principal component analysis (PCA) was conducted in GCTA v1.94.1 [50] and MEGA (RRID:SCR_000667) v. 7.0 [51] was used to construct NJ trees. Pairwise fixation statistics (Fst) among the 16 populations were calculated using vcftools v. 0.1.15 [42].

Estimation of demographic history

We employed Stairway Plot v.2 [52] and MSMC (RRID:SCR_023677) v.2 [53] to infer population demographic history of *M. oleifera*. For the analysis of Stairway Plot, we firstly performed ancestral sequence reconstruction to infer ancestral status (see details in Note S1 and Table S16). To mitigate the effects of selection, we excluded the upstream and downstream 5kb regions of genes to infer folded site frequency spectrum (SFS) and unfolded SFS using ANGSD v. 0.921 [44]. We set the average generation time of *M. oleifera* as ten years because it takes about ten years for a seed to grow into a seed-producing plant according to our field observations. The mutation rate was set to 2.5×10^{-8} per site per generation (see details in Note S2 and Table S17). MSMC is a Multiple Sequentially Markovian Coalescent approach which uses the density of heterozygous sites to estimate the effective population size (*Ne*) through time. Different individual numbers or haplotypes provide distinct resolutions for the analysis of demographic histories. Therefore, we employed MSMC to separately estimate the coalescence rate within two, four and eight haplotypes, referring to the simulation results of Schiffels & Durbin [53]. A total of 100 random combinations of individuals were used for the three haplotype analyses to estimate the medians and 95% CI values. The average generation time and mutation rate values were set to the same as in Stairway Plot.

Detection of deleterious mutations

Deleterious mutations in *M. oleifera* were predicted using the Sorting Intolerant From Tolerant (SIFT) algorithm [54]. We used a modified approach to perform the SIFT prediction (see Note S3). The TrEMBL plant database [55] was used to search for orthologous genes and the SIFT scores were calculated based on the degree of conservation among loci. Based on dataset 1 (included low frequency variants), SNPs in the coding regions were categorized as deleterious (SIFT score <0.05), tolerated (SIFT score ≥0.05) or synonymous using SIFT4G (RRID:SCR_021850) [56]. The low confidence sites and "NA" sites were not considered.

To provide accurate and direct genetic rescue guidance for *M. oleifera* populations with high genetic load, we selected five populations, including one as the potentially threatened population and four as the candidate pollen-donors. We drew a Venn diagram to explore the distribution of shared or unique homozygous deleterious mutations among the five populations. The four-candidate pollen-donors were characterized by (1) having low genetic load, low levels of inbreeding, high genetic diversity, and high heterozygosity; or (2) low genetic differentiation with the rescued population; or (3) sharing the same genetic lineage as the rescued population based on adaptive loci.

Identification of environment-associated adaptive variants

The environmental data included 19 bioclimatic variables at 2.5-minute resolution (5 km) were downloaded from WorldClim v.2.1 database (RRID:SCR_010244) (Table S9). Each environmental factor is extracted through the coordinates of sampling points. We selected the top climatic factors based on weighted R^2 importance (Figure S14) using a machine-learning gradient forest (GF) model in the R package gradientforest v.0.1-37 [57] by modeling the relationship of climatic variables and SNPs with 500 regression trees. To avoid multicollinearity, we kept variables with |correlation coefficient| < 0.7 by calculating Pearson's correlation coefficient in the R package corrplot v.0.92 (RRID:SCR_024683) [58] and ultimately retained four most important and uncorrelated factors, including BIO3 (Isothermality), BIO7 (Temperature Annual Range), BIO14 (Precipitation of the Driest Month) and BIO15 (Precipitation Seasonality). Next， we used BayeScEnv [59] and Redundancy Analysis (RDA) [60] to identify environment-associated SNPs. BayeScEnv represents a univariate genotype-environment association approach. For BayeScEnv method, the input files included environmental factor standardized by the mean variance and contained codominant data which converted by PGDSpider v. 2.1.1.5 [61] based on dataset 2. RDA is a multivariate linear regression-based method [62]. We ran RDA analysis in the R package vegan v.2.5-7 [63], using function "anova.cca" to check the significance of RDA model and function "outliers" to identify local-adaptation-associated SNPs that load in the tails of ±3 standard deviation cut-off (two tailed P-value = 0.0027). To recognize the gene functions of the candidate SNPs obtained from BayeScEnv and RDA, we employed a Gene Ontology enrichment analysis using the eggNOG-mapper (RRID:SCR_021165)

v.2 [64].

Genomic offset modeling with gradientforest

We used the gradient forest (GF) model in the R package gradientforest v.0.1-37 [57] to predict genomic vulnerability to future climate change. The environmental-associated SNPs detected by both BayeScEnv and RDA were denoted as candidate SNPs dataset. And 500 randomly selected SNPs based on dataset 2 were recorded as reference SNPs dataset to match the magnitude of candidate SNPs dataset. The SNPs data with MAF > 10% were converted into minor allele frequencies (MAF) per population. To ameliorate the linkage effect, we only kept one SNP per 100,000 bp range and finally obtained 326 reference SNPs and 213 candidate SNPs. We employed the GF model with 500 regression trees per SNP to build a function for the four most important environmental factors (BIO3, BIO7, BIO14, BIO15). Genomic offset (GO) was defined by Euclidean distance between current (1970-2000) and future (2081-2100) climate which used the current condition as baseline [16]. To explore possible future climate conditions and predict GO for *M. oleifera*, we employed three widely used global climate models (BCC-CSM2-MR, CNRM-CM6-1 and CNRM-ESM2-1) and two emission scenarios (SSP126 and SSP585) which represent the mild and extreme future carbon emissions. The predicted GO results of three global climate models for each grid were averaged with assigned weights.

Ecological niche modelling

The distribution records of *M. oleifera* were collected from online databases, published academic articles [65, 66] and field investigation, and all records were manually verified using online map. To reduce sampling bias, we only kept one record within 5km using the rarefy function of the R package Humboldt [67], remaining a total of 87 records (Table S10). The 19 bioclimatic variables (Table S9) were also used in ecological niche modelling. Since background or pseudo-absence data of ecological niche models were sampled from the entire modeling map, we recalculated the correlation coefficients of 19 bioclimatic variables based on the whole distribution area. We removed autocorrelated variables (Pearson's r > 0.7 and variance inflation factor > 10) using the R package usdm v.2.1 [68] and kept six uncorrelated variables (BIO1, BIO2, BIO7, BIO12, BIO14 and BIO18) for ecological niche modelling.

The ecological niche model (ENM) was built using an ensemble modeling method that combined outputs of five single models with high performance: GAM (Generalized Additive Model by the R package mgcv v.1.9) [69], MaxEnt (tuned MaxEnt model by the R package dismo v.1.3) [70], RF (Random Forest with down-sampling by the R package randomForest v.4.7) [71], Lasso (by the R package glmnet v.4.1) [72], and BRT (Boosted Regression Trees by the R package dismo) [73]. Each model was run for ten replicates, with pseudo-absence data of 10,000 points randomly generated using the R package Biomod2 [74] for three replicates, resulting in a total of 5*10*3=150 single models. However, only models with positive Somer's D values were employed to create

the final ensemble prediction, which was weighted by the TSS value of each model. The evaluation of single model and ensembled model was performed by the R packages Ecospat v.4.0.0 [75] and prg v.0.5.1 [76].

The niche suitability of *M. oleifera* under future (2081-2100) carbon emission scenarios (SSP126 and SSP585) was predicted using the same climate models (BCC-CSM2-MR, CNRM-CM6-1 and CNRM-ESM2-1) as GO analysis. We used R package PresenceAbsence v. 1.1.11 [77] to calculate the threshold of ecological niche suitability and grids with values higher than the threshold were defined as suitable habitats. Furthermore, referring to the method of Chen et al. [16], we defined NSC as niche suitability change between current and future climate, which is equal to the niche suitability index in the current climate minus the niche suitability index in the future climate. A positive value implies that niche suitability will decrease in the future compared to the present condition, while a negative value means increasing niche suitability. The NSC results of single model for each emission scenario were averaged.

Results

Population structure and phylogeny of *M. oleifera*

Whole genome resequencing generated an average of ~4.71 Gb raw data and 65,007,259 paired-end reads for each sample, and the average sequencing depth was 6.5-fold. After filtering, the average Q20 and Q30 rates of paired-end reads were 97.51% and 92.68%, respectively, with an average mapping rate of 99.39% (Table S2 and S3). Neutral and adaptive genomic variations have inconsistent evolutionary patterns [78] and provide different types of information when determining optimal conservation measures [79]. To disentangle these discrepancies, we used three SNP datasets, including all loci (dataset 3), adaptive loci (dataset 4) and neutral loci (dataset 5), to decipher the genetic relationships within *M. oleifera* by constructing population structure, PCA and phylogenetic trees.

The ADMIXTURE analysis results from all loci and neutral loci both revealed the optimal number of clusters (K) was 14 (Figure S7). Samples in most populations were relatively pure with no or only mild genetic mixture with other populations, except for SG, ML, FS and LY2 populations (Figure S8a and S9a). However, ADMIXTURE analysis based on adaptive loci indicated that K = 10 was optimal (Figure S7) with ML-BB2, BB1-BM2 and ZL-LY1 paired populations have the same genetic composition, implying the paired population have similar adaptability, respectively (Figure 1b). Notably, DX and GL populations were found to be 100% pure based on ADMIXTURE analysis of all datasets. Measures of PCA based on all datasets revealed clear separation of DX population from other populations by PC1 and PC2, which explained 29.6%, 68.4% and 25.2% of the genome covariance based on the results of all loci, neutral loci, and adaptive loci, respectively (Figure 1d, S8b and S9b). The NJ trees based on all datasets were consistent with the corresponding ADMIXTURE analysis, showing populations with similar genetic components had closer phylogenetic relationships

(Figure 1c, S8c and S9c).

Genetic diversity, heterozygosity, and genetic differentiation

The average whole genomic genetic diversity of *M. oleifera* was $3.87\times10^{-3} \pm 1.34\times10^{-3}$ for pairwise nucleotide differences (θ_π) and $3.46\times10^{-3} \pm 1.23\times10^{-3}$ for Watterson's θ (θ_W) (Table 1 and S4). BM1 population showed the highest θ_π and θ_W compared with other populations, while NP, JM and BM2 populations had lower θ_π and θ_W (Figure S4) and showed a more obvious sawtooth-like distribution pattern of genetic diversity across the genome (Figure S5). When we divided the genome into five specific genomic regions, the values of θ_π and θ_W showed the trend of intergenic > fold-4 > intron > CDS > fold-0, which was highly consistent among all 16 populations (Table 1 and S4). The mean heterozygosity rate in *M. oleifera* was 0.50% ± 0.14%, and it varied among populations, with BM1 (0.56% ± 0.16%) population showing the highest values, and with the lowest heterozygosity rate seen in JM (0.30% ± 0.04%), NP (0.31% ± 0.04%) and BM2 (0.35% ± 0.02%) populations (Table S5). As expected, the results of heterozygosity rate across more specific genomic regions showed the same trend as the genetic diversity (Figure S6). Moreover, the genome-wide LD decay analysis revealed that the level of LD varied greatly between populations, with BM2 population showing the slowest decay of LD, whereas BM1 population had the fastest LD decay (Figure S3).

The values of pairwise Fst based on adaptive loci (dataset 4) were significantly higher than those of Fst based on all loci (dataset 3) and neutral loci (dataset 5), showing that adaptive loci > all loci > neutral loci in all paired populations (Table S6). This was particularly prominent between DX population and other populations, with a significant high pairwise Fst ranging from 0.87 to 0.91 based on adaptive loci, compared to 0.26-0.46 and 0.20-0.41 based on all loci and neutral loci, respectively (Table S6). Moreover, BM2, JM and NP populations, which have lowest genetic diversity, showed high genetic differentiation from other populations (average pairwise Fst = 0.54 based on adaptive loci). In contrast, BM1 population with highest genetic diversity, showed relatively low genetic differentiation from other populations (average pairwise Fst = 0.45 based on adaptive loci) (Table S6).

Demographic history of *M. oleifera*

Stairway plot detected two severe population declines of *M. oleifera* based on unfolded SFS. The first occurred around 0.5-0.22 Ma, corresponding to the Middle Pleistocene with climate upheaval and the *Ne* was reduced to ~8230 (Figure 2a). Subsequently, all the populations quickly recovered to $\sim2.4\times10^5$ and remained stable until a recent bottleneck at around 10 Ka during the last glacial maximum (LGM), where there was a sharp population contraction to its lowest level (~1676). The result based on folded SFS also showed two bottleneck events at the corresponding time (Figure S10). MSMC tracked the more recent demographic trajectory of *M. oleifera*, especially within the last 10,000 years (Figure 2b). Based on the analyses results of two, four and eight

haplotypes, the *Ne* of *M. oleifera* experienced a significant decline over time, reaching a nadir (below 75) around 400-500 years ago, followed by a slight population expansion. It is worth mentioning that both programs detected a population decline in *M. oleifera* during the LGM, which strengthened the reliability of the results.

Characterization of runs of homozygosity and deleterious mutations

We investigated whether *M. oleifera* showed signs of recent inbreeding by calculating the runs of homozygosity (ROH). Referring to the method of Robinson et al [10], we used the physical length of ROH to estimate the number of generations to the common ancestor (g) as $g = 100/(2{*}L)$, where L is the mean length of ROH in megabases (Mb). Here, the L of all 16 populations of *M. oleifera* ranged from 0.46 Mb (BM1) to 1.03 Mb (JM) (Figure S11). Our results indicated that inbreeding occurred about 49 to 112 generations ago. Specially, the effects of inbreeding varied greatly among populations (Figure 3a). We found the frequency of runs of homozygosity (FROH) was significantly higher in JM (45.37%-70.95%) population than in other populations, whereas it was lower in LY2 (3.52%-9.82%), BM1 (4.31%-13.92%), FS (7.11%-13.39%) and DX (8.51%-15.60%) populations. Moreover, populations with severe inbreeding would be predicted to have larger numbers of long ROH (> 1 Mb) than short ROH (100 Kb - 1 Mb) (Figure 3b). Specifically, JM population harbored maximum number of ROH > 1 Mb, which represented 37.07% of the total genome. In contrast, BM1 population had minimum number of long ROH, with only 1.20% of ROH being longer than 1 Mb (Table S7).

Based on the modified SIFT prediction approach, we detected a total of 2,404 deleterious mutations, 5,040 tolerated mutations and 6,172 synonymous mutations (Table S8 and Figure S13a). Particularly, the frequency of deleterious mutations of homozygous-derived alleles reflects genetic load and adaptability of species, and it varies greatly among populations even within the same species [80]. Our results showed that the number of homozygous deleterious sites to the total deleterious mutations in JM population was significantly higher than in most other populations except NP and SG, suggesting that JM population had higher genetic load (Figure 3c). Interestingly, the ratio of 0-fold to 4-fold degenerate sites heterozygosity showed a significant negative correlation with intergenic (neutral) heterozygosity (Figure 3d). It implied that more severely deleterious mutations can be effectively purged by purifying selection, which may be the maintaining mechanism of *M. oleifera* with small population size [45]. To provide accurate and direct genetic rescue guidance for JM population, we selected four populations as potential pollen-donors to construct a Venn diagram of homozygous deleterious variants (Figure S12a). Our results showed that the most homozygous deleterious variants were shared among all the five populations and JM population had least shared homozygous deleterious variants with BM1 population (172 variants).

Signals of genomic offset to future climate change

Potential genomic variants related to climate adaptation were detected using BayeScEnv and Redundancy Analysis (RDA). For BayeScEnv analysis, with a q-value cut-off of 0.05, we identified 589 SNPs related to climate adaptation. Of these, 491 SNPs were associated with BIO7 and BIO14, respectively, followed by BIO3 (471 SNPs) and BIO15 (168 SNPs) (Table S11). For RDA, 694 SNPs were detected along five significant RDA axes, of which 459 SNPs were correlated most to BIO14, 156 SNPs to BIO3, 40 SNPs to BIO7 and 39 SNPs to BIO15. To avoid false positives, we assigned the 380 SNPs that detected by both BayeScEnv and RDA as environment-associated SNPs (Table S11). To figure out the potential function of genomic variants associated with climate adaptation, we conducted a functional annotation of outlier SNPs. Gene Ontology enrichment analysis assigned a total of 258 Gene Ontology categories ($p < 0.05$), of which 158 categories belonged to biological processes and abundant genes were associated with metabolism, transmembrane transport, methylation, cell development, flowering, and telomere maintenance (Table S12).

To assess which population of *M. oleifera* will be most likely disrupted in the future (2081-2100) under two greenhouse gas scenarios (SSP126 and SSP585), we employed gradient forest (GF) method to investigate the genomic offset (GO) using integrated results of BCC-CSM2-MR, CNRM-CM6-1 and CNRM-ESM2-1 climate models. The GO is measured by the Euclidean distance of future climate condition compared to current climate status. Higher GO means greater allele frequency changes are required to adapt to the changing climate [81]. GF modeling showed that the degree of GO of all populations increased under scenario SSP585 compared to scenario SSP126, suggesting that extreme future climate change will cause severe genomic vulnerability to *M. oleifera* (Figure 4). Compared with all SNPs (reference), we found that adaptive SNPs (candidate) exhibited higher GO under the same scenarios, implying that adaptive variants were more sensitive to climate change (Figure 4). In addition, we found a strong negative correlation between GO and altitude, with populations at higher altitudes generally having lower GO values (Figure 4).

Ecological niche modelling predicted niche suitability change

We integrated results from five models to perform ecological niche modelling for *M. oleifera* (Table S13). The Area Under the Curve (AUC) value, Somer's D value, True Skill Statistic (TSS) value, Boyce value and the area under the precision-recall gain curve (AUCprg) were about 0.99, 0.99, 0.98, 0.74 and 0.97, respectively, indicating high performance of the ecological niche models (Table S14). Compared to the current state, the potential suitable region in 2100 will reduce by 71.15% and 98.79% under scenarios SSP126 and SSP585, respectively, with the threshold of ecological niche suitability equal to 0.56 (Figure S15). Further, we calculated niche suitability change (NSC) between current and future climate for each grid using the equation: NSC = niche suitability index in the current climate − niche suitability index in the future climate. A

positive value indicates that niche suitability will be decreased under future climate change. Higher positive value means more severe degree of unsuitability. Our results showed slightly higher NSC in the southern part of the distribution range under scenario SSP126 (Figure 5a). However, under scenario SSP585, the NSC increased to a much higher extent in the northern part of the distribution range (Figure 5b), which is the Karst basin harboring Nanpan river, Beipan river and Tuoniang river. This suggest that drastic climate change will exacerbate ecological vulnerability in karst landforms by affecting hydrological processes [82, 83].

Discussion

The genome harbors valuable evolutionary information of a species and provides deep insights into genetic diversity and evolutionary dynamics, however, the full implementation of conservation genomics in practice is still limited [84]. In this study, we conducted a conservation genomics study on *M. oleifera* based on population-wide genome resequencing data, including 165 individuals. We aim to distinguish the potential factors that affect genetic diversity of *M. oleifera* and to reveal the causes for the formation of its extremely small population patterns, and to assess its adaptability under future climate change. It is our hope that based on the comprehensive results of conservation genomics, it will be possible to provide practical and meaningful suggestions for the conservation actions of this ecologically and economically important species.

Recent inbreeding affected genetic diversity

M. oleifera has relatively high genetic diversity among endangered woody plants (Table S15). This is confirmed by population structure result, which shows K = 14 is optimal based on all loci (Figure S8a), suggesting *M. oleifera* has complex ancestral components despite it occupies a narrow distribution. It seems optimistic, however, genetic diversity cannot determine the endangered status of a species even though it is an important criterion for species conservation [85]. Genetic diversity is affected by many factors such as inbreeding, gene flow, life form, distribution, and rarity [86]. The key to perform conservation actions for endangered species is to realize the pivotal factor affecting genetic diversity. Our results showed that populations with low genetic diversity (JM, NP and BM2) have severe degree of recent inbreeding and displayed more pronounced sawtooth-like distribution patterns of nucleotide diversity across the genome due to long ROH (Figure 3a, S4 and S5). Furthermore, high levels of inbreeding in JM, NP and BM2 populations have led to the accumulation of deleterious mutations (Figure 3c) and they showed greater differentiation from other populations (Table S6), which may result in a vicious circle of inbreeding depression if without intervention [87].

Causes for the formation of small and isolated population

Historical climate disturbances were one of the reasons for the formation of currently

observed small and isolated *M. oleifera* populations. Based on the estimations of Stairway Plot v.2 and MSMC v.2, we observed a bottleneck event of *M. oleifera* during the LGM, resulting in a swift decline in *Ne* (Figure 2). Although the Stairway Plot suggested the *Ne* recovered to its historical peak at the end of the LGM (Figure 2a), this inference is deemed unreliable of very recent demographic events based on site frequency spectrum (SFS) [88]. In contrast, the MSMC results suggested that the *Ne* of *M. oleifera* underwent a protracted decline after the LGM, with a slight recovery occurring approximately 500 years ago (Figure 2b). Moreover, we utilized the mean length of ROH as a metric to estimate the generations of inbreeding, referring to the method of Robinson et al. [10]. Our findings revealed that JM population had experienced the most recent inbreeding, approximately 490 years ago (Figure S11). Intriguingly, the demographic history inferred by MSMC showed that the *Ne* of *M. oleifera* reached its nadir (below 75) about 400-500 years ago (Figure 2b), which may have contributed to the extensive inbreeding. At the same time, human overexploitation and destruction of wild resources have exerted unbearable demographic pressures and resulted in further population fragmentation, as it is hard to find wild individuals again according to a large number of previous distribution records like Mashan, Pingguo, Tiandong, Tianyang, Youjiang, Longzhou counties in Guangxi province [34]. Overall, the combined effect of historical bottleneck events, recent inbreeding, and excessive human disturbance may have led to the formation of small and isolated populations of *M. oleifera.*

Local adaptation-related alleles lead to climate change-driven genomic vulnerability

The climate is currently shifting, and many species face the challenge of keeping pace with ongoing climate changes [89, 90]. Therefore, supporting species adapt to the variable climate is a key but tough task to future conservation action. For *M. oleifera*, we detected a total of 380 SNPs that are related to climate adaptation. And the associated genes are significantly enriched in key processes of metabolism, transmembrane transport, flower development, and telomere maintenance (Table S12). Moreover, each climate factor is associated with dozens to hundreds of SNPs accordingly (Table S11). This is consistent with the polygenic effects underlying local adaptability, meaning that organisms can adapt to rapid climate change through small polygenic allele frequency shifts [18, 91].

Inequivalent response to climate change exists within populations of the same species, due to local adaptation to heterogeneous environments [92]. Analyzing local adaptation pattern can help us better understand how species respond to future climate change. Here, we used the Euclidean distance between future and current climate environments to measure GO. By incorporating intraspecific variations into the predictive GF model, our results showed *M. oleifera* populations distributed in the low elevation exhibit higher GO under both future scenarios (Figure 4). And elevation showed strong negative correlation with annual mean temperature ($R = -0.91$, $P < 2.2\times10^{-16}$) across

the distribution range of *M. oleifera* (Figure S16). This suggests populations in low altitude have a more significant adaptive lag in response to rapid climate change (especially temperature), indicating a greater risk of local extinction if appropriate conservation measures are not taken.

Ecological niche modelling provides insights into ex situ conservation

Ecological niche modelling can predict potential current distribution ranges and suitable habitats under future climate change by linking observed species distribution and abundance to selected environmental variables [93]. Our results showed the suitable habitats for *M. oleifera* will be decreased and ecological niche suitability will be further reduced in the future (Figure S15). However, the degree of niche suitability change is varied under different climate scenarios. The extreme climate (SSP585) is likely to have direct impact on hydrological processes in karst landforms, making the northern part across the distribution range which harbors Nanpan river, Beipan river and Tuoniang river becomes most unsuitable for living (Figure 5b). BM2, SG, ZL and LY1 populations that located in the area deserve highest priority for ex situ conservation when future climate becomes extremely severe. Ecological niche modelling reveals niche suitability change, while GO provides information about genomic inadaptation to future climate change [16]. The two methods provide disparate views to estimate climate-driven vulnerability. It is necessary to combine the methods of ecological niche modelling and genomic offset to make conservation decisions.

Implications for conservation guidelines and management strategies

Currently, a wide range of field investigation and in situ protection of existing *M. oleifera* resources have been implemented [94, 95]. For example, in 2017, Guangnan County labeled 7,941 wild individuals and recorded their growth state [34]. But these measures only have limited impact on guiding future conservation actions. The conservation guidelines and management strategies should be made under demarcating reasonable management units (MUs) and adaptive units (AUs) [96]. Based on the result of population structure and phylogenetic tree (neutral loci), we suggest delineating 14 MUs of *M. oleifera* with most single population being separate MU (Figure S9). Maintaining multiple MUs ensures long-term persistence of the species. Based on adaptive loci, we identified 10 AUs, including JM, SG-NP, ML-BB2, BB1-BM2, GL, ZS, ZL-LY1, LY2-FS, BM1 and DX AUs (Figure 1b). Different AUs represent varied evolutionary potential. Understanding the patterns of adaptive differentiation is crucial when considering conservation priorities, assisted gene flow, migration, and supplementation [79].

For populations with recent inbreeding, genetic rescue is necessary through assisted gene flow [97]. JM population has the lowest genetic diversity and the highest inbreeding and genetic load (Figure 3 and S4), thus it needs urgent genetic rescue. In some cases, high Fst combination among parents generated offspring with high heterozygosity, which may produce heterozygous advantage, however, in most cases,

outbreeding depression occurred [98]. It may occur between interpopulation crossing even in the same species [99]. So, to minimize the risk of outbreeding depression, we do not consider DX population as a potential pollen donor because it has highest Fst (0.90) with JM population based on adaptive SNPs (Table S6), suggesting severely adaptive differentiation has occurred. Under comprehensive evaluation, we propose BM1 population as the prior pollen donor for JM population, because (1) it displays moderate genetic differentiation (Fst = 0.51) with JM population based on adaptive SNPs (Table S6); (2) it has the highest genetic diversity and heterozygosity (Figure S4 and S6); and (3) it has the lowest inbreeding level and fewest shared homozygous deleterious mutations with JM population (Figure S12a), positively reducing the impact of genetic load on hybrid offspring. Moreover, previous research has shown that *M. oleifera* seeds have low germination rates under natural conditions [24], so it is better to keep the seeds for artificial germination after implementing assisted gene flow measures and introduce robust seedlings back to the natural population later.

GO analysis predict that populations located at lower altitudes require a greater change in adaptive allele frequencies to adapt to extreme climates (Figure 4). Low altitude areas are more susceptible to extreme temperature than higher elevations (Figure S16). Therefore, we suggest cultivating heat-resistant individuals and screening pre-adapted genotype under controlled conditions in the laboratory, and then regression experiments can be conducted. Future work should prioritize the conservation of *M. oleifera* because its lasting existence is a prerequisite to exploit resources.

Data Availability

Raw resequencing data are available at the NCBI Sequence Read Archive under BioProject PRJNA978997. All additional supporting data are available in the *GigaScience* repository, GigaDB [100].

Additional Files

Supplementary note S1. Ancestral sequence reconstruction.

Supplementary note S2. Estimation of mutation rate.

Supplementary note S3. Detection of deleterious mutations based on REF-ALT strategy.

Supplementary Fig. S1. Resequencing data processing workflow of *Malania oleifera*.

Supplementary Fig. S2. The distribution of SNPs (dataset 2) across 13 pseudochromosomes under 1 Mb windows.

Supplementary Fig. S3. Genome-wide linkage disequilibrium (LD) decay of *Malania oleifera*. (a) Considering 16 populations separately and (b) as a whole.

Supplementary Fig. S4. The comparison of mean θ_π and θ_W among 16 populations of

Malania oleifera in whole genome (a), intergenic (b), CDS (c), intron (d), fold-0 (e) and fold-4 (f) regions.

Supplementary Fig. S5. Distributions of nucleotide diversity (θπ) across the genome with (a)(b)(c) representing NP, JM and BM2 populations (lowest average θπ), respectively and (d) representing BM1 population (highest average θπ).

Supplementary Fig. S6. The comparison of heterozygosity rate among 16 populations of *Malania oleifera* in whole genome (a), intergenic (b), CDS (c), intron (d), fold-0 (e) and fold-4 (f) regions.

Supplementary Fig. S7. Cross-validation error curve based on all loci (a), neutral loci (b) and adaptive loci (c) for the 16 populations of *Malania oleifera* inferred by ADMIXTURE.

Supplementary Fig. S8. The inference of population structure (a), principal component analysis (b) and NJ tree (c) of *Malania oleifera* based on all loci.

Supplementary Fig. S9. The inference of population structure (a), principal component analysis (b) and NJ tree (c) of *Malania oleifera* based on neutral loci.

Supplementary Fig. S10. Demographic history of *Malania oleifera* inferred by Stairway Plot v.2 based on folded SFS.

Supplementary Fig. S11. Runs of homozygosity (ROH) frequency differences among 16 populations of *Malania oleifera.* The dashed lines correspond to the mean ROH lengths for each population.

Supplementary Fig. S12. The Venn diagrams of share and private homozygous deleterious mutations of JM (a), SG (b) and BM2 (c) populations with candidate pollen donors.

Supplementary Fig. S13. Differences in the number of deleterious mutations detected by REF-ALT strategy and ancestral status-based strategy of *Malania oleifera* (a) and *Acer yangbiense* (b).

Supplementary Fig. S14. The importance of environmental variables inferred by gradient forest modeling. *Top uncorrelated environment variables (Pearson's $|r| < 0.7$) used in BayeScEnv, RDA and GF analysis.

Supplementary Fig. S15. Integrated results of ecological niche modelling based on five models in the current (a) and future SSP126 (b) and SSP585 (c) scenarios. Higher values represent higher suitability.

Supplementary Fig. S16. Correlation of altitude with BIO1, BIO2, BIO4, BIO13 and BIO14 climate variables used in GO analysis.

Supplementary Table S1. Geographical location of all sampled individuals of *Malania oleifera*.

Supplementary Table S2. Statistical analysis of resequencing data before and after filtering with Fastp.

Supplementary Table S3. Statistical analysis of each individual mapping to the *Malania oleifera* reference genome.

Supplementary Table S4. Statistics on Watterson's θ (θW) of *Malania oleifera* populations within whole genome, CDS, fold-0, fold-4, intergenic and intron regions.

Supplementary Table S5. Statistics of heterozygosity and homozygosity rates of all individuals used in the whole genome resequencing.

Supplementary Table S6. Weighted fixation statistics (Fst) between populations of all loci, adaptive loci and neutral loci, and geographical distances between populations of *Malania oleifera*.

Supplementary Table S7. Estimation of runs of homozygosity (ROH) and frequency of runs of homozygosity (FROH).

Supplementary Table S8. Summary of deleterious, tolerated, and synonymous mutations of *Malania oleifere* based on REF-ALT strategy.

Supplementary Table S9. A list of 19 bioclimatic variables used in this study.

Supplementary Table S10. The projection coordinates of *M. oleifera* distribution records for ecological niche modelling.

Supplementary Table S11. Environment-associated SNPs and corresponding genes detected by BayeScEnv and Redundancy Analysis (RDA).

Supplementary Table S12. Gene ontology enrichment analysis of environment-associated genetic variants.

Supplementary Table S13. Predicted GO and NSC values of populations under future SSP126 and SSP585 scenarios using BCC-CSM2-MR, CNRM-CM6-1 and CNRM-ESM2-1 climate models.

Supplementary Table S14. The evaluation of ecological niche models.

Supplementary Table S15. Comparison of nucleic acid diversity of endangered species.

Supplementary Table S16. Information of 17 individuals for ancestral sequence reconstruction.

Supplementary Table S17. The genome list of 17 published species used in our research to estimate mutation rate for *Malania oleifera*.

Authors' contributions

Y.P.M., W.B.S. and R.G.Z. designed the study. G.Y. collected and prepared the materials. Y.T.S., L.D.T., M.J.Z. and R.G.Z. performed the research and analyzed the data. Y.T.S. wrote the manuscript. Y.P.M. and W.B.S. revised the manuscript. All authors approved the final manuscript.

Competing Interests

We declare we have no competing interests.

Funding

This work was supported by the Key Project of Natural Science Foundation of Yunnan Province (Grant No. 202001AS070019), the CAS "Light of West China" Program and the Ten Thousand Talent Program of Yunnan Province (Grant No. YNWRQNBJ-2018-174).

Ethics Statement

All plant molecular materials and specimens were collected with permission.

References

1. Miraldo A, Li S, Borregaard MK, et al. An Anthropocene map of genetic diversity. Science 2016;353(6307):1532-35. doi:10.1126/science.aaf4381.
2. Lynch M, Conery J, Burger R. Mutation accumulation and the extinction of small populations. American Society of Naturalists 1995;146(4):489-518. doi:https://doi.org/10.1086/285812.
3. Charlesworth D, Willis JH. The genetics of inbreeding depression. Nat Rev Genet 2009;10(11):783-96. doi:10.1038/nrg2664.
4. Xue Y, Prado-Martinez J, Sudmant PH, et al. Mountain gorilla genomes reveal the impact of long-term population decline and inbreeding. Science 2015;348(6231):242-45. doi:10.1126/science.aaa3952.
5. Feng S, Fang Q, Barnett R, et al. The genomic footprints of the fall and recovery of the crested ibis. Curr Biol 2019;29(2):340-49 e7. doi:10.1016/j.cub.2018.12.008.
6. Keller MC, Visscher PM, Goddard ME. Quantification of inbreeding due to distant ancestors and its detection using dense single nucleotide polymorphism data. Genetics 2011;189(1):237-49. doi:10.1534/genetics.111.130922.
7. Ma Y, Liu D, Wariss HM, et al. Demographic history and identification of threats revealed by population genomic analysis provide insights into conservation for an endangered maple. Mol Ecol 2022;31(3):767-79. doi:10.1111/mec.16289.
8. Hedrick PW, Garcia-Dorado A. Understanding inbreeding depression, purging, and genetic rescue. Trends Ecol Evol 2016;31(12):940-52. doi:10.1016/j.tree.2016.09.005.
9. Caballero A, Bravo I, Wang J. Inbreeding load and purging: implications for the short-term survival and the conservation management of small populations. Heredity (Edinb) 2017;118(2):177-85. doi:10.1038/hdy.2016.80.
10. Robinson JA, Kyriazis CC, Nigenda-Morales SF, et al. The critically

endangered vaquita is not doomed to extinction by inbreeding depression. Science 2022;376(6593):635-39. doi:10.1126/science.abm1742.

11. Malcolm JR, Liu C, Neilson RP, et al. Global warming and extinctions of endemic species from biodiversity hotspots. Conserv Biol 2006;20(2):538-48. doi:10.1111/j.1523-1739.2006.00364.x.

12. Wiens JJ. Climate-related local extinctions are already widespread among plant and animal Species. PLoS Biol 2016;14(12):e2001104. doi:10.1371/journal.pbio.2001104.

13. Bay RA, Harrigan RJ, Underwood VL, et al. Genomic signals of selection predict climate-driven population declines in a migratory bird. Science 2018;359(6371):83-86. doi:10.1126/science.aan4380.

14. Aitken SN, Yeaman S, Holliday JA, et al. Adaptation, migration or extirpation: climate change outcomes for tree populations. Evol Appl 2008;1(1):95-111. doi:10.1111/j.1752-4571.2007.00013.x.

15. Aguirre-Liguori JA, Ramirez-Barahona S, Gaut BS. The evolutionary genomics of species' responses to climate change. Nat Ecol Evol 2021;5(10):1350-60. doi:10.1038/s41559-021-01526-9.

16. Chen Y, Jiang Z, Fan P, et al. The combination of genomic offset and niche modelling provides insights into climate change-driven vulnerability. Nat Commun 2022;13(1):4821. doi:10.1038/s41467-022-32546-z.

17. Jia KH, Zhao W, Maier PA, et al. Landscape genomics predicts climate change-related genetic offset for the widespread *Platycladus orientalis* (Cupressaceae). Evol Appl 2020;13(4):665-76. doi:10.1111/eva.12891.

18. Sang Y, Long Z, Dan X, et al. Genomic insights into local adaptation and future climate-induced vulnerability of a keystone forest tree in East Asia. Nat Commun 2022;13(1):6541. doi:10.1038/s41467-022-34206-8.

19. Yang H, Li J, Milne RI, et al. Genomic insights into the genotype-environment mismatch and conservation units of a Qinghai-Tibet Plateau endemic cypress under climate change. Evol Appl 2022;15(6):919-33. doi:10.1111/eva.13377.

20. Li SG. *Malania*, a new genus of oil-yielding plant. Bulletin of Botanical Laboratory of North-Eastern Forestry Institute 1980;1:67-72.

21. Lv SH, Wei CQ, Huang FZ, et al. Fruit and seed traits and adaptability to rocky desertification mountain of rare tree species *Malania oleifera*. Chinese Journal of Ecology 2016;35(1):57-62. doi:10.13292/1.1000-4890.201601.008.

22. Yang T, Yu Q, Xu W, et al. Transcriptome analysis reveals crucial genes involved in the biosynthesis of nervonic acid in woody *Malania oleifera* oilseeds. BMC Plant Biol 2018;18(1):247. doi:10.1186/s12870-018-1463-6.

23. Lu SG, Lei LB, Yang QS, et al. The current status and the cause of the endangerment of *Malania oleifera* Chun et Lee in southeast Yunnan. In: Chen YY, (ed.). *Biodiversity Conservation and Regional Sustainable Development-The 4th Biodiversity Conservation and Sustainable Use Conference*. China Forestry Press, 2000, p. 169-72.

24. Xu DB, Chen F, Guo XC, et al. Research on the bottleneck of resource protection and industrialization development of rarely endangered *Malania oleifera*. Issues of Forestry Economics 2018;38(3):13-20. doi:10. 16832 /j. cnki. 1005-9709. 2018. 03. 003.
25. Wu YQ, Li XD, Hu YJ. Reproductive biology of *Malania oleifera*. Acta Scientiarum Naturalium Universitatis Sunyatseni 2004;43(2):81-83.
26. Lai JY, Shi HM, Pan CL, et al. Pollination biology of rare and endangered species *Malania oleifera* Chun et Lee. Journal of Beijing Forestry University 2008;30(2):59-64. doi:10.13332/j.1000-1522.2008.02.021.
27. Li XD. Life-table analysis of *Malania oleifera*, a rare and endangered plant. Journal of Central South University of Forestry & Technology 2009;29(2):73-76.
28. Xu SS, Kan W, Kong BH, et al. First report of *Fusarium oxysporum* and *Fusarium solani* causing root rot on *Malania oleifera* in China. Plant Disease 2020;104(2):584-84. doi:10.1094/PDIS-07-19-1426-PDN.
29. Fu LG. Red data book of Chinese plant-the rare and endangered plants. Beijing: Science Press; 1992.
30. Ma Y, Chen G, Edward Grumbine R, et al. Conserving plant species with extremely small populations (PSESP) in China. Biodiversity and Conservation 2013;22(3):803-09. doi:10.1007/s10531-013-0434-3.
31. Yang T, Zhang R, Tian X, et al. The chromosome-level genome assembly and genes involved in biosynthesis of nervonic acid of *Malania oleifera*. Sci Data 2023;10(1):298. doi:10.1038/s41597-023-02218-8.
32. Chen W, Wang P, Pu T, et al. Symbiotic effect of co-cultivated plants on *Malania oleifera* seedlings. Acta Agriculturae Universitatis Jiangxiensis 2022;44(5):1197-206. doi:10.13836/j.jjau.2022119.
33. Chen Q, Li Y, Li Y, et al. Dynamics of tissue nutrient content in relation to declining seedling growth in *Malania Oleifera*. Guihaia 2024;44(1):137-46. doi:10.11931/guihaia.gxzw202303048.
34. Su C, Wang G, Gao Y, et al. Resource protection and development counterplants of *Malania Oleifera*. J Anhui Agric Sci 2023;51(12):104-07. doi:10.3969/j.issn.0517-6611.2023.12.024.
35. Supple MA, Shapiro B. Conservation of biodiversity in the genomics era. Genome Biology 2018;19(1) doi:10.1186/s13059-018-1520-3.
36. Doyle JJ, Doyle JL. A rapid DNA isolation procedure for small quantities of fresh leaf tissue. Phytochemical Bulletin 1987;19(1):11-15. doi:10.1016/0031-9422(80)85004-7.
37. Chen S, Zhou Y, Chen Y, et al. fastp: an ultra-fast all-in-one FASTQ preprocessor. Bioinformatics 2018;34(17):i884-i90. doi:10.1093/bioinformatics/bty560.
38. Li H. Aligning sequence reads, clone sequences and assembly contigs with BWA-MEM. arXiv: Genomics 2013; https://doi.org/10.48550/arXiv.1303.3997.

39. Danecek P, Bonfield JK, Liddle J, et al. Twelve years of SAMtools and BCFtools. Gigascience. 2021,10(2):giab008. doi: 10.1093/gigascience/giab008
40. Tarasov A, Vilella AJ, Cuppen E, et al. Sambamba: fast processing of NGS alignment formats. Bioinformatics 2015;31(12):2032-4. doi:10.1093/bioinformatics/btv098.
41. Garrison E, Marth G. Haplotype-based variant detection from short-read sequencing. arXiv:12073907 [q-bioGN] 2012; doi:10.48550/arXiv.1207.3907.
42. Danecek P, Auton A, Abecasis G, et al. The variant call format and VCFtools. Bioinformatics 2011;27(15):2156-8. doi:10.1093/bioinformatics/btr330.
43. Zhang C, Dong SS, Xu JY, et al. PopLDdecay: a fast and effective tool for linkage disequilibrium decay analysis based on variant call format files. Bioinformatics 2019;35(10):1786-88. doi:10.1093/bioinformatics/bty875.
44. Korneliussen TS, Albrechtsen A, Nielsen R. ANGSD: Analysis of next generation sequencing data. BMC Bioinformatics 2014;15(1):356. doi:10.1186/s12859-014-0356-4.
45. Yang Y, Ma T, Wang Z, et al. Genomic effects of population collapse in a critically endangered ironwood tree *Ostrya rehderiana*. Nat Commun 2018;9(1):5449. doi:10.1038/s41467-018-07913-4.
46. Frichot E, François O, O'Meara B. LEA: An R package for landscape and ecological association studies. Methods in Ecology and Evolution 2015;6(8):925-29. doi:10.1111/2041-210x.12382.
47. Luu K, Bazin E, Blum MG. pcadapt: an R package to perform genome scans for selection based on principal component analysis. Mol Ecol Resour 2017;17(1):67-77. doi:10.1111/1755-0998.12592.
48. Chang C , Chow C, Tellier L, et al. Second-generation PLINK: Rising to the Challenge of Larger and Richer Datasets. Gigascience. 2015, doi: 10.1186/s13742-015-0047-8.
49. Alexander DH, Novembre J, Lange K. Fast model-based estimation of ancestry in unrelated individuals. Genome Res 2009;19(9):1655-64. doi:10.1101/gr.094052.109.
50. Yang J, Lee SH, Goddard ME, et al. GCTA: a tool for genome-wide complex trait analysis. Am J Hum Genet 2011;88(1):76-82. doi:10.1016/j.ajhg.2010.11.011.
51. Kumar S, Stecher G, Tamura K. MEGA7: Molecular evolutionary genetics analysis version 7.0 for bigger datasets. Mol Biol Evol 2016;33(7):1870-4. doi:10.1093/molbev/msw054.
52. Liu X, Fu YX. Stairway Plot 2: demographic history inference with folded SNP frequency spectra. Genome Biol 2020;21(1):280. doi:10.1186/s13059-020-02196-9.
53. Schiffels S, Durbin R. Inferring human population size and separation history from multiple genome sequences. Nat Genet 2014;46(8):919-25. doi:10.1038/ng.3015.

54. Sim NL, Kumar P, Hu J, et al. SIFT web server: predicting effects of amino acid substitutions on proteins. Nucleic Acids Res 2012;40(Web Server issue):W452-7. doi:10.1093/nar/gks539.
55. Boeckmann B, Bairoch A, Apweiler R, et al. The SWISS-PROT protein knowledgebase and its supplement TrEMBL in 2003. Nucleic Acids Res 2003;31(1):365-70. doi:10.1093/nar/gkg095.
56. Vaser R, Adusumalli S, Leng SN, et al. SIFT missense predictions for genomes. Nat Protoc 2016;11(1):1-9. doi:10.1038/nprot.2015.123.
57. Ellis N, Smith SJ, Pitcher CR. Gradient forests: calculating importance gradients on physical predictors. Ecology 2012;93(1):156-68. doi:10.1890/11-0252.1.
58. Friendly M. Corrgrams: Exploratory displays for correlation matrices. The American Statistician 2002;56(4):316-24. doi:10.1198/000313002533.
59. Villemereuil P, Gaggiotti OE. A new FST-based method to uncover local adaptation using environmental variables. Methods in Ecology and Evolution 2015;6(11):1248-58. doi:10.1111/2041-210x.12418.
60. Legendre P, Legendre L. Numerical ecology. Elsevier; 2012.
61. Lischer HE, Excoffier L. PGDSpider: an automated data conversion tool for connecting population genetics and genomics programs. Bioinformatics 2012；28(2):298-9. doi: 10.1093/bioinformatics/btr642.
62. Forester BR, Lasky JR, Wagner HH, et al. Comparing methods for detecting multilocus adaptation with multivariate genotype-environment associations. Mol Ecol 2018;27(9):2215-33. doi:10.1111/mec.14584.
63. Oksanen J, Blanchet FG, Kindt R, et al. Package 'vegan': Community ccology package. 2013. R package version 2.3-0. https://cran.r-project.org/web/packages/vegan/index.html.
64. Cantalapiedra CP, Hernandez-Plaza A, Letunic I, et al. eggNOG-mapper v2: Functional annotation, orthology assignments, and domain prediction at the metagenomic scale. Mol Biol Evol 2021;38(12):5825-29. doi:10.1093/molbev/msab293.
65. Yu X, Dai M, Pu T, et al. Population structure and dynamics analysis of rare and endangered plant *Malania oleifera*. Journal of West China Forestry Science 2023;52(3):8-16. doi: 10.16473/j.cnki.xblykx1972.2023.03.002.
66. Gong MJ, Wang J, Fu XY, et al. Suitable regions forecasting and environmental influencing factors of *Malania oleifera* in Yunnan and Guangxi. Journal of Nanjing Forestry University (Natural Sciences Edition) 2022;46(2):44-52. doi:10. 12302/j. issn. 1000-2006. 202109039.
67. Brown JL, Carnaval AC. A tale of two niches: methods, concepts, and evolution. Frontiers of Biogeography 2019;11(4) doi:10.21425/f5fbg44158.
68. Naimi B, Hamm NAS, Groen TA, et al. Where is positional uncertainty a problem for species distribution modelling? Ecography 2013;37(2):191-203. doi:10.1111/j.1600-0587.2013.00205.x.

69. Wood SN. Fast stable restricted maximum likelihood and marginal likelihood estimation of semiparametric generalized linear models. J R Statist Soc B 2011;73:3-36. doi: 10.1111/j.1467-9868.2010.00749.x.
70. Hijmans RJ, Phillips S, Leathwick J, et al. dismo: Species distribution Modelling. 2023. R package version 1.3-14. https://CRAN.R-project.org/package=dismo.
71. Liaw A, Wiener M. Classification and regression by randomForest. R news 2002;2:18-22.
72. Friedman J, Tibshirani R, Hastie T. Regularization paths for generalized linear models via coordinate descent. J Stat Softw 2010;33(1):1-22. doi:10.18637/jss.v033.i01.
73. Valavi R, Guillera-Arroita G, Lahoz-Monfort JJ, et al. Predictive performance of presence-only species distribution models: a benchmark study with reproducible code. Ecol Monogr 2022; 92: e01486. doi: 10.1002/ecm.1486
74. Wilfried T, Damien G, Maya G, et al. biomod2: Ensemble platform for species distribution modeling. 2023. R package version 4.2-4. https://CRAN.R-project.org/package=biomod2.
75. Di Cola V, Broennimann O, Petitpierre B, et al. ecospat: an R package to support spatial analyses and modeling of species niches and distributions. Ecography 2017;40(6):774-87. doi:10.1111/ecog.02671.
76. Kull M, Flach P. prg: creates the Precision-Recall-Gain curve and calculates the area under the curve. 2023. R package version 0.5.1. https://github.com/meeliskull/prg.
77. Elizabeth F. PresenceAbsence: Presence-Absence Model Evaluation. 2023. R package version 1.1.11. https://CRAN.R-project.org/package=PresenceAbsence.
78. Guzmán S, Giudicelli GC, Turchetto C, et al. Neutral and outlier single nucleotide polymorphisms disentangle the evolutionary history of a coastal Solanaceae species. Mol Ecol 2022;31(10):2847-64. doi:10.1111/mec.16441.
79. Funk WC, McKay JK, Hohenlohe PA, et al. Harnessing genomics for delineating conservation units. Trends Ecol Evol 2012;27(9):489-96. doi:10.1016/j.tree.2012.05.012.
80. Hu JY, Hao ZQ, Frantz L, et al. Genomic consequences of population decline in critically endangered pangolins and their demographic histories. Natl Sci Rev 2020;7(4):798-814. doi:10.1093/nsr/nwaa031.
81. Fitzpatrick MC, Keller SR. Ecological genomics meets community-level modelling of biodiversity: mapping the genomic landscape of current and future environmental adaptation. Ecol Lett 2015;18(1):1-16. doi:10.1111/ele.12376.
82. Rao W, Shen Z, Duan X. Spatiotemporal patterns and drivers of soil erosion in Yunnan, Southwest China: Rulse assessments for recent 30 years and future predictions based on CMIP6. Catena 2023;220 doi:10.1016/j.catena.2022.106703.

83. Liu H, Zhang M, Lin Z, et al. Spatial heterogeneity of the relationship between vegetation dynamics and climate change and their driving forces at multiple time scales in Southwest China. Agricultural and Forest Meteorology 2018;256-257:10-21. doi:10.1016/j.agrformet.2018.02.015.
84. Theissinger K, Fernandes C, Formenti G, et al. How genomics can help biodiversity conservation. Trends in Genetics 2023;39(7):545-59. doi:10.1016/j.tig.2023.01.005.
85. Kahilainen A, Puurtinen M, Kotiaho JS. Conservation implications of species–genetic diversity correlations. Global Ecology and Conservation 2014;2:315-23. doi:10.1016/j.gecco.2014.10.013.
86. He ZZ, Stotz GC, Liu X, et al. A global synthesis of the patterns of genetic diversity in endangered and invasive plants. Biological Conservation 2024;291 doi:10.1016/j.biocon.2024.110473.
87. Ellegren H, Galtier N. Determinants of genetic diversity. Nat Rev Genet 2016;17(7):422-33. doi:10.1038/nrg.2016.58.
88. Chen H, Hey J, Chen K. Inferring very recent population growth rate from population-scale sequencing data: Using a large-sample coalescent estimator. Mol Biol Evol 2015;32(11):2996-3011. doi:10.1093/molbev/msv158.
89. Dawson TP, Jackson ST, House JI, et al. Beyond predictions: biodiversity conservation in a changing climate. Science 2011;332(6025):53-8. doi:10.1126/science.1200303.
90. Pacifici M, Foden WB, Visconti P, et al. Assessing species vulnerability to climate change. Nature Climate Change 2015;5(3):215-24. doi:10.1038/nclimate2448.
91. Fagny M, Austerlitz F. Polygenic adaptation: Integrating population genetics and gene regulatory networks. Trends Genet 2021;37(7):631-638. doi: 10.1016/j.tig.2021.03.005.
92. Yuan S, Shi Y, Zhou BF, et al. Genomic vulnerability to climate change in *Quercus acutissima*, a dominant tree species in East Asian deciduous forests. Mol Ecol 2023;32(7):1639-55. doi:10.1111/mec.16843.
93. Thuiller W. Ecological niche modelling. Current Biology 2024;34(6):R225-R29. doi:10.1016/j.cub.2024.02.018.
94. Jia D, Mao J, Chen F, et al. Investigation and analysis of wild garlic fruit resources in Guangnan. Forest By-Product and Speciality in China 2017;3:72-76. doi:10.13268/j.cnki, fbsic.2017.03.032.
95. Liu Y, Ning S. Status and evaluation of natural resources of emphasis protective wilding plant in Guangxi. Guangxi Sciences 2002;9(2):124-32. doi:10.13656/j.cnki, gxkx.2002.02.012.
96. Barbosa S, Mestre F, White TA, et al. Integrative approaches to guide conservation decisions: Using genomics to define conservation units and functional corridors. Mol Ecol 2018;27(17):3452-65. doi:10.1111/mec.14806.
97. Pavlova A, Beheregaray LB, Coleman R, et al. Severe consequences of habitat

fragmentation on genetic diversity of an endangered Australian freshwater fish: A call for assisted gene flow. Evol Appl 2017;10(6):531-50. doi:10.1111/eva.12484.

98. Frankham R, Ballou JD, Eldridge MD, et al. Predicting the probability of outbreeding depression. Conserv Biol 2011;25(3):465-75. doi: 10.1111/j.1523-1739.2011.01662.x.

99. Severns PM. Precautionary hand pollination suggests outbreeding depression between potential seed donor populations for a rare wetland plant. J Torrey Bot Soc 2013;140(1), 20-25. doi: 10.3159/TORREY-D-12-00046.1.

100. Shen YT, Tao LD, Yao G, et al. Supporting data for "Genomic insights into endangerment and conservation of the garlic-fruit tree (Malania oleifera), a plant species with extremely small populations" GigaScience Database. 2024. https://doi.org/10.5524/102555.

Table

Table 1. Sample sizes and nucleotide diversity (θ_π) in *M. oleifera* populations within the whole genome, CDS, fold-0, fold-4, intergenic and intron regions.

**Population**	**Sample size**	**Number of SNPs**	**θ_π_whole × 10^{-3}**	**θ_π_CDS × 10^{-3}**	**θ_π_fold-0 × 10^{-3}**	**θ_π_fold-4 × 10^{-3}**	**θ_π_intergenic × 10^{-3}**	**θ_π_intron × 10^{-3}**
BB1	10	141725	3.10 ± 0.42	1.19 ± 0.15	0.99 ± 0.13	1.93 ± 0.23	3.52 ± 0.49	1.83 ± 0.18
BB2	10	132480	2.79 ± 0.41	1.08 ± 0.16	0.90 ± 0.13	1.75 ± 0.25	3.17 ± 0.47	1.68 ± 0.24
BL	10	146763	3.56 ± 0.47	1.38 ± 0.17	1.15 ± 0.14	2.19 ± 0.27	4.07 ± 0.57	2.12 ± 0.25
BM1	17	218460	6.13 ± 0.58	2.41 ± 0.18	2.00 ± 0.15	3.92 ± 0.26	7.56 ± 0.44	3.87 ± 0.20
BM2	5	101436	2.15 ± 0.25	0.86 ± 0.13	0.73 ± 0.11	1.34 ± 0.17	2.45 ± 0.29	1.30 ± 0.13
DX	10	155019	4.57 ± 0.57	1.73 ± 0.27	1.44 ± 0.22	2.77 ± 0.43	5.20 ± 0.62	2.71 ± 0.39
FS	9	189667	5.13 ± 0.41	1.94 ± 0.22	1.61 ± 0.18	3.15 ± 0.31	5.86 ± 0.45	3.04 ± 0.26
GL	10	137835	3.56 ± 0.45	1.35 ± 0.18	1.13 ± 0.15	2.13 ± 0.26	4.11 ± 0.52	2.05 ± 0.26
JM	10	98031	2.07 ± 0.40	0.83 ± 0.20	0.69 ± 0.17	1.34 ± 0.30	2.36 ± 0.46	1.25 ± 0.22
LY1	10	157624	3.89 ± 0.47	1.47 ± 0.22	1.22 ± 0.19	2.39 ± 0.33	4.47 ± 0.52	2.25 ± 0.31
LY2	14	200098	5.50 ± 0.27	2.03 ± 0.19	1.68 ± 0.16	3.28 ± 0.26	6.28 ± 0.27	3.20 ± 0.21
ML	10	151563	3.53 ± 0.40	1.34 ± 0.18	1.12 ± 0.15	2.13 ± 0.26	4.05 ± 0.47	2.08 ± 0.21
NP	10	106945	2.03 ± 0.48	0.82 ± 0.25	0.69 ± 0.20	1.30± 0.38	2.31 ± 0.54	1.25 ± 0.30
SG	10	133980	2.75 ± 0.34	1.10 ± 0.20	0.91 ± 0.17	1.80 ± 0.30	3.12 ± 0.40	1.65 ± 0.18
ZL	10	169320	4.48 ± 0.41	1.67 ± 0.21	1.41 ± 0.17	2.61 ± 0.32	5.15 ± 0.43	2.59 ± 0.29
ZS	10	163672	4.35 ± 0.32	1.63 ± 0.14	1.36 ± 0.12	2.60 ± 0.21	4.99 ± 0.36	2.55 ± 0.20

Figures

Figure 1. Population genomics of *M. oleifera.* (a) Geographic distribution and sampled populations of *M. oleifera*. Different colors in the pie chart represent the genetic groups identified by ADMIXTURE based on adaptive loci, and the size of the pie corresponds to the level of heterozygosity. The optimal population genetic structure of *M. oleifera* with K = 10 (b) and a neighbor-joining (NJ) phylogenetic tree (c) based on adaptive loci. Samples in the STRUCTURE and phylogenetic tree results correspond. Node bootstrap values below 0.8 are not shown. (d) Results of PCA based on adaptive loci, with the first two PCs explaining 25.2% of the genome covariance. Populations are defined as BB1 = Banbeng, BB2 = Babao, BL = Banlun, BM1 = Bama, BM2 = Bamei, DX = Daxin, FS = Fengshan, GL = Gaolong, JM = Jiumo, LY1 = Leye, LY2 = Linyun, ML = Mulun, NP = Nanping, SG = Shuguang, ZL = Zhemiao, ZS = Zhesang.

Figure 2. Demographic history of *M. oleifera* inferred by Stairway Plot v.2 based on unfolded SFS (a) and MSMC v.2 within two, four and eight haplotypes (b). The light blue lines correspond to the upper and lower bounds of the 95% confidence intervals. The severe effective population size (*Ne*) declines observed during the last glacial maximum (LGM) and the Middle Pleistocene are highlighted with gray vertical bars.

Figure 3. Levels of inbreeding and genetic load in different *M. oleifera* populations. (a) Fractions of the runs of homozygosity (FROH) show discrepancies in inbreeding levels in *M. oleifera* populations. (b) Distributions of long (> 1 Mb) and medium (100 kb-1 Mb) runs of homozygosity (ROH) among 16 populations of *M. oleifera.* Solid dots represent ROH > 1 Mb and hollow dots represent 1 Mb > ROH > 100 Kb. (c) Ratios of homozygous-derived deleterious mutations show the discrepancies in genetic load in different *M. oleifera* populations. Populations marked with the same letters in (a) and (c) are not significantly different. (d) Distributions of the ratio of 0- to 4-fold heterozygosity versus the intergenic heterozygosity across the 16 populations of *M. oleifera*. The dark line represents the significant negative correlation between these populations, with $R = -0.21$ and $p = 0.006$. Each dot represents an individual, which is colored by population.

Figure 4. Predicted genetic offset of *M. oleifera* in the year 2100 under the SSP126 and SSP585 scenarios based on all SNPs (a, b) and adaptive SNPs (c, d), with higher values (red) representing more severe genomic vulnerability to future climate change. The inner mini plot represents the correlation between altitude and GO value in the corresponding scenario.

Figure 5. Predicted niche suitability change (NSC) of *M. oleifera* in the year 2100 under the SSP126 (a) and SSP585 (b) scenarios. Higher positive value suggests more severe degree of unsuitability.

Figure1

Click here to access/download;Figure;Figure 1.jpg

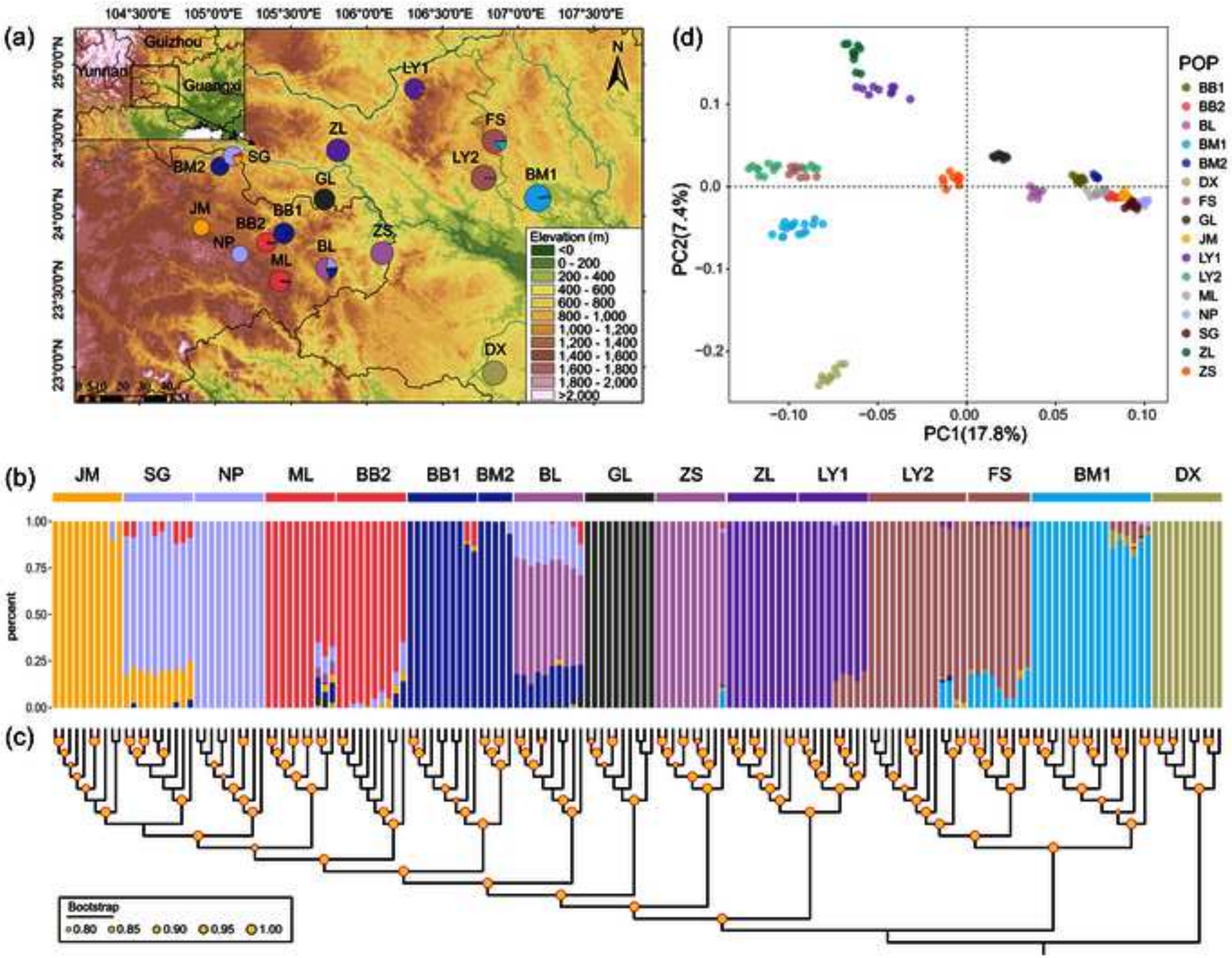

Figure2

Click here to access/download;Figure;Figure 2.jpg

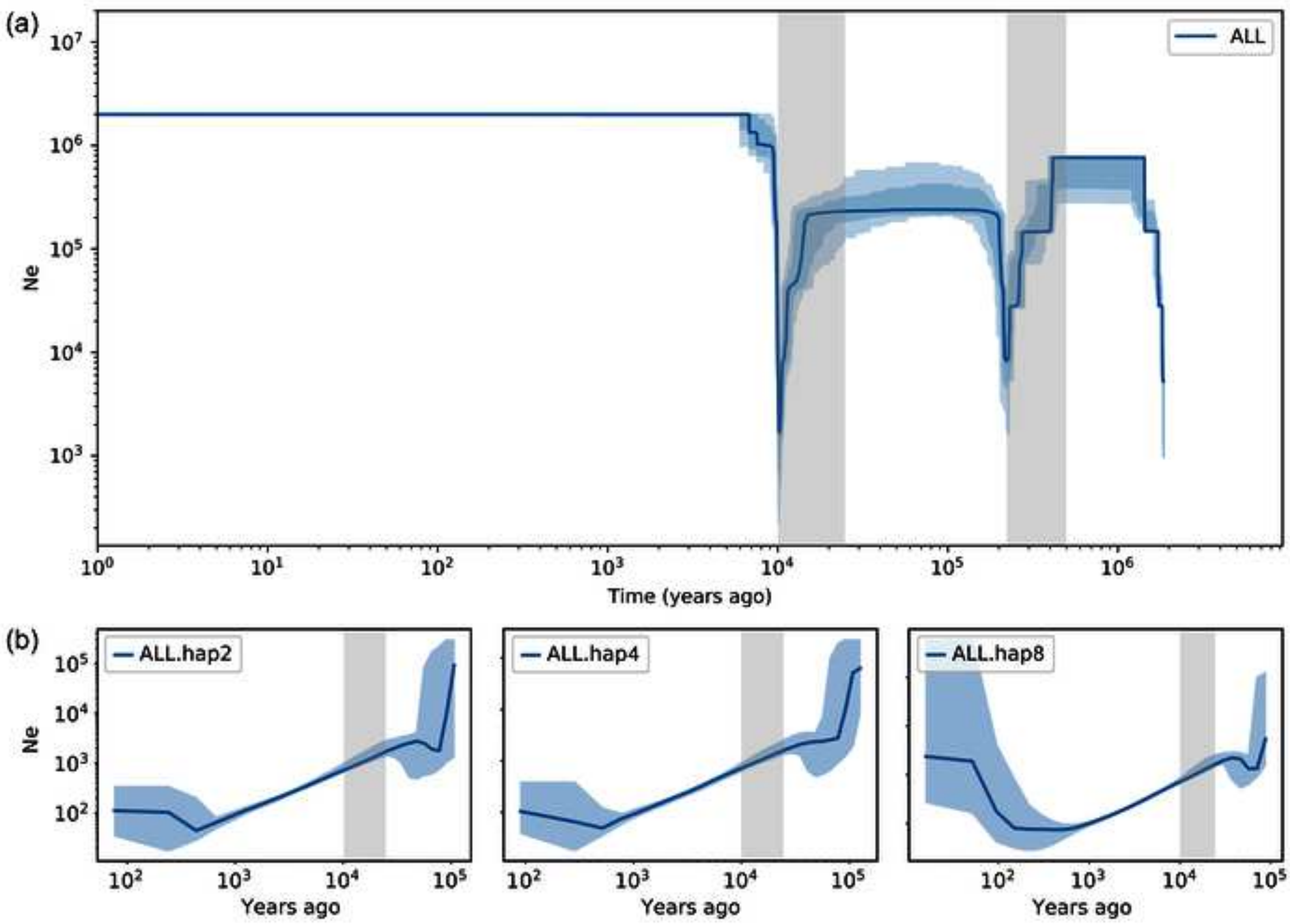

Figure3

Click here to access/download;Figure;Figure 3.jpg

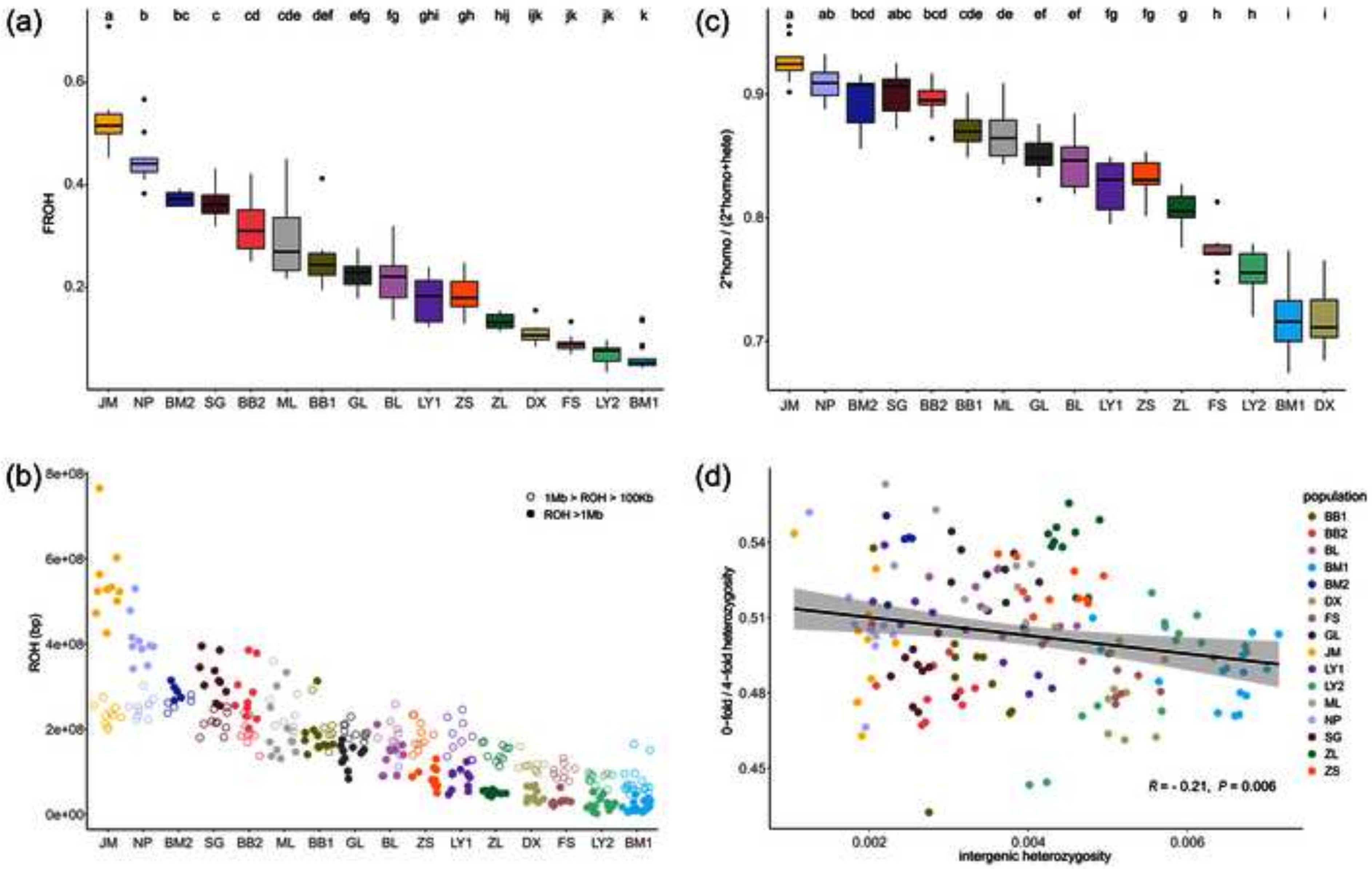

Figure4

Click here to access/download;Figure;2024-07-17Figure 4.jpg

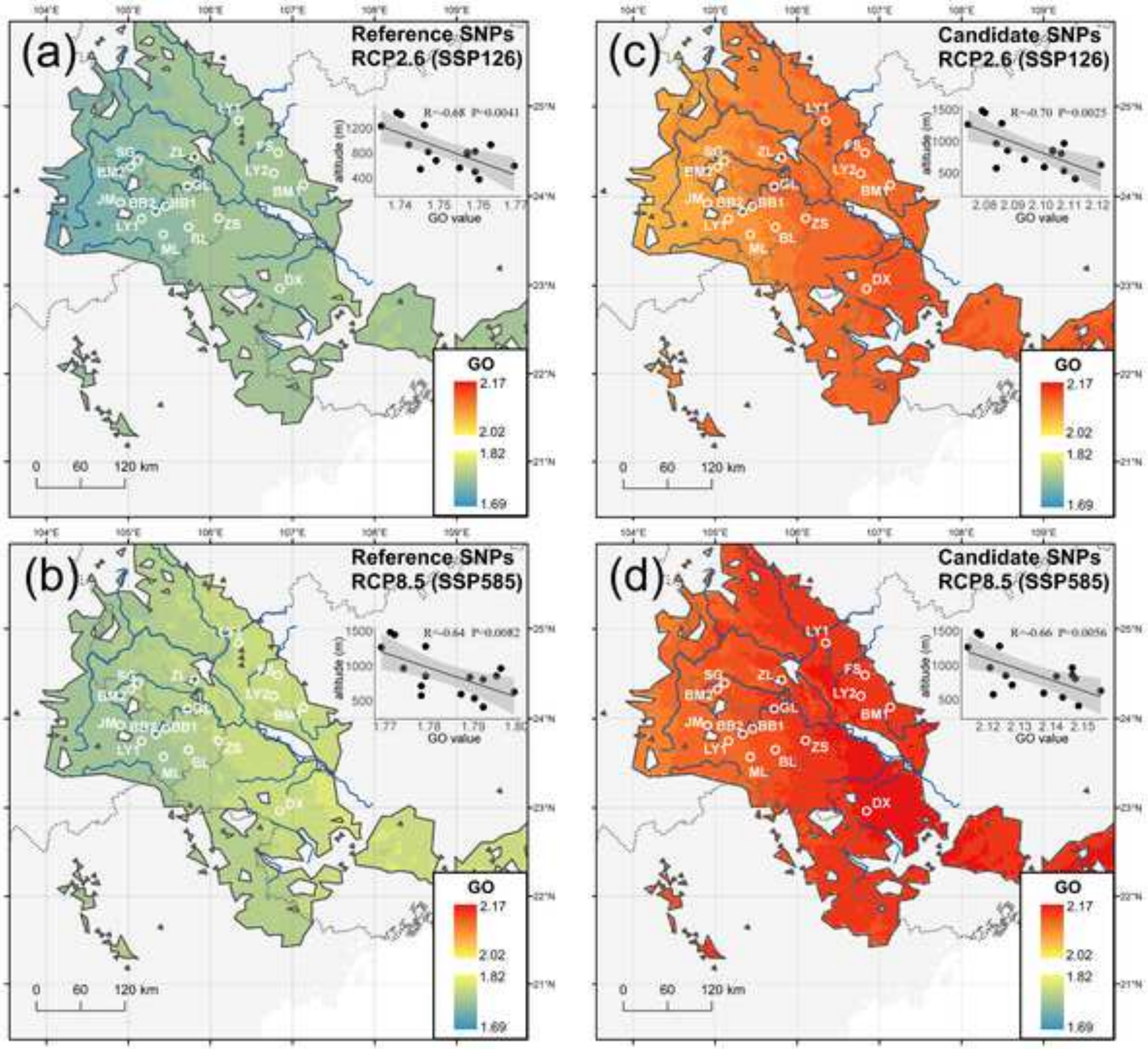

 Click here to access/download;Figure;Figure 5.jpg

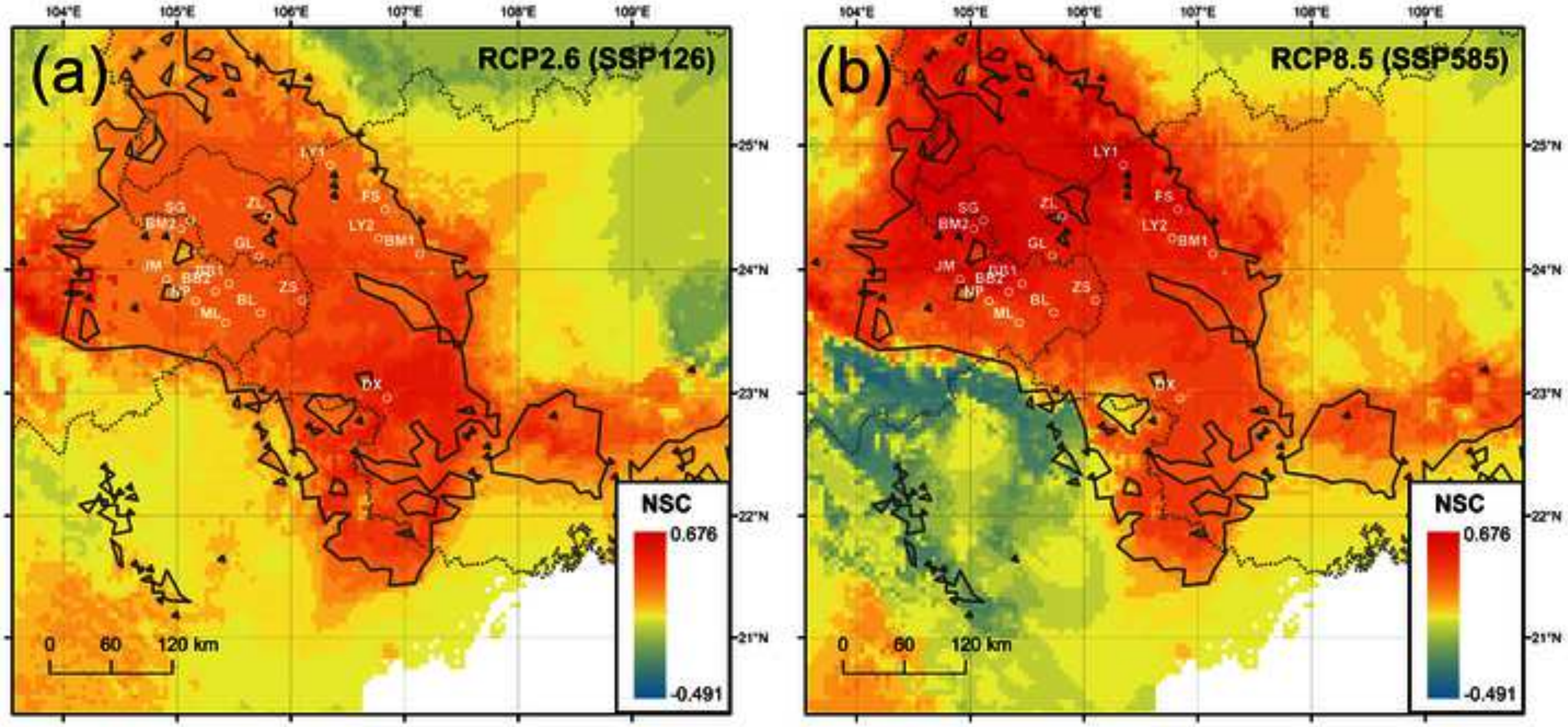

Click here to access/download
Supplementary Material
Supplementary note S1-S3.docx

Supplementary Figure

Click here to access/download
Supplementary Material
2024-07-17Supplementary figure S1-S16.docx

Supplementary Table

Click here to access/download
Supplementary Material
2024-07-17supplementary table S1-S17.xlsx

Dear editor,

Thanks for your efforts and three reviewers' comments regarding our manuscript entitled "Genomic insights into endangerment and conservation of the garlic-fruit tree (*Malania oleifera*), a plant species with extremely small populations" (No: GIGA-D-24-00159). These comments and suggestions are highly constructive and helpful for strengthening our paper.

We have revised the manuscript carefully according to the comments of three reviewers, with revised words marked in blue in the resubmitted paper. Our point-by-point responses to the reviewers' comments are shown below. Hopefully, this version is now suitable for the publication in GigaScience.

If you have any further questions or requirements, please do not hesitate to contact the corresponding author.

Sincerely yours,

Yongpeng Ma
