# Supplementary material for: Genomic insights into endangerment and conservation of the garlic-fruit tree (Malania oleifera), a plant species with extremely small populations
Source: Gigascience. 2024 Sep 23;13:giae070. doi: 10.1093/gigascience/giae070 (PMC11417964; doi:10.1093/gigascience/giae070)
Supplement: giae070_Supplemental_Files [file giae070_supplemental_files.zip › 2024-07-17Supplementary figure S1-S16.docx]

**
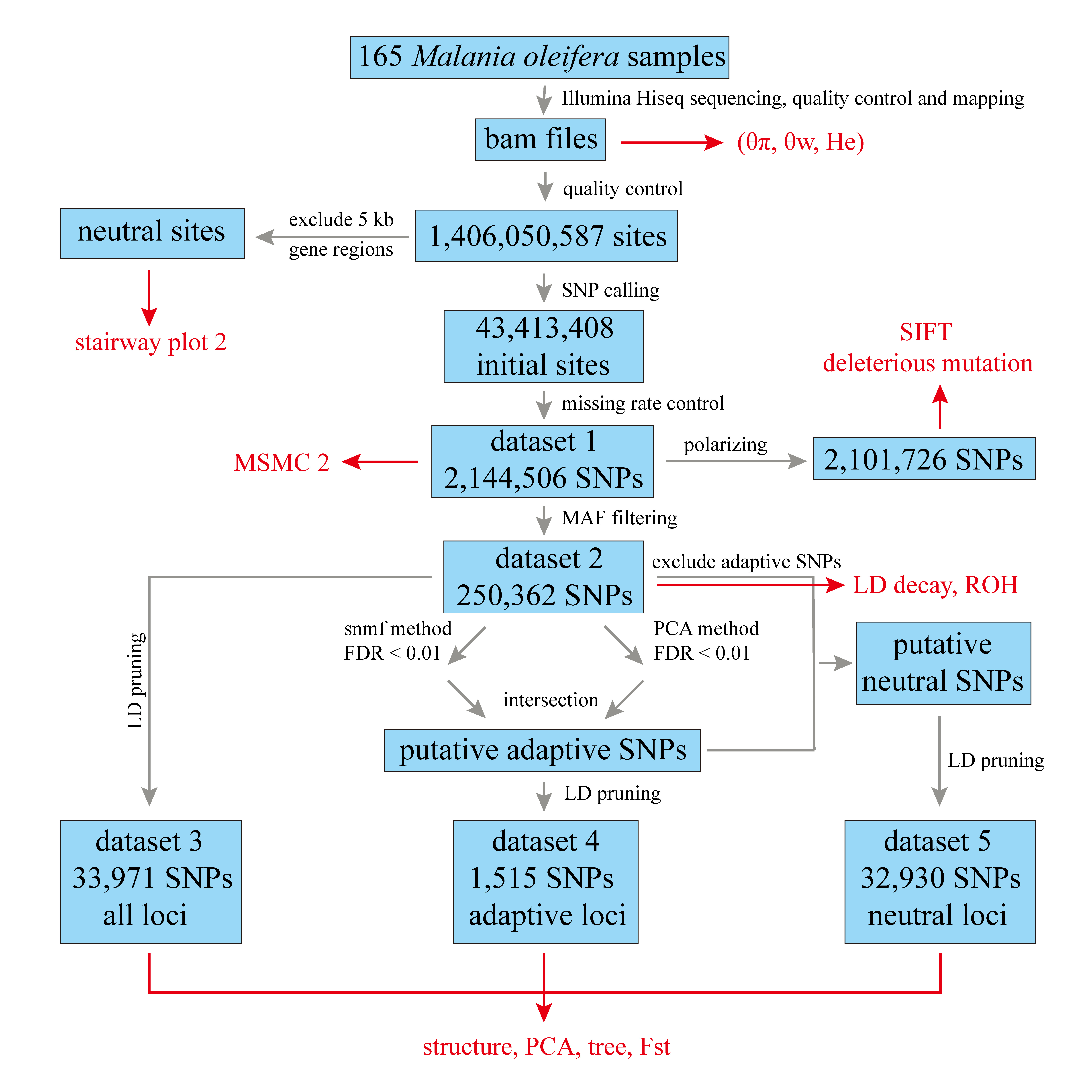
**

**Figure S1.** Resequencing data processing workflow of *Malania oleifera.*


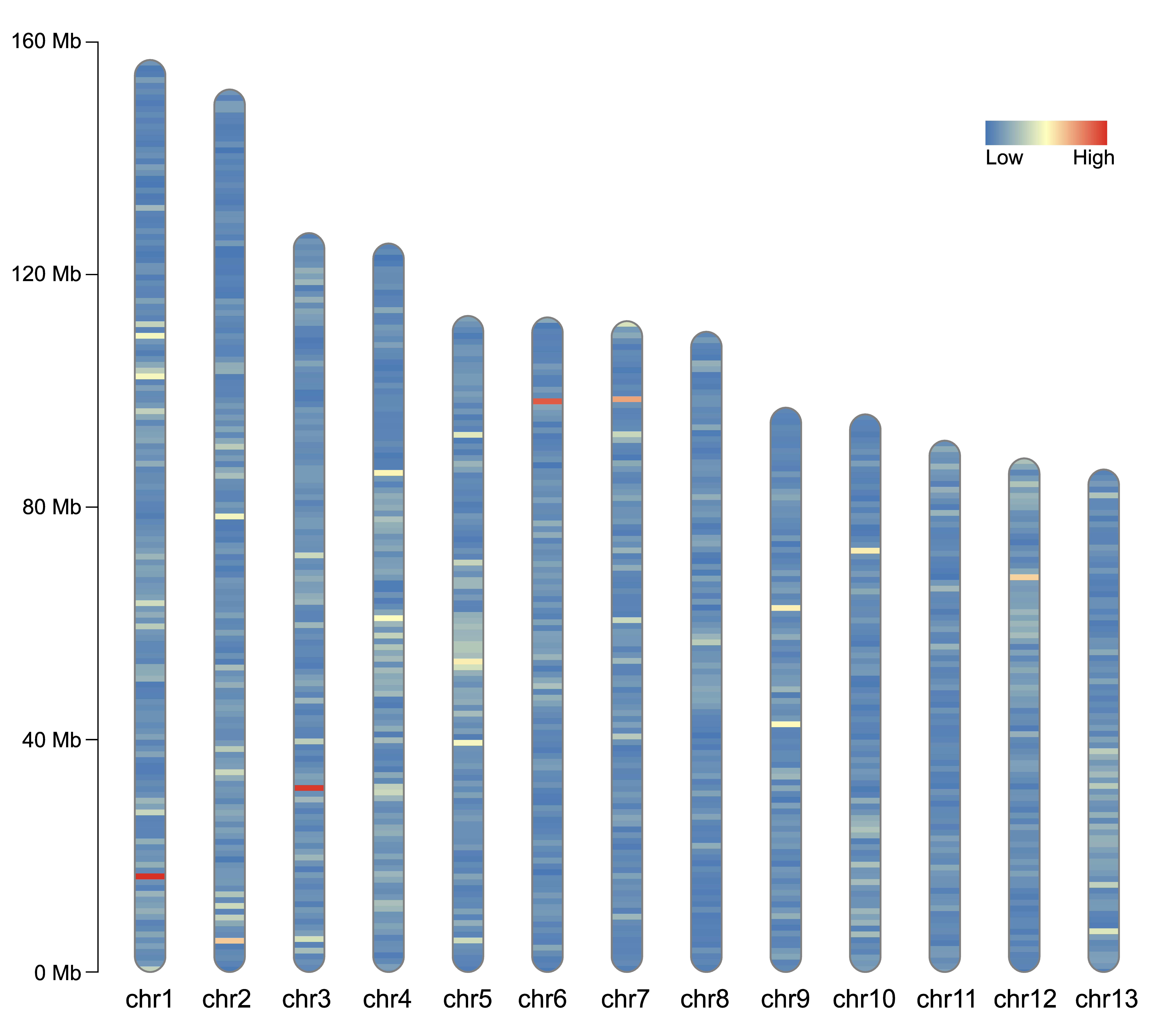


**Figure S2.** The distribution of SNPs (dataset 2) across 13 pseudochromosomes under 1 Mb windows.

(a)

**
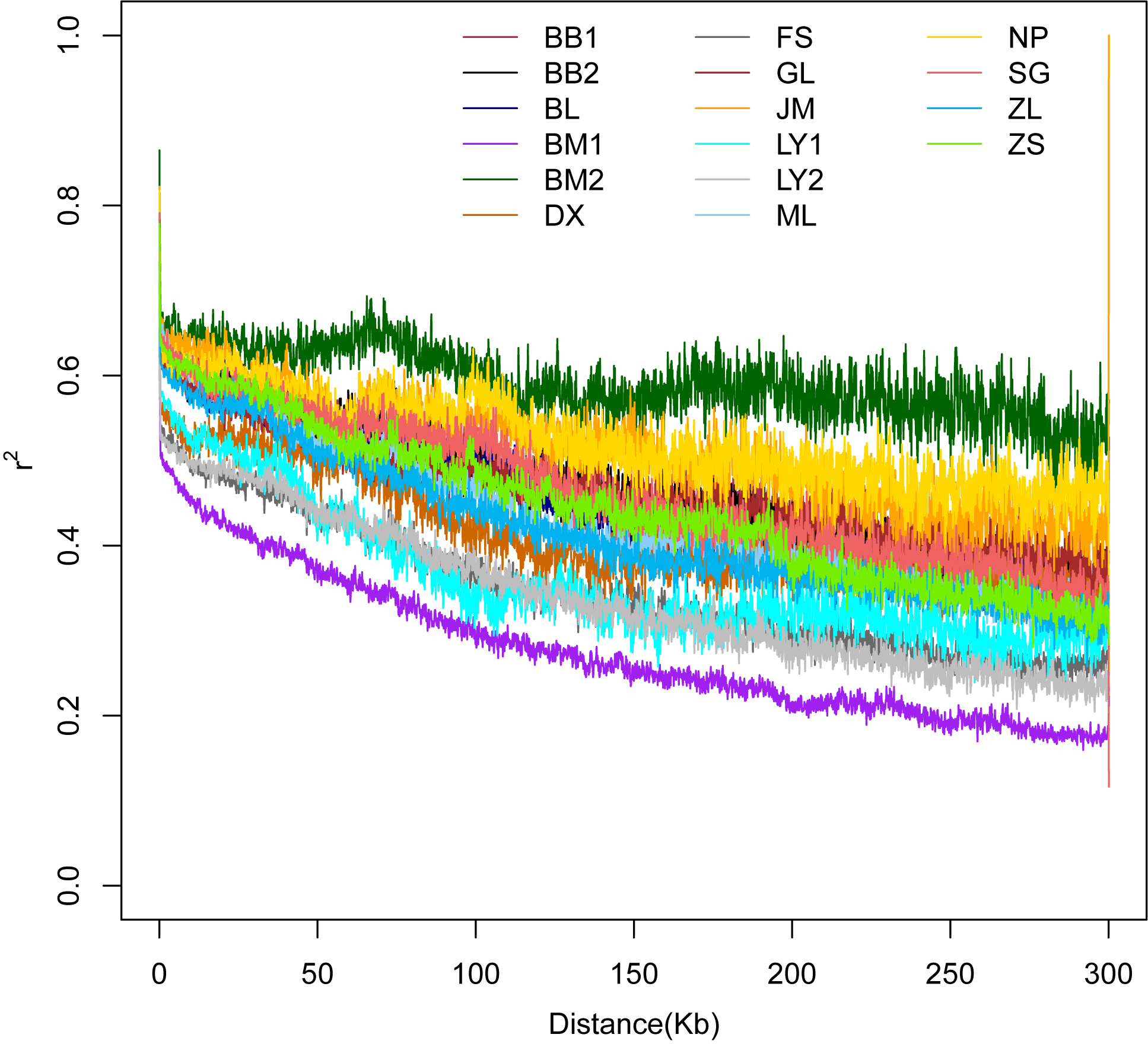
**

(b)

**
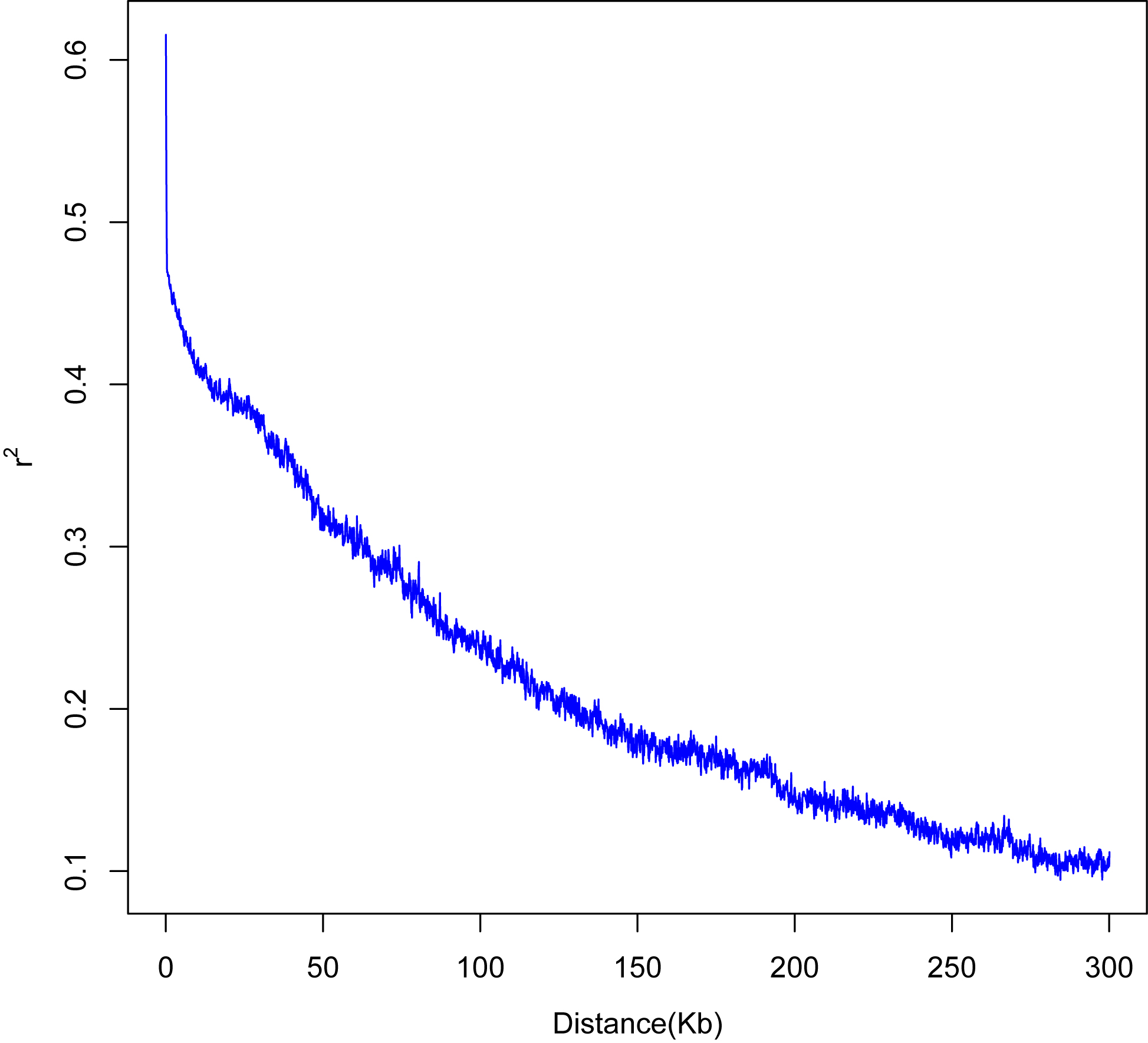
**

**Figure S3.** Genome-wide linkage disequilibrium (LD) decay of *Malania oleifera*. (a) Considering 16 populations separately and (b) as a whole.

(a) (b)


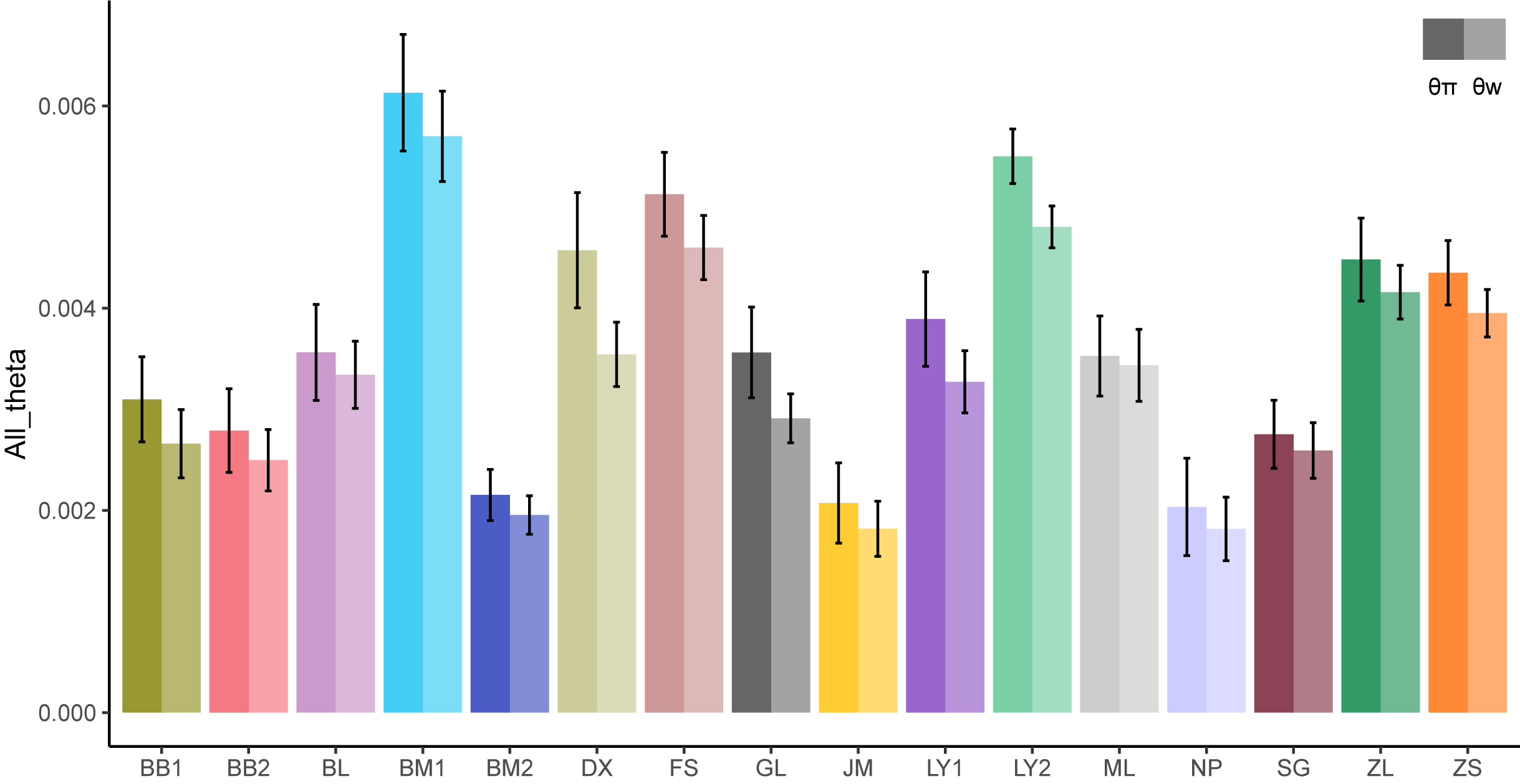

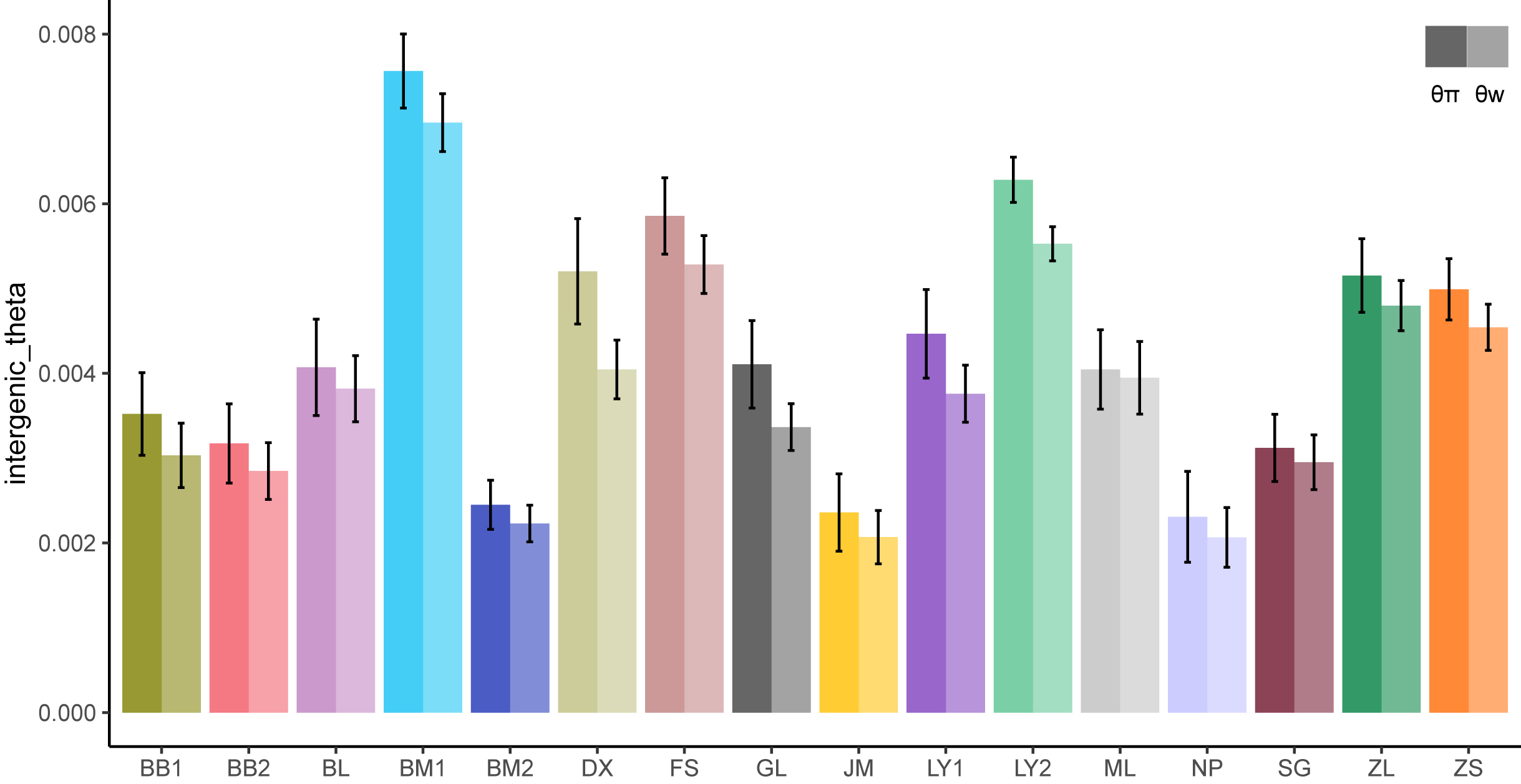


(c) (d)


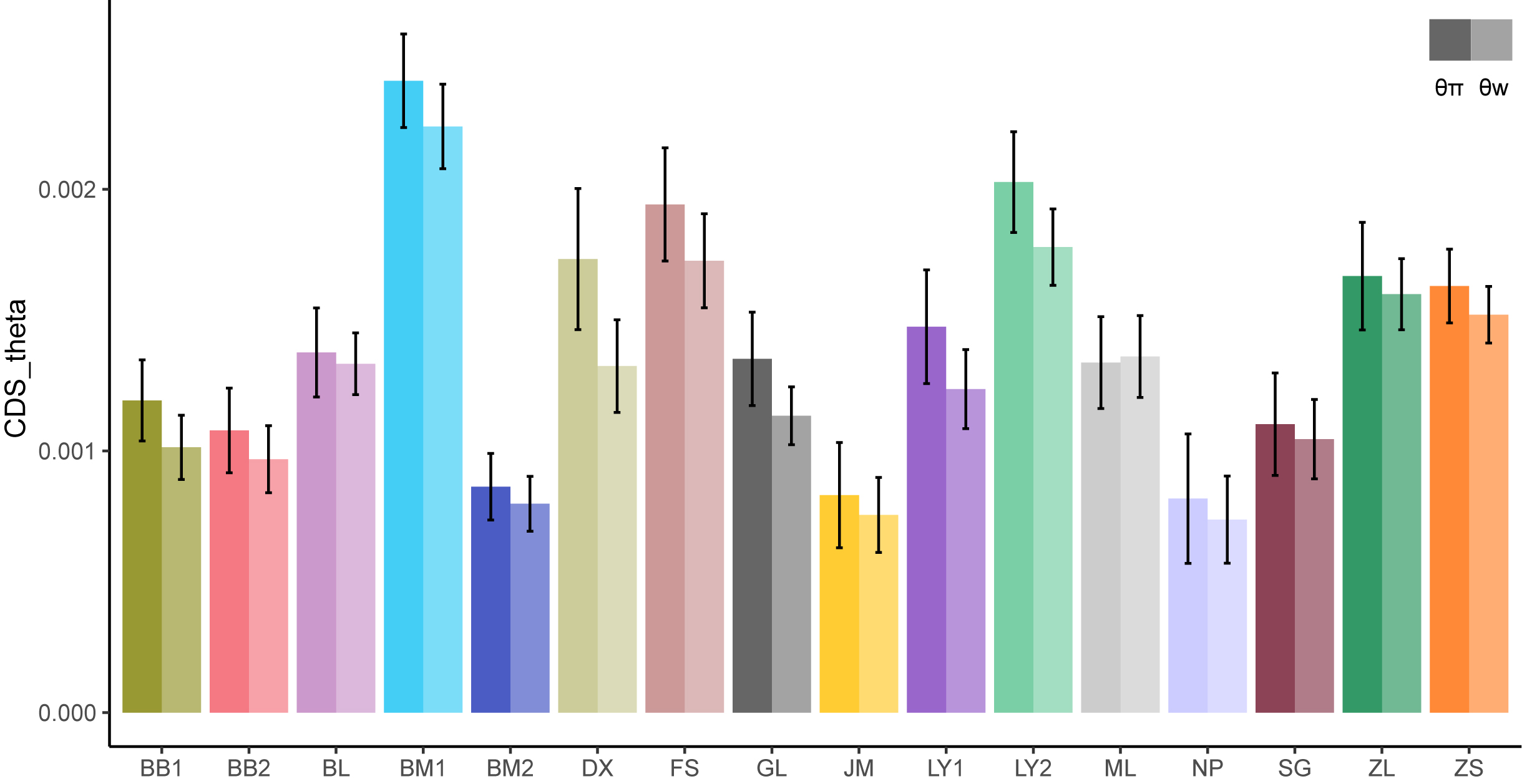

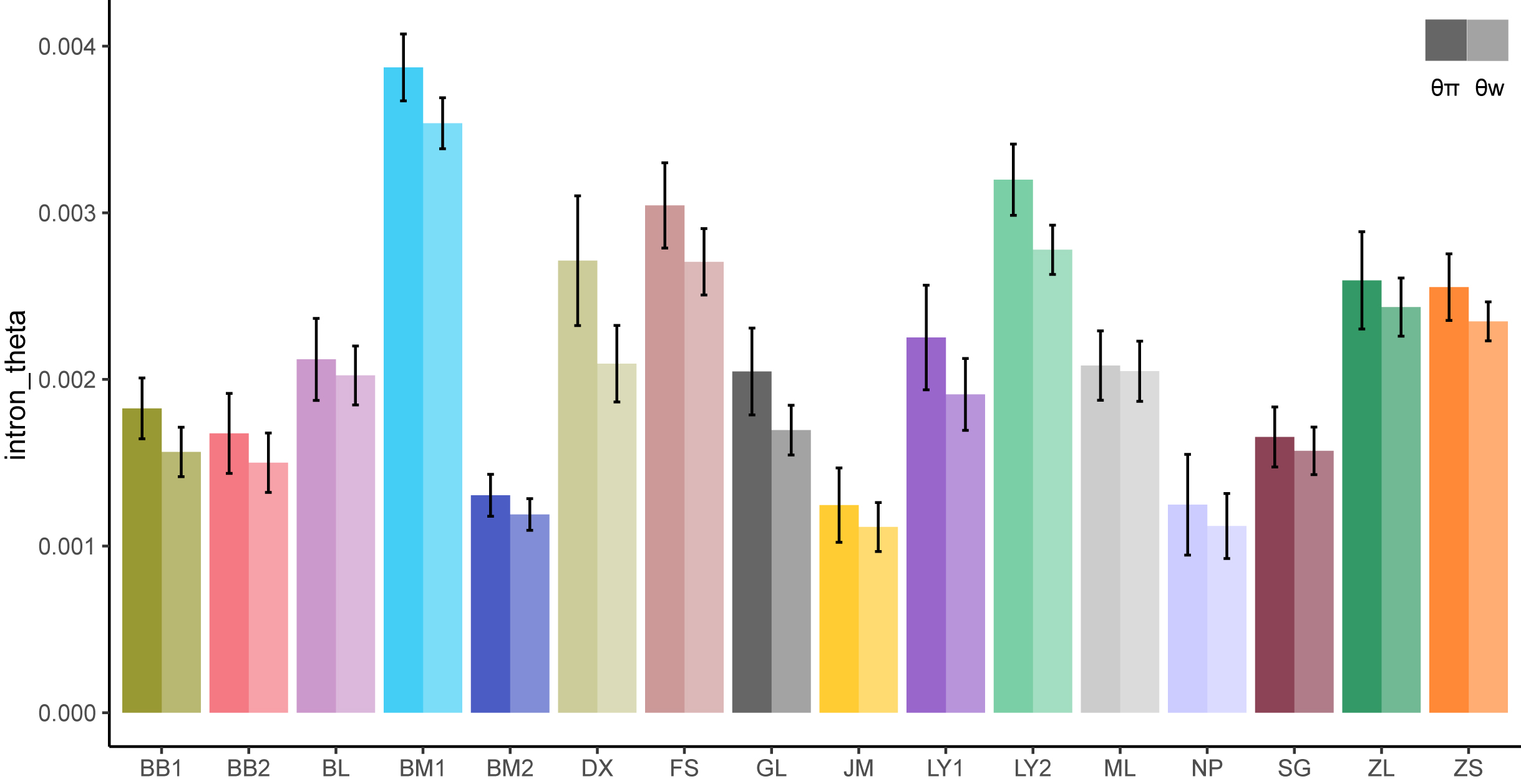


(e) (f)


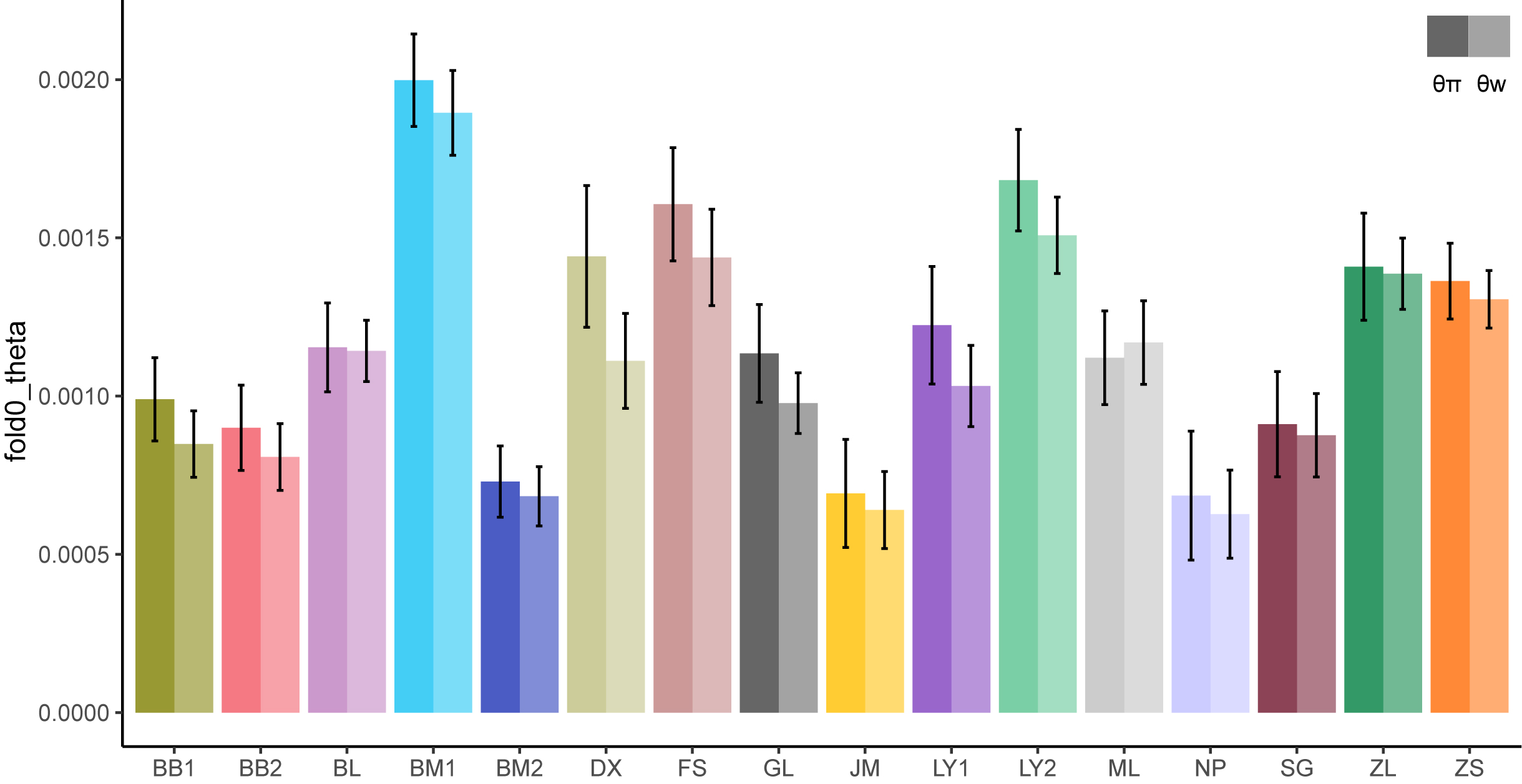

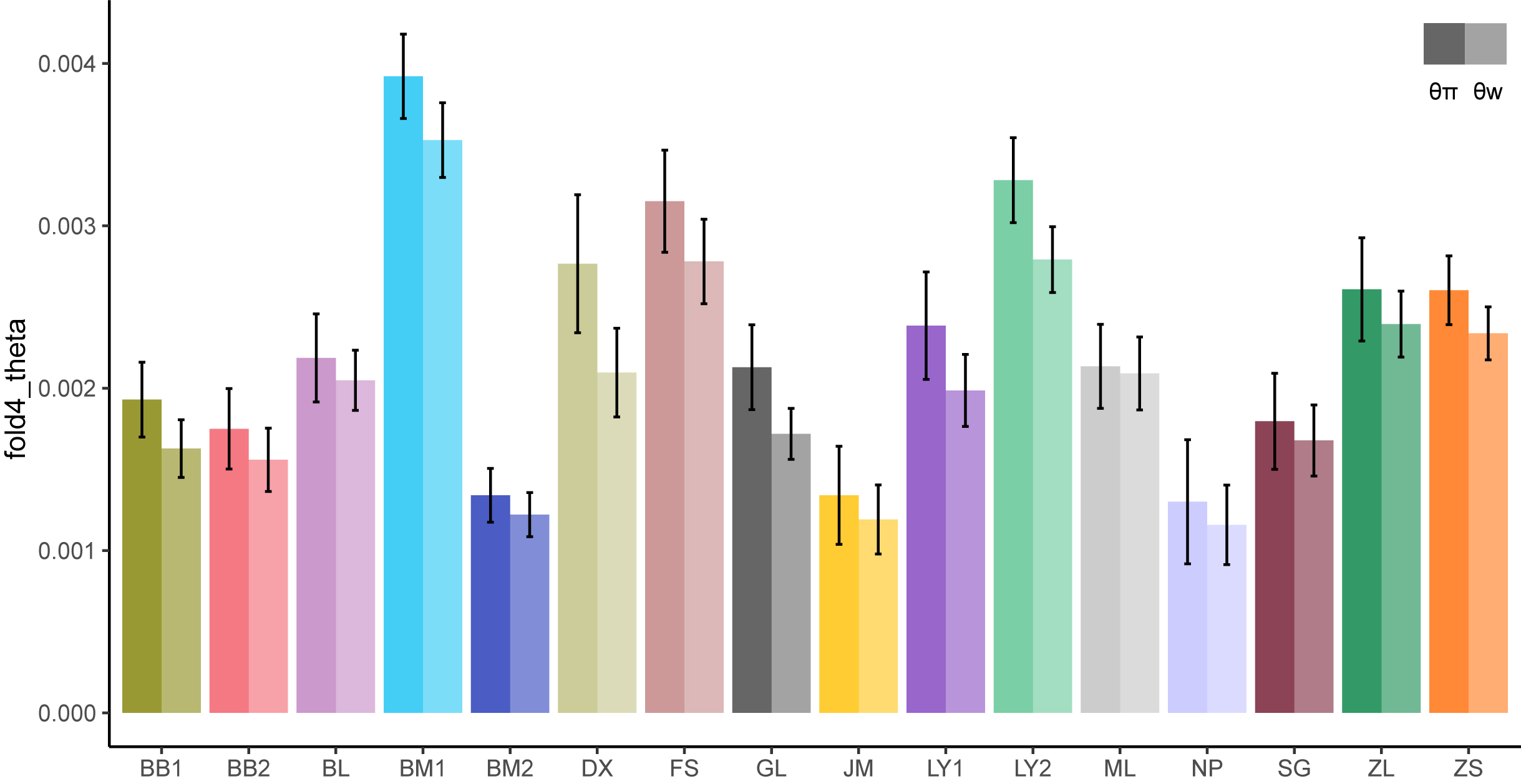


**Figure S4.** The comparison of mean θ_π_ and θ_W_ among 16 populations of *Malania oleifera* in whole genome (a), intergenic (b), CDS (c), intron (d), fold-0 (e) and fold-4 (f) regions.

(a)


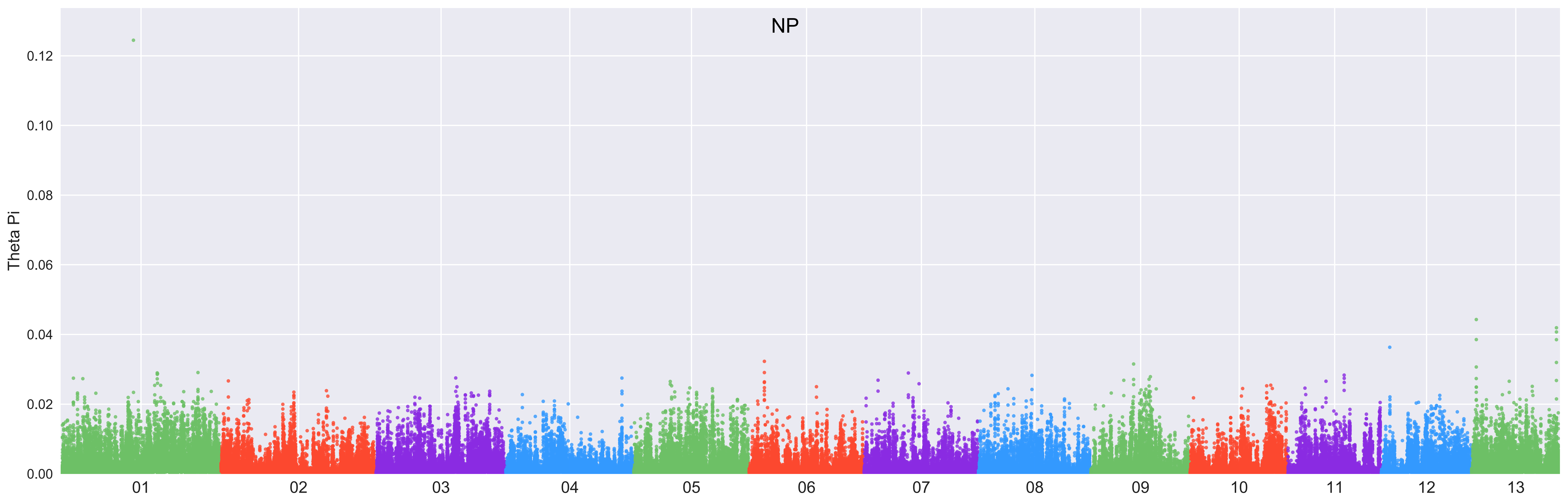


(b)


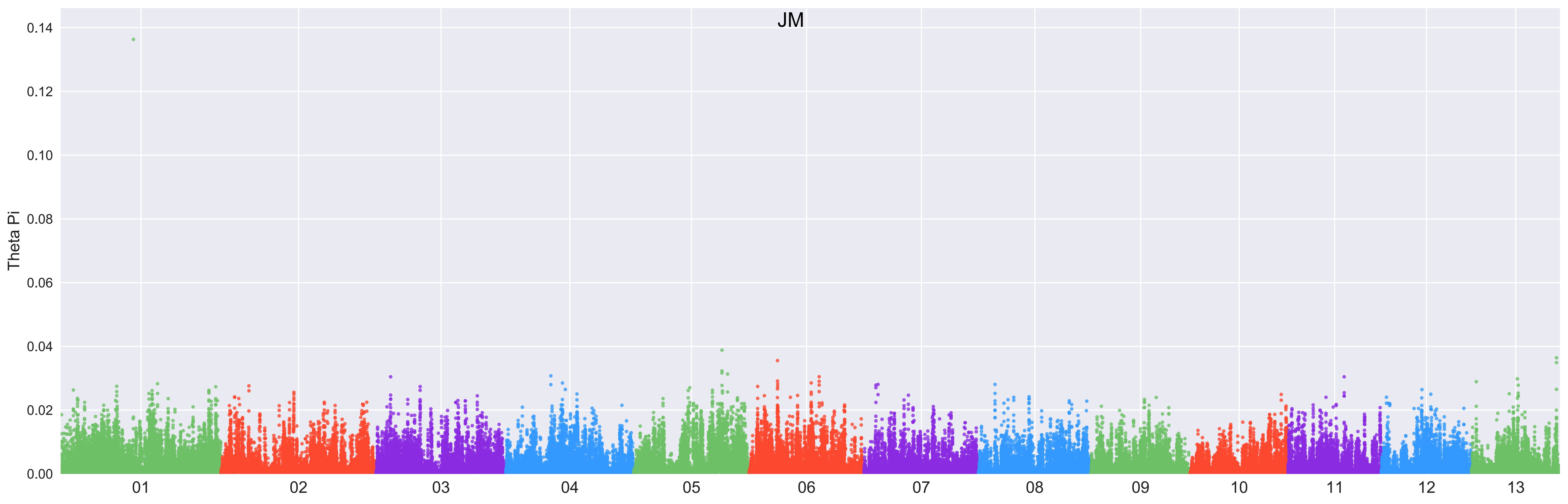


(c)


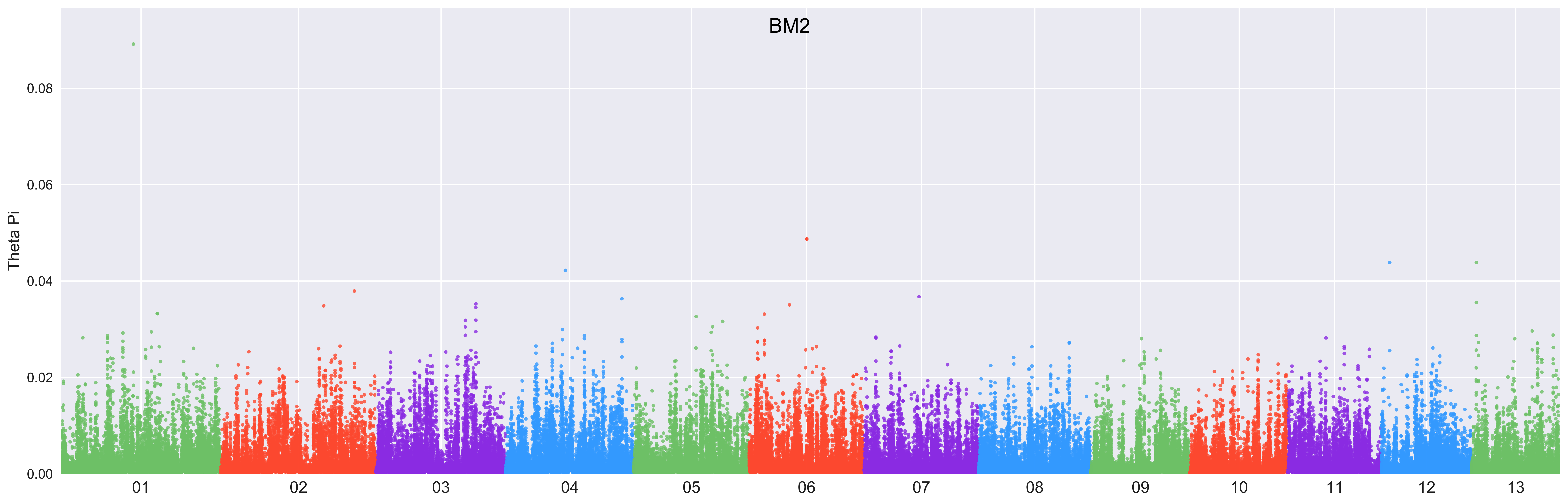


(d)


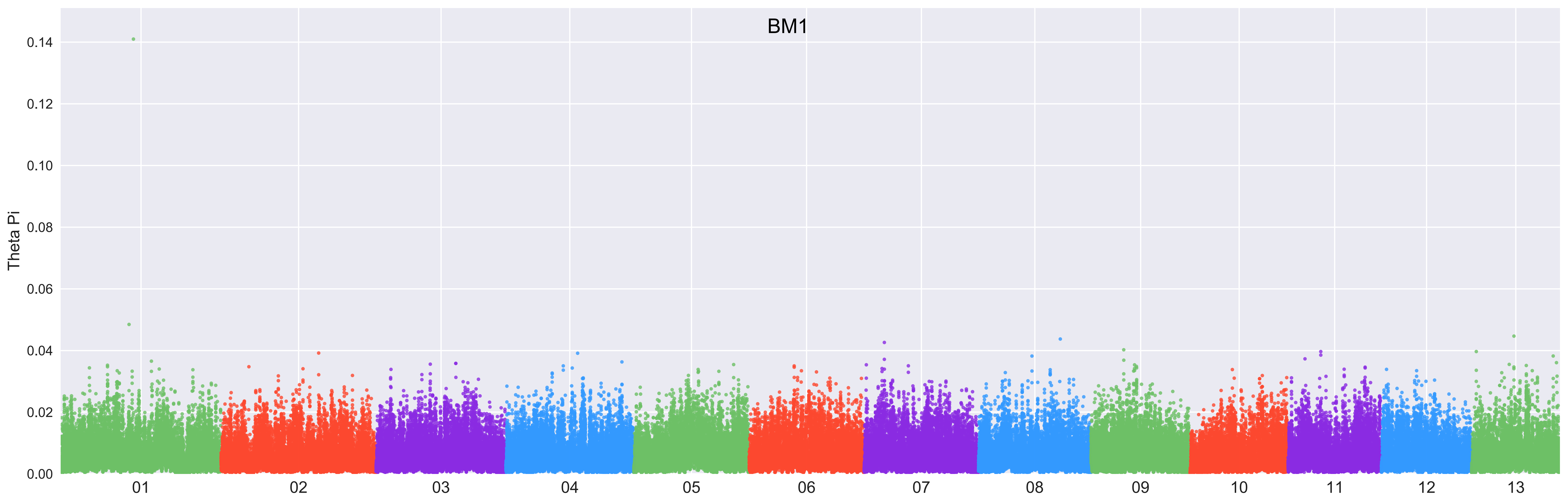


**Figure S5.** Distributions of nucleotide diversity (θπ) across the genome with (a)(b)(c) representing NP, JM and BM2 populations (lowest average θπ), respectively and (d) representing BM1 population (highest average θπ).

(a) (b)


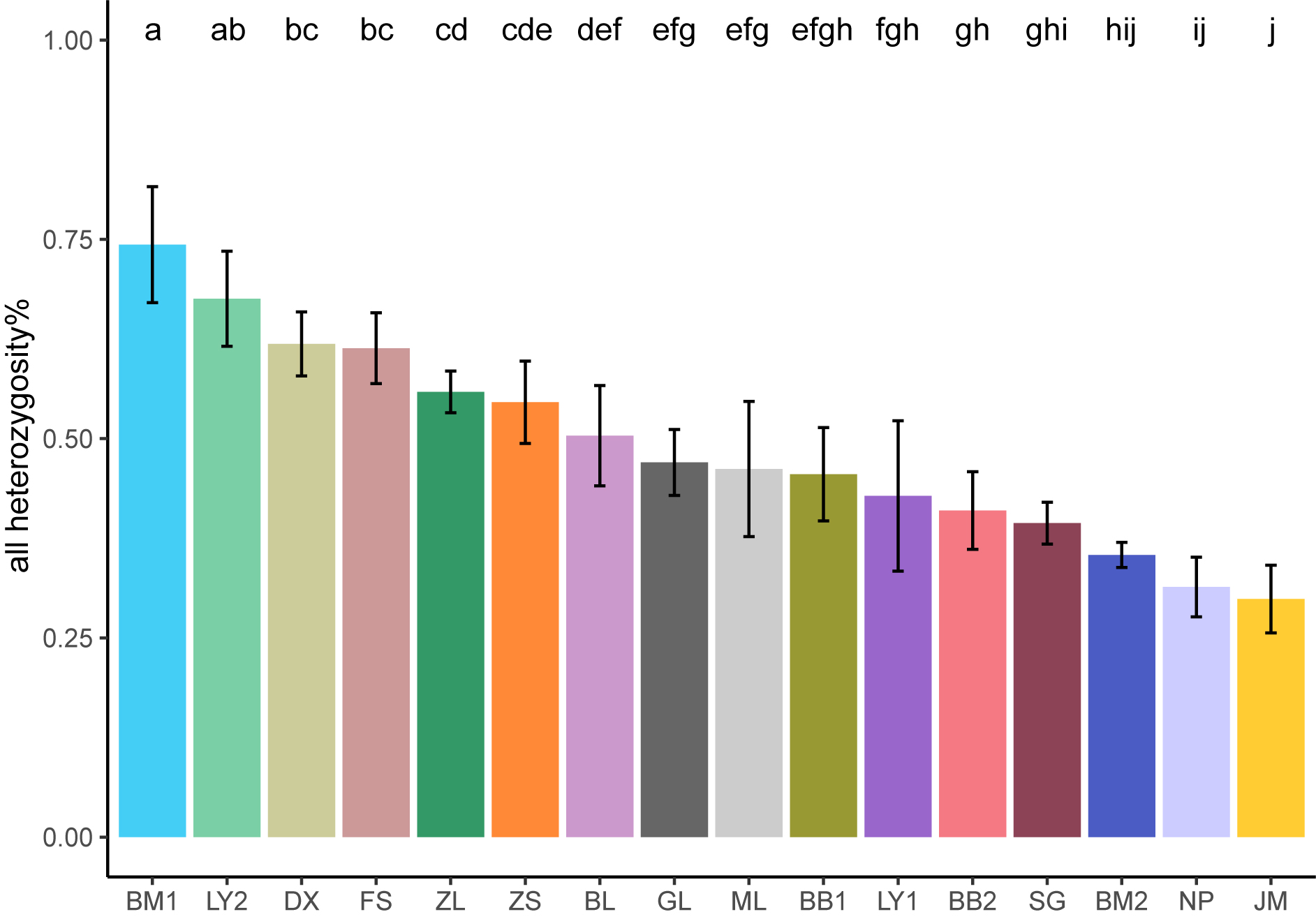

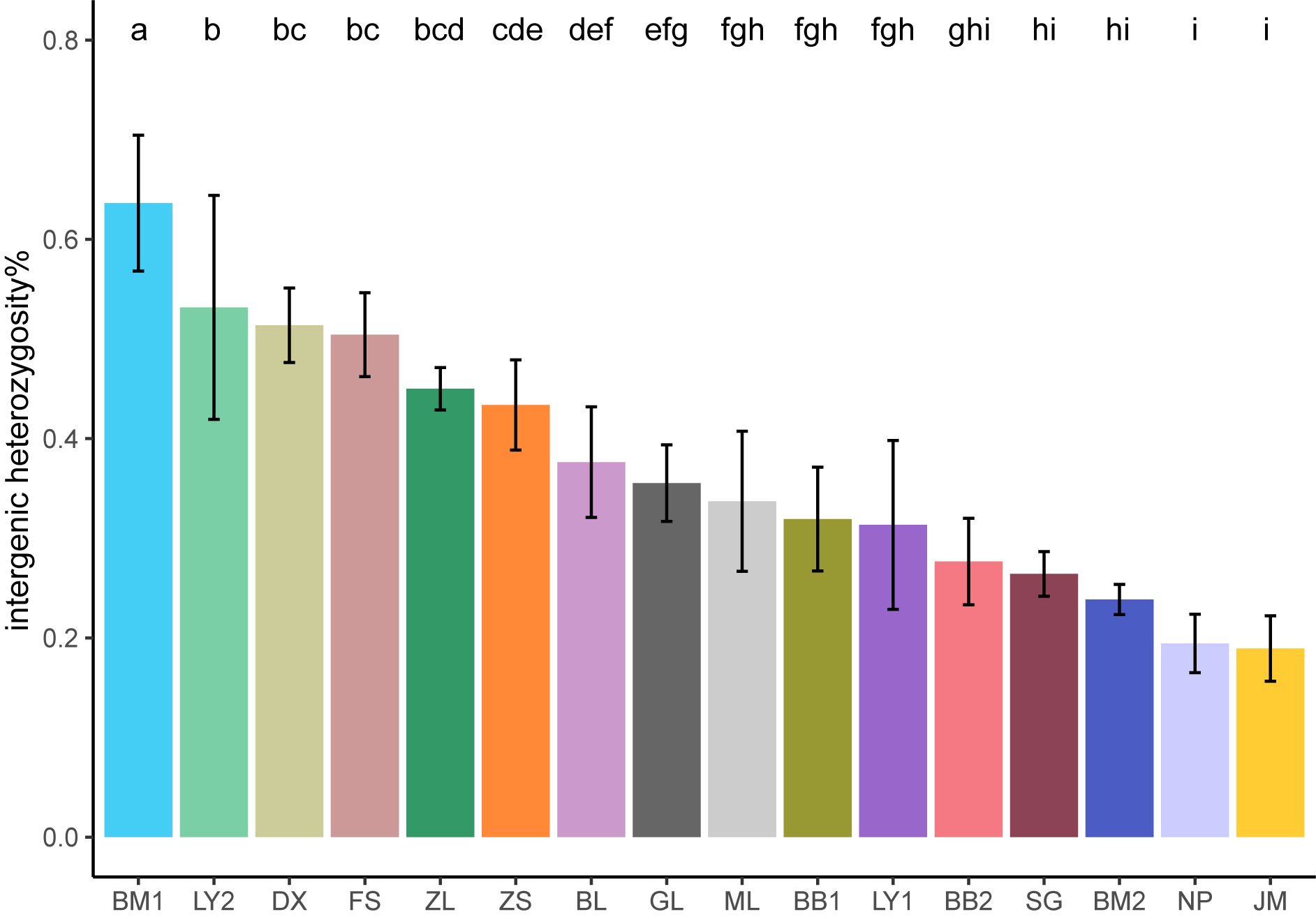


(c) (d)


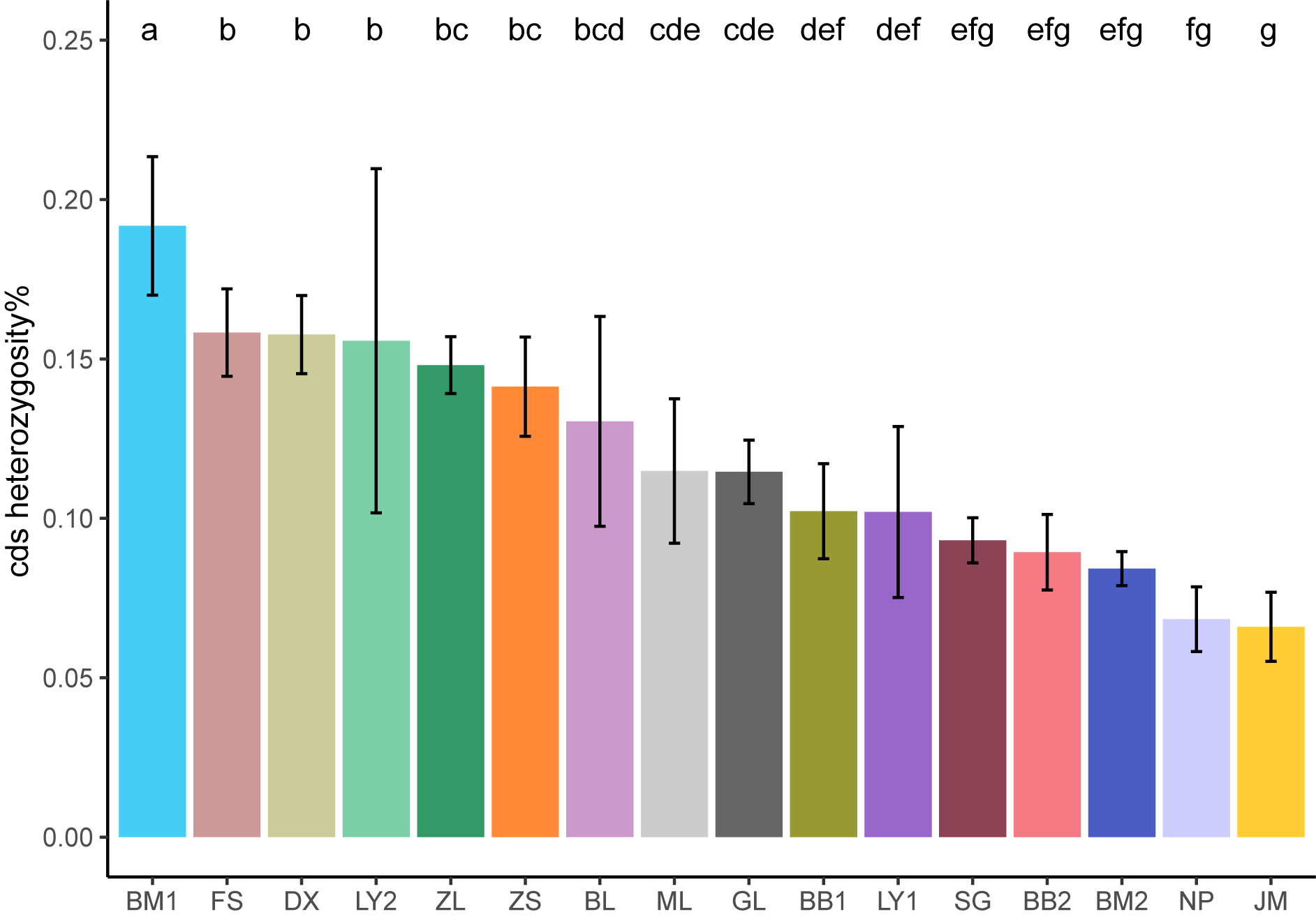

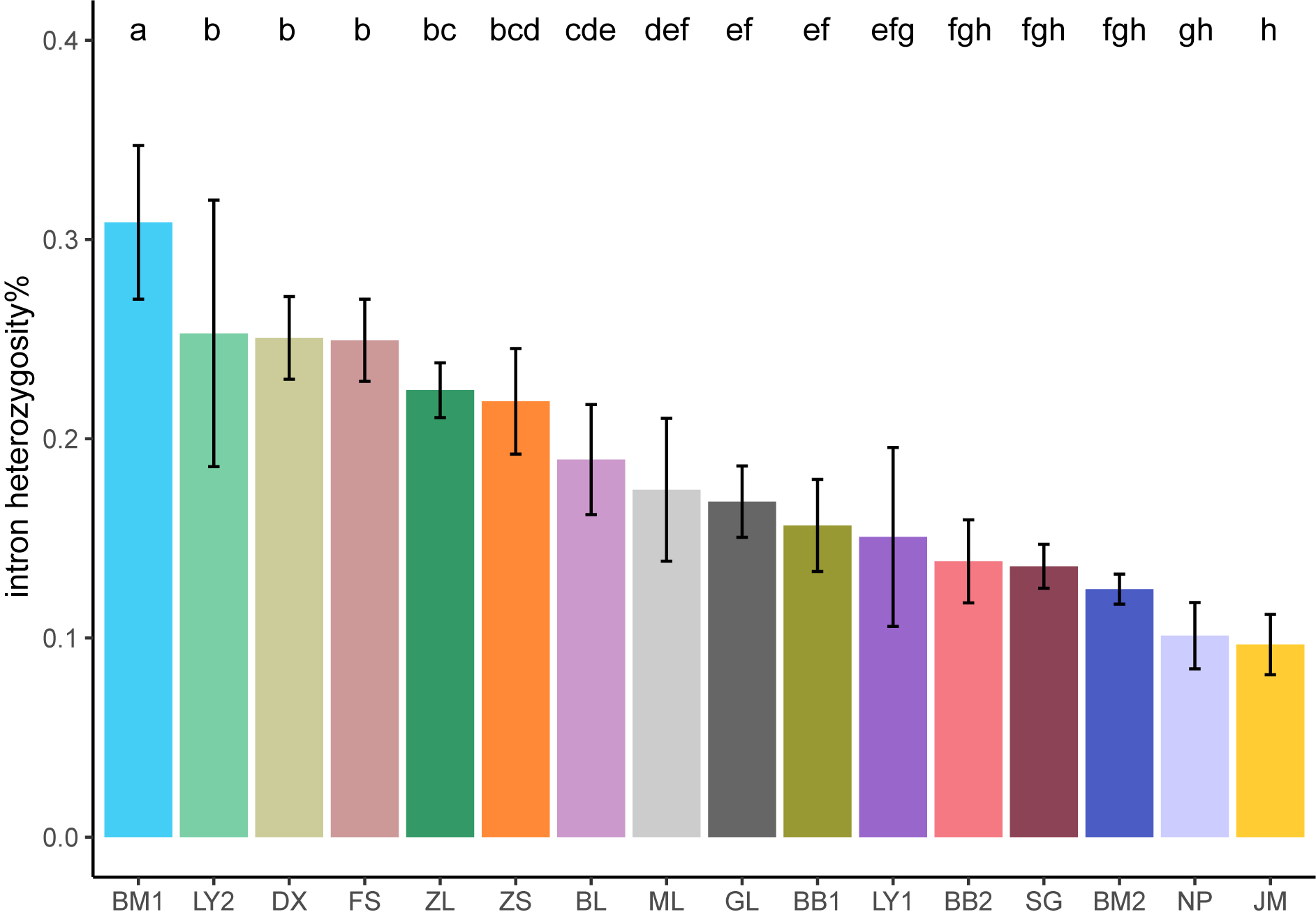


(e) (f)


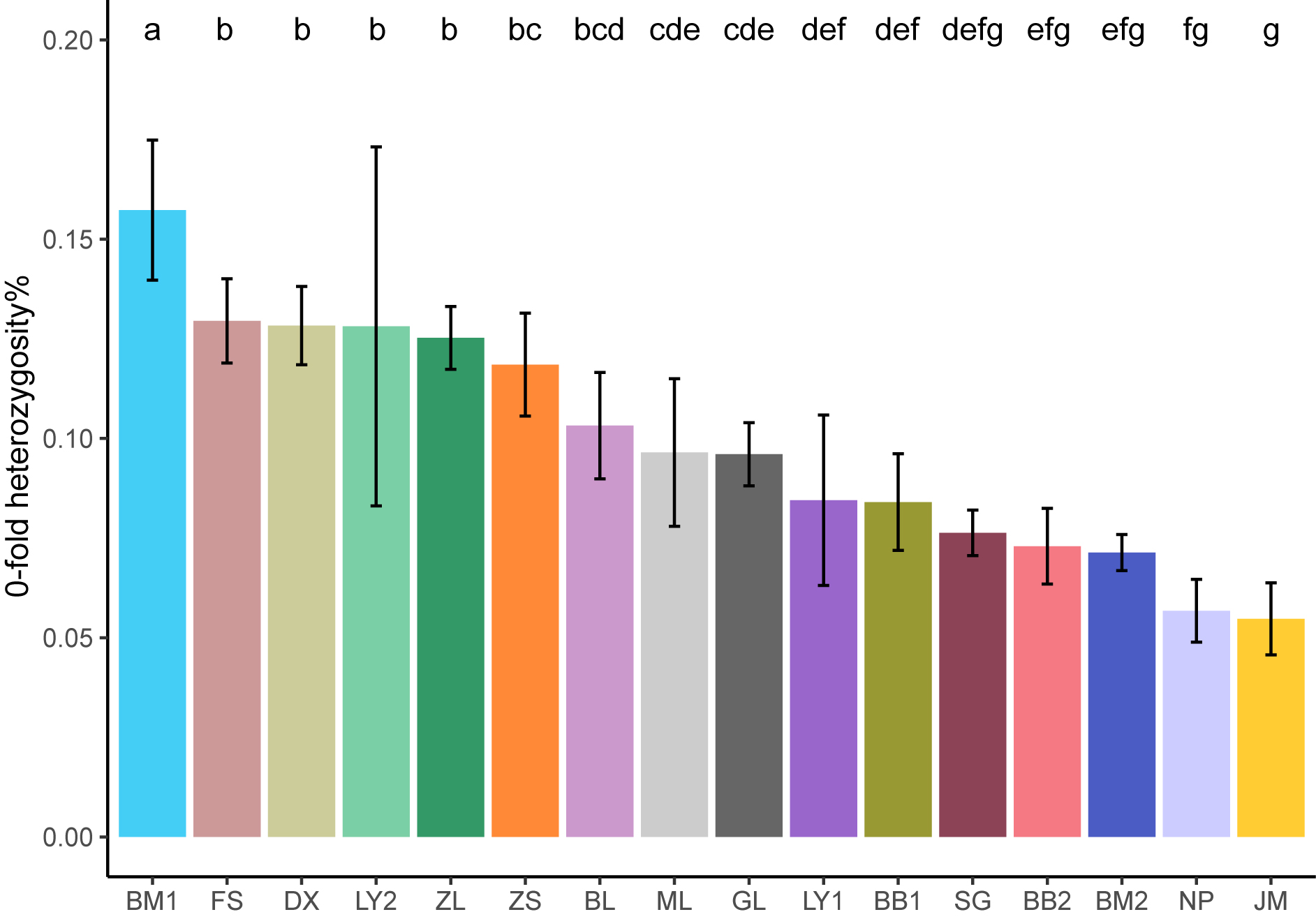

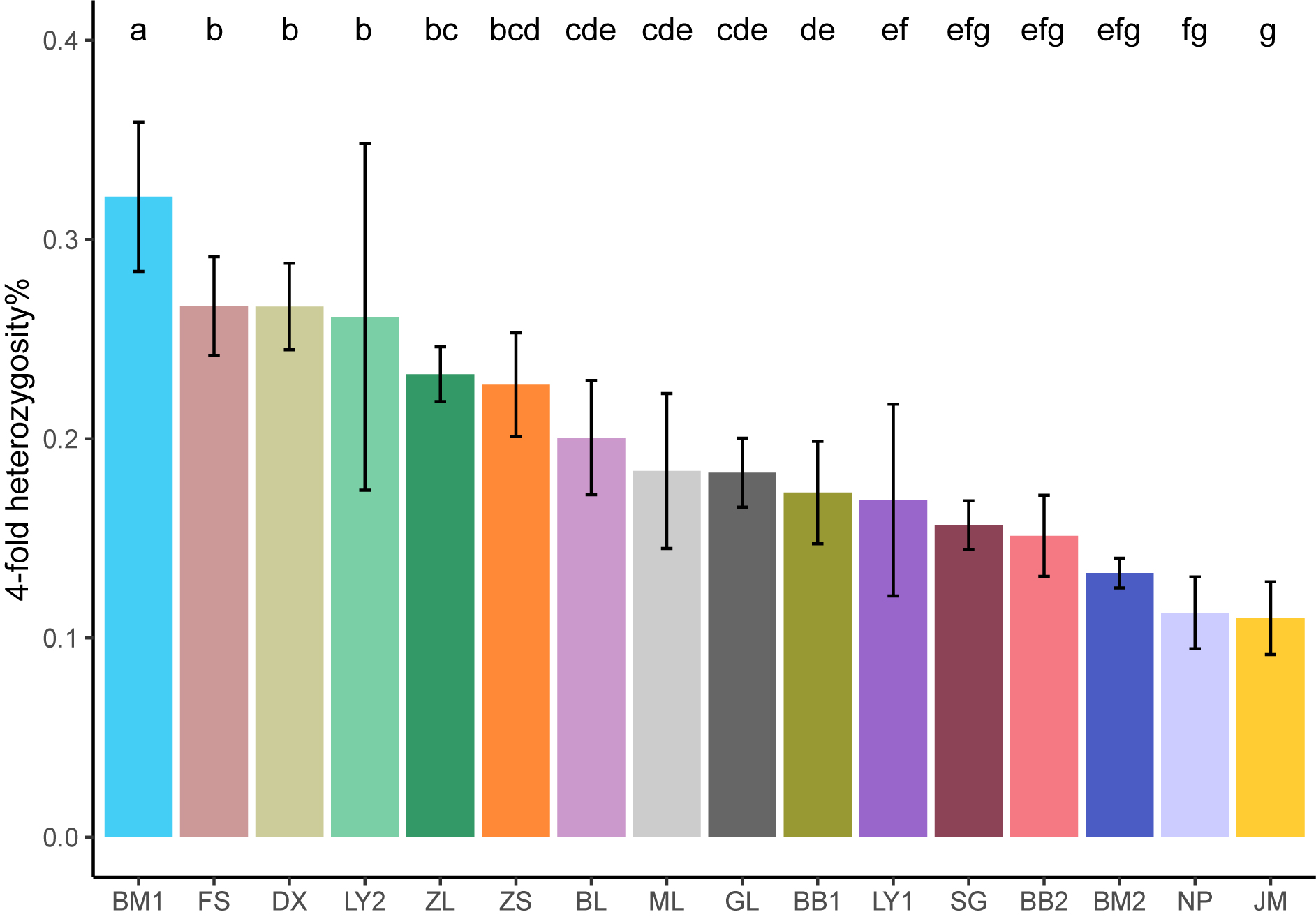


**Figure S6.** The comparison of heterozygosity rate among 16 populations of *Malania oleifera* in whole genome (a), intergenic (b), CDS (c), intron (d), fold-0 (e) and fold-4 (f) regions.

(a)


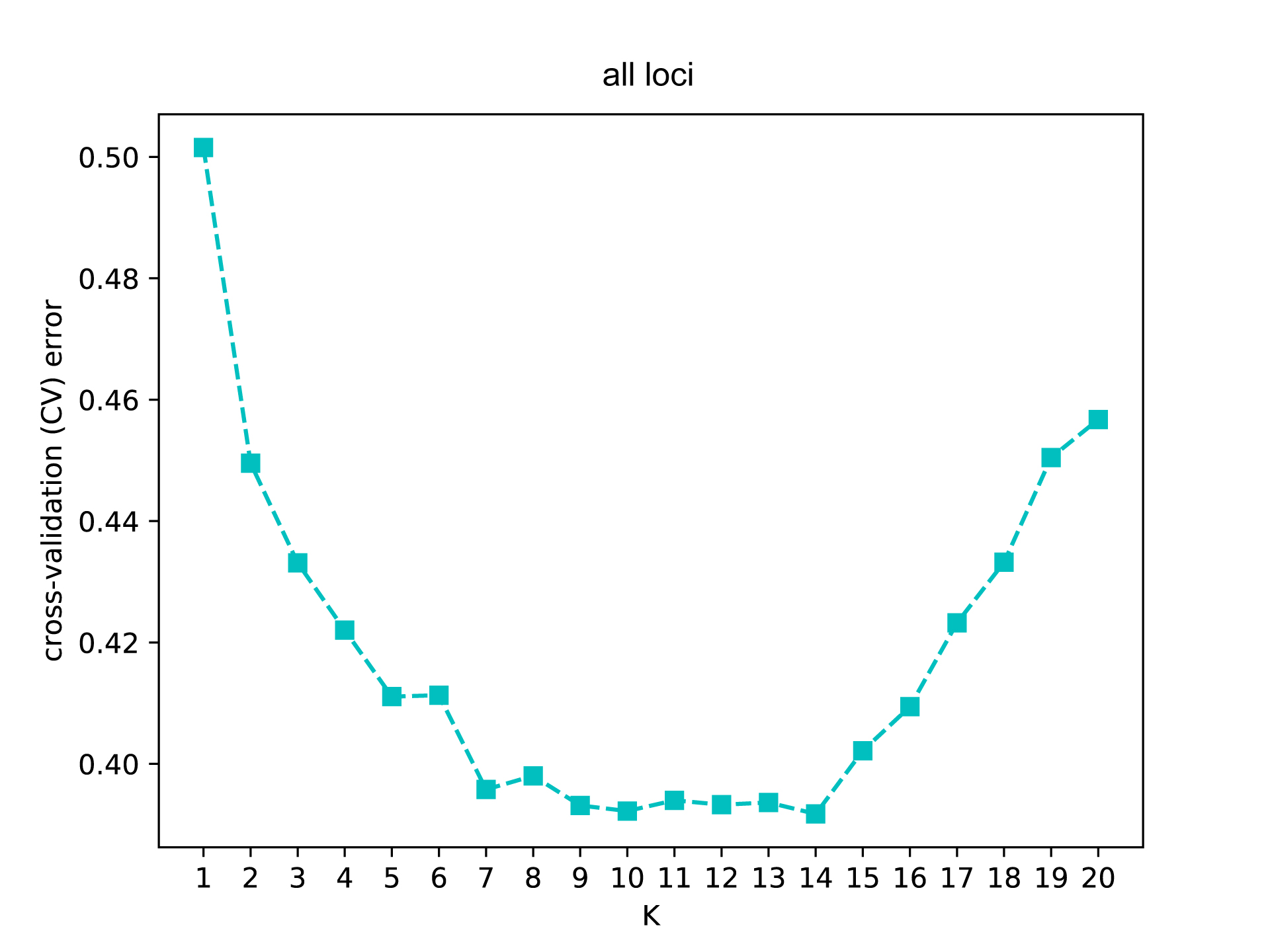


(b)


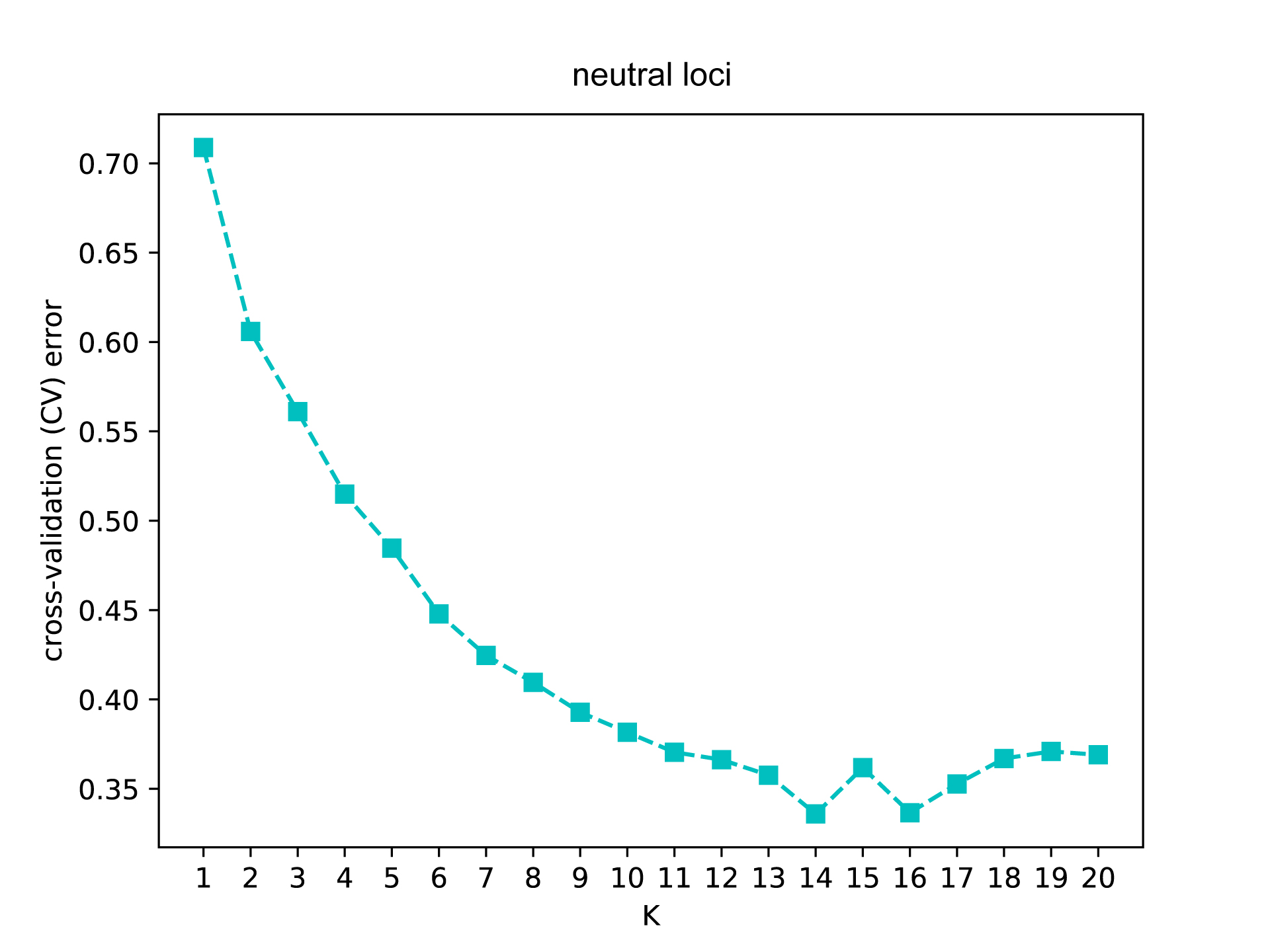


(c)


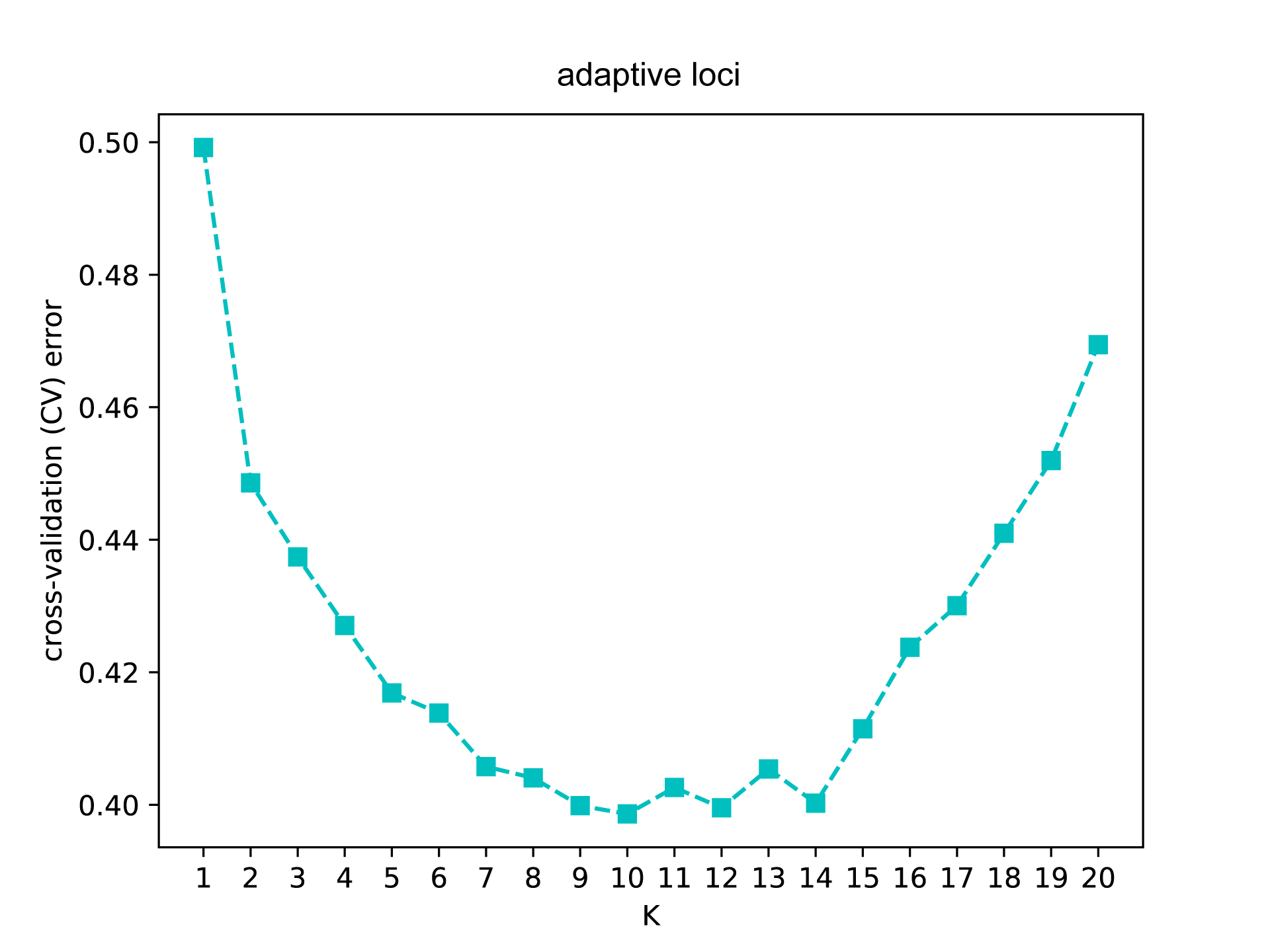


**Figure S7.** Cross-validation error curve based on all loci (a), neutral loci (b) and adaptive loci (c) for the 16 populations of *Malania oleifera* inferred by ADMIXTURE.

(a)


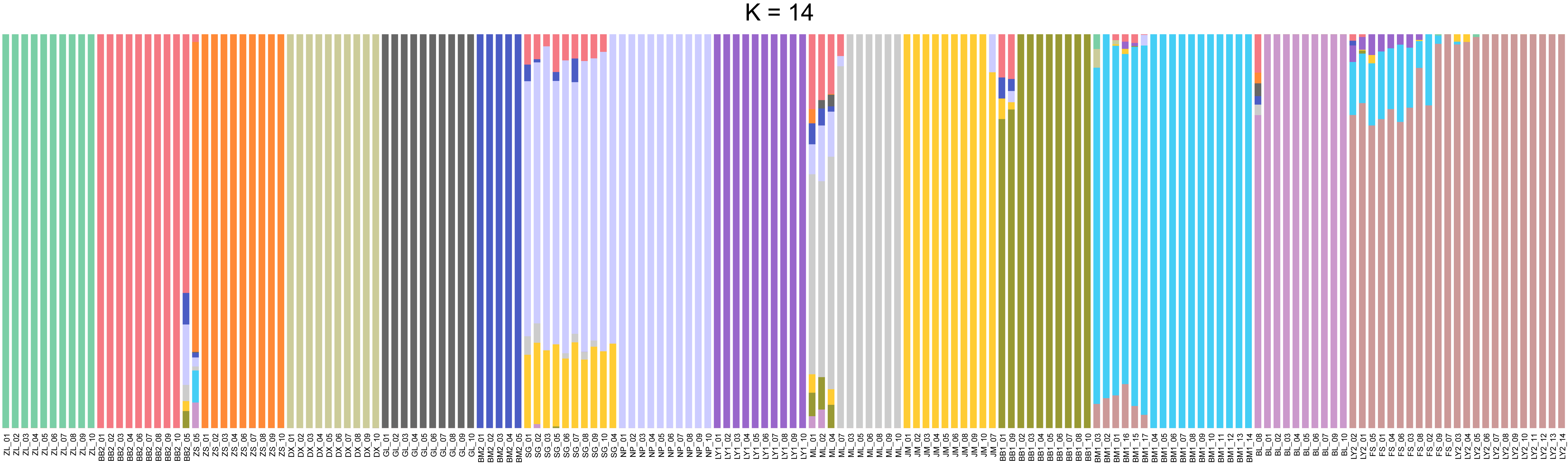


(b) (c)


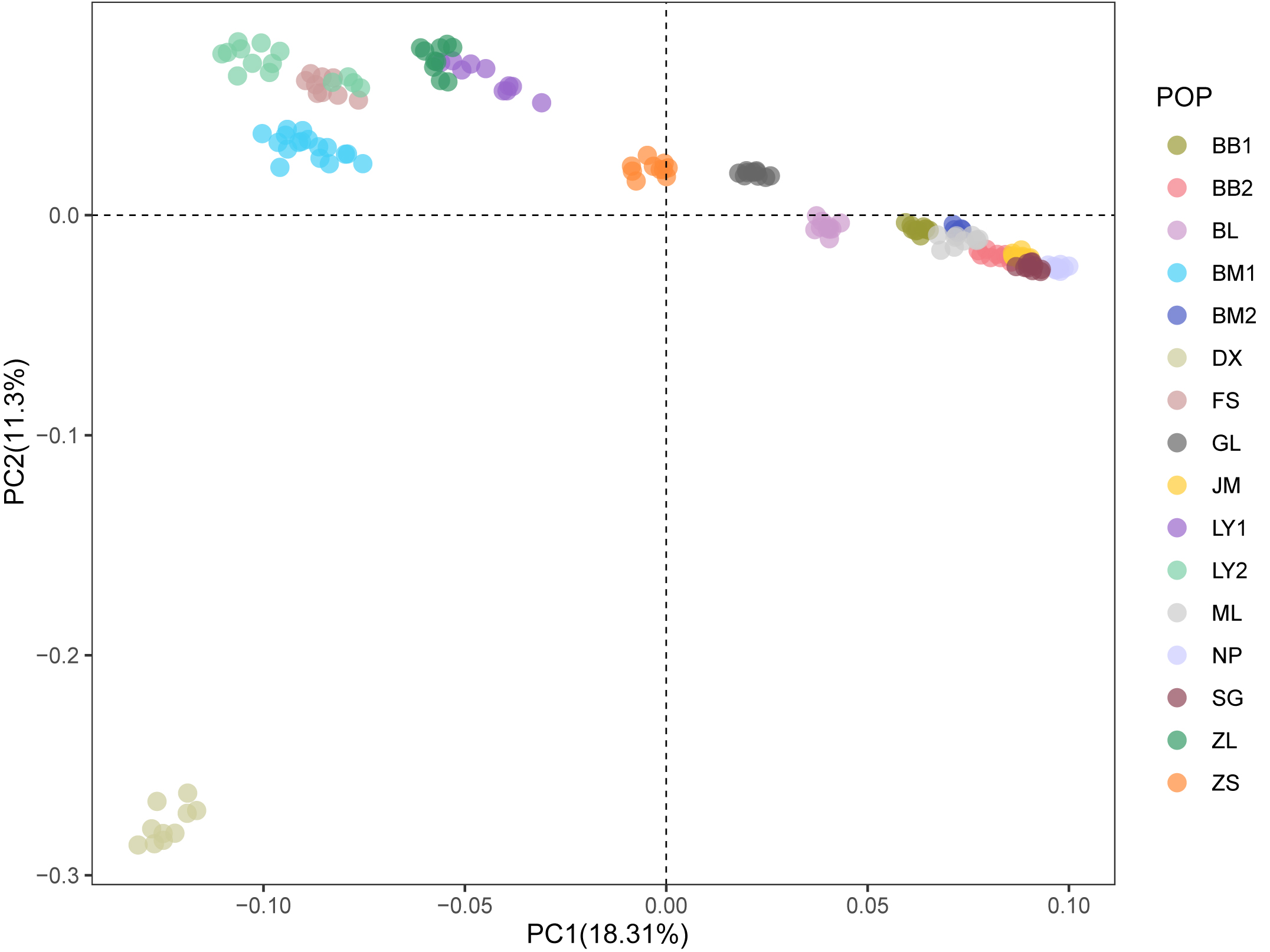

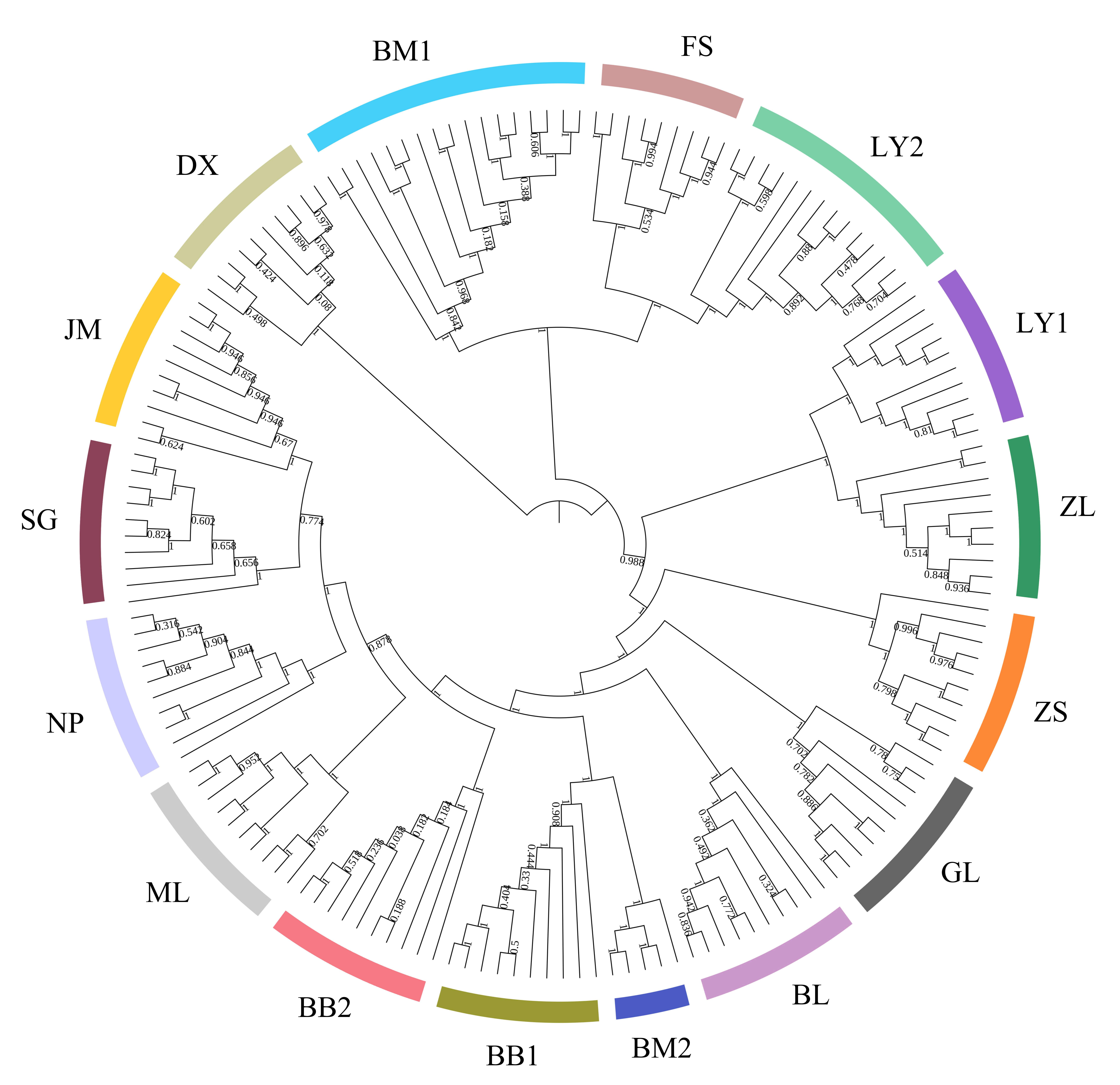


**Figure S8.** The inference of population structure (a), principal component analysis (b) and NJ tree (c) of *Malania oleifera* based on all loci.

(a)





(b) (c)


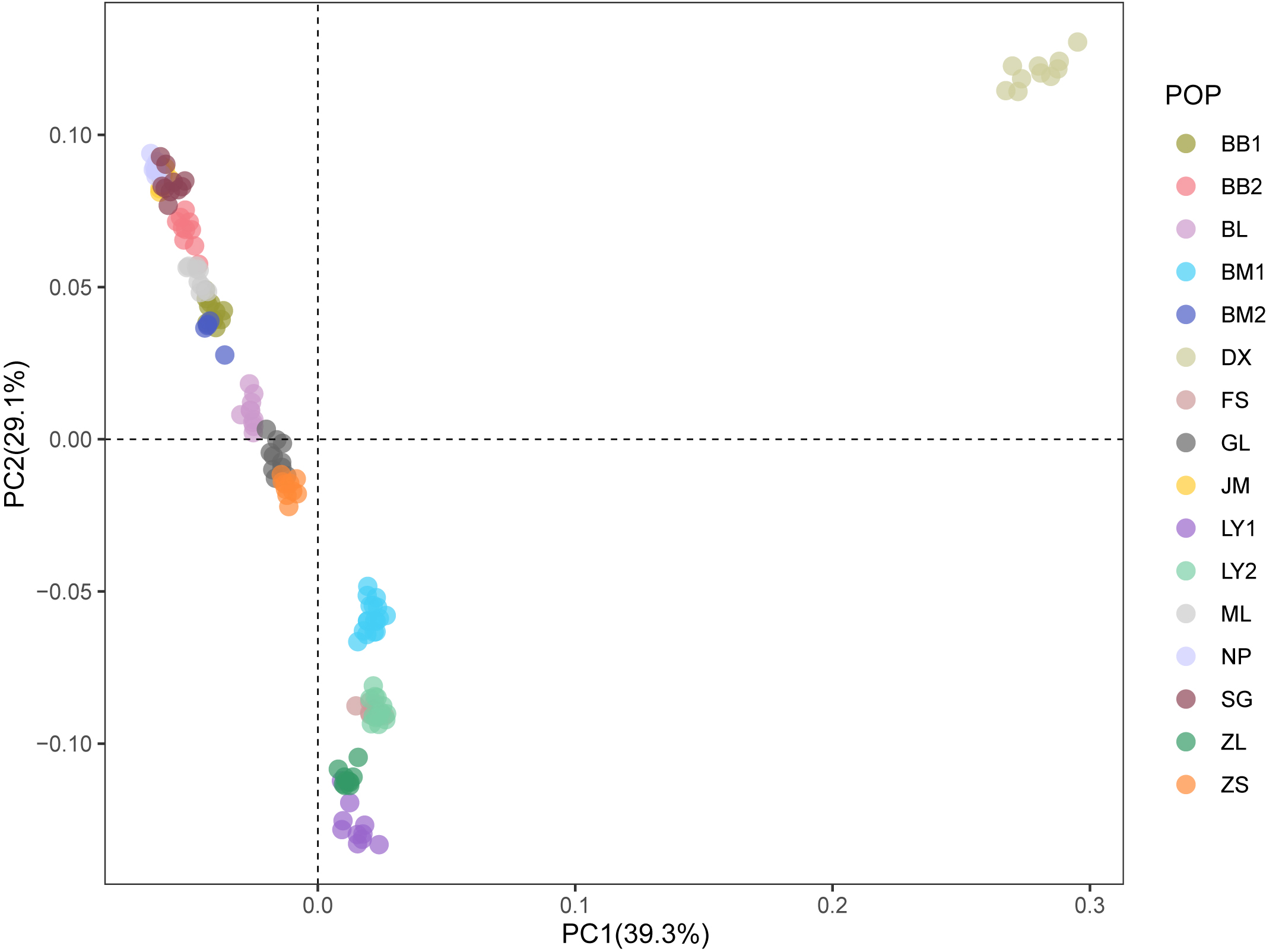

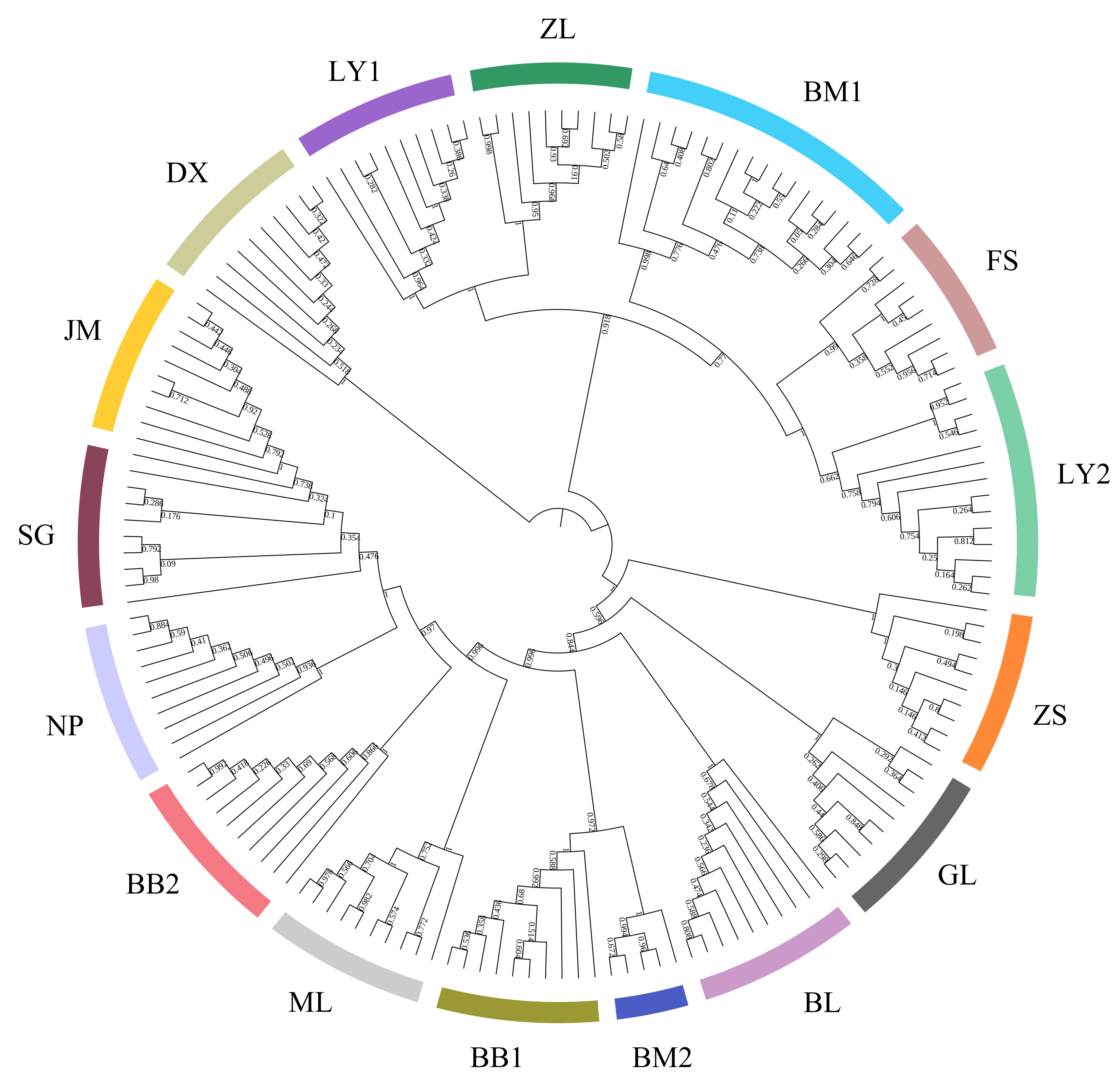


**Figure S9.** The inference of population structure (a), principal component analysis (b) and NJ tree (c) of *Malania oleifera* based on neutral loci.


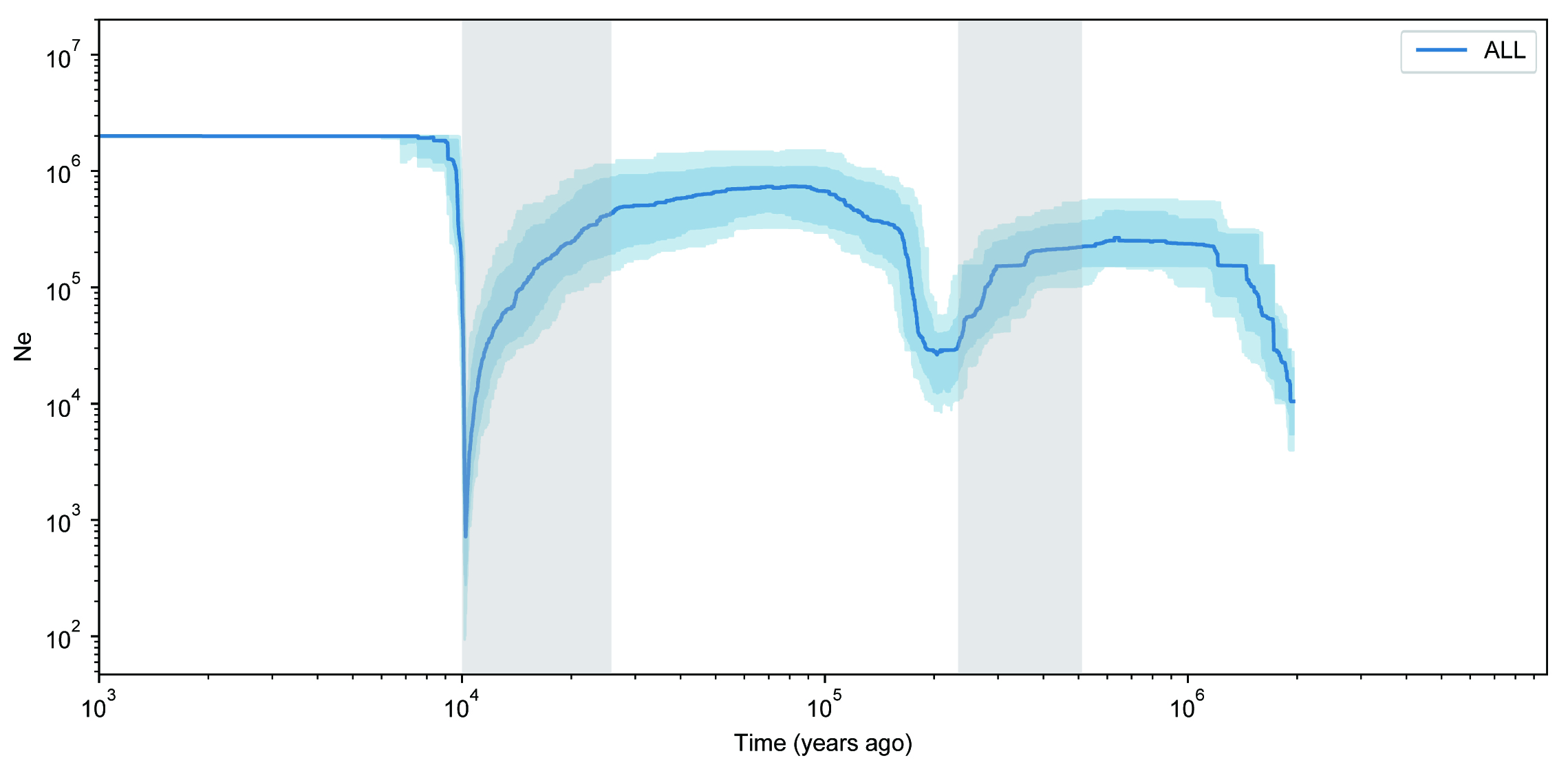


**Figure S10.** Demographic history of *Malania oleifera* inferred by Stairway Plot v.2 based on folded SFS.


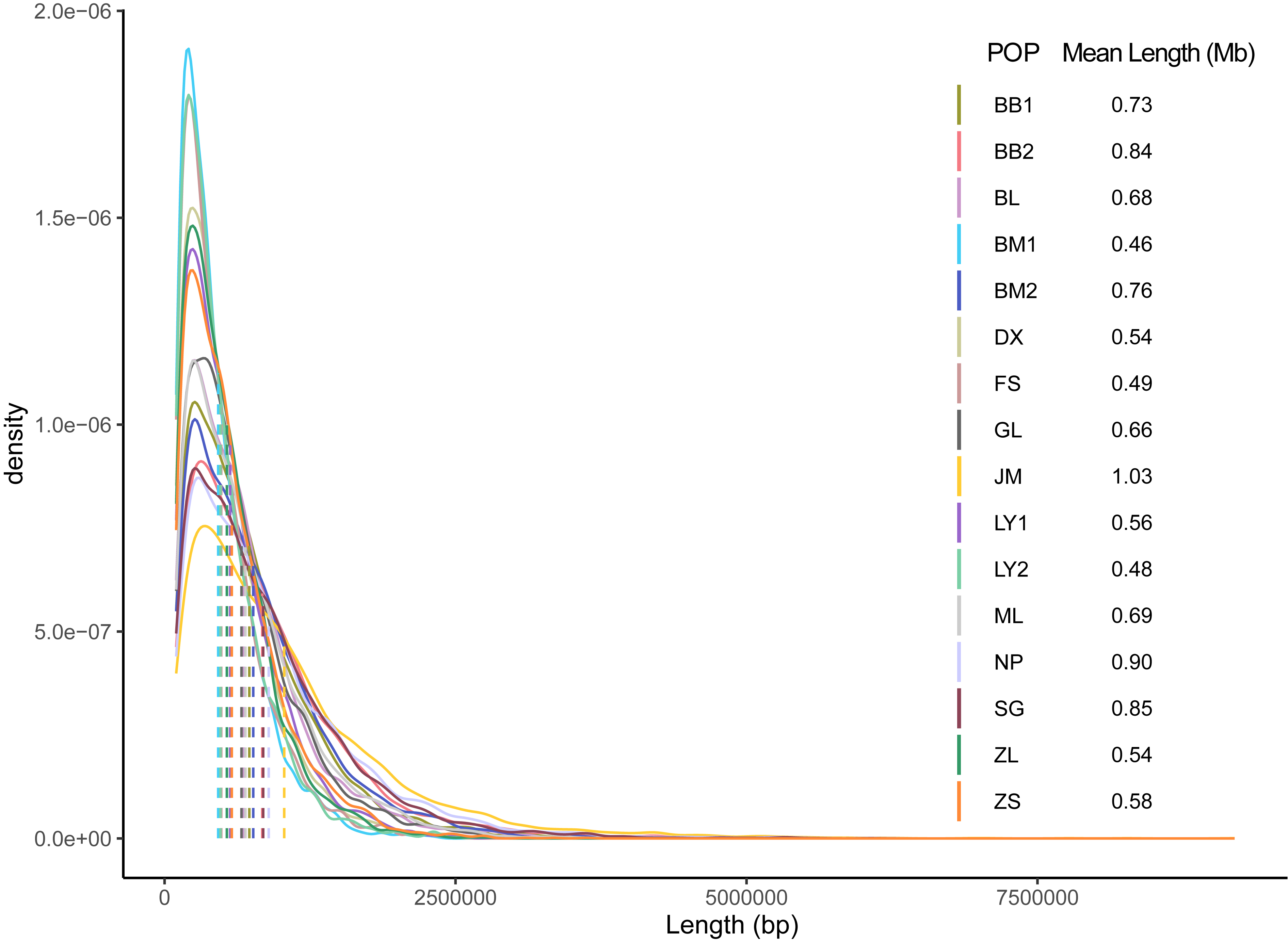


**Figure S11.** Runs of homozygosity (ROH) frequency differences among 16 populations of *Malania oleifera.* The dashed lines correspond to the mean ROH lengths for each population.


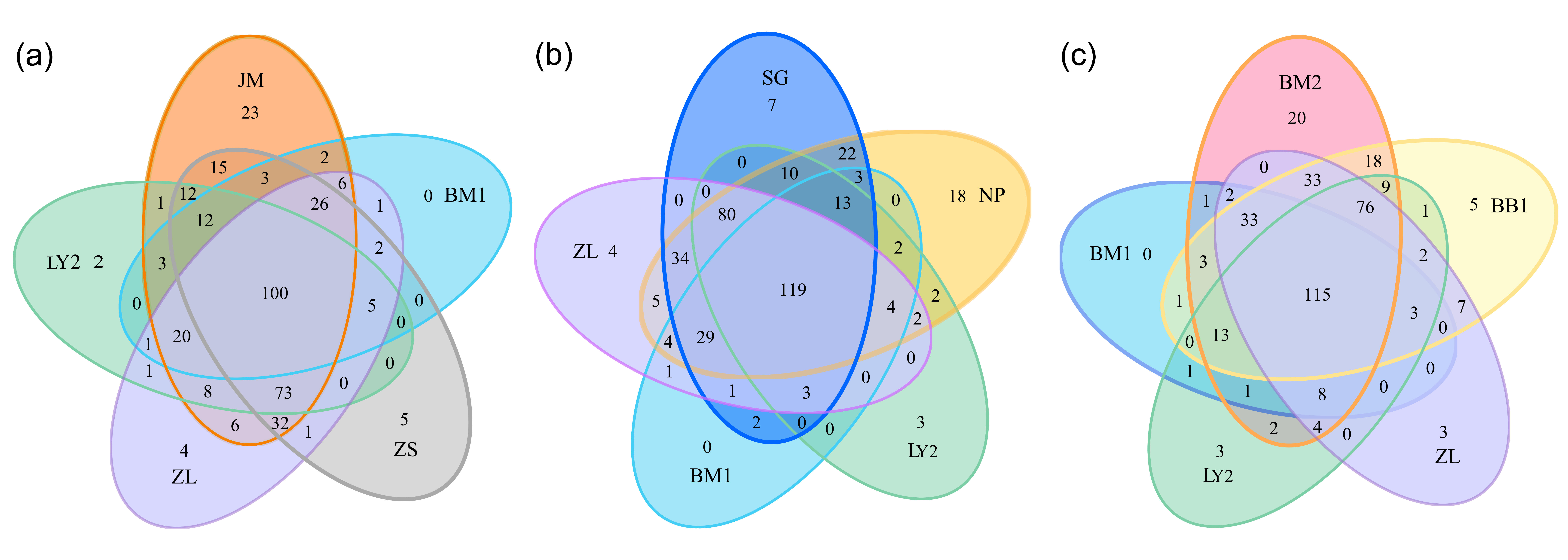


**Figure S12.** The Venn diagrams of share and private homozygous deleterious mutations of JM (a), SG (b) and BM2 (c) populations with candidate pollen donors.

(a) (b)

**
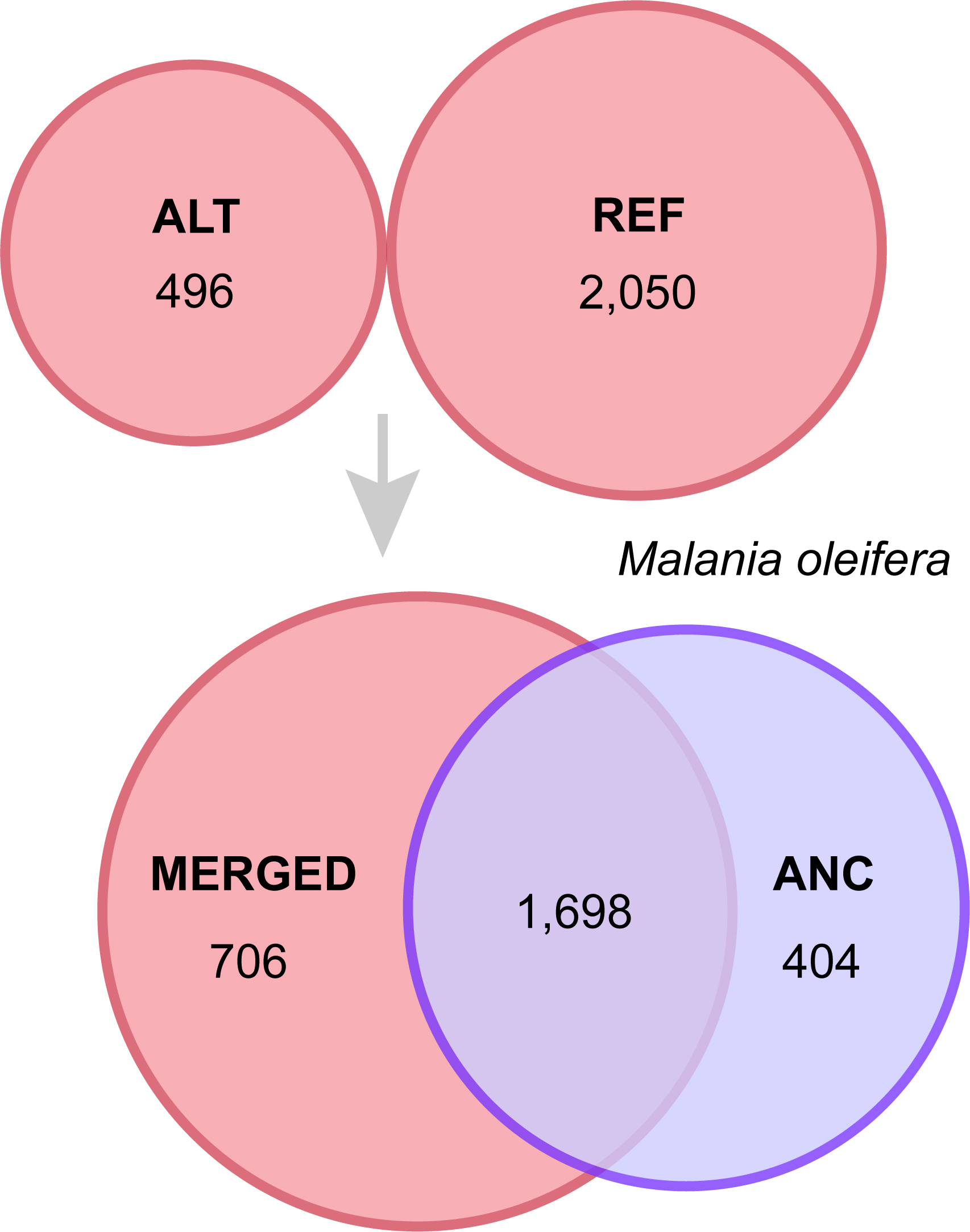

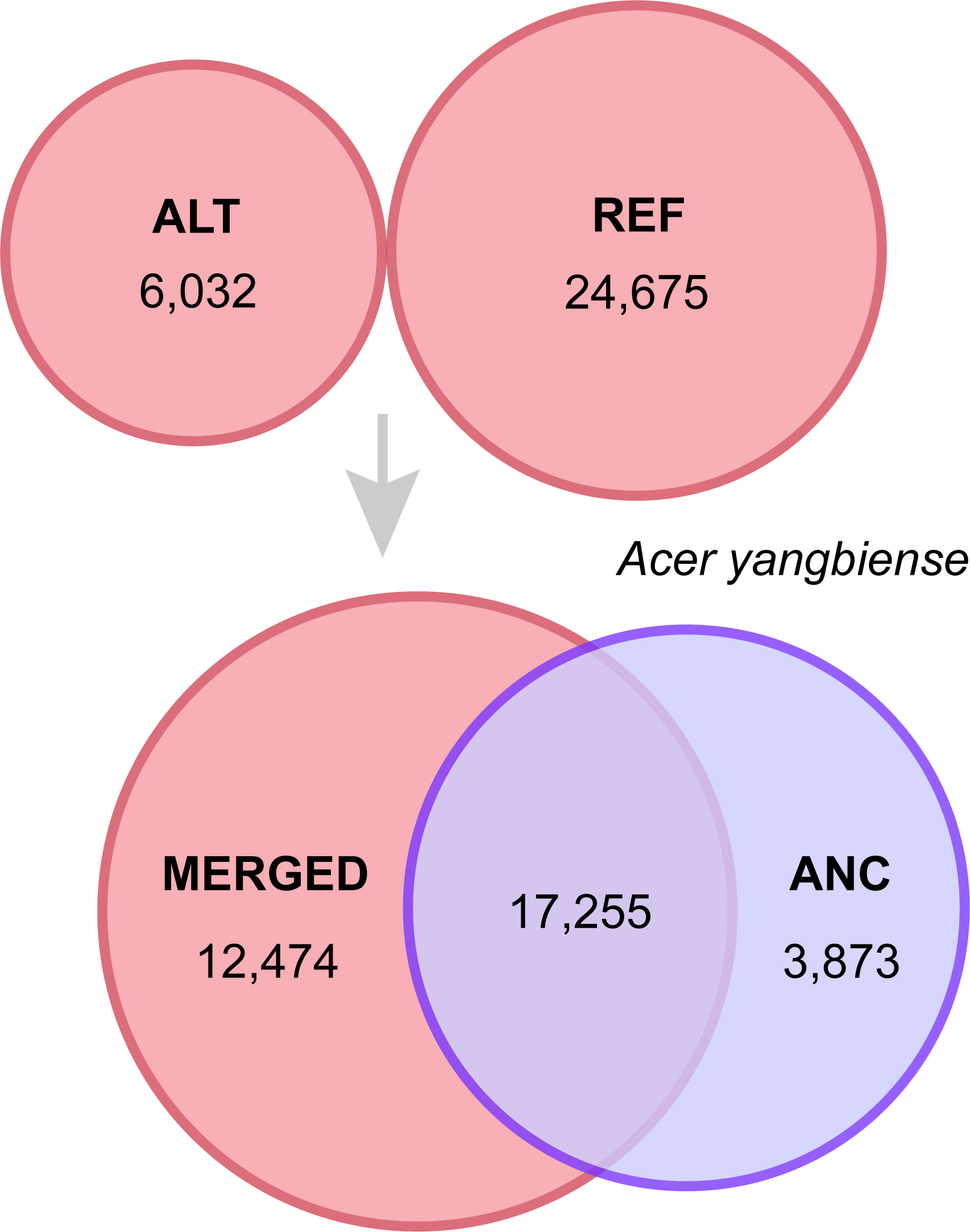
**

**Figure S13.** Differences in the number of deleterious mutations detected by REF-ALT strategy and ancestral status-based strategy of *Malania oleifera* (a) and *Acer yangbiense* (b)*.*

*
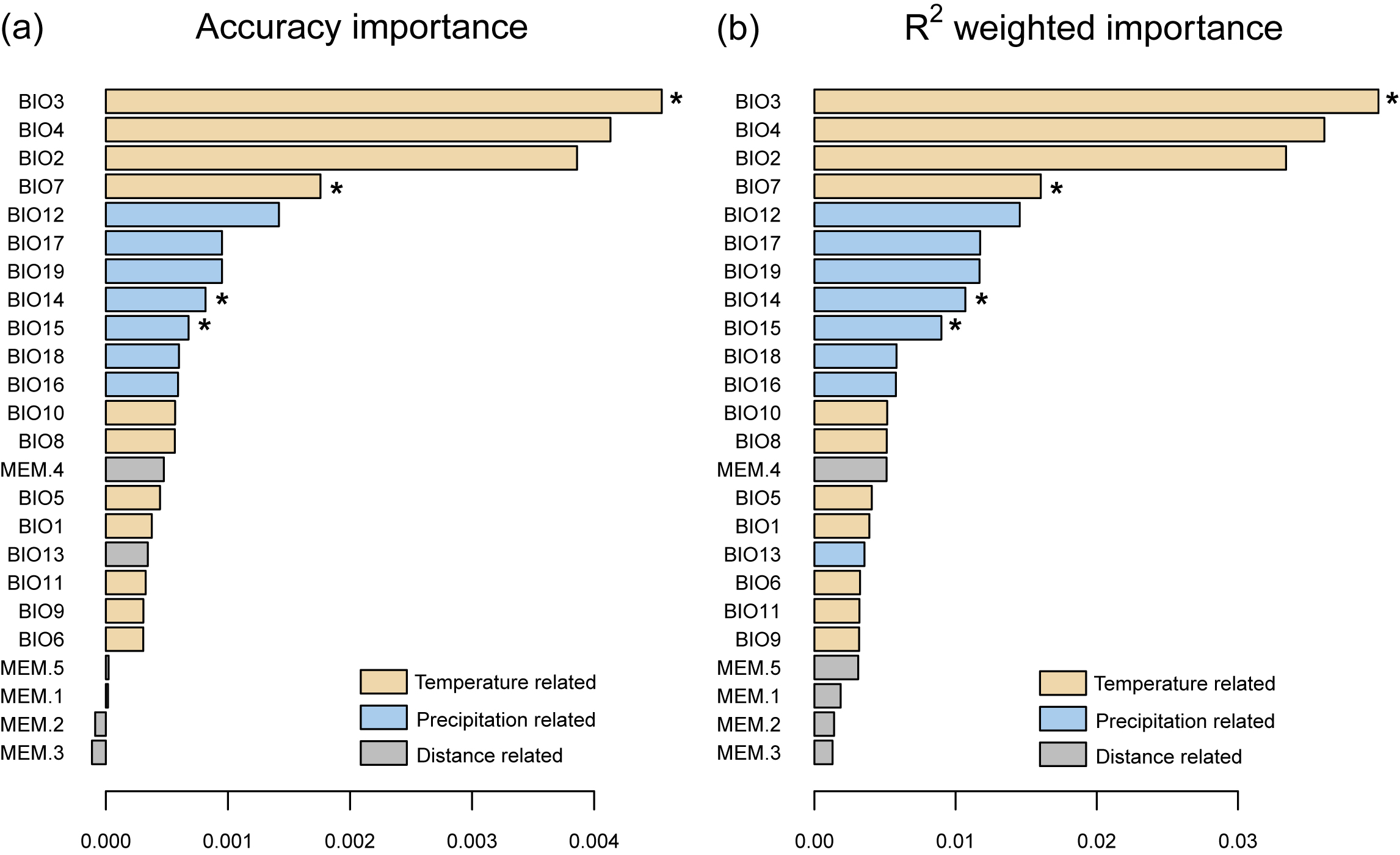
*

**Figure S14.** The importance of environmental variables inferred by gradient forest modeling. *Top uncorrelated environment variables (Pearson's |r| < 0.7) used in BayeScEnv, RDA and GF analysis.


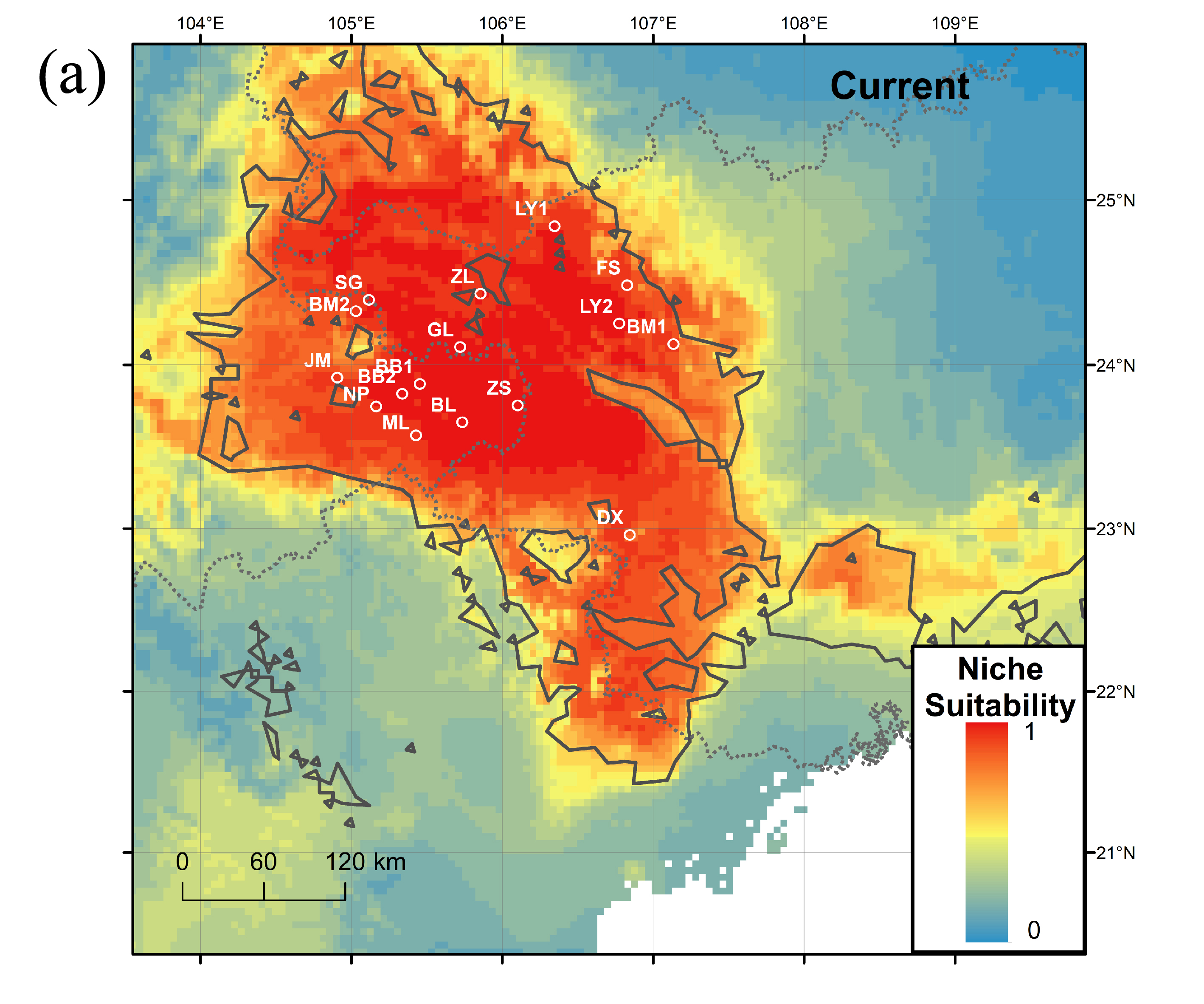

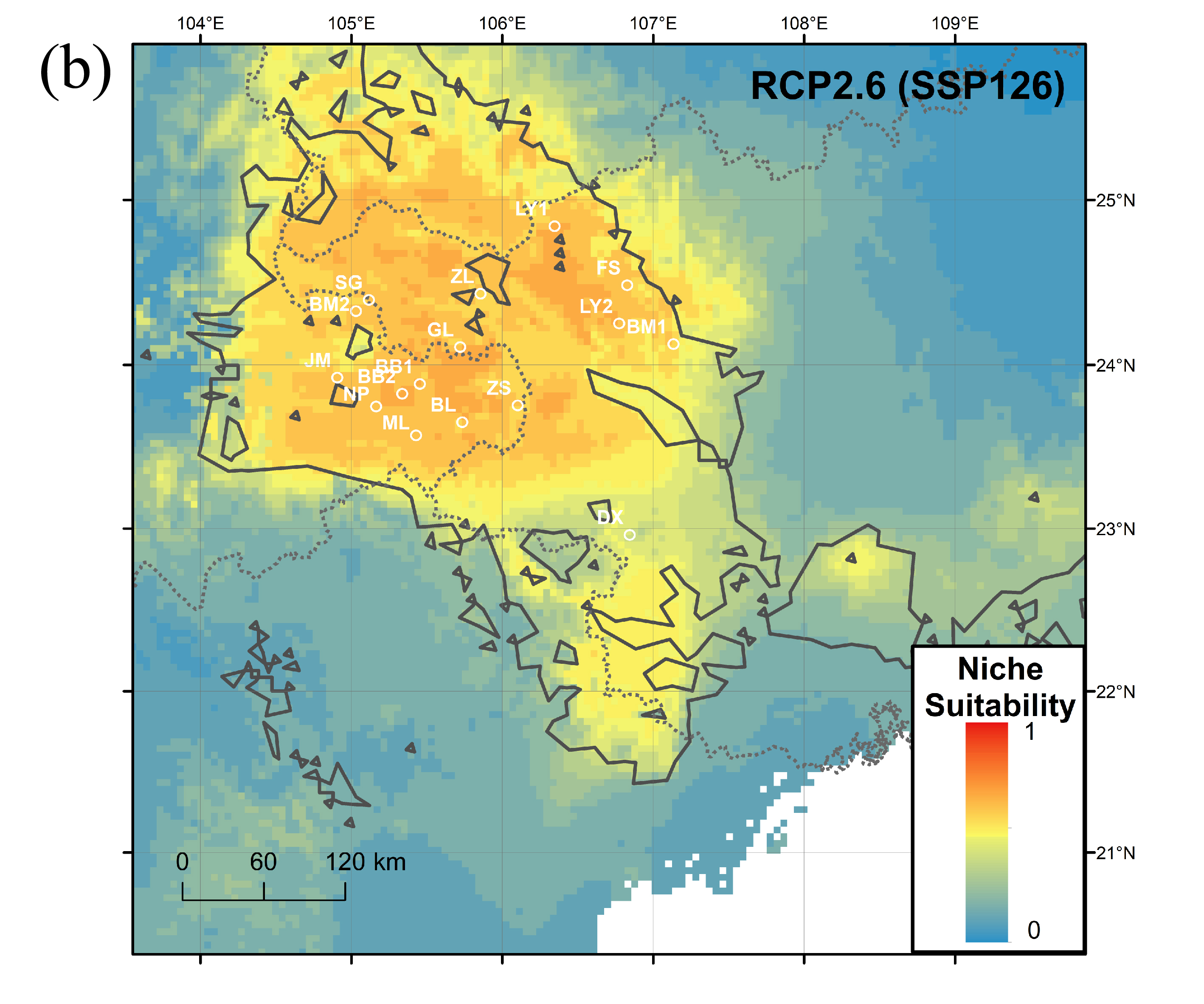

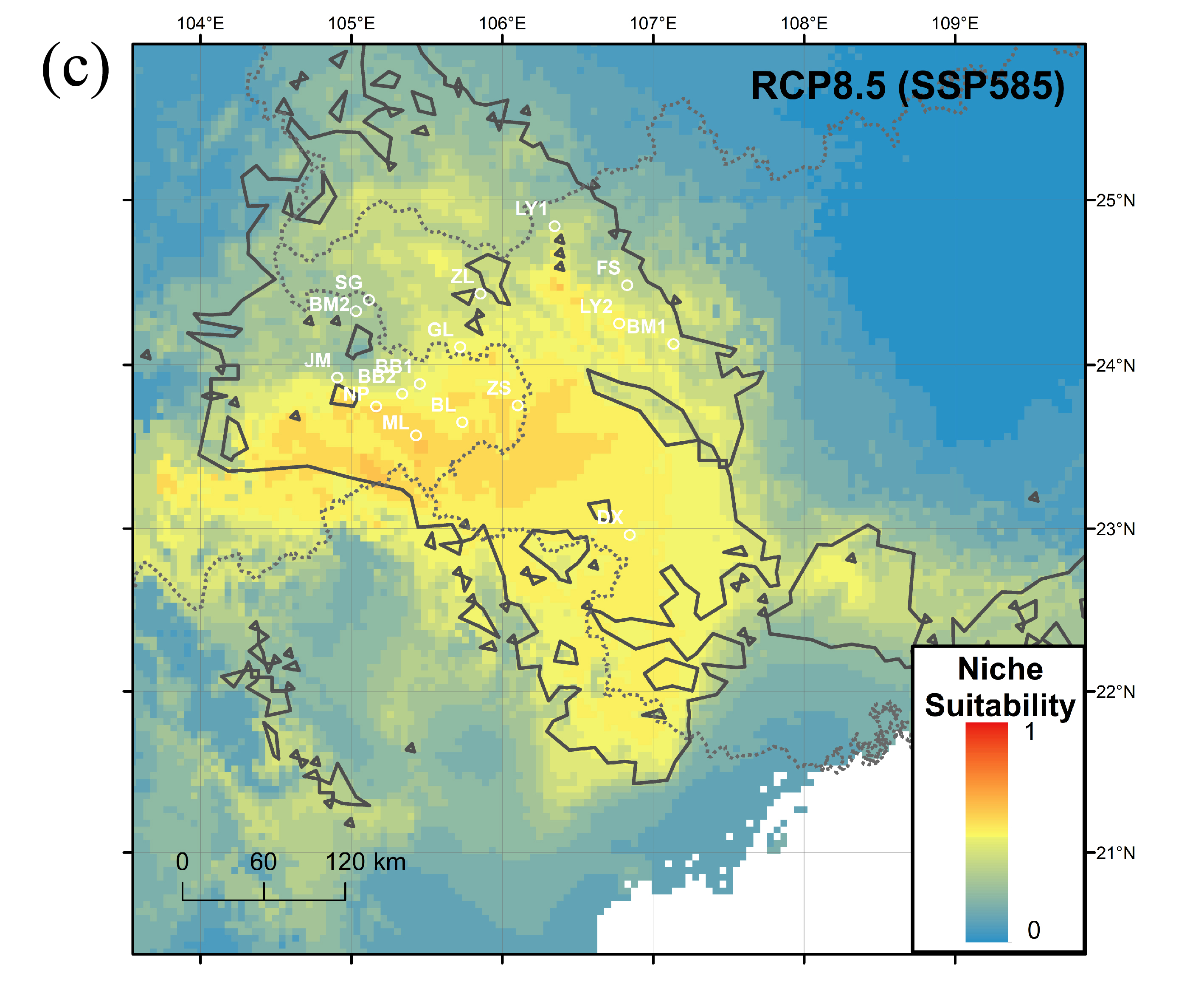


**Figure S15.** Integrated results of ecological niche modelling based on five models in the current (a) and future SSP126 (b) and SSP585 (c) scenarios. Higher values represent higher suitability.

**
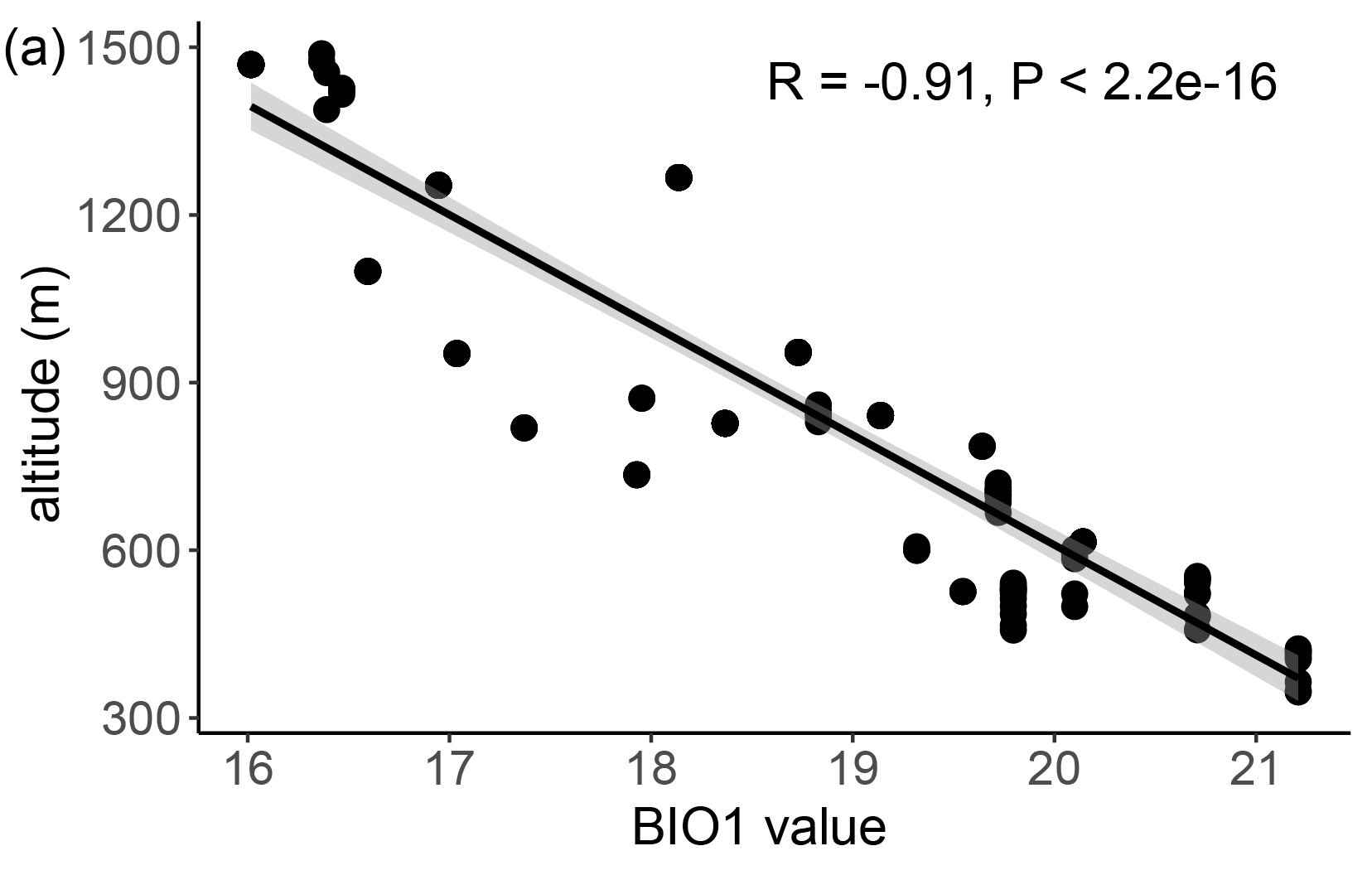

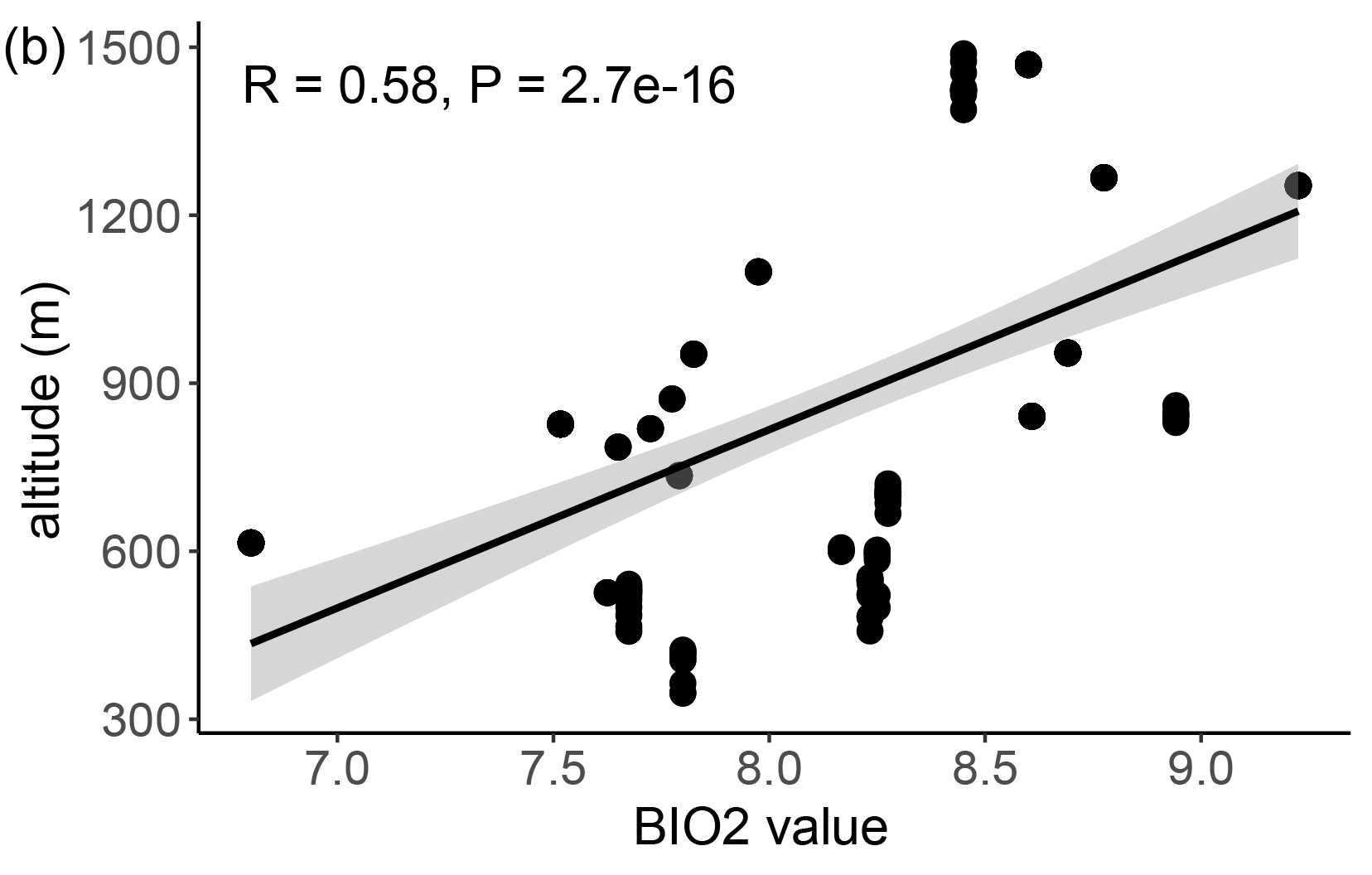
**

**
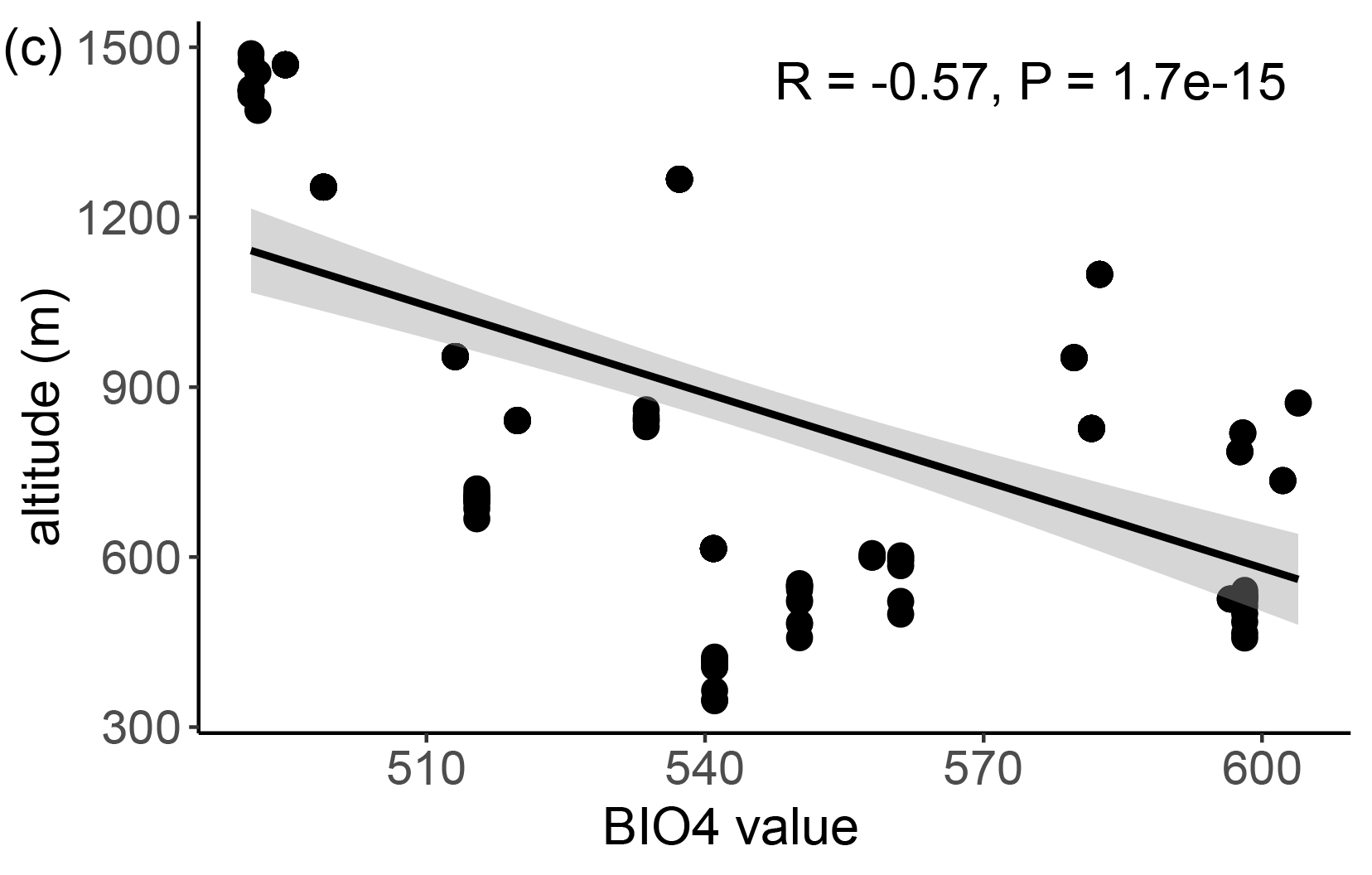

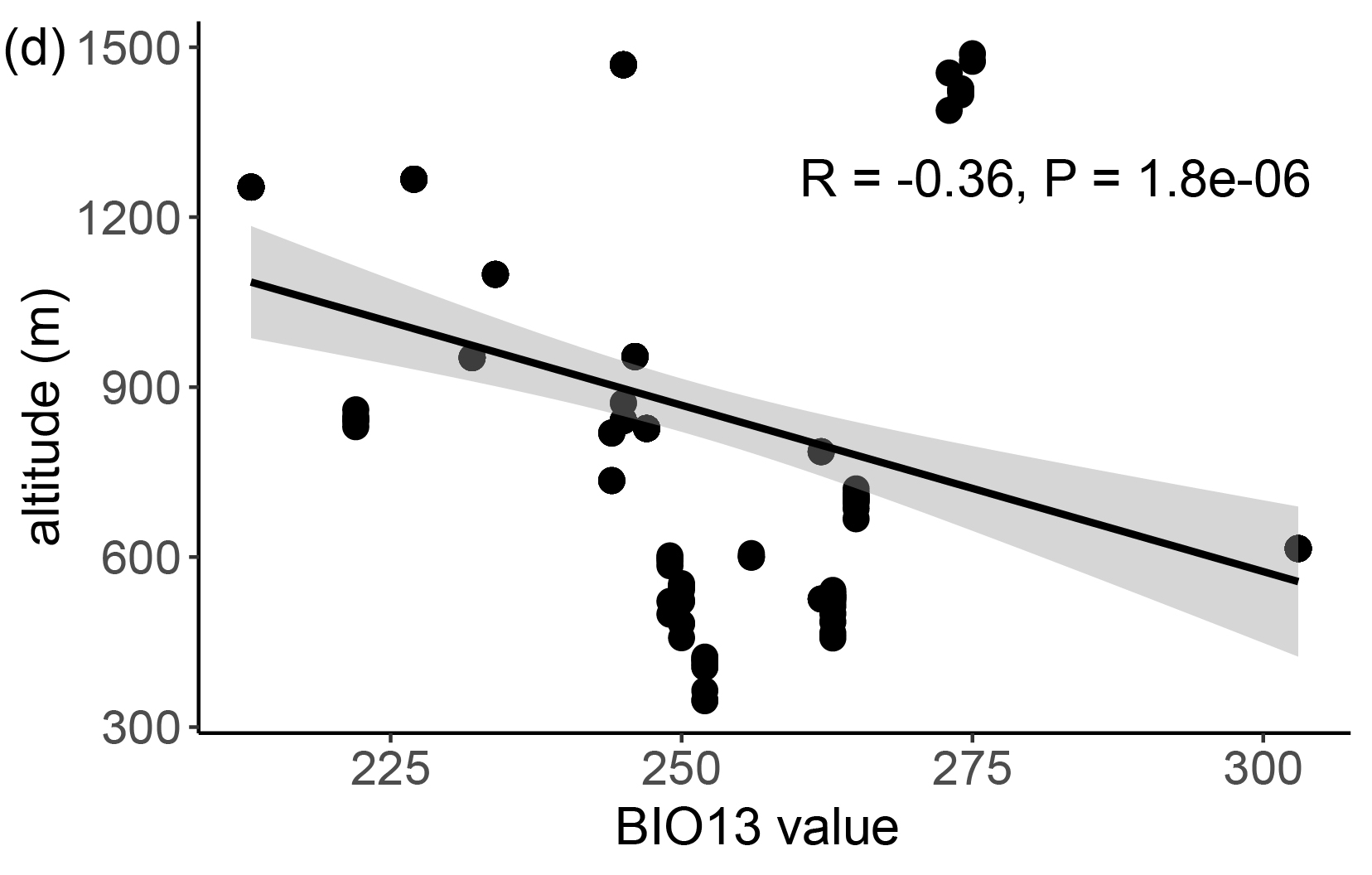
**

**
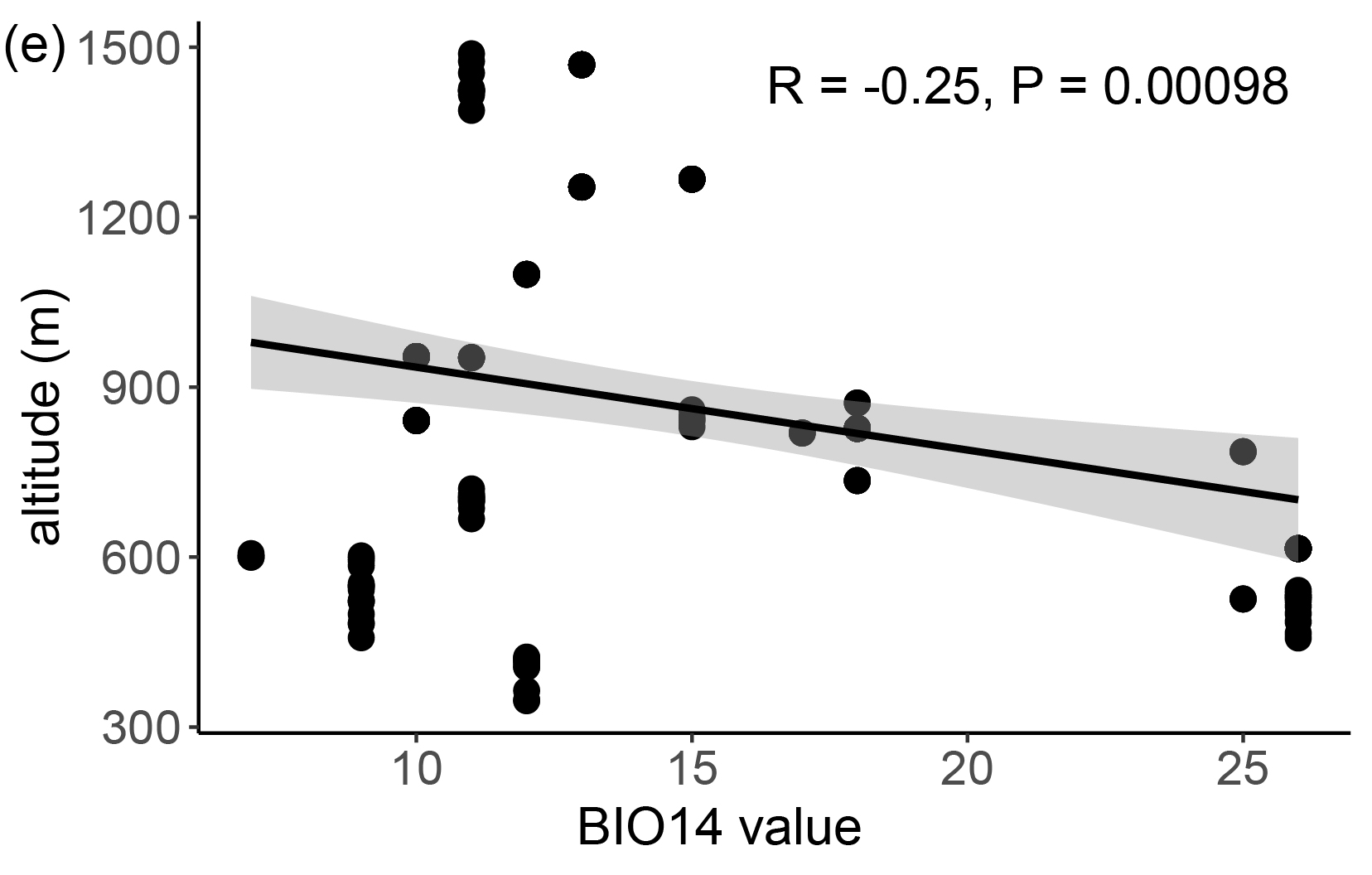
**

**Figure S16.** Correlation of altitude with BIO1, BIO2, BIO4, BIO13 and BIO14 climate variables used in GO analysis.
