# Supplementary material for: Genomic insights into endangerment and conservation of the garlic-fruit tree (Malania oleifera), a plant species with extremely small populations
Source: Gigascience. 2024 Sep 23;13:giae070. doi: 10.1093/gigascience/giae070 (PMC11417964; doi:10.1093/gigascience/giae070)
Supplement: giae070_Supplemental_Files [file giae070_supplemental_files.zip › Supplementary note S1-S3.docx]

**Supplementary note S1. Ancestral sequence reconstruction.**

The ancestral sequence of *M. oleifera* was reconstructed based on the resequencing data of representative individuals of 16 populations (Table S13). We mapped the sequences to the reference genome, transformed the format and removed duplicates, just as the process in section 2.2. We then used Freebayes v. 1.3.6 (Garrison & Marth, 2012) to call genotypes with the similar parameters mentioned in section 2.2, aside from the extra parameter “--report-monomorphic” to genotype all sites including variants and non-variants. Sites with genotyping quality less than 20, with sequencing depth less than three or greater than 500 were defined as missing and labeled as “N”. And indels were removed (marked as “N”). We obtained a total of 119,436 SNPs with a missing rate < 20%. Based on these high-quality SNPs, we implemented IQ-TREE v. 1.6.12 (Nguyen et al., 2015) to construct a guide tree for ancestral state reconstruction, using ModelFinder (Kalyaanamoorthy et al., 2017) to determine the best fit substitution model with *Santalum album* (SRR5150443) as an outgroup. Then, an empirical Bayesian method implemented in IQ-TREE was executed to construct ancestral states for each site of each chromosome of *M. oleifera*. We redefined the ancestral state of each site based on the posterior probability produced by IQ-TREE: (1) sites with posterior probabilities ≥ 0.99 were considered to have high confidence; (2) sites with posterior probabilities < 0.99 were regarded as ambiguous loci and were marked as “N”; at the same time, (3) each site should be supported by at least two individuals, otherwise, it will be redefined as “N”. We obtained 1,420,500,229 high confidence loci of *M. oleifera* ancestral sequences, accounting for 95% of the total loci.

**Supplementary note S2. Estimation of mutation rate.**

Based on the genomes of 17 published species and *M. oleifera* (Table S14), we obtained a total of 201 single-copy orthologous protein sequences using OrthoFinder v. 2.3.14 (Emms & Kelly, 2019). Protein sequences alignment was performed using MAFFT (Katoh & Standley, 2013), and the aligned protein sequences were then converted to corresponding codon alignments using PAL2NAL v. 14.1 (Suyama et al., 2006). A maximum likelihood phylogenetic tree was constructed using IQ-TREE v. 1.6.12 (Nguyen et al., 2015). Species divergence times were estimated using the MCMCTree module in PAML v. 4.10.0 (Yang, 2007). The molecular clock model used independent rates (clock=2) and the nucleotide substitution model was GTR. The MCMC chain with the first 100,000 iterations were discarded as burn-in, and then samples were taken every 100 iterations for a total of 100,000 times. We used four fossil calibration points: Stem Santalales (>65.5 Mya), Crown Ericales (>89.3 Mya), Stem Solanaceae (>33.9 Mya) and Stem Brassicales (>89.3 Mya). Finally, we estimated the neutral substitution rate of *M. oleifera* based on fourfold degenerate sites, and the mutation rate was approximately 2.5e-9 per site per year.

**Supplementary note S3. Detection of deleterious mutations based on REF-ALT strategy.**

The accumulation of deleterious mutations of *M. oleifera* were predicted using SIFT4G (Vaser et al., 2016). Initially, ancestral sequences were used as the SIFT prediction database referring to the method of Ma et al. (2022). But the results showed only a few homozygous deleterious mutations were detected, with an average of five per sample. Therefore, we adopted a relative strategy, assuming either the reference (REF) or alternative (ALT) state of each site as ancestral states which were used as SIFT prediction reference, respectively. This REF-ALT approach abandoned the assumption that ancestral states are harmless, however, it adopted the premise that relatively harmless sites are ancestral states. Thus, the two prediction results were merged by only retaining lower SIFT score for each site (Table S8).

In order to confirm the reliability of this method, the deleterious mutations of *Acer yangbiense* (Ma et al., 2022) were re-predicted according to the REF-ALT strategy and 29,729 deleterious mutations (SIFT score <0.05) were detected compared to 21,128 (ANC, SIFT score <0.05) using the original method which adopted the precondition that ancestral states are harmless (Figure S11b). The two-approach shared 17,255 sites, accounting for 81.67% of ANC, indicating that this strategy has high reliability.

**References**

Emms, D. M., & Kelly, S. (2019). OrthoFinder: phylogenetic orthology inference for comparative genomics. *Genome Biol, 20*(1), 238. doi:10.1186/s13059-019-1832-y

Garrison, E., & Marth, G. (2012). Haplotype-based variant detection from short-read sequencing. *arXiv:1207.3907 [q-bio.GN]*. doi:10.48550/arXiv.1207.3907

Kalyaanamoorthy, S., Minh, B. Q., Wong, T. K. F., von Haeseler, A., & Jermiin, L. S. (2017). ModelFinder: fast model selection for accurate phylogenetic estimates. *Nat Methods, 14*(6), 587-589. doi:10.1038/nmeth.4285

Katoh, K., & Standley, D. M. (2013). MAFFT multiple sequence alignment software version 7: improvements in performance and usability. *Mol Biol Evol, 30*(4), 772-780. doi:10.1093/molbev/mst010

Ma, Y., Liu, D., Wariss, H. M., Zhang, R., Tao, L., Milne, R. I., & Sun, W. (2022). Demographic history and identification of threats revealed by population genomic analysis provide insights into conservation for an endangered maple. *Mol Ecol, 31*(3), 767-779. doi:10.1111/mec.16289

Nguyen, L. T., Schmidt, H. A., von Haeseler, A., & Minh, B. Q. (2015). IQ-TREE: a fast and effective stochastic algorithm for estimating maximum-likelihood phylogenies. *Mol Biol Evol, 32*(1), 268-274. doi:10.1093/molbev/msu300

Suyama, M., Torrents, D., & Bork, P. (2006). PAL2NAL: robust conversion of protein sequence alignments into the corresponding codon alignments. *Nucleic Acids Res, 34*(Web Server issue), W609-612. doi:10.1093/nar/gkl315

Vaser, R., Adusumalli, S., Leng, S. N., Sikic, M., & Ng, P. C. (2016). SIFT missense predictions for genomes. *Nat Protoc, 11*(1), 1-9. doi:10.1038/nprot.2015.123

Yang, Z. (2007). PAML 4: phylogenetic analysis by maximum likelihood. *Mol Biol Evol, 24*(8), 1586-1591. doi:10.1093/molbev/msm088
