# Supplementary material for: Genomic insights into endangerment and conservation of the garlic-fruit tree (Malania oleifera), a plant species with extremely small populations
Source: Gigascience. 2024 Sep 23;13:giae070. doi: 10.1093/gigascience/giae070 (PMC11417964; doi:10.1093/gigascience/giae070)
Supplement: giae070_Response_to_Reviewer_Comments_Original_Submission [file giae070_response_to_reviewer_comments_original_submission.pdf]

Author's Response To Reviewer Comments

Reviewer #1: This article presents a fascinating case that combines genomic and ecological vulnerability to help protect endangered plants. I enjoyed the analyses, particularly the use of genomic offset to predict genomic vulnerability. This approach takes into account the entire genome's environment-related variations, rather than focusing on single SNP. I have a few minor comments that could improve the manuscript's readability.
Response: Thanks for the positive assessment. We have revised our manuscript according to your below comments.
1. According to general reading customs, readers are usually interested in understanding the population structure before delving into population factors such as LD, nucleotide diversity, and heterozygosity at the population level. Therefore, I suggest that the author swap the order of the first two paragraphs in the results section to provide a better flow of information.
Response: Thanks for your advice. We have swapped the order of the first two paragraphs in the results section. Please see page 8 for details.
2. It would be helpful to use three-letter abbreviations for the populations. The current use of two-letter abbreviations, such as BB1 and BB2 or BM1 and BM2, can lead to confusion since these populations were not closely related geographically.
Response: Thanks for pointing out this deficiency. To keep consistency with the name of raw data that we have already uploaded to NCBI, we still use the current naming convention. To avoid confusing readers, we have provided detailed descriptions of the abbreviations for each population in the legend of figure 1. See page 26, line 10-12.
3. Since "GO" represents genomic offset throughout the article, I recommend deleting the repeated abbreviation "GO" for gene ontology in the title of Supplementary Table S12.
Response: Revised.
4. Are there any references that support the selection of low Fst populations for genetic rescue? Alternatively, is there weak reproductive isolation among populations with high Fst? If not, why not consider using populations with high Fst and lower deleterious accumulations? High Fst combination will generate a high heterozygosity, which will greatly use heterozygous advantage. So, the second reason (Page 14: Lines 23-24) you provided does not persuade me, and I would appreciate additional clarification or references to support the chosen approach.
Response: Thanks for the reviewer’s constructive comment. Without knowing the changing relationships between Fst and reproductive isolation among populations in advance, it is not rigorous to propose genetic rescue program. In some cases, high Fst combination among parents generated offspring with high heterozygosity, which may produce heterozygous advantage, however, in most cases, outbreeding depression occurred (Frankham et al., 2010). It may occur between interpopulation crossing even in the same species (Severns, 2013). So, to minimize the risk of outbreeding depression, we do not consider DX population as a potential pollen donor because it has highest Fst (~0.90) with JM population based on adaptive SNPs (see below), suggesting severely adaptive differentiation has occurred. Under comprehensive evaluation, we still propose BM1 population as the prior pollen donor for JM population, because (1) it displays moderate genetic differentiation (Fst = 0.51) with JM population based on adaptive SNPs; (2) it has the highest genetic diversity and heterozygosity; and (3) it has the lowest inbreeding level and fewest shared homozygous deleterious mutations with JM population. See more details in page 14-15 and page 9, line 15-25.

Reviewer #2: In the study "Genomic insights into endangerment and conservation of the garlic-fruit tree (Malania oleifera), a plant species with extremely small populations" the authors examine an at risk species under upcoming climate models and advise on potential strategies to ensure its survival. The authors point out the plant’s usefulness to people, nervonic acid, however this is not the main motivation presented for the protection of this species, rather its ecological importance is. This is a viewpoint I advocate for, too much attention can be given to human important species to the exclusion of other study species. The manuscript’s introduction effectively introduces the study species, study goals, and motivation for study. All the analysis performed make sense and support the study goals. The presentation of the results was clear and methodical. The discussion was thoughtful and supported by the results. Furthermore, the

discussion made clear how the conclusions should be viewed "to provide practical and meaningful suggestions for the conservation actions".
Response: Thanks for the positive assessment of our manuscript. Hopefully these comments are satisfactorily addressed in the revised manuscript.
1. My only criticism of this manuscript is that it lacks details on the Malania oleifera genome and SNP distribution. While referenced, the manuscript doesn't provide the genome size, ploidy level, or assembly status. This makes it difficult to assess the significance of the reported SNP count (43,413,408 in dataset 1). After filtering the SNP set is heavily reduced (as expected due to the low number of samples and small population sizes), again is this a lot of SNPs, what percent of the genome does this represent?
Response: Thanks for your advice. We have added the information of genome size, ploidy level, and assembly status of M. oleifera. See page 3, line 6 and page 4, line 10. Moreover, after strict quality controlling based on 43,413,408 initial variant sites, we got 250,362 SNPs (dataset 2) and showed the distribution of these SNPs across 13 pseudochromosomes of M. oleifera. These SNPs covered most genome regions and can well represent genome-wide variation. Please see the newly added supplementary figure S2 for details.

Reviewer #3: This study employs comprehensive conservation genomic analysis to investigate the endangerment and conservation of Malania oleifera. The manuscript is well-written, and the authors have utilized appropriate techniques and data analyses. Their results will provide a solid foundation for management efforts. However, I have several concerns about this manuscript.
Response: Thanks for the positive assessment of our manuscript. We have considered your suggestions seriously and hope our revised manuscript can fully address your concerns.
1. Page 3 Line 40, "The number of individuals collected per population varied from 5 to 17, and if populations contained fewer than ten individuals, all individuals were sampled." This sentence needs clarification. Are you referring to samples from the germplasm bank?
Response: Sorry for the unclear description. We have clarified the sentence. See page 3, line 39-40.
2. Page 4 Line 15, Page 5 lines 2-4,7, If the dataset was not used in the subsequent analysis, it does not need to be named. Only the datasets used in the following analysis should have a name. Therefore, the authors may consider reducing the naming of datasets.
Response: Thanks for your suggestion. We have deleted the redundant name of dataset across the whole manuscript.
3. Page 4 Line 29, Which BAM files did you use for the diversity analysis? Please clarify.
Response: Sorry for the unclear description. The diversity analysis was performed by ANGSD v. 0.921 which used binary alignment map (BAM) format file of each sample as input. To prepare the input BAM files, we firstly used BWA v. 2.1 to align paired-end clean reads to the genome of M. oleifera. And then used SAMtools v. 1.9 to convert sequence alignment map (SAM) format files to sorted BAM files. Finally, we employed Sambamba v.0.7.1 to mark and remove duplicate reads. We have clarified the sentence. See page 4, line 9-13 and line 29.
4. Page 4 Line 38, The methods you used are both based on Fst, so they are not very distinct.
Response: Revised. See page 4, line 37.
5. Page 5 Line 20, Reconstructing the ancestral sequence is not easy. The Stairway Plot v.2 can now use folded SFS to perform demographic analysis.
Response: Thanks for your advice. In the revised manuscript, we used both folded and unfolded SFS to infer demographic history of M. oleifera by Stairway Plot v.2. The two results showed similar bottleneck events at the corresponding time. See Figure 2a, Figure S10, line 18-22 in page 5 and line 27-33 in page 9.
6. Page 6 Lines 15-20, Selecting variables based solely on the correlation between them seems somewhat arbitrary. It would be more robust to first use analyses such as gradient forest to rank the importance of all the variables. Then, select variables based on the results of variable importance and their correlations. For the environment-associated analysis, you may consider skipping this step, as there are no model overfitting issues.
Response: Thanks for pointing out the shortcoming. As you advised, we ranked the climatic factors based on weighted R^2 importance using gradient forest model and selected four most important and uncorrelated factors (Pearson's $|r| < 0.7$), including BIO3, BIO7, BIO14 and BIO15 for BayeScEnv, RDA and GF analysis. See Figure S14 and page 6, line 16-23.
7. Page 8 Lines 23-26, The values of mean heterozygosity rate should be converted into percentages.
Response: Revised. See page 9, line 6-9.

8. Page 10 Lines 15-17, The conclusion that "we found that populations with severe inbreeding also had stronger genetic load, but experienced weaker purifying selection against nonsynonymous mutations" is not very straightforward. You need to provide more explanation. Additionally, the ratio of 0-fold to 4-fold degenerate nucleotide diversity (π0/π4) is commonly used as an index of purifying selection efficiency.
Response: We are sorry for the unclear description. Relevant description has been verified. See page 10, line 25-29. We used the ratio of 0-fold/4-fold degenerate sites heterozygosity to intergenic (neutral) heterozygosity as an index of purifying selection efficiency referring to the method of Yang et al (2018) and Liu et al (2022). Significant negative correlation implied that severely deleterious mutations can be effectively purged, which may be the maintaining mechanism of M. oleifera with small population size.
9. Page 10 Lines 32-33, Each outlier detection method has a proportion of false positives (FP). When you combine the outliers from different methods, the FP rate might be even higher. How do you control for this?
Response: Thanks for pointing out the deficiency. To avoid false positives, we assigned the 380 SNPs that detected by both BayeScEnv and RDA as environment-associated SNPs (See page 11, line 1-5). And we re-inferred genomic offset (GO) using R package gradientforest v.0.1-37, also found a strong negative correlation between GO and altitude, with populations at higher altitudes generally having lower GO values (see Figure 4 and Table S13). The result is similar compared to the previous result based on all 1,156 SNPs detected by BayeScEnv and RDA, which highlight the reliability of our methods.
10. One suggestion you may consider: combine the results of niche modeling and genetic offset analysis to infer climate change-driven vulnerability. For more information, see this article (https://www.nature.com/articles/s41467-022-32546-z).
Response: Thanks for your advice. The article you mentioned is a very important reference for us and we have tried to combine the results of niche modeling and genetic offset by calculate genome-niche index (gni). Following the methods of Chen et al (2022), to detect populations that are least interrupted by future climate change, only the niches of increasing future suitability were considered because populations in these areas would not be challenged by niche suitability decline. However, in our case, niches with increasing future suitability were lacking and all sampling sites will have decreased niche fitness (see Figure 5). So, we think this method is not suitable for M. oleifera to combine genomic offset and niche suitability change.

References
Chen Y, Jiang Z, Fan P, et al. The combination of genomic offset and niche modelling provides insights into climate change-driven vulnerability. Nat Commun 2022;13(1):4821. doi:10.1038/s41467-022-32546-z.
Frankham R, Ballou JD, Eldridge MD, et al. Predicting the probability of outbreeding depression. Conserv Biol 2011;25(3):465-75. doi: 10.1111/j.1523-1739.2011.01662.x.
Liu S, Zhang L, Sang Y, et al. Demographic history and natural selection shape patterns of deleterious mutation load and barriers to introgression across populus genome. Mol Biol Evol 2022;39(2):msac008. doi: 10.1093/molbev/msac008.
Severns PM. Precautionary hand pollination suggests outbreeding depression between potential seed donor populations for a rare wetland plant. J Torrey Bot Soc 2013;140(1), 20-25. doi: 10.3159/TORREY-D-12-00046.1.
Yang Y, Ma T, Wang Z, et al. Genomic effects of population collapse in a critically endangered ironwood tree Ostrya rehderiana. Nat Commun 2018;9(1):5449. doi:10.1038/s41467-018-07913-4.
