# Supplementary material for: Genomic insights into endangerment and conservation of the garlic-fruit tree (Malania oleifera), a plant species with extremely small populations
Source: Gigascience. 2024 Sep 23;13:giae070. doi: 10.1093/gigascience/giae070 (PMC11417964; doi:10.1093/gigascience/giae070)
Supplement: giae070_Reviewer_1_Report_Original_Submission — Yongzhi Yang, Ph.D. -- 6/24/2024 Reviewed [file giae070_reviewer_1_report_original_submission.pdf]

Reviewer Report

Title: Genomic insights into endangerment and conservation of the garlic-fruit tree (Malania oleifera), a plant species with extremely small populations

Version: Original Submission **Date:** 6/24/2024

Reviewer name: Yongzhi Yang, Ph.D.

Reviewer Comments to Author:

This article presents a fascinating case that combines genomic and ecological vulnerability to help protect endangered plants. I enjoyed the analyses, particularly the use of genomic offset to predict genomic vulnerability. This approach takes into account the entire genome's environment-related variations, rather than focusing on single SNP. I have a few minor comments that could improve the manuscript's readability:

1, According to general reading customs, readers are usually interested in understanding the population structure before delving into population factors such as LD, nucleotide diversity, and heterozygosity at the population level. Therefore, I suggest that the author swap the order of the first two paragraphs in the results section to provide a better flow of information.

2, It would be helpful to use three-letter abbreviations for the populations. The current use of two-letter abbreviations, such as BB1 and BB2 or BM1 and BM2, can lead to confusion since these populations were not closely related geographically.

3, Since "GO" represents genomic offset throughout the article, I recommend deleting the repeated abbreviation "GO" for gene ontology in the title of Supplementary Table S12.

4, Are there any references that support the selection of low Fst populations for genetic rescue? Alternatively, is there weak reproductive isolation among populations with high Fst? If not, why not consider using populations with high Fst and lower deleterious accumulations? High Fst combination will generate a high heterozygosity, which will greatly use heterozygous advantage. So, the second reason (Page 14: Lines 23-24) you provided does not persuade me, and I would appreciate additional clarification or references to support the chosen approach.

Methods

Are the methods appropriate to the aims of the study, are they well described, and are necessary controls included? Choose an item.

Conclusions

Are the conclusions adequately supported by the data shown? Choose an item.

Reporting Standards

Does the manuscript adhere to the journal's guidelines on minimum standards of reporting? Choose an item.

Choose an item.

Statistics

Are you able to assess all statistics in the manuscript, including the appropriateness of statistical tests used? Choose an item.

Quality of Written English

Please indicate the quality of language in the manuscript: Choose an item.

Declaration of Competing Interests

Please complete a declaration of competing interests, considering the following questions:

- Have you in the past five years received reimbursements, fees, funding, or salary from an organisation that may in any way gain or lose financially from the publication of this manuscript, either now or in the future?
- Do you hold any stocks or shares in an organisation that may in any way gain or lose financially from the publication of this manuscript, either now or in the future?
- Do you hold or are you currently applying for any patents relating to the content of the manuscript?
- Have you received reimbursements, fees, funding, or salary from an organization that holds or has applied for patents relating to the content of the manuscript?
- Do you have any other financial competing interests?
- Do you have any non-financial competing interests in relation to this paper?

If you can answer no to all of the above, write 'I declare that I have no competing interests' below. If your reply is yes to any, please give details below.

I declare that I have no competing interests

I agree to the open peer review policy of the journal. I understand that my name will be included on my report to the authors and, if the manuscript is accepted for publication, my named report including any attachments I upload will be posted on the website along with the authors' responses. I agree for my report to be made available under an Open Access Creative Commons CC-BY license (http://creativecommons.org/licenses/by/4.0/). I understand that any comments which I do not wish to be included in my named report can be included as confidential comments to the editors, which will not be published.

Choose an item.

To further support our reviewers, we have joined with Publons, where you can gain additional credit to further highlight your hard work (see: https://publons.com/journal/530/gigascience). On publication of this paper, your review will be automatically added to Publons, you can then choose whether or not to claim your Publons credit. I understand this statement.

Yes Choose an item.
