# Supplementary material for: Genomic insights into endangerment and conservation of the garlic-fruit tree (Malania oleifera), a plant species with extremely small populations
Source: Gigascience. 2024 Sep 23;13:giae070. doi: 10.1093/gigascience/giae070 (PMC11417964; doi:10.1093/gigascience/giae070)
Supplement: giae070_Reviewer_2_Report_Original_Submission — Scott Ferguson -- 6/26/2024 Reviewed [file giae070_reviewer_2_report_original_submission.pdf]

Reviewer Report

Title: Genomic insights into endangerment and conservation of the garlic-fruit tree (Malania oleifera), a plant species with extremely small populations

Version: Original Submission **Date:** 6/26/2024

Reviewer name: Scott Ferguson

Reviewer Comments to Author:

In the study "Genomic insights into endangerment and conservation of the garlic-fruit tree (Malania oleifera), a plant species with extremely small populations" the authors examine an at risk species under upcoming climate models and advise on potential strategies to ensure its survival. The authors point out the plants usefulness to people, nervonic acid, however this is not the main motivation presented for the protection of this species, rather its ecological importance is. This is a viewpoint I advocate for, to much attention can be given to human important species to the exclusion of other study species.
The manuscripts introduction effectively introduces the study species, study goals, and motivation for study. All the analysis performed make sense and support the study goals. The presentation of the results was clear and methodical. The discussion was thoughtful and supported by the results. Furthermore, the discussion made clear how the conclusions should be viewed "to provide practical and meaningful suggestions for the conservation actions".
My only criticism of this manuscript is that it lacks details on the Malania oleifera genome and SNP distribution. While referenced, the manuscript doesn't provide the genome size, ploidy level, or assembly status. This makes it difficult to assess the significance of the reported SNP count (43,413,408 in dataset 1). After filtering the SNP set is heavily reduced (as expected due to the low number of samples and small population sizes), again is this a lot of SNPs, what percent of the genome does this represent?

Methods

Are the methods appropriate to the aims of the study, are they well described, and are necessary controls included? Choose an item.

Conclusions

Are the conclusions adequately supported by the data shown? Choose an item.

Reporting Standards

Does the manuscript adhere to the journal’s guidelines on minimum standards of reporting? Choose an item.

Choose an item.

Statistics

Are you able to assess all statistics in the manuscript, including the appropriateness of statistical tests used? Choose an item.

Quality of Written English

Please indicate the quality of language in the manuscript: Choose an item.

Declaration of Competing Interests

Please complete a declaration of competing interests, considering the following questions:

- Have you in the past five years received reimbursements, fees, funding, or salary from an organisation that may in any way gain or lose financially from the publication of this manuscript, either now or in the future?
- Do you hold any stocks or shares in an organisation that may in any way gain or lose financially from the publication of this manuscript, either now or in the future?
- Do you hold or are you currently applying for any patents relating to the content of the manuscript?
- Have you received reimbursements, fees, funding, or salary from an organization that holds or has applied for patents relating to the content of the manuscript?
- Do you have any other financial competing interests?
- Do you have any non-financial competing interests in relation to this paper?

If you can answer no to all of the above, write 'I declare that I have no competing interests' below. If your reply is yes to any, please give details below.

I declare that I have no competing interests

I agree to the open peer review policy of the journal. I understand that my name will be included on my report to the authors and, if the manuscript is accepted for publication, my named report including any attachments I upload will be posted on the website along with the authors' responses. I agree for my report to be made available under an Open Access Creative Commons CC-BY license (http://creativecommons.org/licenses/by/4.0/). I understand that any comments which I do not wish to be included in my named report can be included as confidential comments to the editors, which will not be published.

Choose an item.

To further support our reviewers, we have joined with Publons, where you can gain additional credit to further highlight your hard work (see: https://publons.com/journal/530/gigascience). On publication of this paper, your review will be automatically added to Publons, you can then choose whether or not to claim your Publons credit. I understand this statement.

Yes Choose an item.
