# Supplementary material for: Genomic insights into endangerment and conservation of the garlic-fruit tree (Malania oleifera), a plant species with extremely small populations
Source: Gigascience. 2024 Sep 23;13:giae070. doi: 10.1093/gigascience/giae070 (PMC11417964; doi:10.1093/gigascience/giae070)
Supplement: giae070_Reviewer_3_Report_Original_Submission — Wei Zhao -- 6/30/2024 Reviewed [file giae070_reviewer_3_report_original_submission.pdf]

Reviewer Report

Title: Genomic insights into endangerment and conservation of the garlic-fruit tree (Malania oleifera), a plant species with extremely small populations

Version: Original Submission **Date:** 6/30/2024

Reviewer name: Wei Zhao

Reviewer Comments to Author:

This study employs comprehensive conservation genomic analysis to investigate the endangerment and conservation of Malania oleifera. The manuscript is well-written, and the authors have utilized appropriate techniques and data analyses. Their results will provide a solid foundation for management efforts. However, I have several concerns about this manuscript.

Page 3 Line 40, "The number of individuals collected per population varied from 5 to 17, and if populations contained fewer than ten individuals, all individuals were sampled." This sentence needs clarification. Are you referring to samples from the germplasm bank?

Page 4 Line 15, Page 5 lines 2-4,7, If the dataset was not used in the subsequent analysis, it does not need to be named. Only the datasets used in the following analysis should have a name. Therefore, the authors may consider reducing the naming of datasets.

Page 4 Line 29, Which BAM files did you use for the diversity analysis? Please clarify.

Page 4 Line 38, The methods you used are both based on Fst, so they are not very distinct.

Page 5 Line 20, Reconstructing the ancestral sequence is not easy. The Stairway Plot v.2 can now use folded SFS to perform demographic analysis.

Page 6 Lines 15-20, Selecting variables based solely on the correlation between them seems somewhat arbitrary. It would be more robust to first use analyses such as gradient forest to rank the importance of all the variables. Then, select variables based on the results of variable importance and their correlations. For the environment-associated analysis, you may consider skipping this step, as there are no model overfitting issues.

Page 8 Lines 23-26, The values of mean heterozygosity rate should be converted into percentages.

Page 10 Lines 15-17, The conclusion that "we found that populations with severe inbreeding also had stronger genetic load, but experienced weaker purifying selection against nonsynonymous mutations" is not very straightforward. You need to provide more explanation. Additionally, the ratio of 0-fold to 4-fold degenerate nucleotide diversity (Ï€0/Ï€4) is commonly used as an index of purifying selection efficiency.

Page 10 Lines 32-33, Each outlier detection method has a proportion of false positives (FP). When you combine the outliers from different methods, the FP rate might be even higher. How do you control for this?

One suggestion you may consider: combine the results of niche modeling and genetic offset analysis to infer climate change-driven vulnerability. For more information, see this article (https://www.nature.com/articles/s41467-022-32546-z).

Methods

Are the methods appropriate to the aims of the study, are they well described, and are necessary controls included? Choose an item.

Conclusions

Are the conclusions adequately supported by the data shown? Choose an item.

Reporting Standards

Does the manuscript adhere to the journal's guidelines on minimum standards of reporting? Choose an item.

Choose an item.

Statistics

Are you able to assess all statistics in the manuscript, including the appropriateness of statistical tests used? Choose an item.

Quality of Written English

Please indicate the quality of language in the manuscript: Choose an item.

Declaration of Competing Interests

Please complete a declaration of competing interests, considering the following questions:

- Have you in the past five years received reimbursements, fees, funding, or salary from an organisation that may in any way gain or lose financially from the publication of this manuscript, either now or in the future?
- Do you hold any stocks or shares in an organisation that may in any way gain or lose financially from the publication of this manuscript, either now or in the future?
- Do you hold or are you currently applying for any patents relating to the content of the manuscript?
- Have you received reimbursements, fees, funding, or salary from an organization that holds or has applied for patents relating to the content of the manuscript?
- Do you have any other financial competing interests?
- Do you have any non-financial competing interests in relation to this paper?

If you can answer no to all of the above, write 'I declare that I have no competing interests' below. If your reply is yes to any, please give details below.

No

I agree to the open peer review policy of the journal. I understand that my name will be included on my report to the authors and, if the manuscript is accepted for publication, my named report including any attachments I upload will be posted on the website along with the authors' responses. I agree for my report to be made available under an Open Access Creative Commons CC-BY license (http://creativecommons.org/licenses/by/4.0/). I understand that any comments which I do not wish to be included in my named report can be included as confidential comments to the editors, which will not be published.

Choose an item.

To further support our reviewers, we have joined with Publons, where you can gain additional credit to further highlight your hard work (see: https://publons.com/journal/530/gigascience). On publication of this paper, your review will be automatically added to Publons, you can then choose whether or not to claim your Publons credit. I understand this statement.

Yes Choose an item.
